# Deoxyfluorination of Electron-Deficient Phenols

**DOI:** 10.1021/acs.orglett.3c01018

**Published:** 2023-05-16

**Authors:** Jan Jelen, Gašper Tavčar

**Affiliations:** † Department of Inorganic Chemistry and Technology, Jožef Stefan Institute, Jamova cesta 39, 1000 Ljubljana, Slovenia; ‡ Jožef Stefan International Postgraduate School, Jamova 39, 1000 Ljubljana, Slovenia

## Abstract

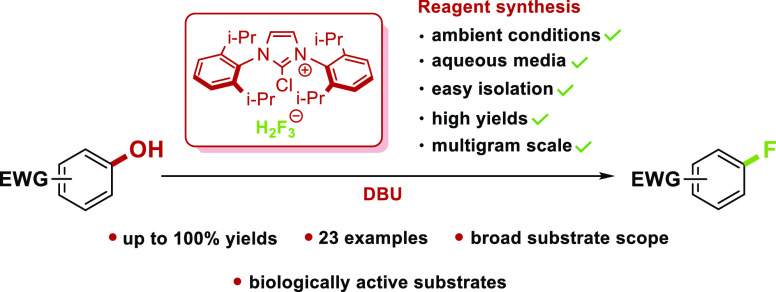

In this study, we report a facile synthesis of 2-chloro-1,3-bis(2,6-diisopropylphenyl)imidazolium salts in aqueous media under ambient conditions using hypochlorite as a chlorinating agent. In addition, an air-stable and moisture-insensitive deoxyfluorination reagent based on poly[hydrogen fluoride] salt is presented, which is capable of converting electron-deficient phenols or aryl silyl ethers into the corresponding aryl fluorides in the presence of DBU as a base, with good to excellent yields and high tolerance to functional groups.

Fluorine compounds have been extensively exploited in pharmaceutical chemistry, agricultural chemistry, and drug design owing to the unique properties of carbon–fluorine bonds.^1−4^ Incorporation of fluorine into a molecular skeleton can beneficially influence conformational changes resulting from gauche effect, p*K*_a_ value due to the electron-withdrawing properties of fluorine, membrane permeability due to increased lipophilicity, and metabolic pathways on account of the strong carbon–fluorine bond.^5^ Despite the great interest in fluorinated molecules, synthetic methodologies for the introduction of fluorine still remain challenging to date, and most fluorination reaction protocols lack cost-efficiency.^2^ One of the promising methods developed in the past decade for formation of a C–F bond is deoxyfluorination, a one-step nucleophilic substitution reaction between a hydroxyl (−OH) group and fluoride, since it uses readily available alcohols, phenols, and carboxylic acids as starting materials.^6^ Many reagents have been developed for deoxyfluorination reactions, most notably PyFluor,^6^ PhenoFluor,^7^ AlkylFluor,^8^ PhenoFluorMix,^9^ CpFluor,^10,11^ and SO_2_F_2_/TMAF reagent system.^12^ While developed deoxyfluorination reagents proved themselves very useful in terms of substrate scope and reaction yields, most of them require synthesis under inert conditions and the use of dry organic solvents or other expensive reagents.^6−12^ Therefore, our goal was to synthesize a prominent reagent based on known motifs. We identified the 2-chloro-1,3-bis(aryl)imidazolium moiety, which constitutes the reagent PhenoFluorMix, as a potential target. To date, its synthesis entails formation of an NHC carbene intermediate under an inert atmosphere in dry organic solvent using Schlenk techniques^9^ (Scheme 1).

**Scheme 1:**
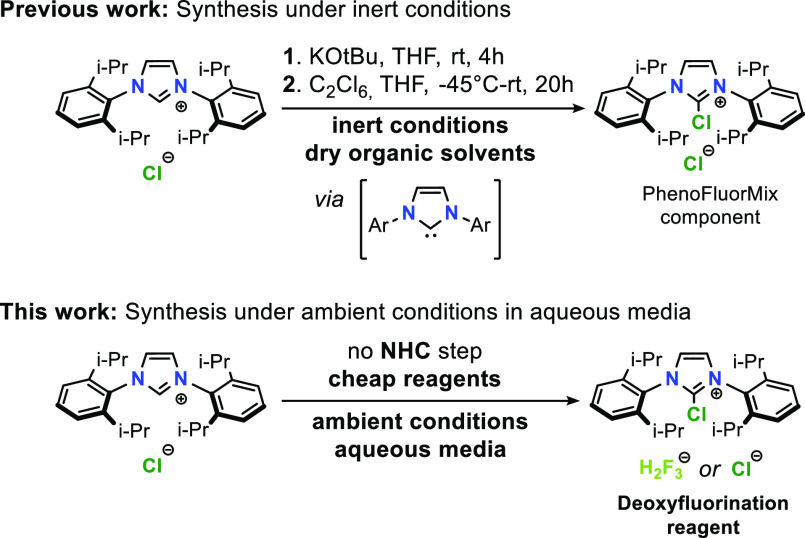
Synthesis of the 2-Chloro-1,3-bis(aryl)imidazolium Moiety

We reasoned that it would be feasible to furnish the desired C2 chlorination in one step, forming an active NHC carbene nucleophile in situ under basic conditions. Bleach solution (hypochlorite) is one of the most accessible reagents, which satisfies both needs: alkaline conditions and an electrophilic chlorine source. Formation of 2-imidazolones from the corresponding 2*H*-imidazolium salts using NaOCl in THF was previously reported,^13^ and 2-chloroimidazolium salt was suggested as a competent intermediate, which is susceptible to hydrolysis, affording 2-imidazolones (Scheme 2a). While hydroxide ions possess decent nucleophilicity in organic solvents, aqueous alkaline solutions display a much poorer nucleophilic profile, due to the strong hydrogen bonding capabilities of water. Therefore, we were prompted to isolate intermediate 2-chloro-1,3-bis(aryl)imidazolium salts from aqueous solutions. Treating 1,3-bis(2,6-diisopropylphenyl)imidazolium chloride (**1a**) with diluted bleach solution at room temperature yielded a white precipitate. NMR analysis of precipitate at various reaction times revealed instant formation of an insoluble 2*H*-imidazolium salt (**1b**) that slowly converts to 2-chloroimidazolium salt (**2a**) as a major product and 2-imidazolone (**3**) as a side product, presumptively formed due to the aforementioned hydrolysis of **2a**. X-ray single crystal structure analysis of crystals obtained from the insoluble precipitate identified the presence of chlorate(V) counterion, which was furthermore confirmed by Raman spectroscopy. Chlorate(V) ion is a common contaminant in bleach solutions formed as a byproduct of decomposition of hypochlorite ions.

**Scheme 2:**
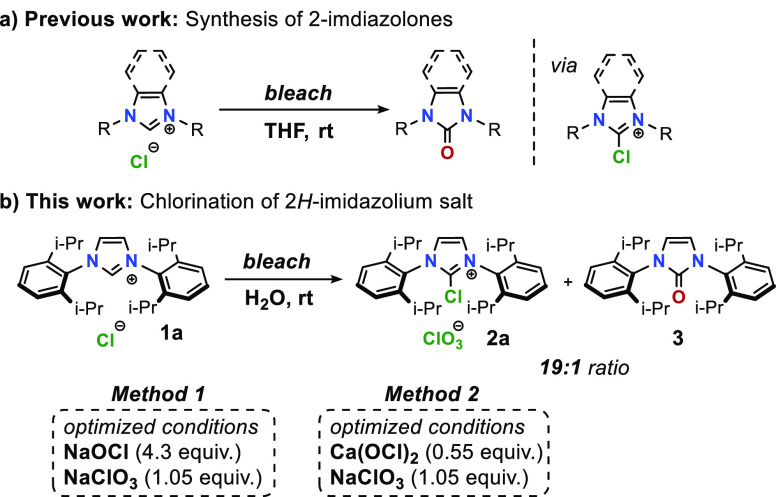
Oxidation of 2*H*-Imidazolium Salts with Bleach

Using the optimal reaction conditions (Table 1, entry 3) resulted in 89% conversion of **2a** and 88% isolated yield on 1 mmol scale (method 1). Note that an additional 1.05 equiv of NaClO_3_ was added before the addition of hypochlorite bleach solution in order to ensure complete precipitation of 2*H*-imidazolium salt (**1b**). While this chlorination reaction protocol works well on smaller scales (up to 5 mmol), a huge decline in reaction yields was observed on multigram scale (Table 1, entry 6). It was reasoned that the heterogeneous nature of the reaction (solid–liquid system) together with long reaction times in a highly oxidizing environment causes severe side reactions. Therefore, to avoid such a scenario, the use of solid calcium hypochlorite without chlorate(V) contamination was required in order to perform the reaction homogeneously in solution. To our delight, use of freshly prepared Ca(OCl)_2_ solution resulted in a homogeneous reaction mixture with complete conversions in less than 1 h. In comparison to the previous method, optimal reaction conditions using calcium hypochlorite as chlorine source (method 2) required lower reaction times, less added hypochlorite, and provided higher reaction yields even on a 10 g scale (Table 2, entry 3 and 6). Longer reaction times noticeably reduce conversion of **2a** on account of 2-imidazolone (**3**) (Table 2, entry 2–5). The mechanistic aspect of both methods is summarized in Scheme 3.

**Table 1:**
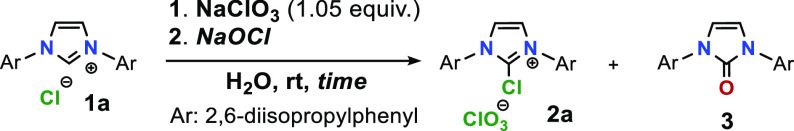
Reaction Conditions Screen for Method 1^a^

entry	NaOCl [equiv]	time [h]	isolated yield [%]^c^	**2a** [%]^b^	**3** [%]^b^
1	1.1	50	n.d.	75	2
2	4.21	5	n.d.	71	1
3	4.34	24	88	89	5
4^d^	4.99	24	86	89	5
5^e^	4.30	24	72	76	16
6^e^^,^^f^	4.30	24	75	79	12

a Reaction conditions: 1 mmol (**1a**), H_2_O: 30 mL/mmol. n.d. = not determined.

b Determined by ^1^H NMR spectroscopy.

c Yield of isolated product upon washing with toluene.

d Other impurities started to form.

e Multigram scale (5 g).

f NaOCl was diluted and added dropwise over 15 min.

**Table 2:**
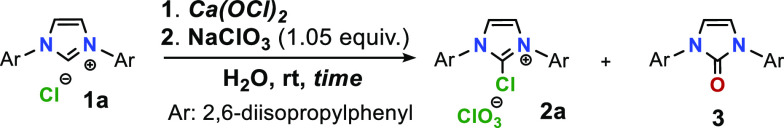
Reaction Conditions Screen for Method 2^a^

entry	Ca(OCl)_2_ [equiv]	time [min]	isolated yield [%]^c^	**2a** [%]^b^	**3** [%]^b^
1	2.0	15	n.d.	86	10
2	0.55	27	n.d.	89	3
3	0.55	37	92	95	5
4	0.55	47	n.d.	86	13
5	0.55	80	n.d.	83	16
6^d^^,^^e^	0.55	42	89	n.d.	n.d.

a Reaction conditions: 1 mmol (**1a**), H_2_O: 18 mL/mmol. n.d. = not determined.

b Determined by ^1^H NMR spectroscopy.

c Yield of isolated product upon washing with toluene.

d Multigram scale (10 g).

e Ca(OCl)_2_ was diluted and added dropwise over 15 min.

**Scheme 3:**
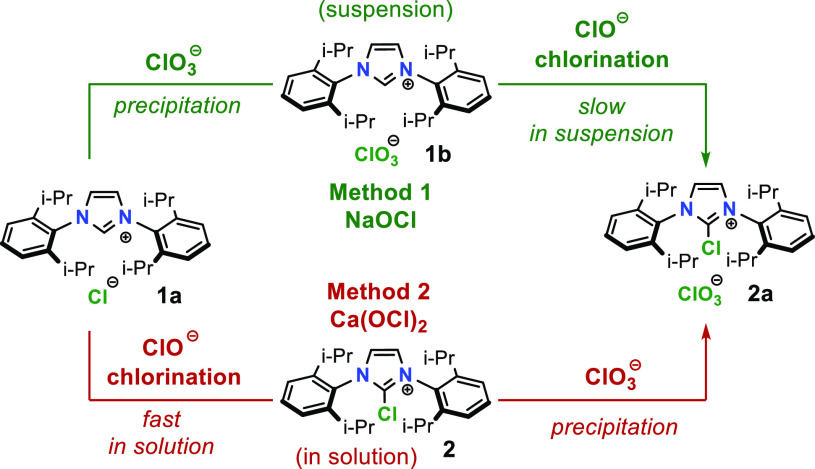
Comparison of Methods

For the purpose of deoxyfluorination, introduction of fluoride into **2a** was studied. There are several reports of poly[hydrogen fluoride] containing fluorinating reagents, with a few containing an imidazolium ring.^14−16^ Our group previously reported the synthesis of an imidazolium-based dihydrogen trifluoride fluorinating reagent prepared by treating **1a** with hydrofluoric acid in aqueous solution.^16^ Anion metathesis occurs due to formation of stable poly[hydrogen fluoride] salt and volatile hydrogen chloride. In case of 2-chloroimidazolium salt (**2a**), the chlorate(V) anion needs to be exchanged. It is a well-known fact that ClO_3_^–^ ion readily decomposes at elevated temperatures in an acidic environment—conditions used for poly[hydrogen fluoride] salt formation—and therefore we expected anion exchange should be feasible. Indeed, when **2a** was subjected to 3–5 cycles of hydrofluoric acid treatment at 85 °C, a 2-chloro-1,3-bis(2,6-diisopropyphenyl)imidazolium dihydrogen trifluoride salt (**2b**) was characterized as a major product with 95% isolated yield obtained on 10 g scale (Scheme 4a). Surprisingly, little to no hydrolysis of 2-chloroimdiazolium moiety was observed during the acid treatments. Synthesis of fluoride-containing 2-chloroimidazolium salt (**2b**) can thus be accomplished in 2 steps from easily accessible 2*H*-imidazolium chloride (**1a**) with 85% overall yield using water as a solvent at ambient conditions. Furthermore, 2-chloro-1,3-bis(2,6-diisopropyphenyl)imidazolium chloride (**2c**) could be prepared in the same manner by using hydrochloric acid (Scheme 4b), which represents an easier alternative route to desirable 2-chloroimdiazolium chloride salts.

**Scheme 4:**
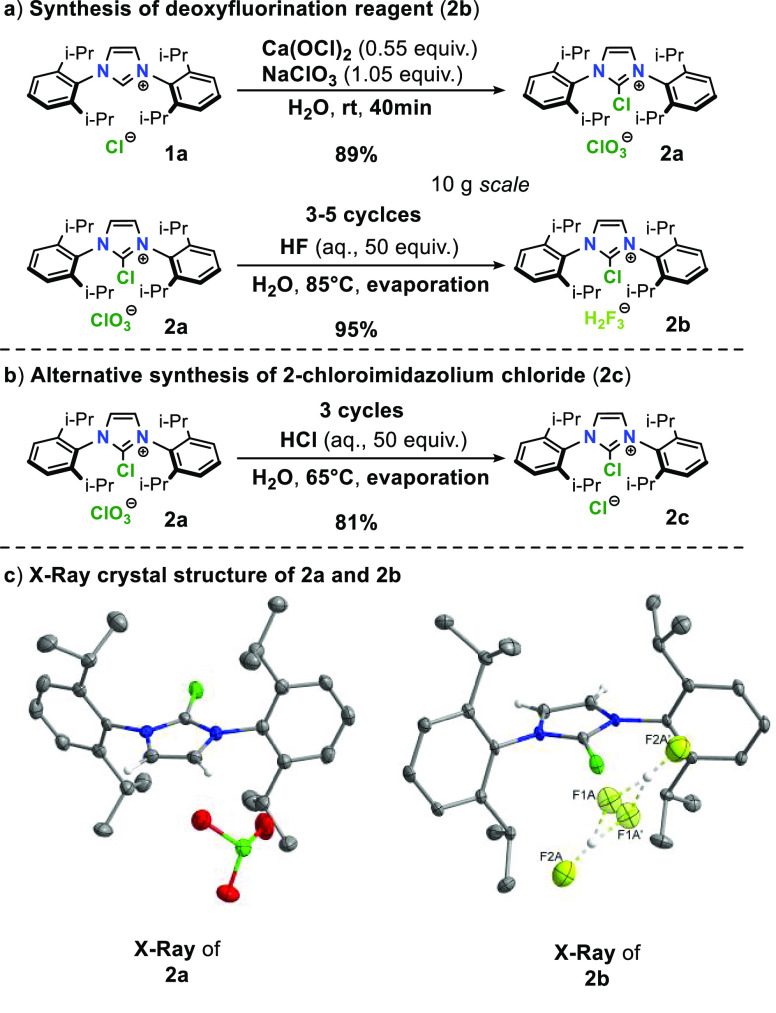
Summary of 2-Chloroimidazolium Salts

Successful synthesis of poly[hydrogen fluoride] salt (**2b**) led to the next step of determining deoxyfluorination capabilities on a variety of phenols. Since deoxyfluorination is water-sensitive, reagent **2b** was dried properly beforehand, under a vacuum at 70 °C for 24 h, which proved to be effective with consistent results. We have chosen 4-hydroxybenzophenone (**4a**) as a suitable testing substrate. To activate dihydrogen trifluoride anion, i.e., to form nucleophilic naked fluoride, hydrogen fluoride needs to be extracted by an organic base or alkali fluoride.^6,16,17^ Screening of several different bases revealed that organic amidine or guanidine bases and alkali fluorides work best (Table 3). Unsurprisingly, deoxyfluorination does not proceed without a base (Table 3, entry 1). Ordinary organic amines do not provide satisfactory conversions (Table 3, entry 6–7), presumptively due to insufficient stability of the formed poly[hydrogen fluoride] salt. Stronger bases with higher p*K*_aH_ values and dispersed positive charge like amidines (DBU) or guanidines (TMG) are able to form stable poly[hydrogen fluorides] and therefore possess greater ability to abstract hydrogen fluoride. Similar findings were obtained when screening for an appropriate base on reagent PyFluor, where hydrogen fluoride is being released.^6^ To obtain satisfactory results, organic amidine or guanidine bases were needed, while organic amines gave negligible conversions. Therefore, DBU was chosen as a cheap and green base. Optimization study revealed that ratio of DBU:**2b** should be at least 2:1 in order to achieve full conversions (Table 3, entry 10–14). An additional amount of base does not affect reaction yields significantly. Only a slight excess of deoxyfluorination reagent **2b** was used (1.1 equiv), surpassing the stoichiometry of reagent PhenoFluorMix consisting of 1.5 equiv of 2-chloroimdiazolium chloride (**2c**) and 10 equiv of CsF.^9^ Additionally, no external fluoride source is required with reagent **2b**, boosting its cost-efficiency.

**Table 3:**
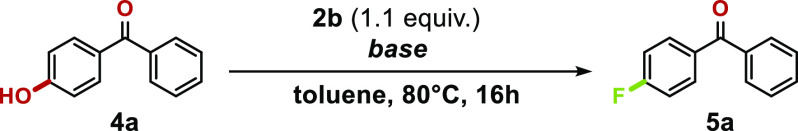
Deoxyfluorination Conditions Screen^a^

entry	base	[equiv]	conversion [%]^b^
1	none	/	0
2	KF	8.0	95
3	CsF	8.0	>99
4	K_2_CO_3_	2.2	1
5	Cs_2_CO_3_	2.2	0
6	DIPEA	2.4	4
7	Et_3_N	2.6	6
8	pyridine	2.9	0
9	TMG	2.2	>99
10	DBU	2.2	>99
11	DBU	1.4	52
12	DBU	3.4	>99
13	DBU	4.7	90
14	DBU	5.8	92

a Reaction conditions: 0.25 mmol (**4a**), 1.1 equiv of (**2b**), toluene, 80 °C, 16 h.

b Determined by ^19^F NMR spectroscopy using 2-nitrobenzotrifluoride as an internal standard.

A mechanistic study conducted by Ritter’s group identified concerted aromatic nucleophilic substitution (CS_N_Ar) as a major pathway.^17^ Only nonpolar solvents afforded sufficient neutral tetrahedral intermediate formation that facilitates concerted rearrangement, which is most noticeable on phenols barring electron-donating groups.^17^ Conducting a solvent screen test on 4-hydroxybenzophenone (**4a**) with reagent **2b** using DBU as a base revealed that most aprotic solvents (polar and nonpolar) work equally well at 80 °C (Supporting Information, Table S4). This apparent discrepancy can be rationalized by the fact that phenols possessing electron-withdrawing substituents allow for a traditional addition–elimination aromatic nucleophilic substitution pathway. The same trend was observed with PhenoFluorMix on electron-deficient substrates. Toluene was chosen as the reaction solvent as it furthermore provided practical separation of the reaction mixture.

As shown in Table 4 a broad scope of electron-deficient phenols was deoxyfluorinated under optimized reaction conditions using reagent **2b**. Functional groups like ketones, aldehydes, nitriles, esters, olefins, sulfonates, nitro groups, diazo compounds, and halogenides are well tolerated with both aryl and heteroaryl compounds suited for deoxyfluorination. Substituents on *para*, *meta*, and even *ortho* (**5n** and **5p**) positions all allow for deoxyfluorination, while electron-withdrawing groups should be present on *para* or *meta* positions, but in most cases not on the *ortho* position, usually due to interference with hydroxyl groups via intramolecular hydrogen bonds (Supporting Information, Table S6). Reaction yields range from fair to quantitative under the mild conditions used. Furthermore, silyl aryl ethers obtained by trimethylsilylation of the corresponding phenols (**5a**, **5b**, **5c**, and **5f**) were also successfully deoxyfluorinated in one step without any loss of reactivity (Supporting Information, Table S8). Unfortunately, electron-rich phenols were too unreactive toward deoxyfluorination with reagent **2b** (Supporting Information, Table S6). Study of different counterions of 2-chloroimidazolium cation showed great anion dependence on deoxyfluorination reaction conversions. Larger anions with distributed negative charge (NO_3_^–^, ClO_3_^–^, PF_6_^–^, BF_4_^–^) form tight ionic pairs in solutions and therefore render the large 2-chloroimidazolium cation rather unreactive toward fluoride exchange with an external fluoride source. On the other hand, smaller ions like chloride readily exchange with external fluoride,^9,17^ thus providing necessary conditions for deoxyfluorination (Supporting Information, Table S7). Poly[hydrogen fluorides] and fluorides formed by deprotonation during the reaction proved to be not nucleophilic enough to facilitate fluorination on electron-rich phenols, even in the presence of a large excess of cesium fluoride to promote hydrogen fluoride exchange.

**Table 4:**
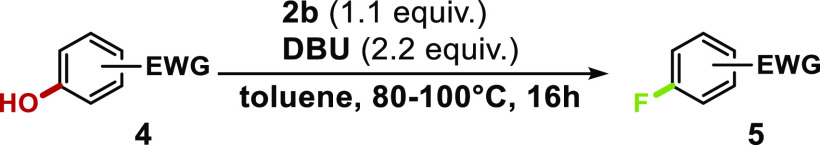

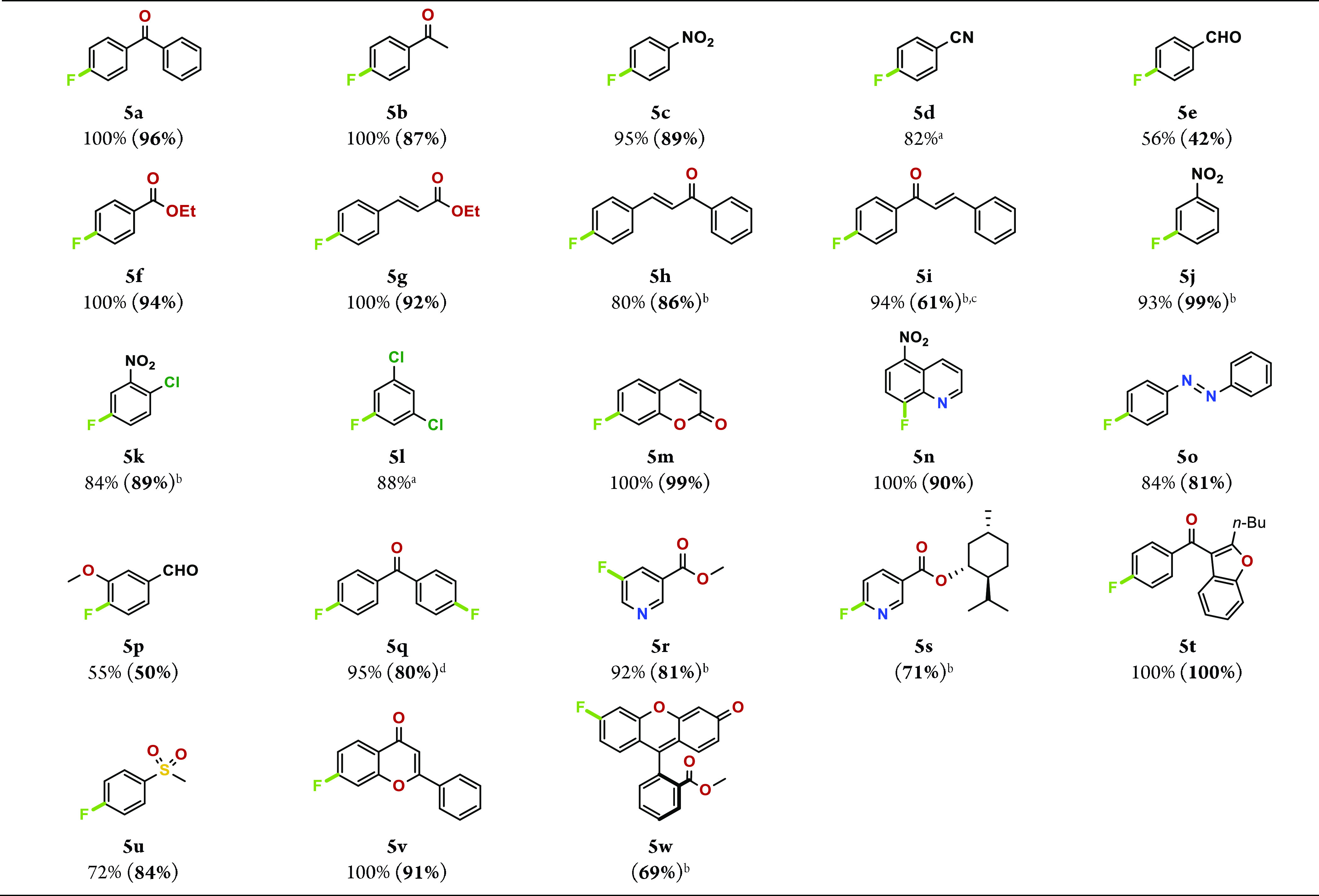
Substrate Scope^e^

a Product was too volatile to isolate.

b 1.3 equiv (**2b**) and 2.6 equiv DBU was used.

c Yields vary due to heterogeneous reaction mixture. Better yields are obtained using DME as a solvent.

d 4,4′-Dihydroxybenzophenone (**4q**) was used as a starting phenol; 2.0 equiv (**2b**) and 4.0 equiv DBU.

e Bolded numbers in parentheses represent isolated yields on 0.5 mmol scale, and percentages in front represent conversions on 0.25 mmol scale determined by ^19^F NMR spectroscopy using 2-nitrobenzotrifluoride as an internal standard.

In conclusion, we have developed a convenient low-cost reaction protocol utilizing aqueous media and bleach for direct one-pot synthesis of 2-chloro-1,3-bis(2,6-diisopropylphenyl)imidazolium chlorate(V) salt. Moreover, chloride and dihydrogen trifluoride salts were both easily accessed by anion metathesis in acidic medium at elevated temperatures with hydrochloric or hydrofluoric acid treatments, respectively. Synthesized 2-chloro-1,3-bis(2,6-diisopropylphenyl)imidazolium dihydrogen trifluoride salt proved to be a useful deoxyfluorination reagent insensitive toward moisture and storable in air for longer periods of time. It is capable of converting electron-deficient phenols to the corresponding aryl fluorides with up to quantitative yields under mild reaction conditions using optimized reaction conditions: DBU as a base and toluene as a solvent. We demonstrated a wide functional group tolerance on a broad substrate scope. Furthermore, silyl aryl ethers obtained by trimethylsilylation of phenols were equally well deoxyfluorinated in one step without loss of reactivity.

## Data Availability

The data underlying this study are available in the published article and its Supporting Information.

## Supporting Information Available

Experimental procedures, materials, full analysis data and copies of spectra, X-ray crystallographic data (PDF)
