# Supplementary material for: Deoxyfluorination of Electron-Deficient Phenols
Source: Org Lett. 2023 May 16;25(20):3649–53. doi: 10.1021/acs.orglett.3c01018 (PMC10226106; doi:10.1021/acs.orglett.3c01018)
Supplement: Supplementary file 1 — ol3c01018_si_001.pdf [file ol3c01018_si_001.pdf]

Supporting Information

Deoxyfluorination of Electron-Deficient Phenols

Jan Jelen[a,b] and Gašper Tavčar[a,b]*

[a] Department of Inorganic Chemistry and Technology, Jožef Stefan Institute, Jamova 39, 1000 Ljubljana, Slovenia

[b] Jožef Stefan International Postgraduate School, Jamova 39, 1000 Ljubljana, Slovenia

*E-mail: gasper.tavcar@ijs.si

Table of Contents

S1 Materials and Methods **S6**
S2 Deoxyfluorination reagent synthesis **S7**
1,4-Bis(2,6-diisopropylphenyl)-1,4-diazabutadiene (**S1**) S7
1,3-Bis(2,6-diisopropylphenyl)imidazolium chloride (**1a**) S7
1,3-Bis(2,6-diisopropylphenyl)-2-chloroimidazolium chlorate(V) (**2a**) S7
1,3-Bis(2,6-diisopropylphenyl)-2-chloroimidazolium dihydrogen trifluoride (**2b**) S9
1,3-Bis(2,6-diisopropylphenyl)-2-chloroimidazolium chloride (**2c**) S9
S3 Study of chlorination with hypochlorite **S11**
Preliminary product scan test S11
1,3-Bis(2,6-diisopropylphenyl)-2-imidazolone (**3**) S11
1,3-Bis(2,6-diisopropylphenyl)-2H-imidazolium chlorate(V) (**1b**) S12
Chlorination kinetics S12
Optimization of method 1 (sodium hypochlorite) S13
Optimization of method 2 (calcium hypochlorite) S14
S4 Synthesis of various phenol substrates **S15**
Ethyl 4-hydroxybenzoate (**4f**) S15
Ethyl p-coumarate (**4g**) S15
Menthyl 6-hydroxynicotinate (**4s**) S15
S5 Synthesis of trimethylsilyl (TMS) protected phenols **S17**
(4-benzoylphenoxy)trimethylsilane (**6a**): S17
(4-acetylphenoxy)trimethylsilane (**6b**): S17
(4-nitrophenoxy)trimethylsilane (**6c**): S17
Ethyl 4-trimethylsilyloxybenzoate (**6f**): S18
S6 Deoxyfluorination of phenol substrates **S19**
General procedure for deoxyfluorination S19
4-Fluorobenzophenone (**5a**) S19
4-Fluoroacetophenone (**5b**) S19
4-Fluoronitrobenzene (**5c**) S20
4-Fluorobenzonitrile (**5d**) S20
4-Fluorobenzaldehyde (**5e**) S21
Ethyl 4-fluorobenzoate (**5f**) S21
Ethyl 4-fluorocinnamate (**5g**) S22
4-Fluorochalcone (**5h**) S22
4’-Fluorochalcone (**5i**) S23
3-Fluoronitrobenzene (**5j**) S23
4-Fluoro-1-chloro-2-nitrobenzene (**5k**) S24
1-Fluoro-3,5-dichlorobenzene (**5l**) S24
7-Fluorocoumarin (**5m**) S24

8-Fluoro-5-nitroquinoline (**5n**) S25
4-Fluoroazobenzene (**5o**) S25
4-Fluoro-3-methoxybenzaldehyde (**5p**) S26
4,4'-Difluorobenzophenone (**5q**) S26
Methyl 5-fluoronicotinate (**5r**) S27
Menthyl 6-fluoronicotinate (**5s**) S27
2-Butyl-3-(4-fluorobenzoyl)benzofuran (**5t**) S28
4-Fluoro(methylsulfonyl)benzene (**5u**) S29
7-Fluoroflavone (**5v**) S29
Methyl 2-(6-fluoro-3-oxoxanthen-9-yl)benzoate (**5w**) S30
S7 Deoxyfluorination on large scale synthesis S31
S8 Evaluation of different bases for deoxyfluorination S32
S9 Evaluation of different solvents for deoxyfluorination S33
S10 Determination of deoxyfluorination reaction conversions S34
S11 Deoxyfluorination of other phenol substrates S35
S12 Deoxyfluorination anion dependance study S36
S13 Deoxyfluorination of silyl protected phenols S37
S14 Crystal Structure data S38
1,3-Bis(2,6-diisopropylphenyl)-2-chloroimidazolium chlorate(V) (**2a**) S38
1,3-Bis(2,6-diisopropylphenyl)-2-chloroimidazolium dihydrogen trifluoride (**2b**) S43
1,3-Bis(2,6-diisopropylphenyl)-2-imidazolone (**3**) S47
Methyl 2-(6-fluoro-3-oxoxanthen-9-yl)benzoate (**5w**) S48
S15 Raman Spectroscopic data S52
Raman spectra of 1,3-bis(2,6-diisopropylphenyl)imidazolium chlorate(V) (**1b**) S52
Raman spectra comparison between (**1b**) and sodium chlorate(V) S52
Raman spectra of 1,3-bis(2,6-diisopropylphenyl)-2-chloroimidazolium chlorate(V) (**2a**) S53
Raman spectra comparison between (**2a**) and sodium chlorate(V) S53
Raman spectra of 1,3-bis(2,6-diisopropylphenyl)-2-chloroimidazolium dihydrogen trifluoride (**2b**) S54
Raman spectra of 1,3-bis(2,6-diisopropylphenyl)-2-chloroimidazolium chloride (**2c**) S54
S16 NMR Spectroscopic data S55
^{1}H NMR of 1,3-Bis(2,6-diisopropylphenyl)-2H-imidazolium chlorate(V) (**1b**) S55
^{13}C NMR of 1,3-Bis(2,6-diisopropylphenyl)-2H-imidazolium chlorate(V) (**1b**) S55
^{1}H NMR of 1,3-Bis(2,6-diisopropylphenyl)-2-chloroimidazolium chlorate(V) (**2a**) S56
^{13}C NMR of 1,3-Bis(2,6-diisopropylphenyl)-2-chloroimidazolium chlorate(V) (**2a**) S56
^{1}H NMR of 1,3-Bis(2,6-diisopropylphenyl)-2-chloroimidazolium dihydrogen trifluoride (**2b**) S57
^{13}C NMR of 1,3-Bis(2,6-diisopropylphenyl)-2-chloroimidazolium dihydrogen trifluoride (**2b**) S57
^{19}F NMR of 1,3-Bis(2,6-diisopropylphenyl)-2-chloroimidazolium dihydrogen trifluoride (**2b**) S58
^{1}H NMR of 1,3-Bis(2,6-diisopropylphenyl)-2-chloroimidazolium chloride (**2c**) S58
^{13}C NMR of 1,3-Bis(2,6-diisopropylphenyl)-2-chloroimidazolium chloride (**2c**) S59
^{1}H NMR of 1,3-Bis(2,6-diisopropylphenyl)-2-imidazolone (**3**) S59

^{13}C NMR of 1,3-Bis(2,6-diisopropylphenyl)-2-imidazolone (**3**) S60
^{1}H NMR of Menthyl 6-hydroxynicotinate (**4s**) S60
^{13}C NMR of Menthyl 6-hydroxynicotinate (**4s**) S61
^{1}H NMR of 4-Fluorobenzophenone (**5a**) S61
^{19}F NMR of 4-Fluorobenzophenone (**5a**) S62
^{1}H NMR of 4-Fluoroacetophenone (**5b**) S62
^{13}C NMR of 4-Fluoroacetophenone (**5b**) S63
^{19}F NMR of 4-Fluoroacetophenone (**5b**) S63
^{1}H NMR of 4-Fluoronitrobenzene (**5c**) S64
^{13}C NMR of 4-Fluoronitrobenzene (**5c**) S64
^{19}F NMR of 4-Fluoronitrobenzene (**5c**) S65
^{1}H NMR of 4-Fluorobenzaldehyde (**5e**) S65
^{13}C NMR of 4-Fluorobenzaldehyde (**5e**) S66
^{1}H NMR of Ethyl 4-fluorobenzoate (**5f**) S66
^{13}C NMR Spectra of Ethyl 4-fluorobenzoate (**5f**) S67
^{19}F NMR Spectra of Ethyl 4-fluorobenzoate (**5f**) S67
^{1}H NMR of Ethyl 4-fluorocinnamate (**5g**) S68
^{13}C NMR of Ethyl 4-fluorocinnamate (**5g**) S68
^{19}F NMR of Ethyl 4-fluorocinnamate (**5g**) S69
^{1}H NMR of 4-Fluorochalcone (**5h**) S69
^{13}C NMR of 4-Fluorochalcone (**5h**) S70
^{19}F NMR of 4-Fluorochalcone (**5h**) S70
^{1}H NMR of 4’-Fluorochalcone (**5i**) S71
^{13}C NMR of 4’-Fluorochalcone (**5i**) S71
^{19}F NMR of 4’-Fluorochalcone (**5i**) S72
^{1}H NMR of 4-Fluoro-1-chloro-2-nitrobenzene (**5k**) S72
^{13}C NMR of 4-Fluoro-1-chloro-2-nitrobenzene (**5k**) S73
^{19}F NMR of 4-Fluoro-1-chloro-2-nitrobenzene (**5k**) S73
^{1}H NMR of 7-Fluorocoumarin (**5m**) S74
^{19}F NMR of 7-Fluorocoumarin (**5m**) S74
^{1}H NMR of 8-Fluoro-5-nitroquinoline (**5n**) S75
^{13}C NMR of 8-Fluoro-5-nitroquinoline (**5n**) S75
^{19}F NMR of 8-Fluoro-5-nitroquinoline (**5n**) S76
^{1}H NMR of 4-Fluoroazobenzene (**5o**) S76
^{19}F NMR of 4-Fluoroazobenzene (**5o**) S77
^{1}H NMR of 4-Fluoro-3-methoxybenzaldehyde (**5p**) S77
^{13}C NMR of 4-Fluoro-3-methoxybenzaldehyde (**5p**) S78
^{19}F NMR of 4-Fluoro-3-methoxybenzaldehyde (**5p**) S78
^{1}H NMR of 4,4’-Difluorobenzophenone (**5q**) S79
^{13}C NMR of 4,4’-Difluorobenzophenone (**5q**) S79

^{19}F NMR of 4,4’-Difluorobenzophenone (**5q**) ... S80
^{1}H NMR of Methyl 5-fluoronicotinate (**5r**) ... S80
^{13}C NMR of Methyl 5-fluoronicotinate (**5r**) ... S81
^{19}F NMR of Methyl 5-fluoronicotinate (**5r**) ... S81
^{1}H NMR of Menthyl 6-fluoronicotinate (**5s**) ... S82
^{13}C NMR of Menthyl 6-fluoronicotinate (**5s**) ... S82
^{19}F NMR of Menthyl 6-fluoronicotinate (**5s**) ... S83
^{1}H NMR of 2-Butyl-3-(4-fluorobenzoyl)benzofuran (**5t**) ... S83
^{13}C NMR of 2-Butyl-3-(4-fluorobenzoyl)benzofuran (**5t**) ... S84
^{19}F NMR of 2-Butyl-3-(4-fluorobenzoyl)benzofuran (**5t**) ... S84
^{1}H NMR of 4-Fluoro(methylsulfonyl)benzene (**5u**) ... S85
^{13}C NMR of 4-Fluoro(methylsulfonyl)benzene (**5u**) ... S85
^{19}F NMR Spectra of 4-Fluoro(methylsulfonyl)benzene (**5u**) ... S86
^{1}H NMR of 7-Fluoroflavone (**5v**) ... S86
^{13}C NMR of 7-Fluoroflavone (**5v**) ... S87
^{19}F NMR of 7-Fluoroflavone (**5v**) ... S87
^{1}H NMR of Methyl 2-(6-fluoro-3-oxoxanthen-9-yl)benzoate (**5w**) ... S88
^{13}C NMR of Methyl 2-(6-fluoro-3-oxoxanthen-9-yl)benzoate (**5w**) ... S88
^{19}F NMR of Methyl 2-(6-fluoro-3-oxoxanthen-9-yl)benzoate (**5w**) ... S89
S17 References ... S90

S1 Materials and Methods

General Information

All reactions where not stated otherwise were carried out under an ambient atmosphere. All chemicals and solvents were used as received without further purification unless otherwise specified. Synthesis that required inert conditions were carried out using standard Schlenk techniques under dry argon atmosphere. Acetonitrile, toluene, 1,4-dioxane, THF and DME were dried by distillation from deep purple sodium benzophenone ketyl and stored over molecular sieves (3Å or 4Å) for at least 48 h before use. Other solvents were dried with molecular sieves for at least 72h before use. Glassware was oven-dried overnight at 150°C before use. Reactions involving aqueous hydrofluoric acid were carried out using polypropylene plastic equipment. All deoxyfluorination reaction involving reagent (**2b**) were carried out in capped 7 mL glass vials. Reagent (**2b**) was dried overnight at 70°C under vacuum in Teflon tube inserted into 500 mL Schlenk flask.

TLC and Column Chromatography

TLC analysis was performed on analytical TLC chromatographic plates (Merc Silica gel 60 F_{254}). Spots were visualized under UV light (254 nm) or by iodine vapours. Column chromatography was performed using LiChroprep Silica gel 60 (15-25 μm particle size) and forced flow of eluent.

NMR Spectroscopy

NMR samples were prepared under ambient atmosphere. All deuterated solvents were stored over 3 Å molecular sieves. NMR spectra were recorded at the Slovenian NMR Centre (National Institute of Chemistry) using a Bruker AVANCE NEO 600 or 400 MHz NMR Spectrometer. Spectra were recorded at 298 K. Chemical shifts of ^{1}H and ^{13}C were referenced to residual signals of deuterated solvents and are given relative to tetramethylsilane (TMS). The ^{19}F references were calculated according to IUPAC guidelines and are given relative to $CFCl_3$. Spectra were analysed using MestReNova 12 (Mestrelab Research S.L.) or Topspin 4.1.4. (Bruker BioSpin GmbH) program packages. Quantitative ^{19}F NMR spectroscopy was performed by peak integration of analyte and 2-nitrobenzotrifluoride as internal standard (-60 ppm in C_6D_6).

Raman Spectroscopy

Samples were loaded onto microscope slide glass under ambient atmosphere. Raman spectra were recorded using a Horiba Jobin Yvon Labram-HR spectrometer coupled with an Olympus BXFM-ILHS microscope at room temperature. Samples were excited with the 633 nm (red) emission line of He-Ne laser. Data was processed with Microsoft Excel.

Crystal Structure Determination

Crystal data were collected on a Gemini A diffractometer equipped with an Atlas CCD detector using graphite-monochromated Cu Kα radiation. All crystal data were collected at 150 K unless otherwise stated. Data were processed using the CrysAlisPro software package. An analytical absorption correction was applied to all data sets. Structures were solved using the SHELXT program. Structure refinement was performed using the SHELXL software implemented in the Olex2 program package. Figures were prepared using Diamond 4.0. Ellipsoids are drawn at 50% probability.

S2 Deoxyfluorination reagent synthesis

1,4-Bis(2,6-diisopropylphenyl)-1,4-diazabutadiene (S1)

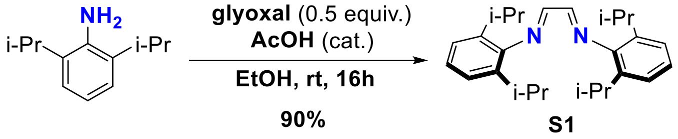

Reaction was carried out in 1 L round-bottom flask. A solution of glyoxal (72.59 g, 40% aq., 0.50 equiv.) in EtOH (200 mL) was added to a solution of 2,6-diisopropylaniline (197 g, 90%, 1 mmol, 1 equiv.) and AcOH (1 mL) in EtOH (200 mL) preheated to 50°C. After 15 minutes a yellow precipitate formed. Reaction mixture was left stirring overnight and then filtered off. The filter cake was washed with ice-cooled EtOH (100 mL) and then left to dry in an oven (60°C) to afford 169 g of (**S1**) as a yellow crystalline solid (**90%** yield).

NMR Spectroscopy:

> **^{1}H NMR** (400 MHz, MeCN-d$_3$): δ 8.08 (s, 2H), 7.24 – 7.19 (m, 4H), 7.14 (dd, J = 8.7, 6.6 Hz, 2H), 2.93 (hept, J = 6.9 Hz, 4H), 1.18 (d, J = 6.9 Hz, 24H).

Spectroscopic data corresponds with that previously reported.[1]

1,3-Bis(2,6-diisopropylphenyl)imidazolium chloride (1a)

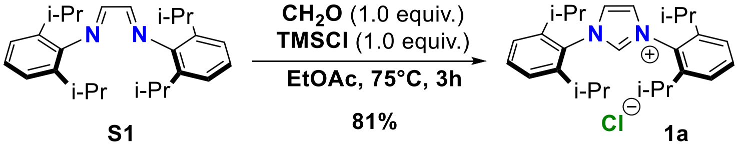

A 2 L round-bottom flask containing EtOAc (1.0 L) was preheated to 70°C. 1,4-bis(2,6-diisopropylphenyl)-1,4-diazabutadiene (**S1**) (58.21 g, 155 mmol, 1.0 equiv.) and paraformaldehyde (4.671 g, 155 mmol, 1.0 equiv.) were added and rinsed with EtOAc (50 mL). Solution of TMSCl (20.0 mL, 158 mmol, 1.02 equiv.) in EtOAc (40 mL) was added dropwise to flask via dropping funnel over 45 min with vigorous stirring. Resulting suspension was stirred for another 2 h at 70°C and then cooled in an ice bath. The reaction mixture was filtered off and the collected solid washed with EtOAc (2x 100 mL), then left to dry in an oven (70°C) to afford 53.45 g of (**1a**) as an off-white solid (**81%** yield).

NMR Spectroscopy:

> **^{1}H NMR** (400 MHz, MeCN-d$_3$): δ 10.29 (t, J = 1.6 Hz, 1H), 7.91 (d, J = 1.6 Hz, 2H), 7.67 (t, J = 7.9 Hz, 2H), 7.49 (d, J = 7.9 Hz, 4H), 2.45 (hept, J = 6.8 Hz, 4H), 1.29 (d, J = 6.8 Hz, 12H), 1.24 (d, J = 6.9 Hz, 12H).

Spectroscopic data corresponds with that previously reported.[1]

1,3-Bis(2,6-diisopropylphenyl)-2-chloroimidazolium chlorate(V) (2a)

Method 1 (scales up to 5 mmol)

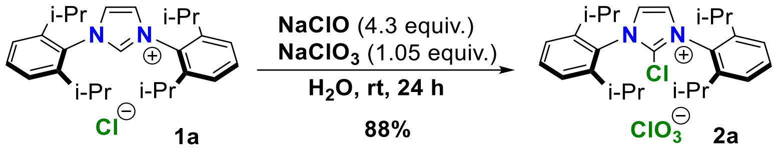

Reaction was carried out in 80 mL beaker. To solution of 1,3-bis(2,6-diisopropylphenyl)imidazolium chloride (**1a**) (420 mg, 0.99 mmol, 1.0 equiv.) in deionized water (15 mL) was added a solution of $NaClO_3$ (110 mg, 1.03 mmol, 1.05 equiv.) in deionized water (5 mL) at room temperature forming a white precipitate. To a suspension was added a diluted solution of sodium hypochlorite (0.85 M, 5 mL, 4.25 mmol, 4.3 equiv.) in deionized water (5 mL) dropwise over 15 min with strong stirring. Reaction mixture was left stirring overnight, then filtered off and the remaining solid washed with deionized water (2x 5 mL) and toluene (2x 5 mL) to afford 441 mg of (**2a**) as a white solid (**88%** yield).

Method 2 (all scales)

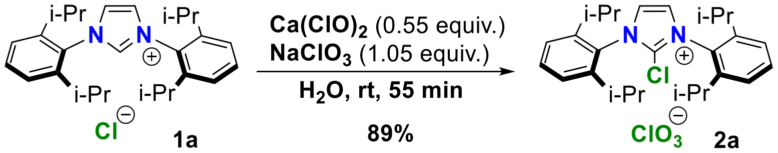

Preparation of reagent solution: $Ca(ClO)_2$ (25 g, 67-75%) was mixed in deionized water (2x 50 mL) for 15 min and then the suspension filtered off. The liquid filtrate was collected and active chlorine concentration determined by iodometric titration resulting in 100 mL of 0.605 M calcium hypochlorite reagent solution (1.21 mmol/mL of hypochlorite) used in next step.

Reaction was carried out in 1 L beaker. To a solution of 1,3-bis(2,6-diisopropylphenyl)imidazolium chloride (**1a**) (10.30 g, 24.2 mmol, 1.0 equiv.) in deionized water (350 mL) was added previously prepared $Ca(ClO)_2$ reagent solution (22 mL, 13.3 mmol, 0.55 equiv.) diluted with deionized water (50 mL) and NaOH (0.25 M, 40 mL) dropwise over 15 min via dropping funnel. The resulting reaction mixture was stirred for 40 min at room temperature and then quenched with addition of $NaClO_3$ (2.70 g, 25.4 mmol, 1.05 equiv.) in deionized water (20 mL) to form a white precipitate. Suspension was vacuum filtered off and the remaining solid washed with deionized water (2x 40 mL) and toluene (2x 40 mL). For further purification, obtained product was mixed in DME (50 mL) or in EtOAc/iPrOH (3:1 (v/v), 30 mL) for 10 minutes, filtered off and dried overnight in an oven (70°C) to afford 10.89 g of (**2a**) as a white solid (**89%** yield).

NMR Spectroscopy:

^{1}H NMR (600 MHz, MeCN-d_3): δ 8.12 (s, 2H), 7.75 (t, J = 7.8 Hz, 2H), 7.56 (d, J = 7.8 Hz, 4H), 2.37 (hept, J = 6.8 Hz, 4H), 1.30 (d, J = 6.8 Hz, 12H), 1.25 (d, J = 6.8 Hz, 12H).

^{13}C NMR (151 MHz, MeCN-d_3): δ 146.4, 135.1, 133.9, 129.4, 127.5, 126.3, 30.2, 24.2, 23.5.

Raman Spectroscopy: (major peaks, cm^{-1}) 121.8, 142.7, 187.4, 258.6, 297.5, 444.7, 473.2, 508.3, 615.6, 888.2, 932.8 (ClO_3^-), 987.9, 1047.7, 1108.5, 1238.6, 1304.1, 1349.7, 1412.3, 1442.7, 1469.3, 1596.6, 2871.9, 2911.8, 2963.0, 3070.3. Full spectra available on page S53.

Elemental Analysis: Anal. Calc'd for $C_{27}H_{36}N_2O_3Cl_2$: C 63.90%, H 7.16%, N 5.54%, Cl 13.97%. Found: C 63.96%, H 7.22%, N 5.51%.

Crystal Structure: Full data on page S38.

Compound (**2a**) is a new compound not reported before in literature.

1,3-Bis(2,6-diisopropylphenyl)-2-chloroimidazolium dihydrogen trifluoride (2b)

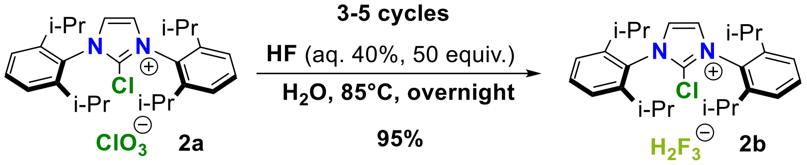

Reaction was carried out in 250 mL polypropylene plastic beaker. To 1,3-bis(2,6-diisopropylphenyl)-2-chloroimidazolium chlorate (V) (**2a**) (10.73 g, 21.1 mmol, 1.0 equiv.) suspended in deionized water (75 mL) was added aqueous hydrofluoric acid (40%, 46 mL, 1.04 mol, 50 equiv.) diluted with deionized water (25 mL) under strong stirring. Suspension was heated to 85°C in an oil bath and left stirring overnight until all the liquid evaporated. The process was repeated two to four more times (3-5 in total) and the remaining solid washed with deionized water (50 mL) and toluene (2x 40 mL). The product was dried completely overnight under vacuum at 70°C in Teflon tube to afford 9.31 g of (**2b**) as a white solid (**95%** yield).

NMR Spectroscopy:

^{1}**H NMR** (600 MHz, MeCN-d$_3$): δ 12.72 (b.s., 2H), 8.08 (s, 2H), 7.75 (t, J = 7.9 Hz, 2H), 7.56 (d, J = 7.9 Hz, 4H), 2.37 (hept, J = 6.8 Hz, 4H), 1.30 (d, J = 6.8 Hz, 12H), 1.25 (d, J = 6.9 Hz, 12H).

^{13}**C NMR** (151 MHz, MeCN-d$_3$): δ 146.4, 135.8, 133.9, 129.5, 127.5, 126.4, 30.2, 24.2, 23.6.

^{19}**F NMR** (565 MHz, MeCN-d$_3$): δ -169.0 (b.s.).

Raman Spectroscopy: (major peaks, cm^{-1}) 118.0, 138.0, 255.7, 296.6, 445.7, 500.7, 621.3, 888.2, 961.3, 989.8, 1044.9, 1106.6, 1238.6, 1305.0, 1349.7, 1419.0, 1449.4, 1468.4, 1589.0, 2871.9, 2910.8, 2936.5, 2973.5, 3074.1. Full spectra available on page S54.

Crystal Structure: Full data available on page S43.

Compound (**2b**) is a new compound not reported before in literature.

1,3-Bis(2,6-diisopropylphenyl)-2-chloroimidazolium chloride (2c)

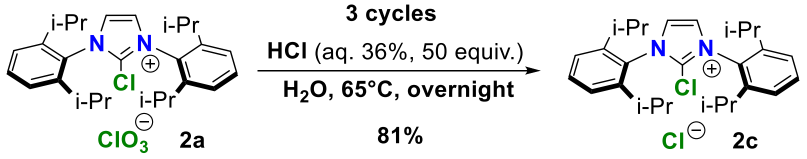

Reaction was carried out in 80 mL beaker. To 1,3-bis(2,6-diisopropylphenyl)-2-chloroimidazolium chlorate (V) (**2a**) (2.54 g, 5.0 mmol, 1.0 equiv.) suspended in deionized water (20 mL) was added aqueous hydrochloric acid (36%, 21 mL, 250 mmol, 50 equiv.) diluted with deionized water (20 mL) under strong stirring. Suspension was heated to 65°C in an oil bath and left stirring overnight until all liquid evaporated and left solid was dry. During the reaction suspended solid readily dissolves and gases are released. The process was repeated two more times (3 in total) and the remaining solid washed with toluene (25 mL), EtOAc (20 mL) and THF (5-10 mL). The product was dried completely for 4 h under strong vacuum at 130°C to afford 1.86 g of (**2c**) as a white solid (**81%** yield).

NMR Spectroscopy:

^{1}H NMR (400 MHz, MeCN-d_3): δ 8.38 (s, 2H), 7.72 (t, *J* = 7.8 Hz, 2H), 7.53 (d, *J* = 7.9 Hz, 4H), 2.34 (hept, *J* = 6.9 Hz, 4H), 1.27 (d, *J* = 6.9 Hz, 12H), 1.21 (d, *J* = 7.0 Hz, 12H).

^{13}C NMR (151 MHz, MeCN-d_3): δ 146.3, 135.7, 133.8, 129.4, 127.7, 126.3, 30.2, 24.3, 23.5.

Raman Spectroscopy: (major peaks, cm^{-1}) 124.8, 136.7, 206.2, 248.9, 302.5, 316.4, 428.6, 449.4, 503.0, 569.5, 618.2, 885.2, 957.7, 985.5, 1042.1, 1104.6, 1234.7, 1305.1, 1353.8, 1421.3, 1445.1, 1466.9, 1593.0, 2869.6, 2914.3, 2969.9, 3070.2. Full spectra available on page S54.

Spectroscopic data corresponds with that previously reported.[1]

*Reference compound (**2c**) for comparison of spectroscopic data was prepared by standard literature procedure from NHC carbene* and C_2Cl_6.[1]

S3 Study of chlorination with hypochlorite

Preliminary product scan test

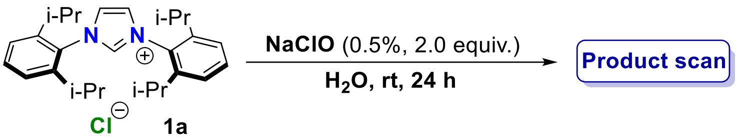

Reaction was carried out in 80 mL beaker. To solution of 1,3-bis(2,6-diisopropylphenyl)imidazolium chloride (**1a**) (425 mg, 1.0 mmol, 1.0 equiv.) in deionized water (20 mL) was added a diluted solution of sodium hypochlorite (0.5%, 29.8 g, 2.0 mmol, 2.0 equiv.) resulting in formation of white precipitate. Reaction mixture was left stirring overnight at room temperature and then vacuum filtered off. The remaining solid was washed with deionized water (2× 5 mL), dried at 70°C and analyzed by NMR spectroscopy (MeCN-d_3 solvent).

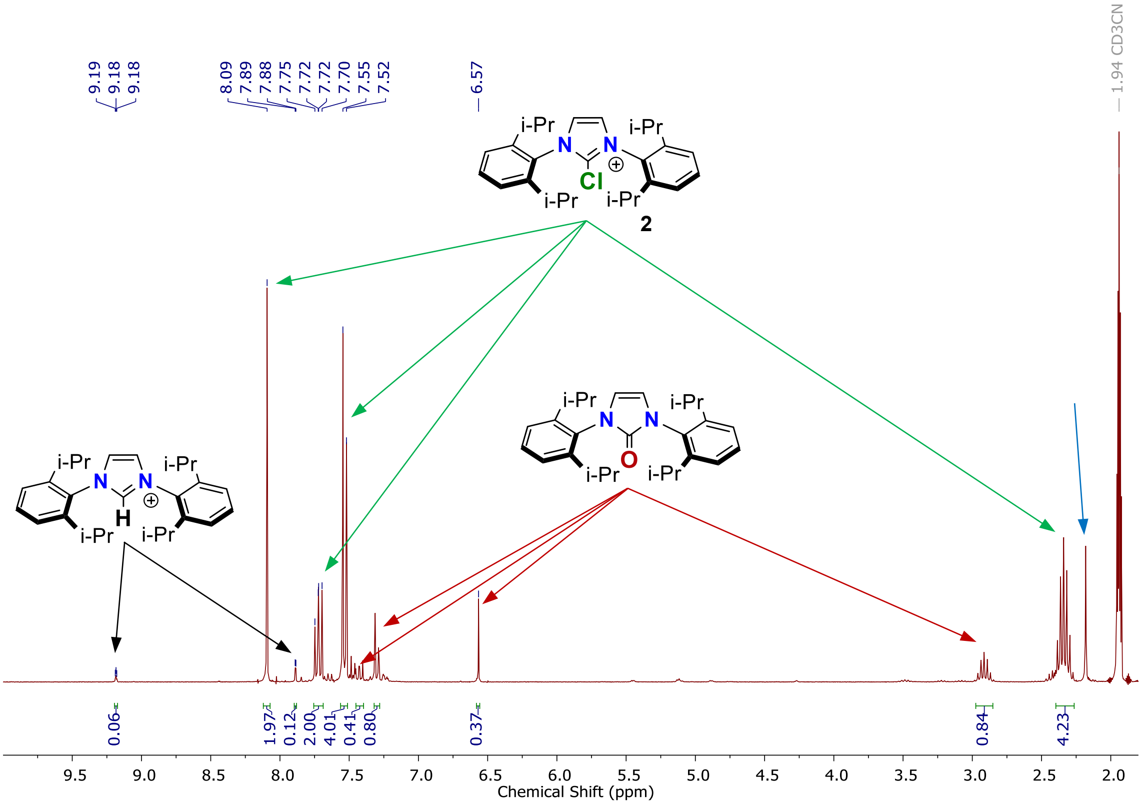

Figure S1. ^{1}H NMR (400 MHz, MeCN-d^3) spectrum of reaction mixture.

Determined ratio (**1**):(**2**):(**3**) = **5:80:15**

1,3-Bis(2,6-diisopropylphenyl)-2-imidazolone (3)

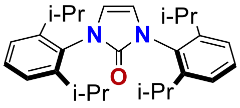

3

2-Imidazolone (**3**) was characterized by comparison of ^{1}H NMR data of pure compound (page S59), IR spectroscopy and determination of single crystal structure (page S47).

NMR Spectroscopy:

^{1}**H NMR** (400 MHz, MeCN-d$_3$): δ 7.43 (dd, *J* = 8.3, 7.2 Hz, 2H), 7.31 (d, *J* = 7.7 Hz, 4H), 6.57 (s, 2H), 2.92 (hept, *J* = 6.9 Hz, 4H), 1.24 (d, *J* = 6.9 Hz, 12H), 1.18 (d, *J* = 6.9 Hz, 12H).

^{13}**C NMR** (101 MHz, MeCN-d$_3$): δ 153.6, 148.6, 133.5, 130.5, 124.8, 114.3, 29.6, 24.0, 23.8.

IR Spectroscopy: 1676.9 cm^{-1} (C=O vibration).

Crystal Structure: Full data available on page S47.

1,3-Bis(2,6-diisopropylphenyl)-2H-imidazolium chlorate(V) (1b)

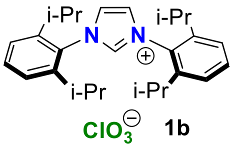

2H-imdiazolium chlorate(V) (**1b**) precipitate was characterized by ^{1}H NMR and Raman spectroscopy. Precipitate is formed by addition of $NaClO_3$ dissolved in deionized water to solution of 1,3-bis(2,6-diisopropylphenyl)imidazolium chloride (**1a**) in deionized water.

NMR Spectroscopy:

^{1}**H NMR** (400 MHz, MeCN-d$_3$): δ 9.20 (t, *J* = 1.5 Hz, 1H), 7.90 (d, *J* = 1.4 Hz, 2H), 7.65 (t, *J* = 7.8 Hz, 2H), 7.47 (d, *J* = 7.9 Hz, 4H), 2.43 (hept, *J* = 6.9 Hz, 4H), 1.27 (d, *J* = 6.8 Hz, 12H), 1.20 (d, *J* = 6.9 Hz, 12H).

^{13}**C NMR** (101 MHz, MeCN-d$_3$): δ 146.3, 139.6, 133.0, 130.9, 127.0, 125.6, 29.9, 24.5, 23.8.

Raman Spectroscopy: (major peaks, cm^{-1}) 115.9, 160.6, 369.8, 316.4, 447.4, 473.3, 528.8, 615.2, 731.4, 886.2, 932.9, 957.7, 1041.1, 1105.6, 1239.6, 1304.1, 1332.9, 1391.5, 1445.1, 1466.0, 1543.4, 1592.0, 2868.6, 2915.3, 2939.1, 2970.9, 3075.1. Full spectra available on page S52.

Chlorination kinetics

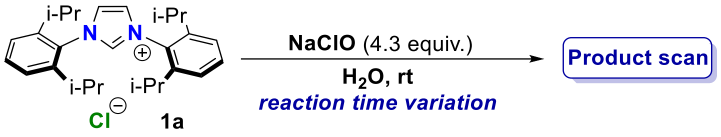

Reactions were carried out in 80 mL beaker. To a solution of 1,3-bis(2,6-diisopropylphenyl)imidazolium chloride (**1a**) (420 mg, 0.99 mmol, 1.0 equiv.) in deionized water (15 mL) was added a solution of $NaClO_3$ (110 mg, 1.03 mmol, 1.05 equiv.) in deionized water (5 mL) at room temperature forming a white precipitate. To a suspension was added a diluted solution of sodium hypochlorite (0.85 M, 5 mL, 4.25 mmol, 4.3 equiv.) in deionized water (5 mL) at once. Reaction mixture was left stirring for various amount of time and then vacuum filtered off. The remaining solid was washed with deionized water (2× 5 mL), dried at 70°C and analyzed by NMR spectroscopy (MeCN-d$_3$ solvent).

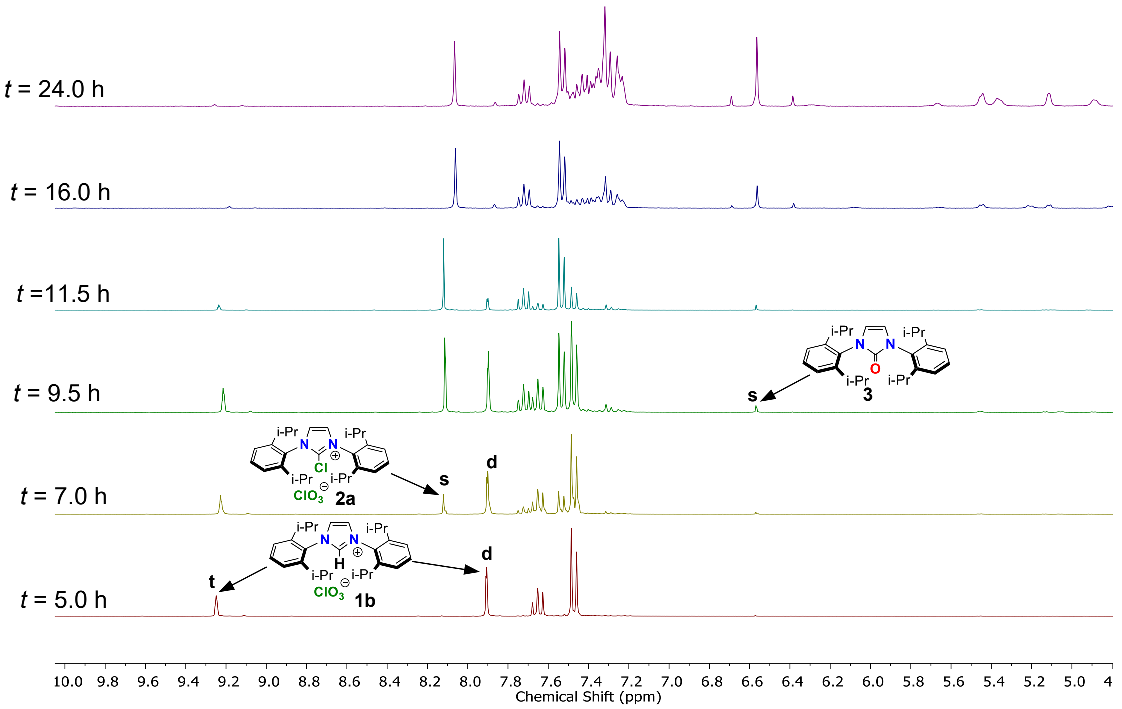

Figure S2. ^{1}H NMR (400 MHz, MeCN-d^{3}) spectra of reaction mixture at various reaction times.

Optimization of method 1 (sodium hypochlorite)

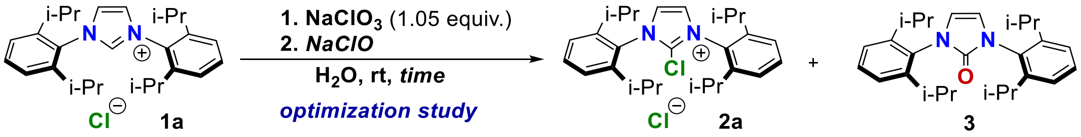

Reactions were carried out in 80 mL beaker. To a solution of 1,3-bis(2,6-diisopropylphenyl)imidazolium chloride (**1a**) (420 mg, 0.99 mmol, 1.0 equiv.) in deionized water (15 mL) was added a solution of $NaClO_3$ (110 mg, 1.03 mmol, 1.05 equiv.) in deionized water (15 mL) at room temperature forming a white precipitate. To a suspension was added a diluted solution of sodium hypochlorite (varied amount) in deionized water (5 mL) at once. Reaction mixture was left stirring for various amount of time and then vacuum filtered off. The remaining solid was washed with deionized water (2× 5 mL), dried at 70°C and analyzed by NMR spectroscopy (MeCN-d_3 solvent).

Table S1. Optimization of chlorination with sodium hypochlorite.

**Entry**	**NaOCl** [equiv.]	**Time** [h]	**isolated yield** [%]	**2a** [%]	**3** [%]
**1**	1.1	50	*n.d.*	75	2
**2**	4.21	5	*n.d.*	71	1
**3**	4.34	24	**88**	89	5
**4**	4.99	24	**86**	89	5
**5**[a]	4.30	24	**72**	76	16
**6**[a,b]	4.30	24	**75**	79	12

[a]Multigram scale (5g). [b]NaClO was added dropwise over 15 minutes.

Optimization of method 2 (calcium hypochlorite)

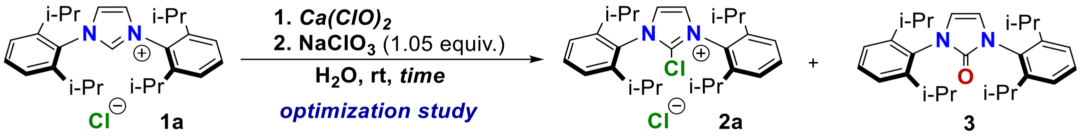

Reaction was carried out in 80 mL beaker. To a solution of 1,3-bis(2,6-diisopropylphenyl)imidazolium chloride (**1a**) (420 mg, 0.99 mmol, 1.0 equiv.) in deionized water (15 mL) was added previously prepared $Ca(ClO)_2$ reagent solution (varied amount) diluted with deionized water to total volume of 3 mL. The resulting reaction mixture was stirred for various amount of time at room temperature and then quenched with addition of $NaClO_3$ (110 mg, 1.03 mmol, 1.05 equiv.) in deionized water (5 mL) to form a white precipitate. Suspension was vacuum filtered off and the remaining solid washed with deionized water (2× 5 mL), dried at 70°C and analyzed by NMR spectroscopy (MeCN-d_3 solvent).

Table S2. Optimization of chlorination with calcium hypochlorite.

**Entry**	**$Ca(OCl)_2$** [equiv.]	**Time** [min]	**isolated yield** [%]	**2a** [%]	**3** [%]
**1**	2.0	15	*n.d.*	86	10
**2**	0.55	27	*n.d.*	89	3
**3**	0.55	37	**92**	95	5
**4**	0.55	47	*n.d.*	86	13
**5**	0.55	80	*n.d.*	83	16
**6**[a,b]	0.55	42	**89**	*n.d.*	*n.d.*

[a]Multigram scale (10g). [b]$Ca(ClO)_2$ was added dropwise over 15 minutes.

S4 Synthesis of various phenol substrates

Ethyl 4-hydroxybenzoate (4f)

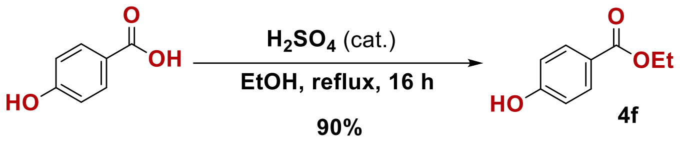

Reaction was carried out in 50 mL round-bottom flask equipped with an air condenser. To 4-hydroxybenzoic acid (691 mg, 5.0 mmol) in absolute ethanol (15 mL) was added conc. H_2SO_4 (3-5 drops). Reaction mixture was refluxed for 16 h and then concentrated under vacuum. The remaining solid was dissolved in EtOAc (10 mL), and organic phase washed with sat. $NaHCO_3$ (2× 15 mL), distilled water (10 mL) and brine (10 mL). Solvent was removed under vacuum to afford 745 mg of (**4f**) as a white solid (**90 %** yield).

NMR Spectroscopy:

^{1}**H NMR** (400 MHz, $CDCl_3$): δ 8.00 – 7.92 (m, 2H), 6.92 – 6.84 (m, 2H), 6.09 (b.s., 1H), 4.36 (q, *J* = 7.1 Hz, 2H), 1.38 (t, *J* = 7.2 Hz, 3H).

Spectroscopic data are consistent with previously reported data.[2]

Ethyl p-coumarate (4g)

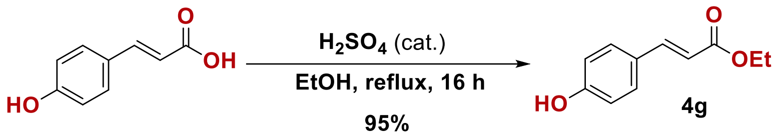

Reaction was carried out in 50 mL round-bottom flask equipped with an air condenser. To p-coumaric acid (821 mg, 5.0 mmol) in absolute ethanol (15 mL) was added conc. H_2SO_4 (3-5 drops). Reaction mixture was refluxed overnight and then concentrated under vacuum. The remaining solid was dissolved in EtOAc (10 mL), and organic phase washed with sat. $NaHCO_3$ (2× 15 mL), distilled water (10 mL) and brine (10 mL). Solvent was removed under vacuum to afford 917 mg of (**4g**) as a white solid (**95 %** yield).

NMR Spectroscopy:

^{1}**H NMR** (400 MHz, $CDCl_3$): δ 7.64 (d, *J* = 16.0 Hz, 1H), 7.46 – 7.38 (m, 2H), 6.90 – 6.82 (m, 2H), 6.30 (d, *J* = 15.9 Hz, 1H), 6.19 (s, 1H), 4.27 (q, *J* = 7.1 Hz, 2H), 1.34 (t, *J* = 7.1 Hz, 3H).

Spectroscopic data are consistent with previously reported data.[3]

Menthyl 6-hydroxynicotinate (4s)

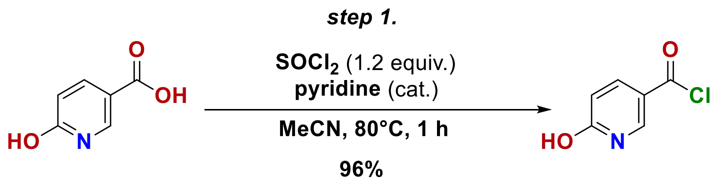

Reaction was carried out in an oven-dried 5 mL round-bottom flask equipped with an air condenser under inert Ar atmosphere. To 6-hydroxynicotinic acid (278 mg, 2.0 mmol) and pyridine (1 drop, cat.) in

anhydrous MeCN (1 mL) was added $SOCl_2$ (174 µL, 284 mg, 2.4 mmol, 1.2 equiv.) dropwise at room temperature. Reaction mixture was stirred at 80°C for 1 h, until pH paper showed no more acidic gas evolution. Once cooled down to room temperature a precipitate appeared. Volatiles were removed under vacuum to afford 302 mg of 6-hydroxynicotinoyl chloride as an off-white solid (**96%** yield). Obtained acyl chloride was used without further purification.

NMR Spectroscopy:

^{1}H NMR (400 MHz, $CDCl_3$): δ 12.41 (b.s., 1H), 8.46 (d, *J* = 2.7 Hz, 1H), 8.02 (dd, *J* = 9.6, 2.7 Hz, 1H), 6.64 (d, *J* = 9.6 Hz, 1H).

^{13}C NMR (101 MHz, $CDCl_3$): δ 165.3, 163.8, 144.3, 140.8, 120.1, 115.2.

Spectroscopic data are consistent with previously reported data.[4]

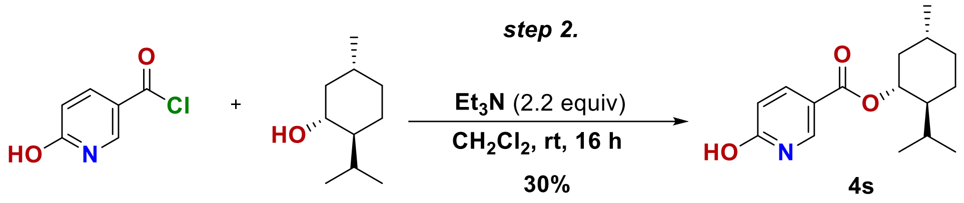

Reaction was carried out in an oven-dried 25 mL round-bottom flask under inert Ar atmosphere. To a stirred mixture of 6-hydroxynicotinoyl chloride (157 mg, 1.0 mmol) in anhydrous CH_2Cl_2 (5 mL) was added a solution of (-)-menthol (172 mg, 1.1 mmol, 1.1 equiv.) and Et_3N (0.30 mL, 218 mg, 2.2 mmol, 2.2 equiv.) in anhydrous CH_2Cl_2 (2 mL) dropwise via syringe over course of 20 min at 0°C. Reaction mixture was stirred at room temperature overnight. Once all the remaining solid had dissolved, solvent was removed under vacuum and the remaining red residue purified by flash silica gel column chromatography using gradient elution with EtOAc/hexane ranging from 1:1 to 3:1 (v/v) to afford 82 mg of (**4s**) as a pale yellow viscous oil (**30%** yield).

R_f = 0.51 (EtOAc/hexane = 3:1 (v/v))

NMR Spectroscopy:

^{1}H NMR (400 MHz, $CDCl_3$): δ 13.34 (b.s., 1H), 8.20 (d, *J* = 2.5 Hz, 1H), 8.02 (dd, *J* = 9.6, 2.5 Hz, 1H), 6.57 (d, *J* = 9.6 Hz, 1H), 4.85 (td, *J* = 10.9, 4.4 Hz, 1H), 2.11 – 2.00 (m, 2H), 1.86 (pd, *J* = 7.0, 2.6 Hz, 1H), 1.70 (dt, *J* = 11.8, 2.8 Hz, 2H), 1.58 – 1.40 (m, 2H), 1.15 – 0.97 (m, 2H), 0.90 (dd, *J* = 6.8, 4.6 Hz, 6H), 0.76 (d, *J* = 6.9 Hz, 3H).

^{13}C NMR (101 MHz, $CDCl_3$): δ 165.9, 163.7, 141.4, 139.7, 119.5, 111.9, 75.3, 47.3, 41.1, 34.3, 31.5, 26.6, 23.6, 22.1, 20.9, 16.5.

S5 Synthesis of trimethylsilyl (TMS) protected phenols

Synthesis of trimethylsilyl (TMS) protected phenols (**6**) was performed according to the modified literature procedure from corresponding phenol (**4**) and HMDS[5].

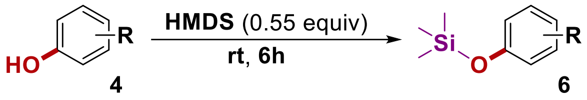

General procedure:

Reaction was performed in 7 mL borosilicate vial. To phenol (**4**) (0.5 mmol, 1.0 equiv.) was added HMDS (58 μL, 0.277 mmol, 0.55 equiv.) dropwise and the suspension stirred for up to 6 h at room temperature. Reaction completion was monitored by TLC. During the reaction liquid product formed. Reaction mixture was then extracted with Et_2O (2× 3 mL) and volatiles removed under vacuum to afford spectroscopically pure TMS protected phenols (**6**) as viscous oils in high yields.

(4-benzoylphenoxy)trimethylsilane (6a):

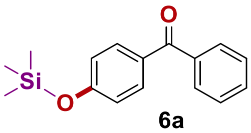

6a

Following general procedure from (**4a**), colorless oil, **81%** yield. Product was purified by flash column chromatography.

NMR Spectroscopy:

^{1}**H NMR** (600 MHz, $CDCl_3$): δ 7.81 – 7.73 (m, 4H), 7.60 – 7.54 (m, 1H), 7.48 (td, *J* = 7.8, 1.7 Hz, 2H), 6.92 (dd, *J* = 8.7, 7.8 Hz, 2H), 0.32 (s, 9H).

Spectroscopic data are consistent with previously reported data.[6]

(4-acetylphenoxy)trimethylsilane (6b):

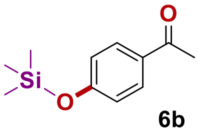

6b

Following general procedure from (**4b**), colorless oil, **94%** yield.

NMR Spectroscopy:

^{1}**H NMR** (600 MHz, $CDCl_3$): δ 7.90 (d, *J* = 8,7 Hz, 2H), 6.88 (d, *J* = 8,7 Hz, 2H), 2.57 (s, 3H), 0.32 (s, 9H)

Spectroscopic data are consistent with previously reported data.[5]

(4-nitrophenoxy)trimethylsilane (6c):

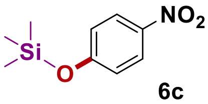

6c

Following general procedure from (**4c**), yellow oil, **92%** yield.

NMR Spectroscopy:

^{1}H NMR (600 MHz, $CDCl_3$): δ 8.15 (d, *J* = 9.1 Hz, 2H), 6.90 (d, *J* = 9.1 Hz, 2H), 0.32 (s, 9H).

Spectroscopic data are consistent with previously reported data.[5]

Ethyl 4-trimethylsilyloxybenzoate (6f):

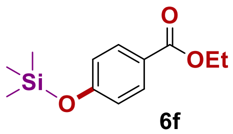

6f

Following general procedure from (**4f**), colorless oil, **87%** yield.

NMR Spectroscopy:

^{1}H NMR (600 MHz, $CDCl_3$): δ 7.95 (d, *J* = 8.7 Hz, 2H), 6.85 (d, *J* = 8.7 Hz, 2H), 4.33 (q, *J* = 7.1 Hz, 2H), 1.36 (t, *J* = 7.2 Hz, 3H), 0.27 (s, 9H).

Spectroscopic data are consistent with previously reported data.[5]

S6 Deoxyfluorination of phenol substrates

Reagent (**2b**) can be treated as a solid insensitive to air and moisture and can be stored under ambient atmosphere. Prior to use it was dried under vacuum at 70°C for 24 h in Teflon tube. DBU was used as received without further purification. Toluene and DME were dried by distillation from sodium-benzophenone ketyl and stored under molecular sieves (4Å). Phenols were used as received. Cesium fluoride and potassium fluoride were dried under vacuum at 200°C for 24 h before use.

General procedure for deoxyfluorination:

Reaction was carried out under ambient atmosphere in a closed oven-dried 7 mL borosilicate vial. To phenol (0.50 mmol, 1.0 equiv.), 1,3-bis(2,6-diisopropylphenyl)-2-chloroimidazolium dihydrogen trifluoride (**2b**) (265 mg, 0.55 mmol, 1.1 equiv.) in toluene or DME (4 mL) is added DBU (170 mg, 1.12 mmol, 2.2 equiv.) dropwise via syringe. Precipitate usually appears. Vial is then sealed, submerged in an oil bath and reaction mixture stirred at 85-100°C for 16 h (exact conditions for each compound are given below). Once cooled to room temperature, the reaction mixture is purified by flash silica gel column chromatography to afford fluorinated product (**5**) and recyclable imidazolone (**3**) side product.

4-Fluorobenzophenone (5a)

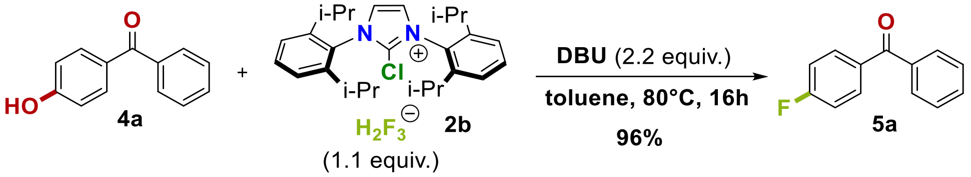

To 4-hydroxybenzophenone (**4a**) (100 mg, 0.50 mmol) and 1,3-bis(2,6-diisopropylphenyl)-2-chloroimidazolium dihydrogen trifluoride (**2b**) (265 mg, 0.55 mmol, 1.1 equiv.) in toluene (4 mL) is added DBU (171 mg, 1.1 mmol, 2.2 equiv.). Mixture is stirred at 80 °C for 16 h, and then purified by flash silica gel column chromatography eluting with EtOAc/hexane = 1:20 (v/v) to afford 97 mg of (**5a**) as a white solid (**96%** yield).

R_f = 0.51 (EtOAc/hexane = 1:10(v/v), R_f(imidazolone **3**) = 0.36)

NMR Spectroscopy:

^{1}**H NMR** (600 MHz, $CDCl_3$): δ 7.87 – 7.83 (m, 2H), 7.78 – 7.76 (m, 2H), 7.62 – 7.58 (m, 1H), 7.51 – 7.47 (m, 2H), 7.18 – 7.14 (m, 2H).

^{19}**F NMR** (565 MHz, $CDCl_3$): δ -106.0 (m).

Spectroscopic data are consistent with previously reported data.[7]

4-Fluoroacetophenone (5b)

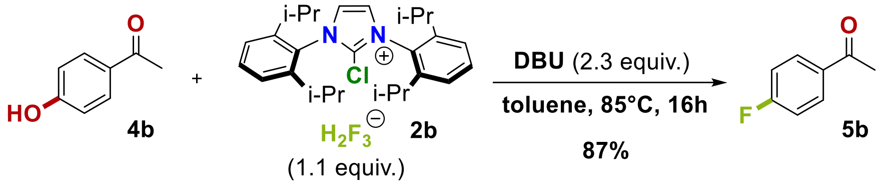

To 4-hydroxyacetophenone (**4b**) (68 mg, 0.50 mmol) and 1,3-bis(2,6-diisopropylphenyl)-2-chloroimidazolium dihydrogen trifluoride (**2b**) (270 mg, 0.55 mmol, 1.1 equiv.) in toluene (4 mL) is added

DBU (175 mg,1.14 mmol, 2.3 equiv.). Mixture is stirred at 85 °C for 16 h, and then directly purified by flash silica gel column chromatography eluting with Et_2O/hexane = 1:10 (v/v) to afford 60 mg of titled compound (**5b**) as a colorless liquid with characteristic almond-like odor (**87%** yield). *Compound* (**5b**) *is volatile and care should be taken when removing solvent under vacuum to prevent significant loses.*

R_f = 0.35 (Et_2O/hexane = 1:5(v/v), R_f(imidazolone **3**) = 0.13)

NMR Spectroscopy:

^{1}**H NMR** (600 MHz, $CDCl_3$): δ 7.99 – 7.96 (m, 2H), 7.12 (t, *J* = 8.6 Hz, 2H), 2.59 (s, 3H).

^{13}**C NMR** (151 MHz, $CDCl_3$): δ 196.5 (s), 165.8 (d, *J* = 254.6 Hz), 133.6 (d, *J* = 2.8 Hz), 131.0 (d, *J* = 9.4 Hz), 115.7 (d, *J* = 21.8 Hz), 26.5 (s).

^{19}**F NMR** (565 MHz, $CDCl_3$): δ -105.4 (ddd, J = 13.9, 8.6, 5.2 Hz).

Spectroscopic data are consistent with previously reported data.[8]

4-Fluoronitrobenzene (5c)

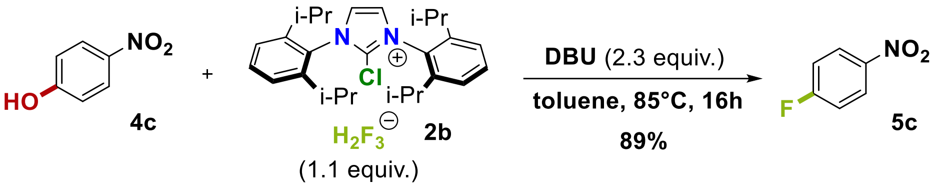

To 4-nitrophenol (**4c**) (70 mg, 0.50 mmol) and 1,3-bis(2,6-diisopropylphenyl)-2-chloroimidazolium dihydrogen trifluoride (**2b**) (271 mg, 0.56 mmol, 1.12 equiv.) in toluene (4 mL) is added DBU (174 mg, 1.11 mmol, 2.3 equiv.). Mixture is stirred at 85 °C for 16 h, and then purified by flash silica gel column chromatography eluting with Et_2O/hexane = 1:10 (v/v) to afford 63 mg of (**5c**) as a yellow oil that solidifies upon cooling with ice (**89%** yield). *Compound* (**5c**) *is volatile and care should be taken when removing solvent under vacuum to prevent significant loses.*

R_f = 0.65 (Et_2O/hexane = 1:5(v/v), R_f(imidazolone **3**) = 0.13)

NMR Spectroscopy:

^{1}**H NMR** (600 MHz, $CDCl_3$): δ 8.30 – 8.24 (m, 2H), 7.23 – 7.19 (m, 2H).

^{13}**C NMR** (151 MHz, $CDCl_3$): δ 166.4 (d, *J* = 257.8 Hz), 144.5 (s), 126.4 (d, *J* = 9.9 Hz), 116.5 (d, *J* = 23.6 Hz).

^{19}**F NMR** (565 MHz, $CDCl_3$): δ -102.0 (tt, J = 7.3, 4.7 Hz).

Spectroscopic data are consistent with previously reported data.[9]

4-Fluorobenzonitrile (5d)

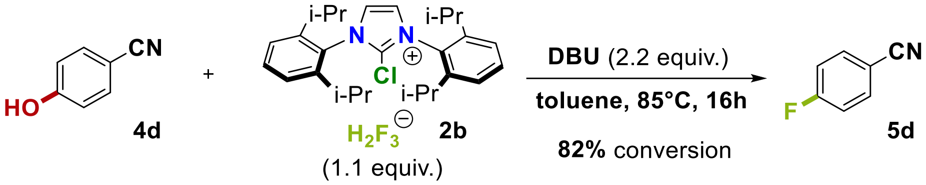

Compound (5d) was too volatile to isolate! Reaction was performed according to general procedure for determination of reaction conversions (page S34).

NMR Spectroscopy:

^{19}F NMR (565 MHz, $CDCl_3$): δ -102.4 (tt, *J* = 8.4, 5.2 Hz)

Spectroscopic data are consistent with previously reported data.[10]

4-Fluorobenzaldehyde (5e)

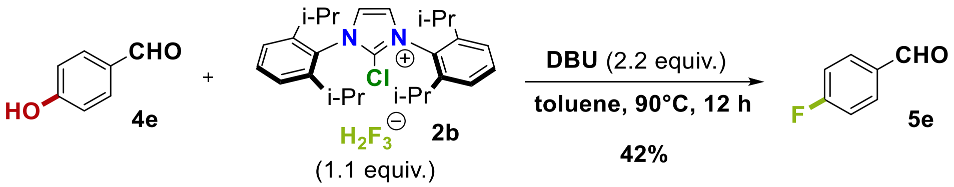

To 4-hydroxybenzaldehyde (**4e**) (61 mg, 0.50 mmol) and 1,3-bis(2,6-diisopropylphenyl)-2-chloroimidazolium dihydrogen trifluoride (**2b**) (271 mg, 0.56 mmol, 1.12 equiv.) in toluene (4 mL) is added DBU (172 mg, 1.13 mmol, 2.3 equiv.). Mixture is stirred at 105 °C for 4 h, and then purified by flash silica gel column chromatography eluting with Et_2O/hexane = 1:10 (v/v) to afford 26 mg of (**5e**) as a colorless oil (**42%** yield).

R_f = 0.31 (Et_2O/hexane = 1:10 (v/v), R_f(imidazolone **3**) = 0.08)

NMR Spectroscopy:

^{1}H NMR (600 MHz, $CDCl_3$): δ 9.97 (s, 1H), 7.91 (dd, *J* = 8.6, 5.5 Hz, 2H), 7.21 (t, *J* = 8.5 Hz, 2H).

^{13}C NMR (151 MHz, $CDCl_3$): δ 190.6, 166.7 (d, *J* = 256.7 Hz), 133.1 (d, *J* = 2.7 Hz), 132.4 (d, *J* = 9.8 Hz), 116.5 (d, *J* = 22.3 Hz).

^{19}F NMR (565 MHz, $CDCl_3$): δ -102.4 (ddd, *J* = 13.9, 8.5, 5.4 Hz).

Spectroscopic data are consistent with previously reported data.[11]

Ethyl 4-fluorobenzoate (5f)

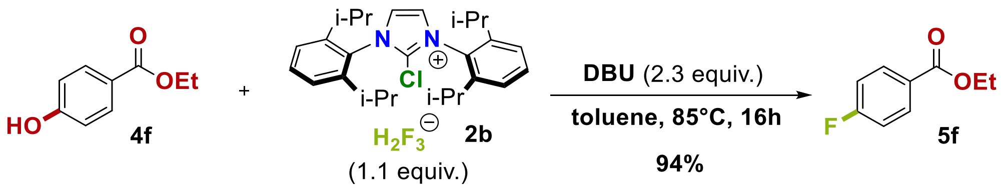

To ethyl 4-hydroxybenzoate (**4f**) (83 mg, 0.50 mmol) and 1,3-bis(2,6-diisopropylphenyl)-2-chloroimidazolium dihydrogen trifluoride (**2b**) (272 mg, 0.56 mmol, 1.13 equiv.) in toluene (4 mL) is added DBU (175 mg, 1.11 mmol, 2.3 equiv.). Mixture is stirred at 85 °C for 16 h, and then purified by flash silica gel column chromatography eluting with Et_2O/hexane = 1:10 (v/v) to afford 79 mg of (**5f**) as a white solid (**94%** yield).

R_f = 0.43 (Et_2O/hexane = 1:10(v/v), R_f(imidazolone **3**) = 0.07)

NMR Spectroscopy:

^{1}H NMR (400 MHz, $CDCl_3$): δ 8.04 (dd, *J* = 8.9, 5.3 Hz, 2H), 7.08 (t, *J* = 8.5 Hz, 2H), 4.35 (q, *J* = 7.1 Hz, 2H), 1.38 (t, *J* = 7.1 Hz, 3H).

^{13}C NMR (101 MHz, $CDCl_3$): δ 165.8 (d, *J* = 253.6 Hz), 165.7 (s), 132.1 (d, *J* = 9.3 Hz), 126.9 (d, *J* = 3.0 Hz), 115.5 (d, *J* = 22.0 Hz), 61.2 (s), 14.4 (s).

^{19}F NMR (376 MHz, $CDCl_3$): δ -106.1 (tt, *J* = 8.3, 5.4 Hz).

Spectroscopic data are consistent with previously reported data.[7]

Ethyl 4-fluorocinnamate (5g)

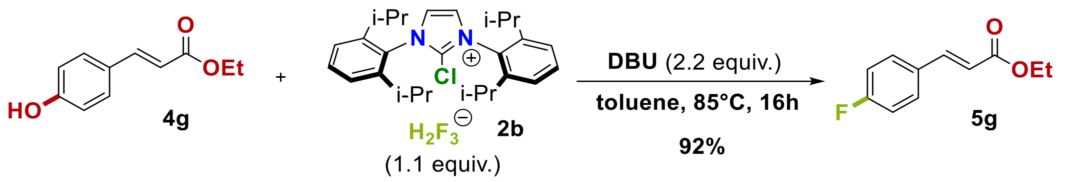

To ethyl p-coumarate (**4g**) (96 mg, 0.50 mmol) and 1,3-bis(2,6-diisopropylphenyl)-2-chloroimidazolium dihydrogen trifluoride (**2b**) (275 mg, 0.57 mmol, 1.14 equiv.) in toluene (3 mL) is added DBU (174 mg, 1.14 mmol, 2.3 equiv.). Mixture is stirred at 85 °C for 16 h, and then purified by flash silica gel column chromatography eluting with Et_2O/hexane = 1:10 (v/v) to afford 89 mg of (**5g**) as a white solid with characteristic minty odor (**92%** yield).

R_f = 0.34 (Et_2O/hexane = 1:10 (v/v), R_f (imidazolone **3**) = 0.07)

NMR Spectroscopy:

^{1}H NMR (400 MHz, $CDCl_3$): δ 7.63 (d, *J* = 16.0 Hz, 1H), 7.49 (dd, *J* = 8.6, 5.5 Hz, 2H), 7.05 (t, *J* = 8.6 Hz, 2H), 6.34 (d, *J* = 16.0 Hz, 1H), 4.25 (q, *J* = 7.1 Hz, 2H), 1.32 (t, *J* = 7.1 Hz, 3H).

^{13}C NMR (101 MHz, $CDCl_3$): δ 166.9 (s), 163.9 (d, *J* = 251.1 Hz), 143.3 (s), 130.8 (d, *J* = 3.4 Hz), 130.0 (d, *J* = 8.4 Hz), 118.1 (d, *J* = 2.2 Hz), 116.1 (d, *J* = 22.0 Hz), 60.6 (s), 14.4 (s).

^{19}F NMR (376 MHz, $CDCl_3$): δ -109.76 (tt, *J* = 8.6, 5.3 Hz).

Spectroscopic data are consistent with previously reported data.[12]

4-Fluorochalcone (5h)

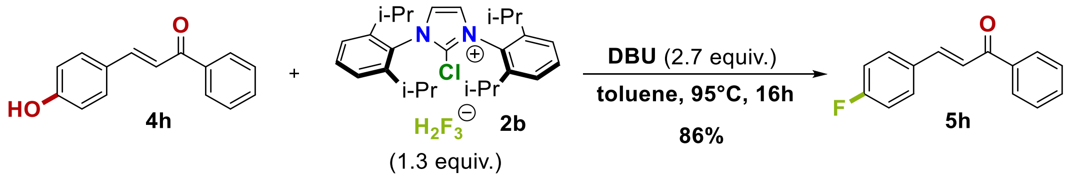

To 4-hydroxychalcone (**4h**) (112 mg, 0.50 mmol) and 1,3-bis(2,6-diisopropylphenyl)-2-chloroimidazolium dihydrogen trifluoride (**2b**) (310 mg, 0.64 mmol, 1.28 equiv.) in toluene (4 mL) is added DBU (204 mg, 1.34 mmol, 2.7 equiv.). Mixture is stirred at 95 °C for 16 h, and then purified by flash silica gel column chromatography eluting with Et_2O/hexane = 1:15 (v/v) to afford 97 mg of (**5h**) as a white solid (**86%** yield).

R_f = 0.27 (Et_2O/hexane = 1:10 (v/v), R_f (imidazolone **3**) = 0.08)

NMR Spectroscopy:

^{1}H NMR (600 MHz, $CDCl_3$): δ 8.01 (dd, *J* = 8.3, 1.4 Hz, 2H), 7.76 (d, *J* = 15.7 Hz, 1H), 7.61 (dd, *J* = 8.7, 5.5 Hz, 2H), 7.60 – 7.54 (m, 1H), 7.49 (t, *J* = 7.7 Hz, 2H), 7.45 (d, *J* = 15.7 Hz, 1H), 7.09 (t, *J* = 8.6 Hz, 2H).

^{13}C NMR (151 MHz, $CDCl_3$): δ 190.3 (s), 164.1 (d, J = 251.8 Hz), 143.5 (d, J = 0.9 Hz), 138.2 (s), 132.9 (s), 131.2 (d, J = 3.4 Hz), 130.4 (d, J = 8.6 Hz), 128.7 (s), 128.5 (s), 121.8 (d, J = 2.4 Hz), 116.2 (d, J = 21.9 Hz).

^{19}F NMR (565 MHz, $CDCl_3$): δ -109.0 (tt, J = 8.5, 5.5 Hz).

Spectroscopic data are consistent with previously reported data.[13]

4'-Fluorochalcone (5i)

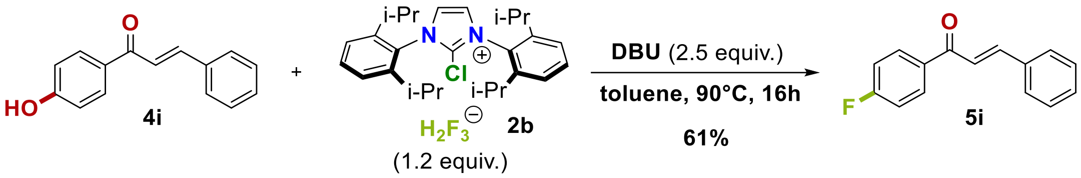

To 4'-hydroxychalcone (**4i**) (112 mg, 0.50 mmol) and 1,3-bis(2,6-diisopropylphenyl)-2-chloroimidazolium dihydrogen trifluoride (**2b**) (298 mg, 0.62 mmol, 1.23 equiv.) in toluene (4 mL) is added DBU (189 mg, 1.24 mmol, 2.5 equiv.). Mixture is stirred at 90 °C for 16 h, and then purified by flash silica gel column chromatography eluting with EtOAc/hexane = 1:20 (v/v) to afford 69 mg of (**5i**) as a white solid (**61%** yield).

Using DME (4 mL) instead of toluene afforded 97 mg of (**5i**) (**86%** yield).

R_f = 0.56 (EtOAc/hexane = 1:10 (v/v), R_f (imidazolone **3**) = 0.37)

NMR Spectroscopy:

^{1}H NMR (400 MHz, $CDCl_3$): δ 8.10 – 8.03 (m, 2H), 7.82 (d, J = 15.7 Hz, 1H), 7.64 (dd, J = 6.6, 3.0 Hz, 2H), 7.50 (d, J = 15.7 Hz, 1H), 7.44 – 7.39 (m, 3H), 7.21 – 7.14 (m, 2H).

^{13}C NMR (101 MHz, $CDCl_3$): δ 188.9 (s), 165.7 (d, J = 254.4 Hz), 145.1 (s), 134.9 (s), 134.6 (d, J = 3.0 Hz), 131.2 (d, J = 9.2 Hz), 130.8 (s), 129.1 (s), 128.6 (s), 121.7 (s), 115.9 (d, J = 21.8 Hz).

^{19}F NMR (376 MHz, $CDCl_3$): δ -105.5 (tt, J = 8.5, 5.5 Hz).

Spectroscopic data are consistent with previously reported data.[14]

3-Fluoronitrobenzene (5j)

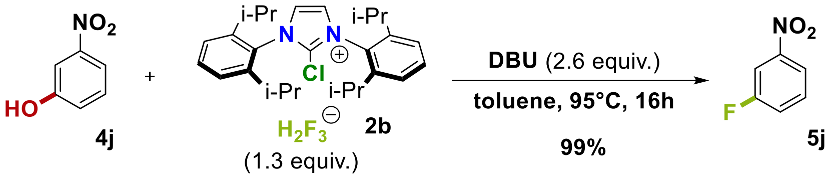

To 3-nitrophenol (**4j**) (70 mg, 0.50 mmol) and 1,3-bis(2,6-diisopropylphenyl)-2-chloroimidazolium dihydrogen trifluoride (**2b**) (315 mg, 0.65 mmol, 1.30 equiv.) in toluene (4 mL) is added DBU (200 mg, 1.31 mmol, 2.6 equiv.). Mixture is stirred at 95 °C for 16 h, and then purified by flash silica gel column chromatography eluting with Et_2O/hexane = 1:20 (v/v) to afford 70 mg of (**5j**) as a yellow oil (**99%** yield).

R_f = 0.49 (Et_2O/hexane = 1:10 (v/v), R_f (imidazolone **3**) = 0.08)

NMR Spectroscopy:

^{1}**H NMR** (400 MHz, $CDCl_3$): δ 8.06 (dd, *J* = 8.3, 2.1 Hz, 1H), 7.94 (dt, *J* = 8.6, 2.4 Hz, 1H), 7.55 (td, *J* = 8.3, 5.5 Hz, 1H), 7.43 (td, *J* = 8.1, 2.5 Hz, 1H).

^{13}**C NMR** (101 MHz, $CDCl_3$): δ 162.5 (d, J = 251.2 Hz), 149.3, 130.9 (d, J = 8.2 Hz), 122.0 (d, J = 21.4 Hz), 119.5 (d, J = 3.4 Hz), 111.5 (d, J = 26.3 Hz).

^{19}**F NMR** (376 MHz, $CDCl_3$): δ -109.0 (td, *J* = 8.2, 5.5 Hz).

Spectroscopic data are consistent with previously reported data.[15]

4-Fluoro-1-chloro-2-nitrobenzene (5k)

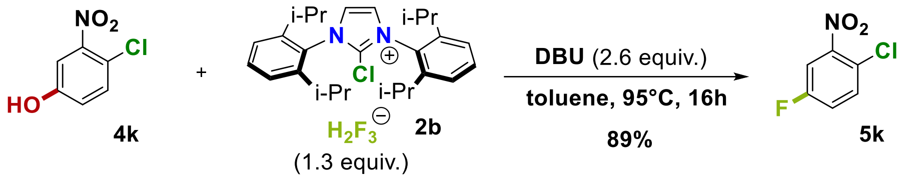

To 4-chloro-3-nitrophenol (**4k**) (87 mg, 0.50 mmol) and 1,3-bis(2,6-diisopropylphenyl)-2-chloroimidazolium dihydrogen trifluoride (**2b**) (312 mg, 0.65 mmol, 1.30 equiv.) in toluene (4 mL) is added DBU (198 mg, 1.30 mmol, 2.6 equiv.). Mixture is stirred at 95 °C for 16 h, and then purified by flash silica gel column chromatography eluting with Et_2O/hexane = 1:20 (v/v) to afford 78 mg of (**5k**) as a yellow solid (**89%** yield).

R_f = 0.41 (Et_2O/hexane = 1:10 (v/v), R_f(imidazolone **3**) = 0.08)

NMR Spectroscopy:

^{1}**H NMR** (400 MHz, $CDCl_3$): δ 7.63 (dd, J = 7.6, 3.0 Hz, 1H), 7.55 (dd, J = 8.9, 4.9 Hz, 1H), 7.28 (ddd, J = 8.7, 7.1, 2.9 Hz, 1H).

^{13}**C NMR** (101 MHz, $CDCl_3$): δ 160.5 (d, *J* = 252.5 Hz), 148.2, 133.3 (d, *J* = 7.9 Hz), 122.6 (d, *J* = 4.2 Hz), 120.9 (d, *J* = 22.5 Hz), 113.5 (d, *J* = 27.6 Hz).

^{19}**F NMR** (376 MHz, $CDCl_3$): δ -110.7 (td, *J* = 7.4, 4.8 Hz).

Spectroscopic data are consistent with previously reported data.[16]

1-Fluoro-3,5-dichlorobenzene (5l)

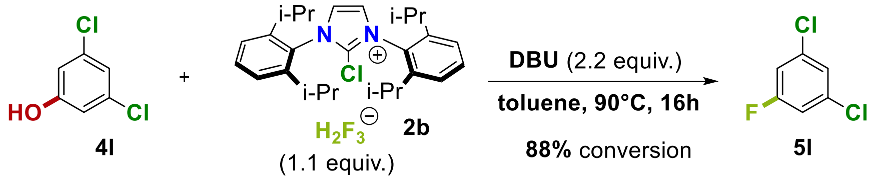

Compound (5l) was too volatile to isolate! Reaction was performed according to general procedure for determination of reaction conversions (page S34).

NMR Spectroscopy:

^{19}**F NMR** (376 MHz, C_6D_6): δ -109.0 (td, *J* = 8.3, 1.1 Hz).

Spectroscopic data are consistent with previously reported data.[7]

7-Fluorocoumarin (5m)

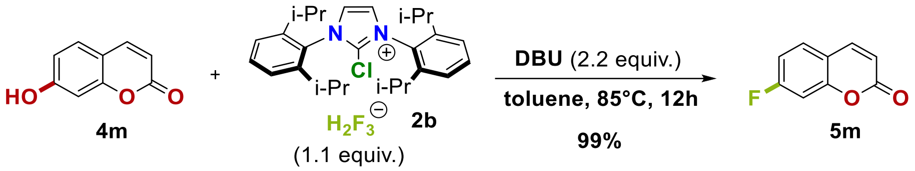

To umbelliferone (**4m**) (81 mg, 0.50 mmol) and 1,3-bis(2,6-diisopropylphenyl)-2-chloroimidazolium dihydrogen trifluoride (**2b**) (269 mg, 0.56 mmol, 1.11 equiv.) in toluene (4 mL) is added DBU (170 mg, 1.11 mmol, 2.2 equiv.). Mixture is stirred at 85 °C for 12 h, and then purified by flash silica gel column chromatography eluting with EtOAc/hexane = 1:8 (v/v) to afford 81 mg of (**5m**) as a white solid with characteristic sweet odor (**99%** yield).

$\boldsymbol{R_f}$ = 0.42 (EtOAc/hexane = 1:5 (v/v), R_f (imidazolone **3**) = 0.45)

NMR Spectroscopy:

^{1}**H NMR** (400 MHz, $CDCl_3$): δ 7.70 (d, J = 9.6, 1H), 7.50 (dd, J = 8.6, 6.0 Hz, 1H), 7.10 – 7.02 (m, 2H), 6.40 (d, J = 9.6, 1H).

^{19}**F NMR** (376 MHz, $CDCl_3$): δ -105.0 (td, J = 8.7, 5.9 Hz).

Spectroscopic data are consistent with previously reported data.[17]

8-Fluoro-5-nitroquinoline (5n)

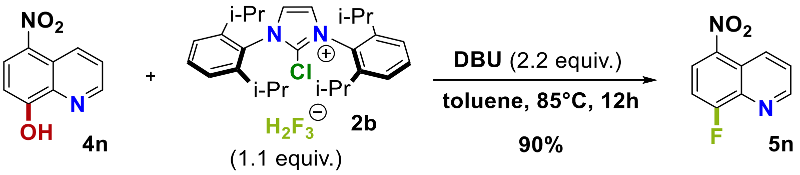

To nitroxoline (**4n**) (48 mg, 0.252 mmol) and 1,3-bis(2,6-diisopropylphenyl)-2-chloroimidazolium dihydrogen trifluoride (**2b**) (134 mg, 0.277 mmol, 1.1 equiv.) in toluene (2 mL) is added DBU (85 mg, 0.558 mmol, 2.2 equiv.). Mixture is stirred at 85°C for 12 h, then cooled down and purified by flash silica gel column chromatography using gradient elution with EtOAc/hexane ranging from 1:10 to 1:4 (v/v) to afford 43 mg of (**5n**) as a white solid (**90%** yield).

$\boldsymbol{R_f}$ = 0.15 (EtOAc/hexane = 1:5(v/v), R_f (imidazolone **3**) = 0.45)

NMR Spectroscopy:

^{1}**H NMR** (600 MHz, $CDCl_3$): δ 9.15 (d, J = 8.9 Hz, 1H), 9.10 (d, J = 4.1 Hz, 1H), 8.48 (dd, J = 8.7, 4.6 Hz, 1H), 7.75 (dd, J = 8.9, 4.1 Hz, 1H), 7.51 (t, J = 8.7 Hz, 1H).

^{13}**C NMR** (151 MHz, $CDCl_3$): δ 161.7 (d, J = 268.7 Hz), 151.7, 138.1 (d, J = 12.0 Hz), 132.5 (d, J = 2.3 Hz), 126.2 (d, J = 10.1 Hz), 125.1, 123.4 (d, J = 2.6 Hz), 112.1 (d, J = 21.7 Hz). (*one C atom not visible*)

^{19}**F NMR** (565 MHz, $CDCl_3$): δ -110.2 (dd, J = 8.8, 4.5 Hz).

Spectroscopic data are consistent with previously reported data.[18]

4-Fluoroazobenzene (5o)

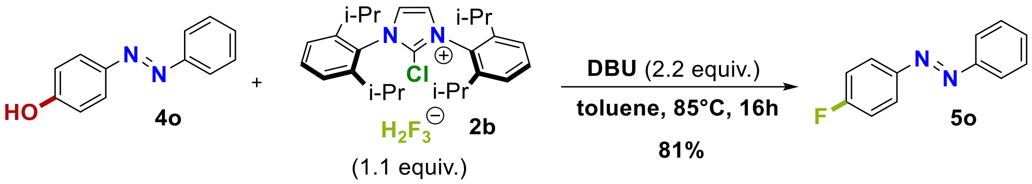

To 4-phenylazophenol (**4o**) (99 mg, 0.50 mmol) and 1,3-bis(2,6-diisopropylphenyl)-2-chloroimidazolium dihydrogen trifluoride (**2b**) (270 mg, 0.56 mmol, 1.12 equiv.) in toluene (4 mL) is added DBU (170 mg, 1.11 mmol, 2.2 equiv.). Mixture is stirred at 85 °C for 16 h, and then purified by flash silica gel column chromatography eluting with EtOAc/hexane = 1:15 (v/v) to afford 81 mg of (**5o**) as an orange solid (**81%** yield).

$\boldsymbol{R_f}$ = 0.82 (EtOAc/hexane = 1:15(v/v), R_f (imidazolone **3**) = 0.15)

NMR Spectroscopy:

^{1}H NMR (600 MHz, $CDCl_3$): δ 7.95 (dd, *J* = 8.6, 5.3 Hz, 2H), 7.91 (d, *J* = 7.7 Hz, 2H), 7.52 (t, *J* = 7.5 Hz, 2H), 7.48 (t, *J* = 7.5 Hz, 1H), 7.20 (t, *J* = 8.4 Hz, 2H).

^{19}F NMR (565 MHz, $CDCl_3$): δ -109.4 (tt, *J* = 8.2, 5.6 Hz).

Spectroscopic data are consistent with previously reported data.[19]

4-Fluoro-3-methoxybenzaldehyde (5p)

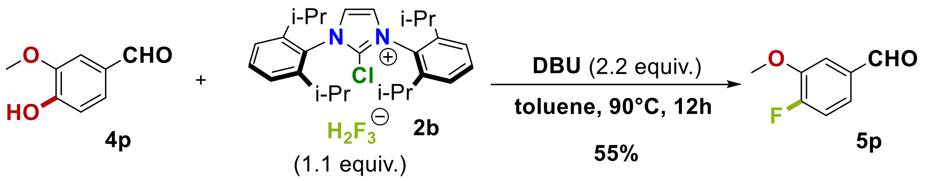

To vanillin (**4p**) (76 mg, 0.50 mmol) and 1,3-bis(2,6-diisopropylphenyl)-2-chloroimidazolium dihydrogen trifluoride (**2b**) (272 mg, 0.55 mmol, 1.12 equiv.) in toluene (4 mL) is added 170 mg of DBU (1.11 mmol, 2.2 equiv.). Mixture is stirred at 90 °C for 12 h, and then purified by flash silica gel column chromatography eluting with Et_2O/hexane = 1:10 (v/v) to afford 42 mg of (**5p**) as a white solid (**55%** yield).

$\boldsymbol{R_f}$ = 0.20 (Et_2O/hexane = 1:10 (v/v), R_f (imidazolone **3**) = 0.09)

NMR Spectroscopy:

^{1}H NMR (400 MHz, $CDCl_3$): δ 9.91 (s, 1H), 7.50 (dd, *J* = 8.2, 1.9 Hz, 1H), 7.44 (ddd, *J* = 8.2, 4.5, 1.9 Hz, 1H), 7.22 (dd, *J* = 10.5, 8.2 Hz, 1H), 3.95 (s, 3H).

^{13}C NMR (101 MHz, $CDCl_3$): δ 190.7, 156.5 (d, *J* = 257.5 Hz), 148.7 (d, *J* = 11.1 Hz), 133.3 (d, *J* = 3.1 Hz), 125.3 (d, *J* = 8.4 Hz), 116.5 (d, *J* = 19.5 Hz), 111.8 (d, *J* = 3.6 Hz), 56.3.

^{19}F NMR (376 MHz, $CDCl_3$): δ -123.8 (ddd, *J* = 10.4, 8.2, 4.4 Hz).

Spectroscopic data are consistent with previously reported data.[20]

4,4'-Difluorobenzophenone (5q)

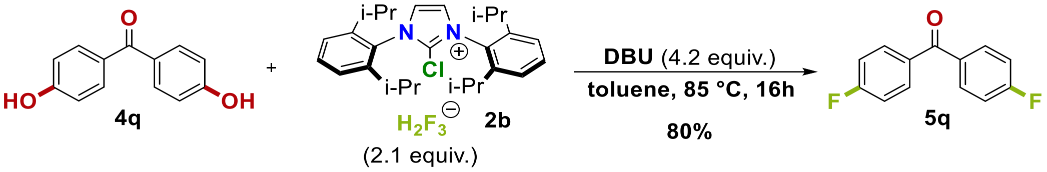

To 4,4'-dihydroxybenzophenone (**4q**) (107 mg, 0.50 mmol) and 1,3-bis(2,6-diisopropylphenyl)-2-chloroimidazolium dihydrogen trifluoride (**2b**) (507 mg, 1.05 mmol, 2.1 equiv.) in toluene (4 mL) is added DBU (320 mg, 2.1 mmol, 4.2 equiv.). Mixture is stirred at 85 °C for 16 h, and then purified by flash silica gel column chromatography eluting with Et_2O/hexane = 1:15 (v/v) to afford 81 mg of (**5q**) as a white solid (**80%** yield).

***R**$_f$* = 0.46 (Et_2O/hexane = 1:10 (v/v), *R$_f$*(imidazolone **3**) = 0.08)

NMR Spectroscopy:

^{1}H NMR (400 MHz, $CDCl_3$): δ 7.80 (td, J = 5.5, 2.3 Hz, 2H), 7.15 (t, J = 8.5 Hz, 2H).

^{13}C NMR (101 MHz, $CDCl_3$): δ 193.9, 165.5 (d, *J* = 254.3 Hz), 133.8 (d, *J* = 3.1 Hz), 132.6 (d, *J* = 9.2 Hz), 115.6 (d, *J* = 22.0 Hz).

^{19}F NMR (376 MHz, $CDCl_3$): δ -105.8 (tt, *J* = 8.3, 5.4 Hz).

Spectroscopic data are consistent with previously reported data.[21]

Methyl 5-fluoronicotinate (5r)

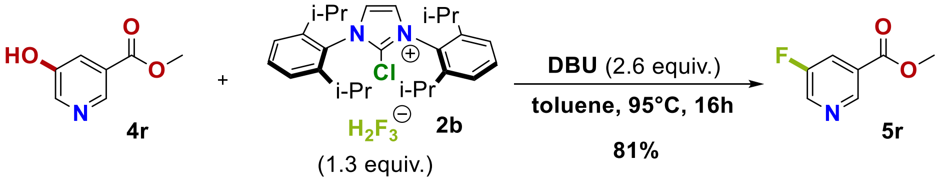

To methyl 5-hydroxynicotinate (**4r**) (77 mg, 0.50 mmol) and 1,3-bis(2,6-diisopropylphenyl)-2-chloroimidazolium dihydrogen trifluoride (**2b**) (308 mg, 0.64 mmol, 1.27 equiv.) in toluene (4 mL) is added DBU (195 mg, 1.28 mmol, 2.6 equiv.). Mixture is stirred at 95 °C for 16 h, and then purified by flash silica gel column chromatography eluting with Et_2O/hexane = 1:10 (v/v) to afford 63 mg of (**5r**) as a white crystalline solid (**81%** yield).

***R**$_f$* = 0.12 (Et_2O/hexane = 1:10 (v/v), *R$_f$*(imidazolone **3**) = 0.08)

NMR Spectroscopy:

^{1}H NMR (400 MHz, $CDCl_3$): δ 9.01 (s, 1H), 8.62 (d, *J* = 2.9 Hz, 1H), 7.96 (dt, *J* = 8.7, 2.3 Hz, 1H), 3.95 (s, 3H).

^{13}C NMR (101 MHz, $CDCl_3$): δ 164.6 (d, *J* = 2.2 Hz), 159.1 (d, *J* = 258.3 Hz), 146.6 (d, *J* = 4.3 Hz), 142.1 (d, *J* = 23.1 Hz), 127.4 (d, *J* = 3.6 Hz), 123.6 (d, *J* = 19.5 Hz), 52.72.

^{19}F NMR (376 MHz, $CDCl_3$): δ -126.0 (dd, *J* = 8.7, 1.5 Hz).

Spectroscopic data are consistent with previously reported data.[22]

Menthyl 6-fluoronicotinate (5s)

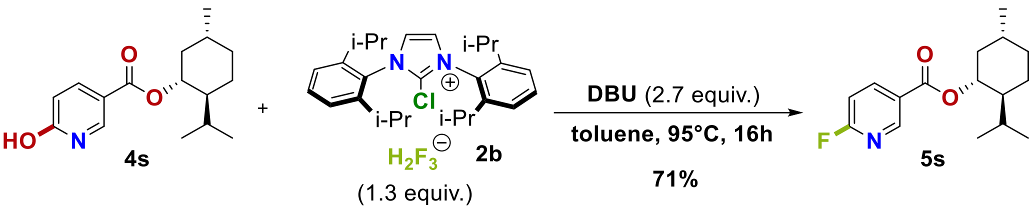

To menthyl 6-hydroxynicotinate (**4s**) (42 mg, 0.15 mmol) and 1,3-bis(2,6-diisopropylphenyl)-2-chloroimidazolium dihydrogen trifluoride (**2b**) (95 mg, 0.20 mmol, 1.3 equiv.) in toluene (1.5 mL) is added DBU (62 mg, 0.41 mmol, 2.7 equiv.). Mixture is stirred at 95 °C for 16 h, and then purified by flash silica gel column chromatography eluting with Et_2O/hexane = 1:25 (v/v) to afford 30 mg of (**5s**) as a colorless oil (**71%** yield).

***R*$_f$** = 0.56 (Et_2O/hexane = 1:10 (v/v), R_f(imidazolone **3**) = 0.07)

NMR Spectroscopy:

^{1}H NMR (600 MHz, $CDCl_3$): δ 8.87 (d, *J* = 2.5 Hz, 1H), 8.39 (ddd, *J* = 8.5, 7.6, 2.4 Hz, 1H), 6.99 (ddd, *J* = 8.5, 2.8, 0.7 Hz, 1H), 4.95 (td, *J* = 10.9, 4.5 Hz, 1H), 2.14 – 2.07 (m, 1H), 1.90 (pd, *J* = 7.0, 2.8 Hz, 1H), 1.77 – 1.70 (m, 2H), 1.55 (dddd, *J* = 14.2, 7.9, 3.9, 2.0 Hz, 2H), 1.14 – 1.09 (m, 2H), 0.92 (dd, *J* = 10.4, 6.8 Hz, 6H), 0.79 (d, *J* = 7.0 Hz, 3H).

^{13}C NMR (151 MHz, $CDCl_3$): δ 165.9 (d, *J* = 245.2 Hz), 163.9, 150.4 (d, *J* = 16.4 Hz), 142.7 (d, *J* = 9.2 Hz), 125.2 (d, *J* = 4.4 Hz), 109.5 (d, *J* = 37.6 Hz), 75.9, 47.3, 41.0, 34.3, 31.6, 26.7, 23.7, 22.1, 20.9, 16.6.

^{19}F NMR (565 MHz, $CDCl_3$): δ -61.8 (dd, *J* = 7.6, 2.9 Hz).

Compound (**5s**) is a new compound not reported before in literature.

2-Butyl-3-(4-fluorobenzoyl)benzofuran (5t)

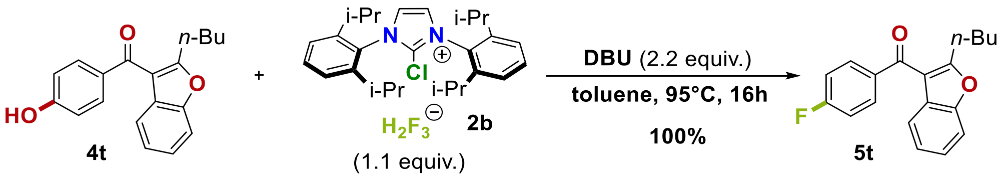

To 2-butyl-3-(4-hydroxybenzoyl)benzofuran (**4t**) (148 mg, 0.50 mmol) and 1,3-bis(2,6-diisopropylphenyl)-2-chloroimidazolium dihydrogen trifluoride (**2b**) (277 mg, 0.66 mmol, 1.14 equiv.) in toluene (4 mL) is added DBU (174 mg, 1.14 mmol, 2.3 equiv.). Mixture is stirred at 95 °C for 16 h, and then purified by flash silica gel column chromatography eluting with Et_2O/hexane = 1:25 (v/v) to afford 149 mg of (**5t**) as a colorless oil (**100%** yield).

***R*$_f$** = 0.61 (Et_2O/hexane = 1:10 (v/v), R_f(imidazolone **3**) = 0.07)

NMR Spectroscopy:

^{1}H NMR (600 MHz, $CDCl_3$): δ 7.90 (dd, *J* = 8.6, 5.5 Hz, 2H), 7.51 (d, *J* = 8.3 Hz, 1H), 7.35 (d, *J* = 7.8 Hz, 1H), 7.31 (t, *J* = 8.0 Hz, 1H), 7.22 (t, *J* = 7.2 Hz, 1H), 7.19 (t, *J* = 8.6 Hz, 2H), 2.96 (t, *J* = 7.6 Hz, 2H), 1.80 (p, *J* = 7.6 Hz, 2H), 1.40 (h, *J* = 7.4 Hz, 2H), 0.94 (t, *J* = 7.4 Hz, 3H).

^{13}C NMR (151 MHz, $CDCl_3$): δ 190.4, 165.8, 165.6 (d, J = 254.4 Hz), 153.7, 135.7 (d, J = 2.8 Hz), 131.9 (d, J = 9.2 Hz), 126.9, 124.5, 123.6, 121.2, 116.5, 115.7 (d, J = 21.9 Hz), 111.2, 30.2, 28.0, 22.5, 13.8.

^{19}F NMR (565 MHz, $CDCl_3$): δ -105.5 (tt, J = 8.4, 5.6 Hz).

Compound (**5t**) is a new compound not reported before in literature.

4-Fluoro(methylsulfonyl)benzene (5u)

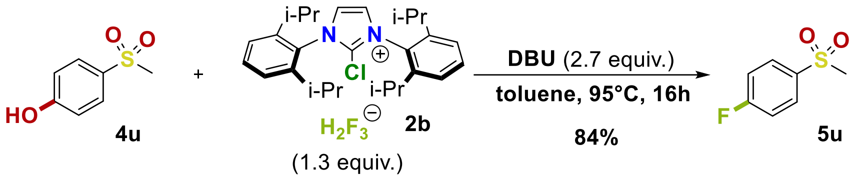

To 4-(methylsulfonyl)phenol (**4u**) (86 mg, 0.50 mmol) and 1,3-bis(2,6-diisopropylphenyl)-2-chloroimidazolium dihydrogen trifluoride (**2b**) (317 mg, 0.66 mmol, 1.31 equiv.) in toluene (4 mL) is added DBU (210 mg, 1.38 mmol, 2.76 equiv.). Mixture is stirred at 95 °C for 16 h, and then purified by flash silica gel column chromatography using gradient elution with EtOAc/hexane ranging from 1:5 to 1:3 (v/v) to afford 73 mg of (**5u**) as a white solid (**84%** yield).

R_f = 0.12 (EtOAc/hexane = 1:5 (v/v), R_f (imidazolone **3**) = 0.45)

NMR Spectroscopy:

^{1}H NMR (400 MHz, $CDCl_3$): δ 7.94 (dd, J = 8.9, 5.0 Hz, 2H), 7.22 (t, J = 8.6 Hz, 2H), 3.03 (s, 3H).

^{13}C NMR (101 MHz, $CDCl_3$): δ 165.8 (d, J = 256.1 Hz), 136.7 (d, J = 3.1 Hz), 130.3 (d, J = 9.6 Hz), 116.7 (d, J = 22.8 Hz), 44.7.

^{19}F NMR (376 MHz, $CDCl_3$): δ -103.6 (tt, J = 8.2, 5.1 Hz).

Spectroscopic data are consistent with previously reported data.[7]

7-Fluoroflavone (5v)

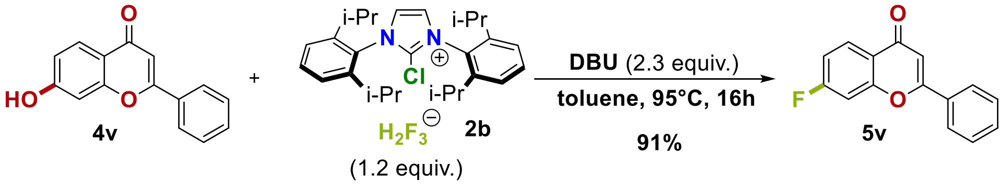

To 7-hydroxyflavone (**4v**) (119 mg, 0.50 mmol) and 1,3-bis(2,6-diisopropylphenyl)-2-chloroimidazolium dihydrogen trifluoride (**2b**) (278 mg, 0.58 mmol, 1.2 equiv.) in toluene (4 mL) is added DBU (172 mg, 1.13 mmol, 2.3 equiv.). Mixture is stirred at 95 °C for 16 h, and then purified by flash silica gel column chromatography eluting with EtOAc/hexane = 1:7 (v/v) to afford 110 mg of (**5v**) as a white solid (**91%** yield).

R_f = 0.22 (EtOAc/hexane = 1:5 (v/v), R_f (imidazolone **3**) = 0.49)

NMR Spectroscopy:

^{1}H NMR (600 MHz, $CDCl_3$): δ 8.20 (dd, J = 8.9, 6.3 Hz, 1H), 7.86 (d, J = 6.8 Hz, 2H), 7.53 – 7.46 (m, 3H), 7.21 (dd, J = 9.0, 2.4 Hz, 1H), 7.11 (td, J = 8.5, 2.4 Hz, 1H), 6.76 (s, 1H).

^{13}**C NMR** (151 MHz, $CDCl_3$): δ 177.4, 165.7 (d, *J* = 254.7 Hz), 163.7, 131.8, 131.4, 129.1, 128.2 (d, *J* = 10,5 Hz), 126.3, 120.8 (d, *J* = 2.2 Hz), 114.0 (d, *J* = 22.8 Hz), 107.6, 104.8 (d, *J* = 25.2 Hz).

^{19}**F NMR** (565 MHz, $CDCl_3$): δ -102.8 (q, *J* = 8.2, 7.6 Hz).

Spectroscopic data are consistent with previously reported data.[23]

Methyl 2-(6-fluoro-3-oxoxanthen-9-yl)benzoate (5w)

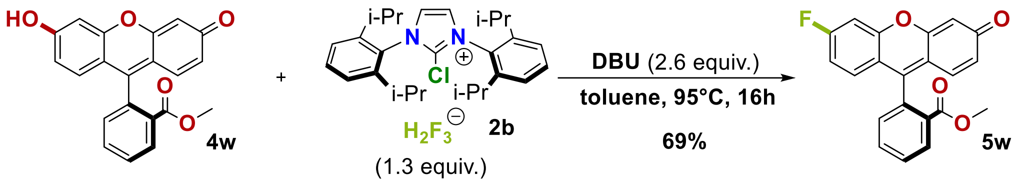

To fluorescein methyl ester (**4w**) (175 mg, 0.50 mmol) and 1,3-bis(2,6-diisopropylphenyl)-2-chloroimidazolium dihydrogen trifluoride (**2b**) (307 mg, 0.64 mmol, 1.27 equiv.) in toluene (4 mL) is added DBU (198 mg, 1.30 mmol, 2.6 equiv.). Mixture is stirred at 95 °C for 16 h, and then purified by flash silica gel column chromatography using gradient elution with EtOAc/hexane ranging from 1:2 to 3:1 (v/v) to afford 121 mg of (**5w**) as an orange crystalline solid (**69%** yield).

R_f = 0.24 (EtOAc/hexane = 1:1 (v/v), R_f (imidazolone **3**) = 0.92)

NMR Spectroscopy:

^{1}**H NMR** (600 MHz, $CDCl_3$): δ 8.24 (dd, *J* = 7.9, 1.4 Hz, 1H), 7.74 (td, *J* = 7.5, 1.4 Hz, 1H), 7.67 (td, *J* = 7.7, 1.3 Hz, 1H), 7.30 (dd, *J* = 7.6, 1.3 Hz, 1H), 7.13 (dd, *J* = 9.0, 2.5 Hz, 1H), 6.93 (dd, *J* = 8.9, 6.0 Hz, 1H), 6.88 – 6.81 (m, 2H), 6.49 (dd, *J* = 9.8, 1.9 Hz, 1H), 6.39 (d, *J* = 1.9 Hz, 1H), 3.63 (s, 3H).

^{13}**C NMR** (151 MHz, $CDCl_3$): δ 185.4, 165.0, 164.6 (d, *J* = 256.2 Hz), 158.1, 152.8 (d, *J* = 13.4 Hz), 148.5, 133.8, 132.5, 130.9, 130.2, 130.1, 129.9, 129.7, 129.5, 129.1 (d, *J* = 10.3 Hz), 118.7 (d, *J* = 2.5 Hz), 117.5 (d, *J* = 2.5 Hz), 112.2 (d, *J* = 22.8 Hz), 105.8, 103.9 (d, *J* = 25.9 Hz), 60.0.

^{19}**F NMR** (565 MHz, $CDCl_3$): δ -102.6 (d, *J* = 7.7 Hz).

Crystal Structure: Full data available on page S48.

Compound (**5w**) is a new compound not reported before in literature.

S7 Deoxyfluorination on large scale synthesis

4-hydroxybenzophenone (**4a**) was chosen as appropriate standard substrate on large scale (2.5 mmol) deoxyfluorination reaction.

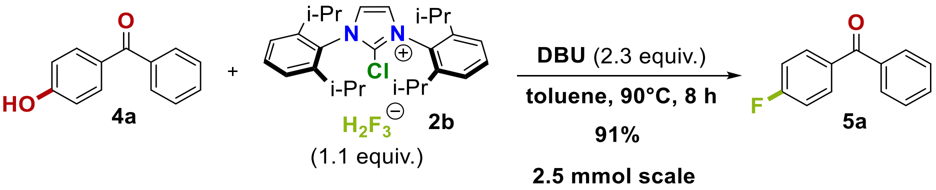

Reaction was carried out in 50 ml round bottom flask. To 4-hydroxybenzophenone (**4a**) (500 mg, 2.50 mmol) and 1,3-bis(2,6-diisopropylphenyl)-2-chloroimidazolium dihydrogen trifluoride (**2b**) (1.342 g, 2.78 mmol, 1.1 equiv.) in toluene (20 mL) was added DBU (876 mg, 5.75 mmol, 2.3 equiv.) dropwise via syringe. Mixture was stirred for 8 h at 90 °C in an oil bath and then quenched by addition of dist. H_2O (30 mL). Organic phase was extracted with Et_2O (15 mL) and dried over anh. Na_2SO_4. Volatiles were removed under vacuum and residue purified by flash silica gel column chromatography eluting with EtOAc/hexane = 1:50 (v/v) to afford 453 mg of (**5a**) as a white solid (**91%** yield).

$\boldsymbol{R_f}$ = 0.51 (EtOAc/hexane = 1:10(v/v), R_f(imidazolone **3**) = 0.36)

NMR Spectroscopy:

> **^{1}H NMR** (600 MHz, $CDCl_3$): δ 7.87 – 7.83 (m, 2H), 7.78 – 7.76 (m, 2H), 7.62 – 7.58 (m, 1H), 7.51 – 7.47 (m, 2H), 7.18 – 7.14 (m, 2H).
>
> **^{19}F NMR** (565 MHz, $CDCl_3$): δ -106.0 (m).

Spectroscopic data are consistent with previously reported data.[7]

S8 Evaluation of different bases for deoxyfluorination

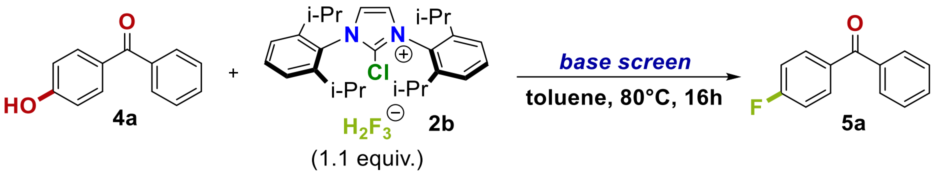

General procedure for solid bases (CsF, KF, Cs_2CO_3 and K_2CO_3):

An oven-dried borosilicate vial is charged consecutively with 4-hydroxybenzophenone (**4a**) (50 mg, 0.25 mmol, 1.0 equiv.), 1,3-bis(2,6-diisopropylphenyl)-2-chloroimidazolium dihydrogen trifluoride (**2b**) (132 mg, 0.27 mmol, 1.1 equiv.), *solid base* and toluene (2 mL). Vial is then sealed, submerged in an oil bath and reaction mixture stirred at 80°C for 16 h. Once cooled down to room temperature, internal standard (2-nitrobenzotrifluoride in toluene, 0.083 M, 1.0 mL, 0.25 mmol of fluorine) is added and the reaction mixture analyzed with quantitative ^{19}F NMR spectroscopy by comparing peak integrals to determine reaction conversion to product (**5a**).

General procedure for liquid bases (DIPEA, Et_3N, pyridine, TMG, DBU):

An oven-dried borosilicate vial is charged consecutively with 4-hydroxybenzophenone (**4a**) (50 mg, 0.25 mmol, 1.0 equiv.), 1,3-bis(2,6-diisopropylphenyl)-2-chloroimidazolium dihydrogen trifluoride (**2b**) (132 mg, 0.27 mmol, 1.1 equiv.) and toluene (2 mL). To mixture is added *liquid base* dropwise via syringe. Vial is then sealed, submerged in an oil bath and reaction mixture stirred at 80°C for 16 h. Once cooled down to room temperature, internal standard (2-nitrobenzotrifluoride in toluene, 0.083 M, 1.0 mL, 0.25 mmol of fluorine) is added and the reaction mixture analyzed with quantitative ^{19}F NMR spectroscopy by comparing peak integrals to determine reaction conversion to product (**5a**).

Table S3. Deoxyfluorination base screen.

**Entry**	**2b** [equiv.]	**Time** [h]	**T** [°C]	**Solvent**	**Base** [equiv.]	**Conversion** (**5a**) [%]
**1**	1.1	16	80	Toluene	none	**0**
**2**	1.1	16	80	Toluene	CsF (8.0)	**>99**
**3**	1.1	16	80	Toluene	KF (8.0)	**95**
**4**	1.1	16	80	Toluene	Cs_2CO_3 (2.2)	**0**
**5**	1.1	16	80	Toluene	K_2CO_3 (2.2)	**1**
**6**	1.1	16	80	Toluene	DIPEA (2.4)	**4**
**7**	1.1	16	80	Toluene	Et_3N (2.6)	**6**
**8**	1.1	16	80	Toluene	Pyridine (2.9)	**0**
**9**	1.1	16	80	Toluene	TMG (2.2)	**>99**
**10**	1.1	16	80	Toluene	DBU (2.2)	**>99**
**11**	1.1	16	80	Toluene	DBU (1.4)	**52**
**12**	1.1	16	80	Toluene	DBU (3.4)	**>99**
**13**	1.1	16	80	Toluene	DBU (4.7)	**90**
**14**	1.1	16	80	Toluene	DBU (5.8)	**92**

S9 Evaluation of different solvents for deoxyfluorination

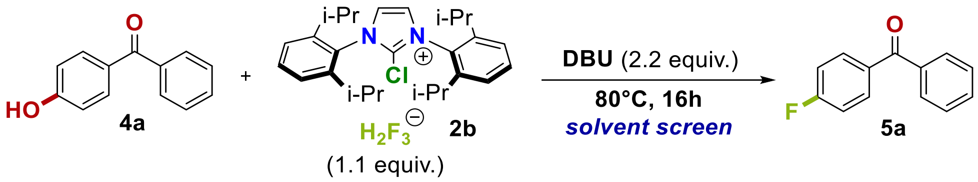

An oven-dried borosilicate vial is charged consecutively with 4-hydroxybenzophenone (**4a**) (50 mg, 0.25 mmol, 1.0 equiv.), 1,3-bis(2,6-diisopropylphenyl)-2-chloroimidazolium dihydrogen trifluoride (**2b**) (132 mg, 0.27 mmol, 1.1 equiv.) and *solvent* (2 mL). To mixture is added DBU (85 mg, 0.56 mmol, 2.2 equiv.) dropwise via syringe. Vial is then sealed, submerged in an oil bath and reaction mixture stirred at 80°C for 16 h. Once cooled down to room temperature, internal standard (2-nitrobenzotrifluoride in toluene, 0.083 M, 1.0 mL, 0.25 mmol of fluorine) is added and the reaction mixture analyzed with quantitative ^{19}F NMR spectroscopy by comparing peak integrals to determine reaction conversion to product (**5a**).

Table S4. Deoxyfluorination solvent screen.

**Entry**	**2b** [equiv.]	**Time** [h]	**T** [°C]	**Solvent**	**Base** [equiv.]	**Conversion** (**5a**) [%]
**1**	1.1	16	80	Toluene	DBU (2.2)	**>99**
**2**	1.1	16	80	DME	DBU (2.2)	**>99**
**3**	1.1	16	80	EtOAc	DBU (2.2)	**>99**
**4**	1.1	16	80	MeCN	DBU (2.2)	**>99**
**5**	1.1	16	80	2-MeTHF	DBU (2.2)	**>99**
**6**	1.1	16	80	1,4-Dioxane	DBU (2.2)	**97**
**7**	1.1	16	80	Anisole	DBU (2.2)	**>99**
**8**	1.1	16	80	Diglyme	DBU (2.2)	**>99**

S10 Determination of deoxyfluorination reaction conversions

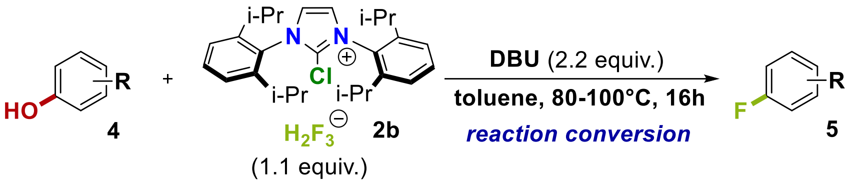

General procedure (0.25 mmol scale):

An oven-dried borosilicate vial is charged consecutively with phenol (**4**) (0.25 mmol, 1.0 equiv.), 1,3-bis(2,6-diisopropylphenyl)-2-chloroimidazolium dihydrogen trifluoride (**2b**) (132 mg, 0.27 mmol, 1.1 equiv.) and toluene (2 mL). To mixture is added DBU (85 mg, 0.56 mmol, 2.2 equiv.) dropwise via syringe. Vial is then sealed, submerged in an oil bath and reaction mixture stirred at 80-100°C for 16 h. Once cooled down to room temperature, internal standard (2-nitrobenzotrifluoride in toluene, 0.083 M, 1.0 mL, 0.25 mmol of fluorine) is added and the reaction mixture analyzed with quantitative ^{19}F NMR spectroscopy by comparing peak integrals to determine reaction conversion to product (**5**).

Table S5. Deoxyfluorination reaction conversions.

Product	Conversion [%]	Product	Conversion [%]	Product	Conversion [%]
**5a**	>99	**5j**	93	**5t**	>99
**5b**	>99	**5k**	84	**5u**	72
**5c**	95	**5l**	88	**5v**	>99
**5d**	82	**5m**	>99		
**5e**	56	**5n**	>99		
**5f**	>99	**5o**	84		
**5g**	>99	**5p**	55		
**5h**	80	**5q**	95		
**5i**	94	**5r**	92		

S11 Deoxyfluorination of other phenol substrates

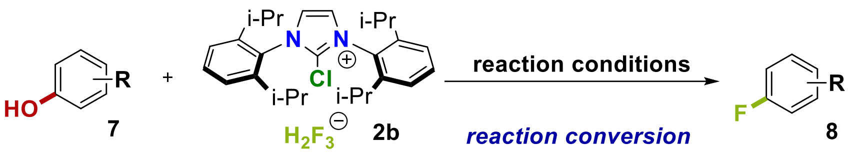

Reactions were performed following the general procedure for determination of reaction conversions (page S34) using corresponding reaction conditions (Table S6).

Table S6. Deoxyfluorination conditions and conversion of various other phenol substrates.

**Product** (**8**)	**2b** [equiv.]	**Base** [equiv.]	**Solvent**	**T** [°C]	**t** [h]	**Conversion** [%]
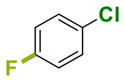 **8a**	1.2	DBU [2.4]	Toluene	85	16	**2**
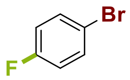 **8b**	1.2	DBU [2.4]	Toluene	85	16	**8**
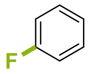 **8c**	1.3	DBU [2.6]	Toluene	110	18	**0**
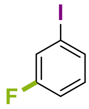 **8d**	1.2	DBU [2.4]	Toluene	85	16	**2**
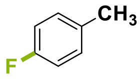 **8e**	1.3	DBU [2.6]	Toluene	110	18	**0**
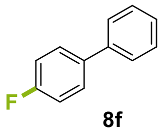 **8f**	1.7	DBU [3.5]	DME	110	48	**42**
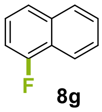 **8g**	1.7	DBU [3.5]	DME	110	24	**21**
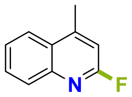 **8h**	1.3	DBU [2.6]	Toluene	110	18	**0**
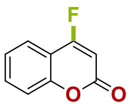 **8i**	1.3	DBU [2.6]	Toluene	110	18	**0**
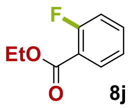 **8j**	1.2	DBU [2.4]	Toluene	85	16	**0**
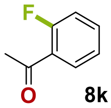 **8k**	1.2	DBU [2.4]	Toluene	85	16	**0**

S12 Deoxyfluorination anion dependance study

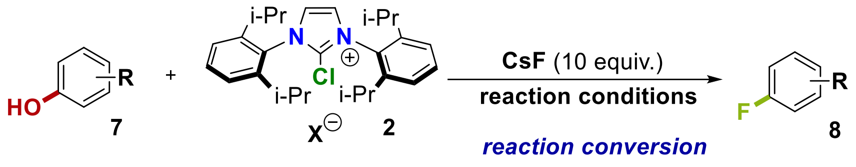

General procedure (0.25 mmol scale):

An oven-dried borosilicate vial is charged consecutively with phenol (**7**) (0.25 mmol, 1.0 equiv.), 1,3-bis(2,6-diisopropylphenyl)-2-chloroimidazolium salt (**2**) (1.5 equiv.), CsF (10 equiv.) and toluene (2 mL). Vial is then sealed, submerged in an oil bath and reaction mixture stirred at specified conditions. Once cooled down to room temperature, internal standard (2-nitrobenzotrifluoride in toluene, 0.083 M, 1.0 mL, 0.25 mmol of fluorine) is added and the reaction mixture analyzed with quantitative ^{19}F NMR spectroscopy by comparing peak integrals to determine reaction conversion to product (**8**).

Table S7. Deoxyfluorination reaction conversions with various reagents (2).

**Product** (conditions)	Conversion [%] X = $ClO_3^{\ominus}$ **2a**	X = $H_2F_3^{\ominus}$ **2b**	X = $Cl^{\ominus}$ **2c**	X = $NO_3^{\ominus}$ **2d**
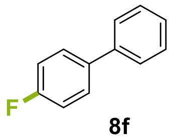 **8f** (DME, 110°C, 18h)	**0**[a]	**42**[b]	**82**[c] (99[d])	**0**[a]
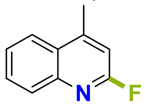 **8h** (Toluene, 110°C, 18h)	*n.d.*	**0**[b]	**71**	*n.d.*
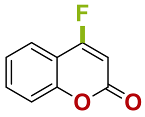 **8i** (Toluene, 110°C, 18h)	*n.d.*	**0**[b]	**41**	*n.d.*
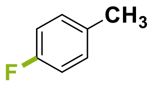 **8e** (Toluene, 110°C, 18h)	*n.d.*	**0**[b]	**66**	*n.d.*

[a]Reaction mixture turned deep dark color and afforded lots of side products. [b]Reaction was performed according to general procedure for deoxyfluorination with reagent (**2b**) (page S34). [c]Using reagent (**2c**) prepared by described procedure on page S9. [d]Reaction performed using PhenoFluorMix.

S13 Deoxyfluorination of silyl protected phenols

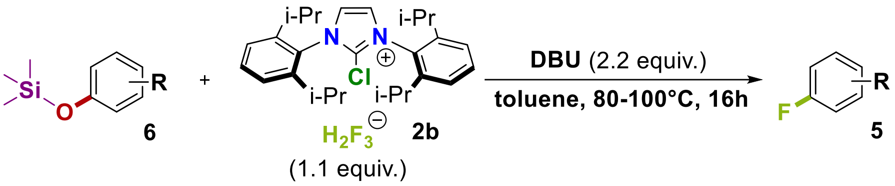

Trimethylsilyl protected phenols were synthesized according to modified literature procedure[5] (page S17).

General procedure (0.25 mmol scale):

An oven-dried borosilicate vial is charged consecutively with trimethylsilyl protected phenol (**6**) (0.25 mmol, 1.0 equiv.), 1,3-bis(2,6-diisopropylphenyl)-2-chloroimidazolium dihydrogen trifluoride (**2b**) (132 mg, 0.27 mmol, 1.1 equiv.) and toluene (2 mL). To mixture is added DBU (85 mg, 0.56 mmol, 2.2 equiv.) dropwise via syringe. Vial is then sealed, submerged in an oil bath and reaction mixture stirred at 80-100°C for 16 h. Once cooled down to room temperature, internal standard (2-nitrobenzotrifluoride in toluene, 0.083 M, 1.0 mL, 0.25 mmol of fluorine) is added and the reaction mixture analyzed with quantitative ^{19}F NMR spectroscopy by comparing peak integrals to determine reaction conversion to product (**5**).

Table S8. Deoxyfluorination reaction conversions of silyl protected phenols.

**Silyl phenol** (**6**)	**Product**	**Conversion** [%]	**Conversion from** (**4**) [%]
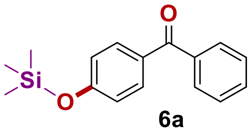 **6a**	**5a**	>99	>99
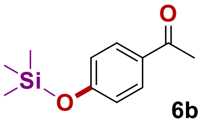 **6b**	**5b**	>99	>99
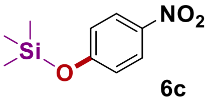 **6c**	**5c**	87	95
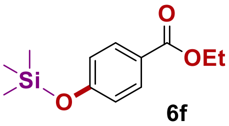 **6f**	**5f**	>99	>99

S14 Crystal Structure data

1,3-Bis(2,6-diisopropylphenyl)-2-chloroimidazolium chlorate(V) (2a)

Crystals were obtained by slow evaporation of solution of compound (**2a**) in MeCN at 25°C.

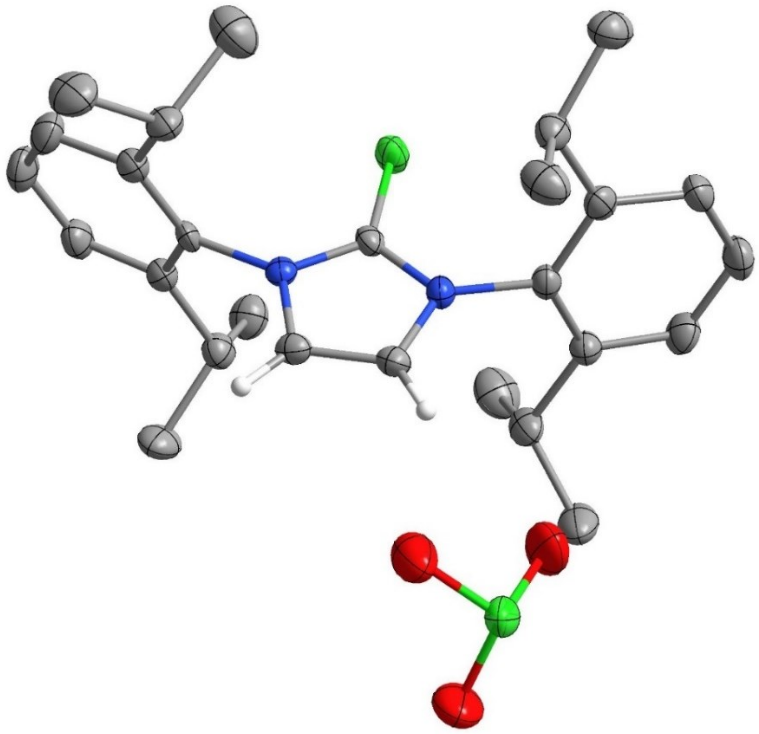

Figure S3. X-Ray crystal structure of (2a).

Table S9. Collection and structure refinement data of compound (2a).

Molecular formula	$C_{27}H_{36}ClN_2 \cdot ClO_3$
Molar mass	507.48
Crystal system / space group	Triclinic, $P\bar{1}$
a [Å]	8.4701 (2)
b [Å]	12.7454 (3)
c [Å]	14.2189 (3)
α [°]	110.978 (2)
β [°]	98.357 (2)
γ [°]	105.521 (2)
V [Å^3]	1330.46 (6)
Z	2
Density [g/cm^3]	1.267
Crystal dimension [mm]	0.42 × 0.37 × 0.16
T [K]	150
Radiation type	Cu *Kα*

λ [Å]	1.54184
μ [mm^{-1}]	2.429
F [000]	540
Θ_{max} [°]	72.3322
Θ_{min} [°]	3.4554
Index range	$-10 \leq h \leq 10$<br>$-15 \leq k \leq 15$<br>$-17 \leq l \leq 16$
No. of measured reflections	44188
No. of independent reflections	5225
No. of reflections with [I > 2σ(I)]	4642
R_{int}	0.043
Data/parameters/restrains	5225/315/0
R [$F^2 > 2\sigma(F^2)$]	0.037
$wR(F^2)$	0.099
$\Delta\rho_{max}$ [eÅ^{-3}]	0.487
$\Delta\rho_{min}$ [eÅ^{-3}]	-0.324

Table S10. Bond lengths of compound (2a).

**Cl1—C2**	1.682 (2)	**C17—H17A**	0.9800
**N1—C2**	1.330 (2)	**C17—H17B**	0.9800
**N1—C5**	1.391 (2)	**C17—H17C**	0.9800
**N1—C6**	1.459 (2)	**C18—C19**	1.396 (2)
**C2—N3**	1.334 (2)	**C18—C23**	1.406 (2)
**N3—C4**	1.388 (2)	**C19—C20**	1.395 (2)
**N3—C18**	1.4534(2)	**C19—C24**	1.522 (2)
**C4—H4**	0.9500	**C20—H20**	0.9500
**C4—C5**	1.351 (2)	**C20—C21**	1.382 (2)
**C5—H5**	0.9500	**C21—H21**	0.9500
**C6—C7**	1.397 (2)	**C21—C22**	1.386 (2)
**C6—C11**	1.403 (2)	**C22—H22**	0.9500
**C7—C8**	1.399 (2)	**C22—C23**	1.392 (2)
**C7—C12**	1.521 (2)	**C23—C27**	1.523 (2)

**C8—H8**	0.9500	**C24—H24**	1.0000
**C8—C9**	1.383 (2)	**C24—C25**	1.526 (2)
**C9—H9**	0.9500	**C24—C26**	1.528 (3)
**C9—C10**	1.385 (3)	**C25—H25A**	0.9800
**C10—H10**	0.9500	**C25—H25B**	0.9800
**C10—C11**	1.391 (2)	**C25—H25C**	0.9800
**C11—C15**	1.517 (2)	**C26—H26A**	0.9800
**C12—H12**	1.0000	**C26—H26B**	0.9800
**C12—C13**	1.529 (2)	**C26—H26C**	0.9800
**C12—C14**	1.532 (2)	**C27—H27**	1.0000
**C13—H13A**	0.9800	**C27—C28**	1.530 (2)
**C13—H13B**	0.9800	**C27—C29**	1.531 (2)
**C13—H13C**	0.9800	**C28—H28A**	0.9800
**C14—H14A**	0.9800	**C28—H28B**	0.9800
**C14—H14B**	0.9800	**C28—H28C**	0.9800
**C14—H14C**	0.9800	**C29—H29A**	0.9800
**C15—H15**	1.0000	**C29—H29B**	0.9800
**C15—C16**	1.526 (2)	**C29—H29C**	0.9800
**C15—C17**	1.525 (2)	**O1—Cl2**	1.462 (1)
**C16—H16A**	0.9800	**Cl2—O2**	1.468 (2)
**C16—H16B**	0.9800	**Cl2—O3**	1.470 (2)
**C16—H16C**	0.9800		

Table S11. Bond angles of compound (2a).

**C2—N1—C5**	107.6 (1)	**C15—C17—H17A**	109.5
**C2—N1—C6**	125.1 (1)	**C15—C17—H17B**	109.5
**C5—N1—C6**	127.1 (1)	**C15—C17—H17C**	109.5

**N1—C2—Cl1**	125.7 (1)	**H17A—C17—H17B**	109.5
**N1—C2—N3**	110.0 (1)	**H17A—C17—H17C**	109.5
**N3—C2—Cl1**	124.3 (1)	**H17B—C17—H17C**	109.5
**C2—N3—C4**	107.5 (1)	**C19—C18—N3**	117.3 (1)
**C2—N3—C18**	124.4 (1)	**C19—C18—C23**	124.0 (1)
**C4—N3—C18**	127.9 (1)	**C23—C18—N3**	118.6 (1)
**N3—C4—H4**	126.2	**C18—C19—C24**	122.8 (1)
**C5—C4—N3**	107.6 (1)	**C20—C19—C18**	116.6 (1)
**C5—C4—H4**	126.2	**C20—C19—C24**	120.5 (1)
**N1—C5—H5**	126.4	**C19—C20—H20**	119.4
**C4—C5—N1**	107.3 (1)	**C21—C20—C19**	121.2 (2)
**C4—C5—H5**	126.4	**C21—C20—H20**	119.4
**C7—C6—N1**	117.8 (1)	**C20—C21—H21**	119.7
**C7—C6—C11**	124.2 (1)	**C20—C21—C22**	120.53 (2)
**C11—C6—N1**	118.0 (1)	**C22—C21—H21**	119.7
**C6—C7—C8**	116.8 (1)	**C21—C22—H22**	119.4
**C6—C7—C12**	123.5 (1)	**C21—C22—C23**	121.2 (2)
**C8—C7—C12**	119.7 (1)	**C23—C22—H22**	119.4
**C7—C8—H8**	119.7	**C18—C23—C27**	121.6 (1)
**C9—C8—C7**	120.6 (2)	**C22—C23—C18**	116.4 (1)
**C9—C8—H8**	119.7	**C22—C23—C27**	122.0 (1)
**C8—C9—H9**	119.6	**C19—C24—H24**	107.7
**C8—C9—C10**	120.7 (2)	**C19—C24—C25**	112.6 (1)
**C10—C9—H9**	119.6	**C19—C24—C26**	109.3 (2)
**C9—C10—H10**	119.2	**C25—C24—H24**	107.7
**C9—C10—C11**	121.5 (2)	**C25—C24—C26**	111.7 (2)
**C11—C10—H10**	119.2	**C26—C24—H24**	107.7

**C6—C11—C15**	122.3 (1)	**C24—C25—H25A**	109.5
**C10—C11—C6**	116.1 (1)	**C24—C25—H25B**	109.5
**C10—C11—C15**	121.6 (4)	**C24—C25—H25C**	109.5
**C7—C12—H12**	108.2	**H25A—C25—H25B**	109.5
**C7—C12—C13**	111.6 (1)	**H25A—C25—H25C**	109.5
**C7—C12—C14**	110.1 (1)	**H25B—C25—H25C**	109.5
**C13—C12—H12**	108.2	**C24—C26—H26A**	109.5
**C13—C12—C14**	110.3 (1)	**C24—C26—H26B**	109.5
**C14—C12—H12**	108.2	**C24—C26—H26C**	109.5
**C12—C13—H13A**	109.5	**H26A—C26—H26B**	109.5
**C12—C13—H13B**	109.5	**H26A—C26—H26C**	109.5
**C12—C13—H13C**	109.5	**H26B—C26—H26C**	109.5
**H13A—C13—H13B**	109.5	**C23—C27—H27**	107.8
**H13A—C13—H13C**	109.5	**C23—C27—C28**	110.7 (1)
**H13B—C13—H13C**	109.5	**C23—C27—C29**	113.2 (1)
**C12—C14—H14A**	109.5	**C28—C27—H27**	107.8
**C12—C14—H14B**	109.5	**C28—C27—C29**	109.3 (1)
**C12—C14—H14C**	109.5	**C29—C27—H27**	107.8
**H14A—C14—H14B**	109.5	**C27—C28—H28A**	109.5
**H14A—C14—H14C**	109.5	**C27—C28—H28B**	109.5
**H14B—C14—H14C**	109.5	**C27—C28—H28C**	109.5
**C11—C15—H15**	107.6	**H28A—C28—H28B**	109.5
**C11—C15—C16**	110.5 (1)	**H28A—C28—H28C**	109.5
**C11—C15—C17**	112.8 (1)	**H28B—C28—H28C**	109.5
**C16—C15—H15**	107.6	**C27—C29—H29A**	109.5
**C17—C15—H15**	107.6	**C27—C29—H29B**	109.5
**C17—C15—C16**	110.6 (1)	**C27—C29—H29C**	109.5

**C15—C16—H16A**	109.5	**H29A—C29—H29B**	109.5
**C15—C16—H16B**	109.5	**H29A—C29—H29C**	109.5
**C15—C16—H16C**	109.5	**H29B—C29—H29C**	109.5
**H16A—C16—H16B**	109.5	**O1—Cl2—O2**	108.1 (1)
**H16A—C16—H16C**	109.5	**O1—Cl2—O3**	106.73 (9)
**H16B—C16—H16C**	109.5	**O2—Cl2—O3**	106.1 (1)

1,3-Bis(2,6-diisopropylphenyl)-2-chloroimidazolium dihydrogen trifluoride (2b)

Crystals were obtained by slow evaporation of solution of compound (**2b**) in MeCN at 25°C.

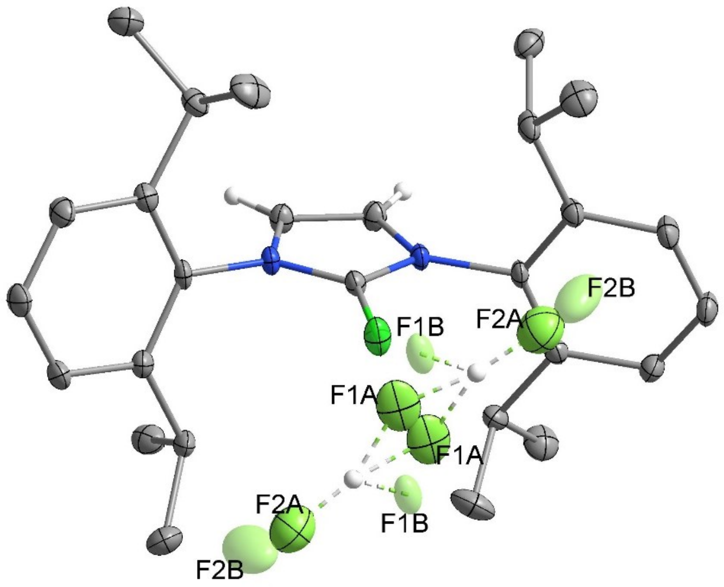

Figure S4. X-Ray crystal structure of compound (2b).

Table S12. Collection and structure refinement data of compound (2b).

Molecular formula	$C_{27}H_{38}ClF_3N_2$
Molar mass	483.04
Crystal system / space group	Monoclinic, $C2/c$
a [Å]	16.4998 (4)
b [Å]	9.4681 (2)
c [Å]	17.0687 (4)
α [°]	90
β [°]	90.681 (2)
γ [°]	90
V [Å^3]	2666.3 (1)

Z	4
Density [g/cm^3]	1.203
Crystal dimension [mm]	0.35 × 0.29 × 0.19
T [K]	100
Radiation type	Mo *K*α
λ [Å]	0.71073
μ [mm^{-1}]	0.181
F [000]	1032
Θ_{max} [°]	28.935
Θ_{min} [°]	2.469
Index range	-22 ≤ h ≤ 20<br>-11 ≤ k ≤ 11<br>-21 ≤ l ≤ 23
No. of measured reflections	30102
No. of independent reflections	3179
No. of reflections with [I > 2σ(I)]	2911
R_{int}	0.046
Data/parameters/restrains	3179/179/0
R [F^2 > 2σ(F^2)]	0.042
$wR(F^2)$	0.124
$\Delta\rho_{max}$ [eÅ^{-3}]	0.572
$\Delta\rho_{min}$ [eÅ^{-3}]	-0.606

Table S13. Bond lengths of compound (2b)

**Cl1—C1**	1.670 (2)	**C6—H4**	0.9500
**N1—C1**	1.334 (2)	**C9—H6**	1.0000
**N1—C3**	1.451 (2)	**C9—C11**	1.533 (2)
**N1—C2**	1.384 (2)	**C9—C10**	1.529 (2)
**F2A—F2B**	0.69 (2)	**C11—H12**	0.9800
**F2A—F1B**	1.72 (1)	**C11—H13**	0.9800
**F2A—H1**	1.109 (9)	**C11—H11**	0.9800
**C4—C3**	1.398 (2)	**C13—H18**	0.9800
**C4—C5**	1.398 (2)	**C13—H17**	0.9800
**C4—C12**	1.519 (2)	**C13—H19**	0.9800

**C3—C8**	1.400 (2)	**C10—H8**	0.9800
**C2—C2^{i}**	1.353 (3)	**C10—H10**	0.9800
**C2—H2**	0.9500	**C10—H9**	0.9800
**C5—H3**	0.9500	**C14—H14**	0.9800
**C5—C6**	1.386 (2)	**C14—H16**	0.9800
**C8—C7**	1.393 (2)	**C14—H15**	0.9800
**C8—C9**	1.523 (2)	**F1B—F1A**	0.881 (9)
**C7—H5**	0.9500	**F1B—F1A^{i}**	1.401 (1)
**C7—C6**	1.389 (2)	**F1B—H1**	0.870 (6)
**C12—H7**	1.0000	**F1A—F1A^{i}**	0.719 (9)
**C12—C13**	1.527 (2)	**F1A—H1**	1.199 (7)
**C12—C14**	1.528 (2)		

Table S14. Bond angles of compound (2b).

**C1—N1—C3**	124.7 (1)	**C11—C9—H6**	107.8
**C1—N1—C2**	108.3 (1)	**C10—C9—H6**	107.8
**C2—N1—C3**	126.9 (1)	**C10—C9—C11**	110.1 (1)
**F2B—F2A—F1B**	150.9 (8)	**C9—C11—H12**	109.5
**F2B—F2A—H1**	160 (1)	**C9—C11—H13**	109.5
**F1B—F2A—H1**	25.9 (3)	**C9—C11—H11**	109.5
**N1—C1—Cl1**	125.48 (8)	**H12—C11—H13**	109.5
**N1^{i}—C1—Cl1**	125.48 (8)	**H12—C11—H11**	109.5
**N1—C1—N1^{i}**	109.0 (2)	**H13—C11—H11**	109.5
**C3—C4—C5**	116.8 (1)	**C12—C13—H18**	109.5
**C3—C4—C12**	122.4 (1)	**C12—C13—H17**	109.5
**C5—C4—C12**	120.7 (1)	**C12—C13—H19**	109.5
**C4—C3—N1**	117.5 (1)	**H18—C13—H17**	109.5

**C4—C3—C8**	124.4 (11)	**H18—C13—H19**	109.5
**C8—C3—N1**	118.1 (1)	**H17—C13—H19**	109.5
**N1—C2—H2**	126.4	**C9—C10—H8**	109.5
**C2^{i}—C2—N1**	107.21 (7)	**C9—C10—H10**	109.5
**C2^{i}—C2—H2**	126.4	**C9—C10—H9**	109.5
**C4—C5—H3**	119.7	**H8—C10—H10**	109.5
**C6—C5—C4**	120.5 (1)	**H8—C10—H9**	109.5
**C6—C5—H3**	119.7	**H10—C10—H9**	109.5
**C3—C8—C9**	121.0 (1)	**C12—C14—H14**	109.5
**C7—C8—C3**	116.3 (1)	**C12—C14—H16**	109.5
**C7—C8—C9**	121.7 (1)	**C12—C14—H15**	109.5
**C8—C7—H5**	119.4	**H14—C14—H16**	109.5
**C6—C7—C8**	121.2 (1)	**H14—C14—H15**	109.5
**C6—C7—H5**	119.4	**H16—C14—H15**	109.5
**C4—C12—H7**	107.9	**F2A—F1B—H1**	33.8 (4)
**C4—C12—C13**	111.8 (1)	**F1A—F1B—F2A**	120.3 (7)
**C4—C12—C14**	110.1 (1)	**F1A^{i}—F1B—F2A**	94.9 (4)
**C13—C12—H7**	107.9	**F1A—F1B—F1A^{i}**	25.4 (6)
**C13—C12—C14**	111.1 (1)	**F1A—F1B—H1**	86.4 (8)
**C14—C12—H7**	107.9	**F1A^{i}—F1B—H1**	61.0 (5)
**C5—C6—C7**	120.8 (1)	**F1B—F1A—F1B^{i}**	154.0 (6)
**C5—C6—H4**	119.6	**F1B—F1A—H1**	46.4 (6)
**C7—C6—H4**	119.6	**F1B^{i}—F1A—H1**	107.8 (3)
**C8—C9—H6**	107.8	**F1A^{i}—F1A—F1B**	122.9 (16)
**C8—C9—C11**	110.2 (1)	**F1A^{i}—F1A—F1B^{i}**	31.7 (10)
**C8—C9—C10**	112.9 (1)	**F1A^{i}—F1A—H1**	76.5 (11)

1,3-Bis(2,6-diisopropylphenyl)-2-imidazolone (3)

Crystals were obtained by slow evaporation of solution of compound (**3**) in EtOAc at 25°C.

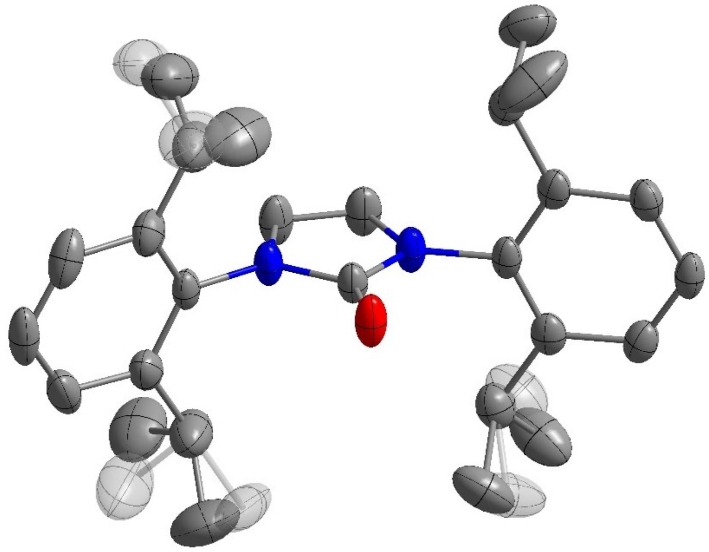

Figure S5. X-Ray crystal structure of (3).

Molecular formula	$C_{27}H_{36}N_2O$
Molar mass	404.58
Crystal system / space group	Monoclinic, $P2_1/n$
a [Å]	19.3974 (2)
b [Å]	6.5240 (4)
c [Å]	20.6105 (4)
α [°]	90
β [°]	106.612 (1)
γ [°]	90
V [Å^3]	2499.37 (5)
Z	4
Density [g/cm^3]	1.075
Crystal dimension [mm]	0.79 × 0.6 × 0.38
T [K]	170
Radiation type	Cu *K*α
λ [Å]	1.54184
μ [mm^{-1}]	0.50
F [000]	880
Θ_{max} [°]	72.360
Θ_{min} [°]	2.760
Index range	-22 ≤ h ≤ 23<br>-8 ≤ k ≤ 7<br>-25 ≤ l ≤ 25
No. of measured reflections	46873

No. of independent reflections	4891
No. of reflections with [I > 2σ(I)]	4582
R_{int}	0.027
Data/parameters/restrains	4891/331/0
R [F^2 > 2σ(F^2)]	0.047
$wR(F^2)$	0.130
$\Delta\rho_{max}$ [eÅ^{-3}]	0.292
$\Delta\rho_{min}$ [eÅ^{-3}]	-0.270

Methyl 2-(6-fluoro-3-oxoxanthen-9-yl)benzoate (5w)

Crystals were obtained by slow evaporation of solution of compound (**5w**) in EtOAc at 25°C.

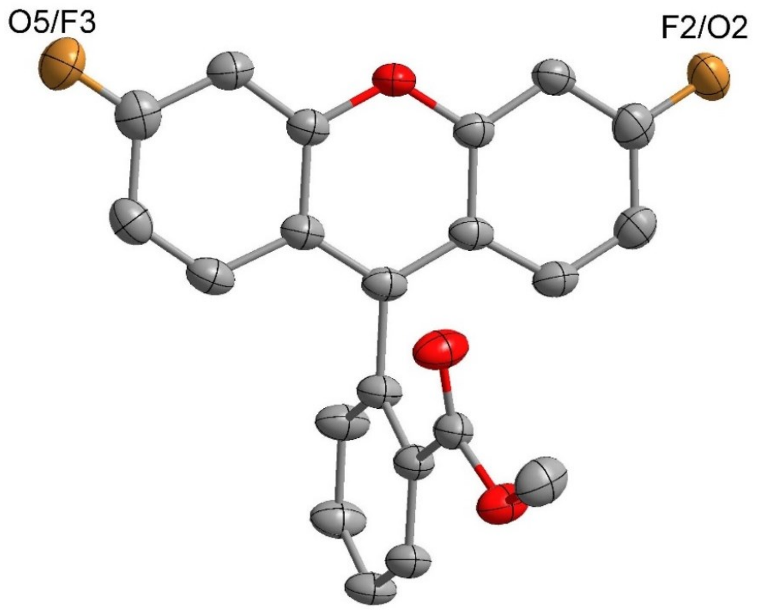

Figure S6. X-Ray crystal structure of (5w).

Table S15. Collection and structure refinement data of (5w).

Molecular formula	$C_{21}H_{13}O_4F$
Molar mass	348.31
Crystal system / space group	Triclinic, $P\bar{1}$
a [Å]	7.6883 (3)
b [Å]	10.6329 (4)
c [Å]	11.01080 (4)
α [°]	106.800 (3)
β [°]	100.781 (3)
γ [°]	105.018 (3)
V [Å^3]	805.22 (6)

Z	2
Density [g/cm^3]	1.437
Crystal dimension [mm]	0.83 × 0.59 × 0.49
T [K]	150
Radiation type	Cu $K\alpha$
λ [Å]	1.54184
μ [mm^{-1}]	0.897
F [000]	360
Θ_{max} [°]	72.3070
Θ_{min} [°]	4.2900
Index range	-9 ≤ h ≤ 9<br>-12 ≤ k ≤ 11<br>-13 ≤ l ≤ 13
No. of measured reflections	27779
No. of independent reflections	3122
No. of reflections with [I > 2σ(I)]	2587
R_{int}	0.049
Data/parameters/restrains	3122/231/0
R [F^2 > 2σ(F^2)]	0.040
wR(F^2)	0.115
$\Delta\rho_{max}$ [eÅ^{-3}]	0.230
$\Delta\rho_{min}$ [eÅ^{-3}]	-0.190

Table S16. Bond lengths of compound (5w)

**O1—C13**	1.372 (2)	**C9—H9**	0.9500
**O1—C1**	1.372 (2)	**C9—C10**	1.369 (2)
**O4—C20**	1.332 (2)	**C12—H12**	0.9500
**O4—C21**	1.453 (2)	**C12—C11**	1.395 (2)
**O3—C20**	1.200 (2)	**C5—H5**	0.9500
**C20—C19**	1.498 (2)	**C5—C4**	1.358 (2)
**C8—C7**	1.419 (2)	**C11—C10**	1.407 (2)
**C8—C13**	1.417 (2)	**C11—F2**	1.322 (2)
**C8—C9**	1.416 (2)	**C11—O2**	1.322 (2)
**C7—C6**	1.392 (2)	**C17—H17**	0.9500

**C7—C14**	1.495 (2)	**C17—C16**	1.381 (2)
**C13—C12**	1.372 (2)	**C16—H16**	0.9500
**C1—C6**	1.436 (2)	**C16—C15**	1.387 (2)
**C1—C2**	1.360 (2)	**C15—H15**	0.9500
**C6—C5**	1.428 (2)	**C10—H10**	0.9500
**C14—C19**	1.403 (2)	**C3—C4**	1.447 (2)
**C14—C15**	1.398 (2)	**C3—O5**	1.277 (2)
**C2—H2**	0.9500	**C3—F3**	1.277 (2)
**C2—C3**	1.421 (2)	**C4—H4**	0.9500
**C18—H18**	0.9500	**C21—H21A**	0.9800
**C18—C19**	1.393 (2)	**C21—H21B**	0.9800
**C18—C17**	1.386 (2)	**C21—H21C**	0.9800

Table S17. Bond angles of compound (5w)

**C1—O1—C13**	120.7 (1)	**C18—C19—C20**	120.5 (1)
**C20—O4—C21**	116.0 (1)	**C18—C19—C14**	120.0 (1)
**O4—C20—C19**	112.3 (1)	**C6—C5—H5**	119.1
**O3—C20—O4**	123.5 (1)	**C4—C5—C6**	121.8 (1)
**O3—C20—C19**	124.2 (1)	**C4—C5—H5**	119.1
**C13—C8—C7**	119.3 (1)	**C12—C11—C10**	122.0 (1)
**C9—C8—C7**	123.6 (1)	**F2—C11—C12**	119.2 (2)
**C9—C8—C13**	117.1 (1)	**F2—C11—C10**	118.8 (1)
**C8—C7—C14**	119.5 (1)	**O2—C11—C12**	119.2 (2)
**C6—C7—C8**	119.4 (1)	**O2—C11—C10**	118.8 (2)
**C6—C7—C14**	120.9 (1)	**C18—C17—H17**	120.2
**O1—C13—C8**	120.8 (1)	**C16—C17—C18**	119.7 (1)
**C12—C13—O1**	116.7 (1)	**C16—C17—H17**	120.2

**C12—C13—C8**	122.5 (1)	**C17—C16—H16**	119.8
**O1—C1—C6**	120.2 (1)	**C17—C16—C15**	120.4 (1)
**C2—C1—O1**	116.9 (1)	**C15—C16—H16**	119.8
**C2—C1—C6**	122.9 (1)	**C14—C15—H15**	119.6
**C7—C6—C1**	119.6 (1)	**C16—C15—C14**	120.8 (1)
**C7—C6—C5**	123.9 (1)	**C16—C15—H15**	119.6
**C5—C6—C1**	116.5 (1)	**C9—C10—C11**	118.6 (1)
**C19—C14—C7**	123.8 (1)	**C9—C10—H10**	120.7
**C15—C14—C7**	117.6 (1)	**C11—C10—H10**	120.7
**C15—C14—C19**	118.6 (1)	**C2—C3—C4**	118.2 (1)
**C1—C2—H2**	120.0	**O5—C3—C2**	121.6 (2)
**C1—C2—C3**	119.9 (1)	**O5—C3—C4**	120.1 (2)
**C3—C2—H2**	120.0	**F3—C3—C2**	121.6 (2)
**C19—C18—H18**	119.7	**F3—C3—C4**	120.1 (2)
**C17—C18—H18**	119.7	**C5—C4—C3**	120.7 (2)
**C17—C18—C19**	120.6 (1)	**C5—C4—H4**	119.7
**C8—C9—H9**	119.1	**C3—C4—H4**	119.7
**C10—C9—C8**	121.8 (1)	**O4—C21—H21A**	109.5
**C10—C9—H9**	119.1	**O4—C21—H21B**	109.5
**C13—C12—H12**	121.0	**O4—C21—H21C**	109.5
**C13—C12—C11**	117.9 (1)	**H21A—C21—H21B**	109.5
**C11—C12—H12**	121.0	**H21A—C21—H21C**	109.5
**C14—C19—C20**	119.4 (1)	**H21B—C21—H21C**	109.5

S15 Raman Spectroscopic data

Raman spectra of 1,3-bis(2,6-diisopropylphenyl)imidazolium chlorate(V) (1b)

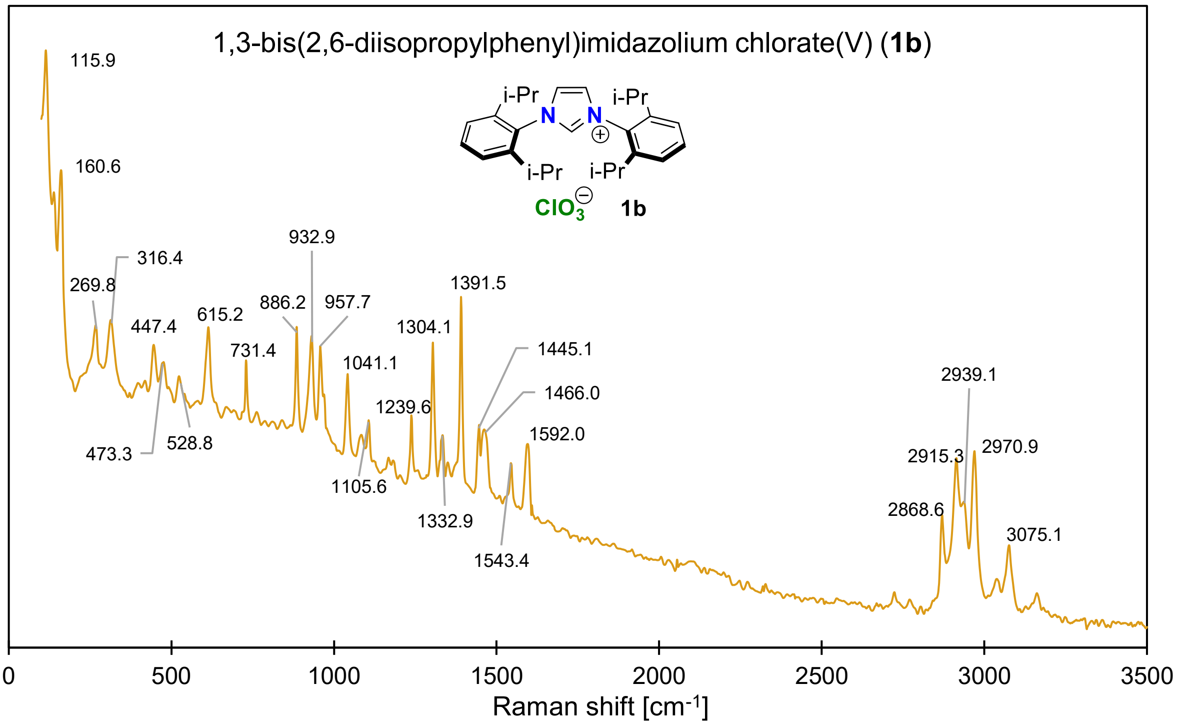

Raman spectra comparison between (1b) and sodium chlorate(V)

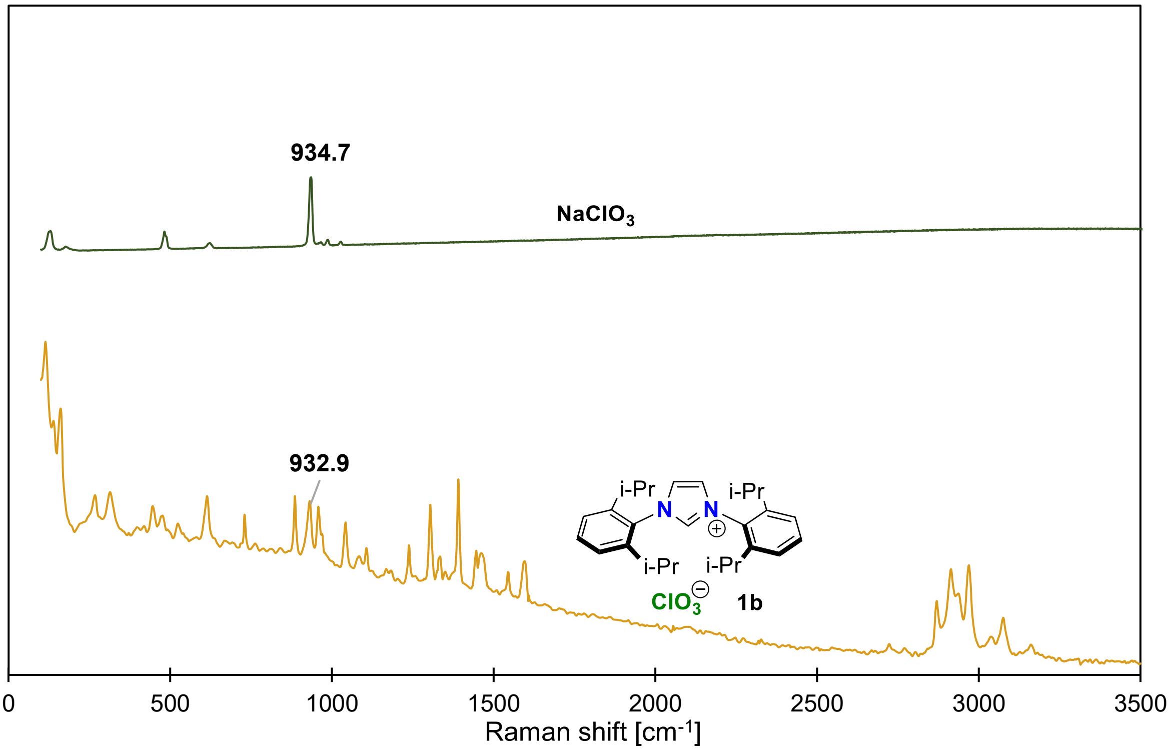

Raman spectra of 1,3-bis(2,6-diisopropylphenyl)-2-chloroimidazolium chlorate(V) (2a)

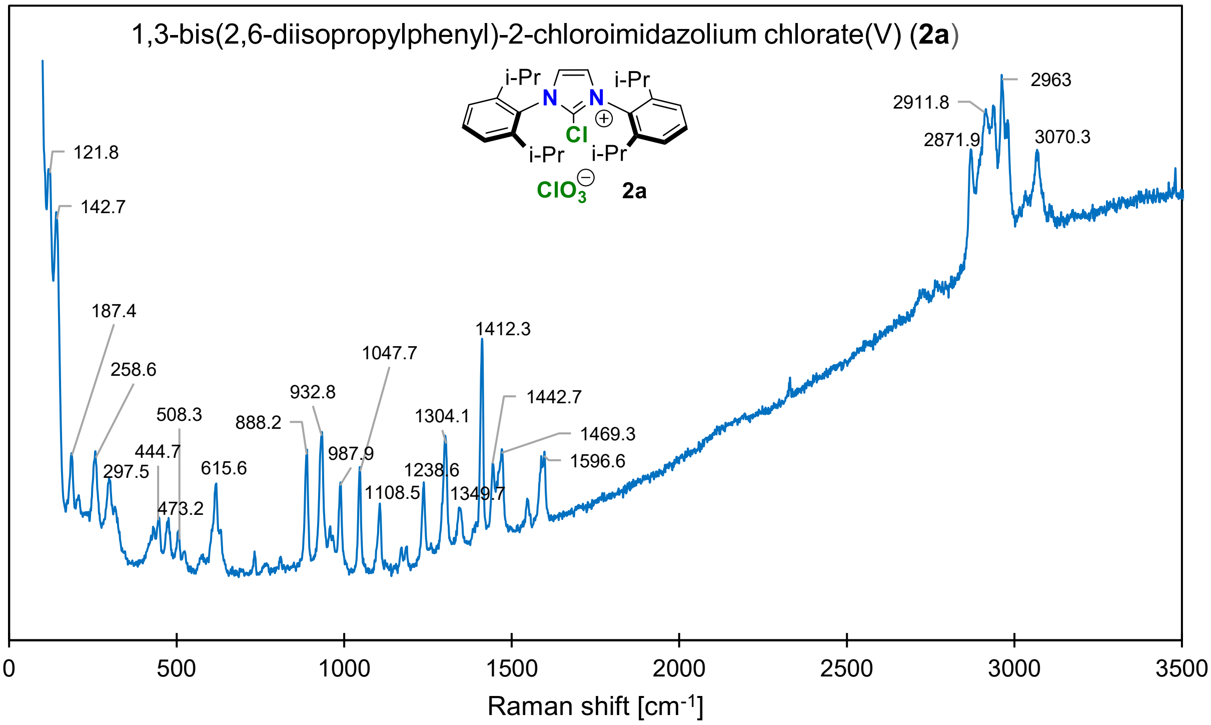

Raman spectra comparison between (2a) and sodium chlorate(V)

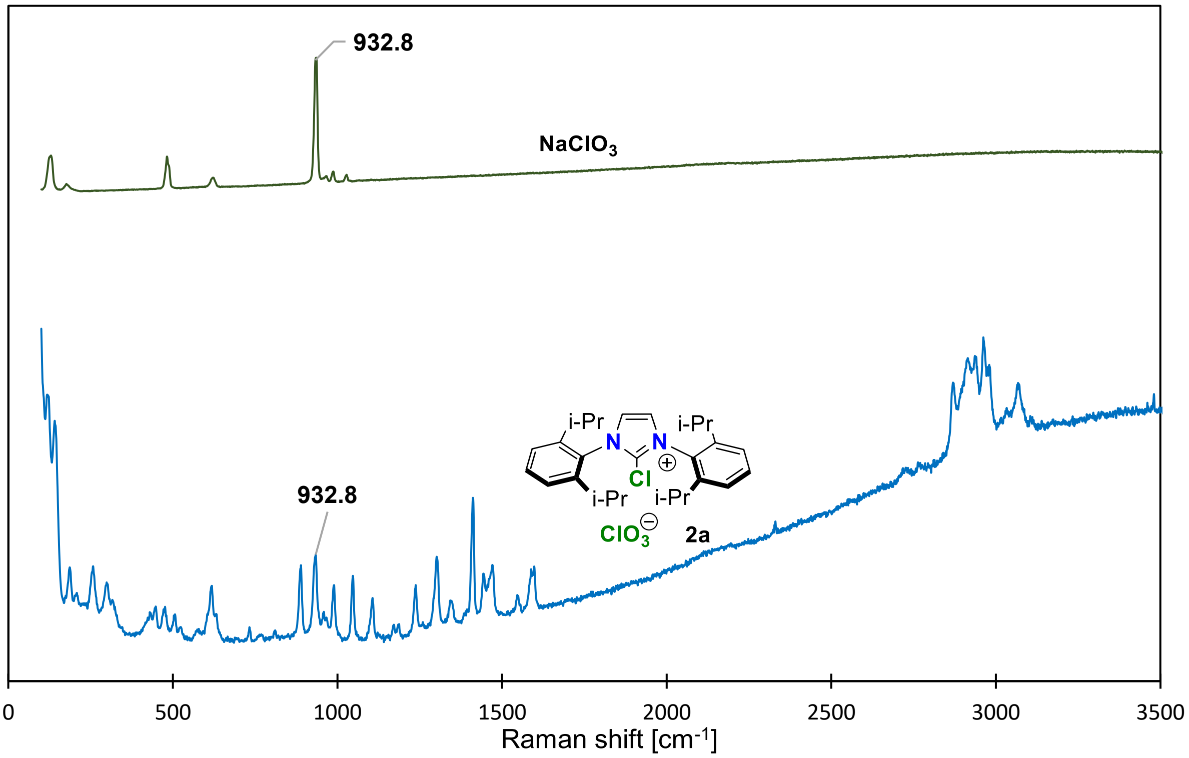

Raman spectra of 1,3-bis(2,6-diisopropylphenyl)-2-chloroimidazolium dihydrogen trifluoride (2b)

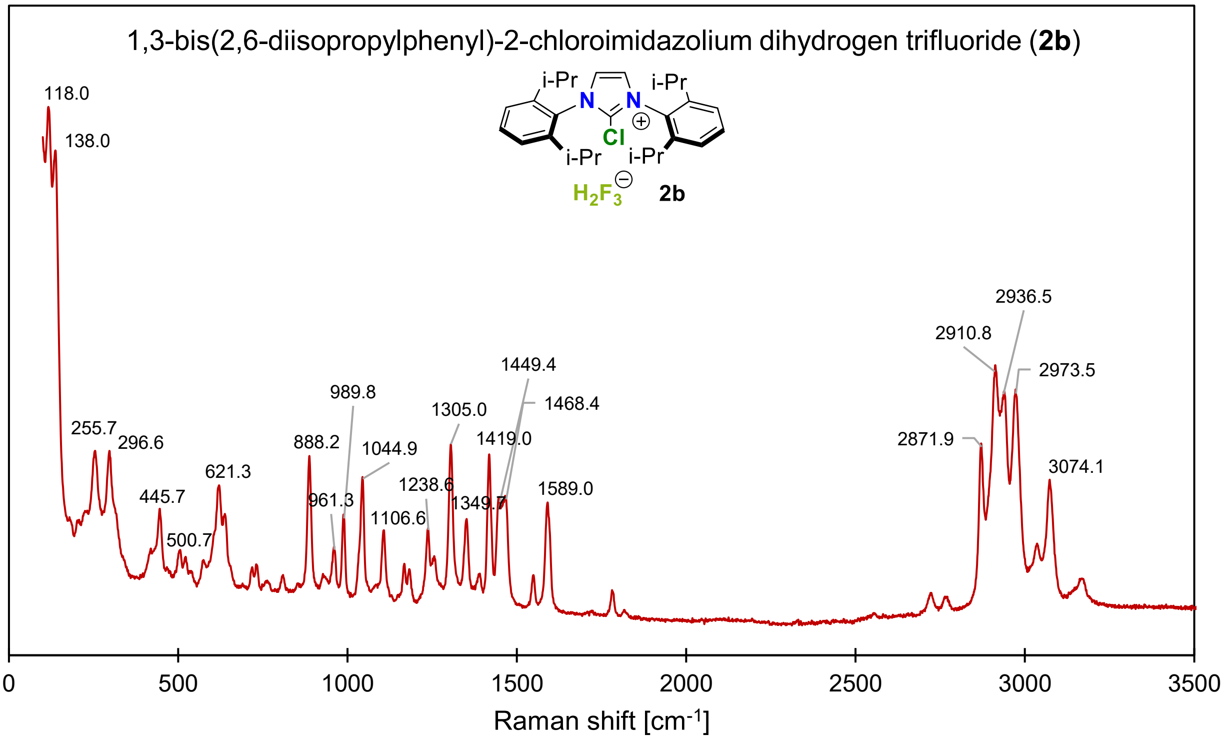

Raman spectra of 1,3-bis(2,6-diisopropylphenyl)-2-chloroimidazolium chloride (2c)

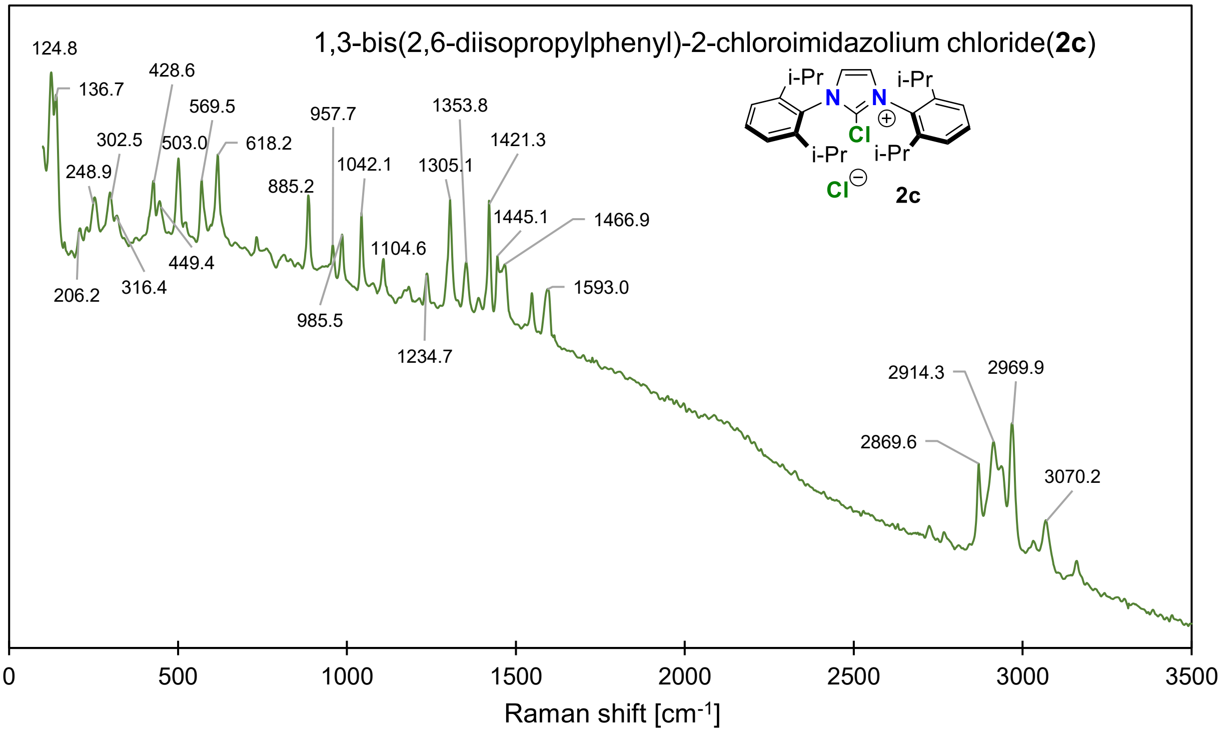

S16 NMR Spectroscopic data

^{1}H NMR of 1,3-Bis(2,6-diisopropylphenyl)-2H-imidazolium chlorate(V) (1b)

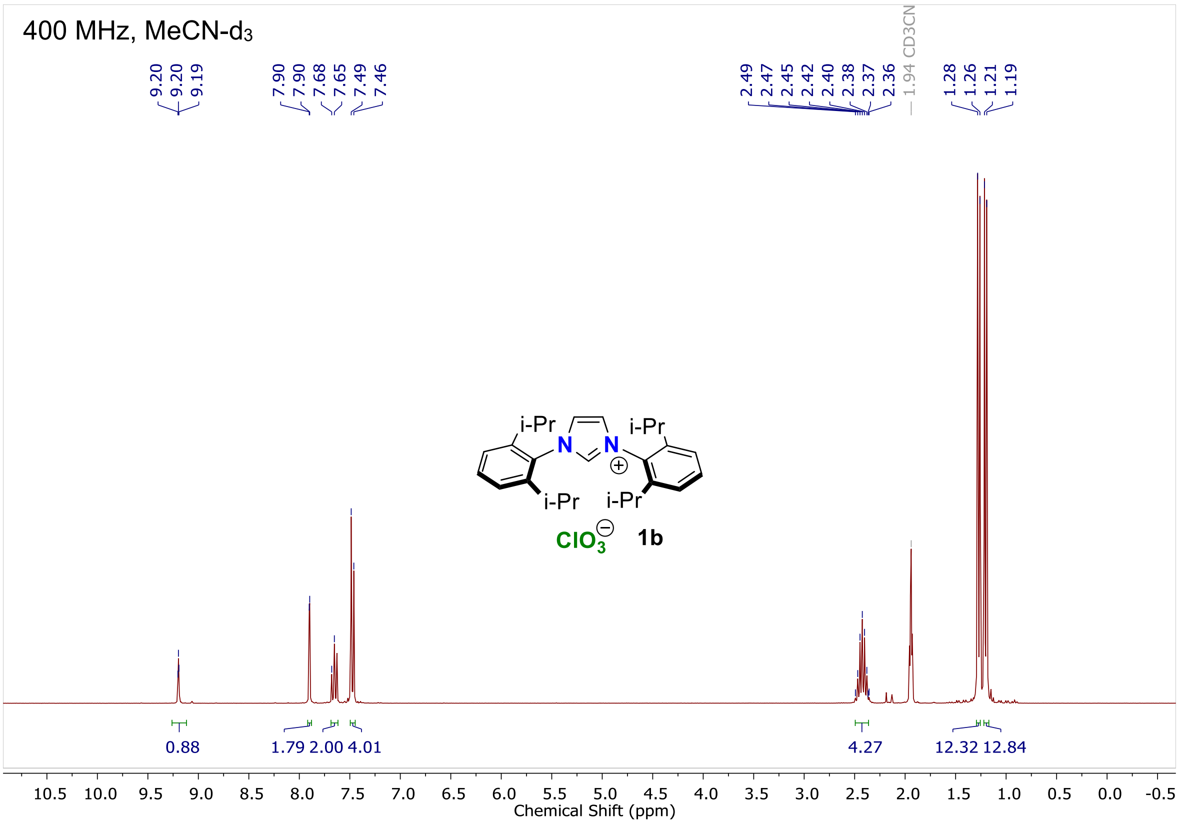

^{13}C NMR of 1,3-Bis(2,6-diisopropylphenyl)-2H-imidazolium chlorate(V) (1b)

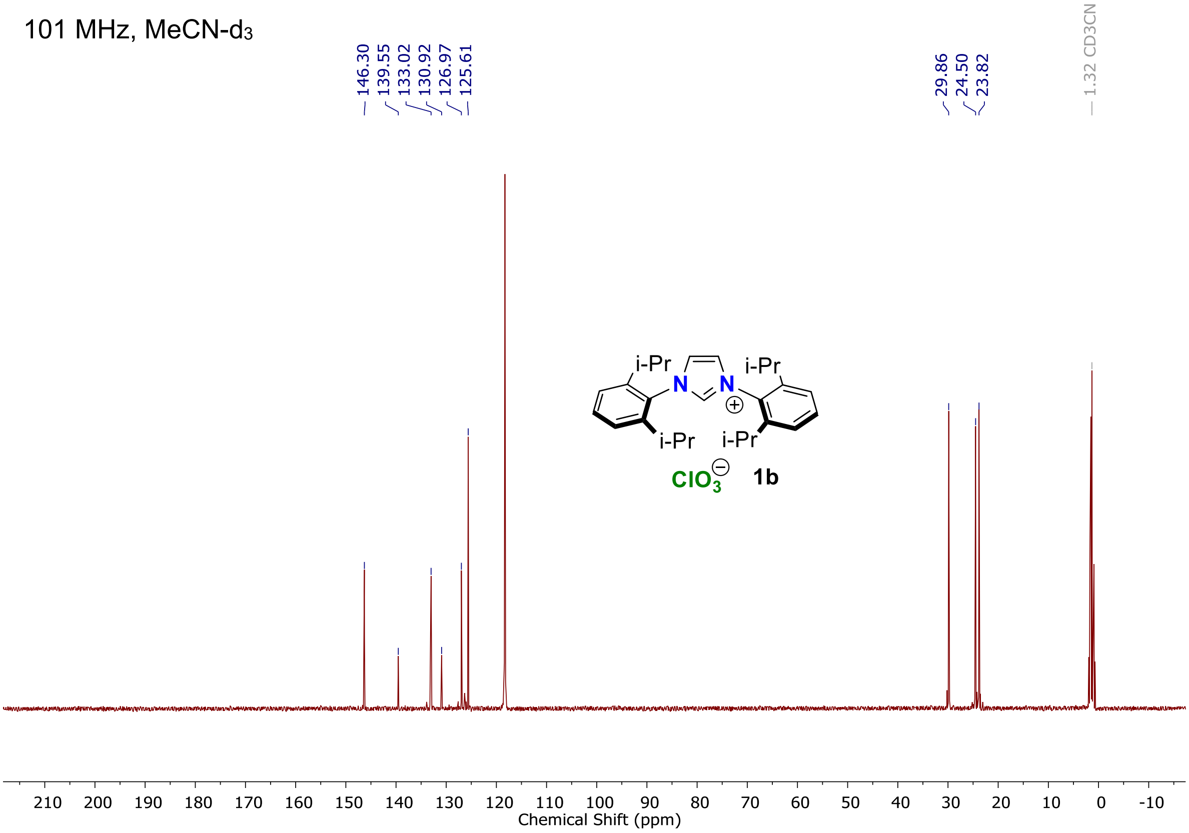

^{1}H NMR of 1,3-Bis(2,6-diisopropylphenyl)-2-chloroimidazolium chlorate(V) (2a)

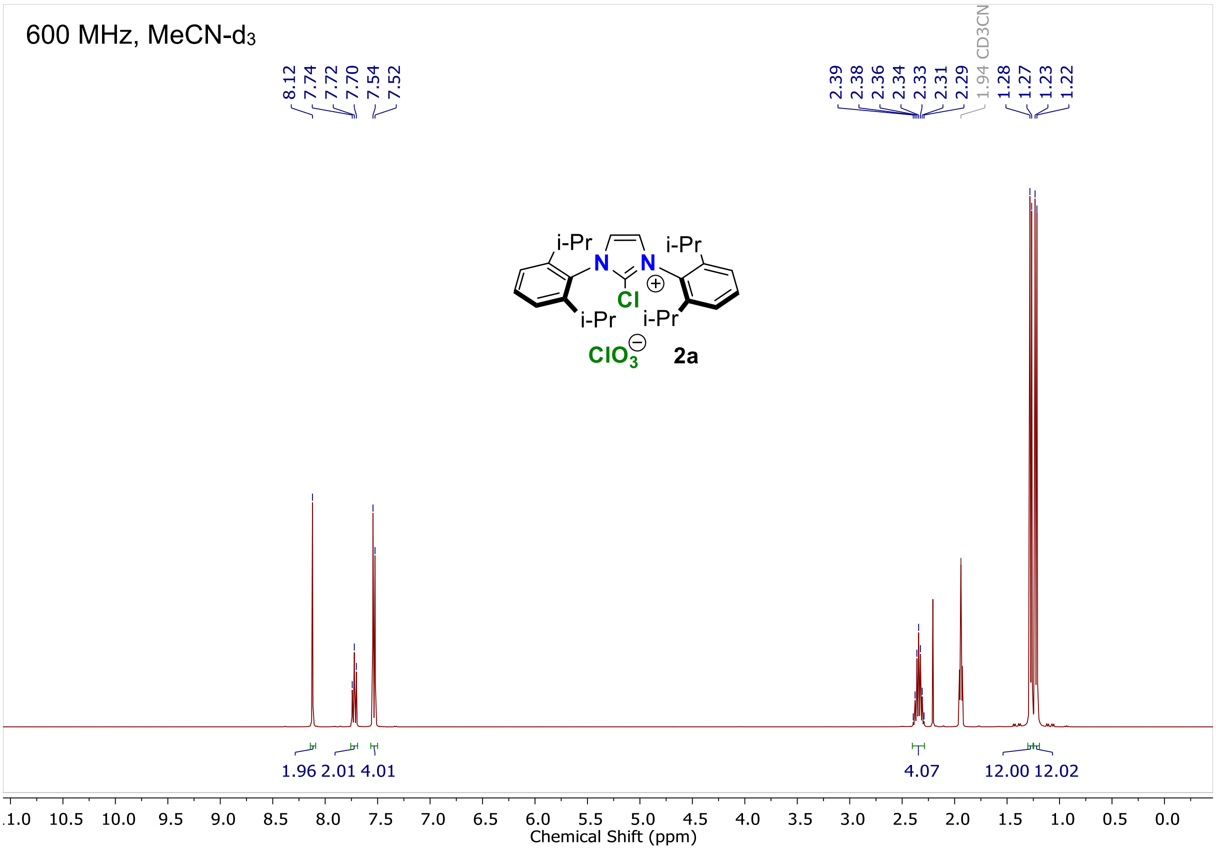

^{13}C NMR of 1,3-Bis(2,6-diisopropylphenyl)-2-chloroimidazolium chlorate(V) (2a)

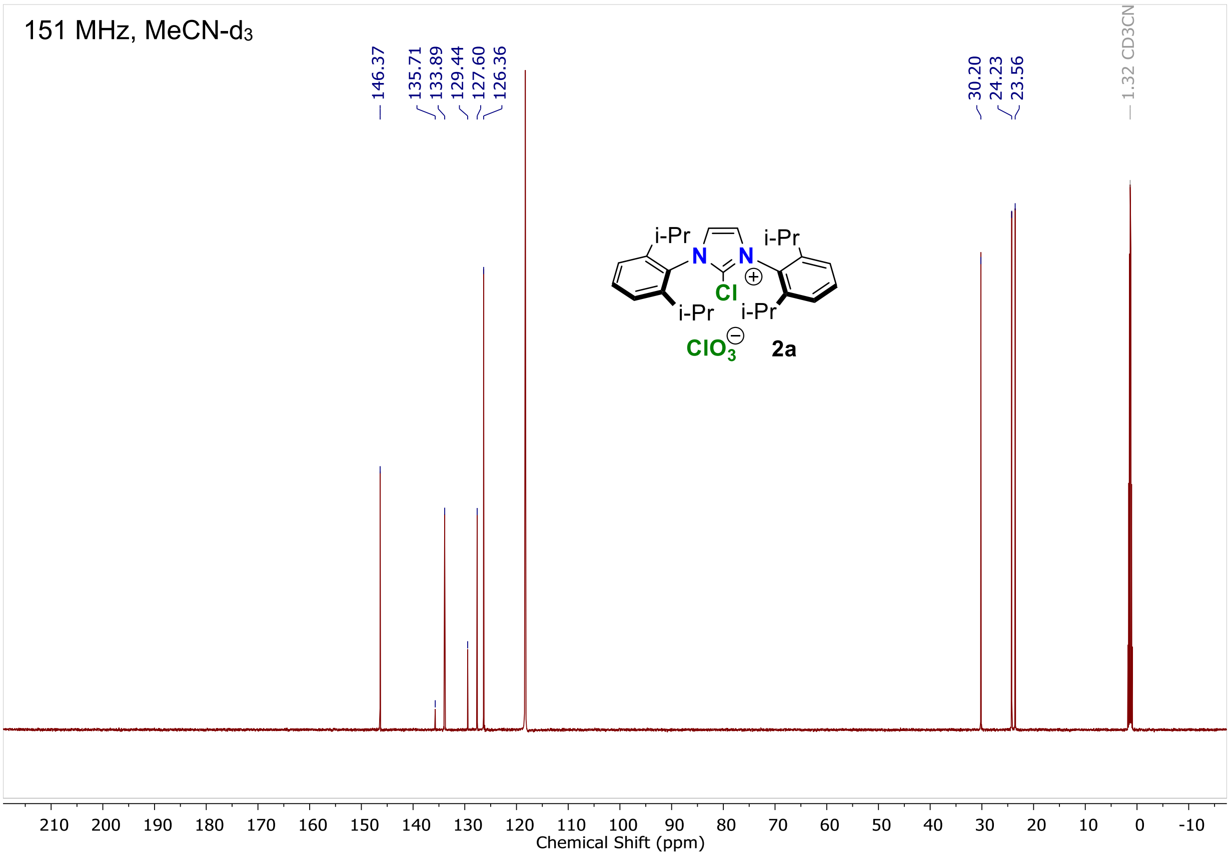

^{1}H NMR of 1,3-Bis(2,6-diisopropylphenyl)-2-chloroimidazolium dihydrogen trifluoride (2b)

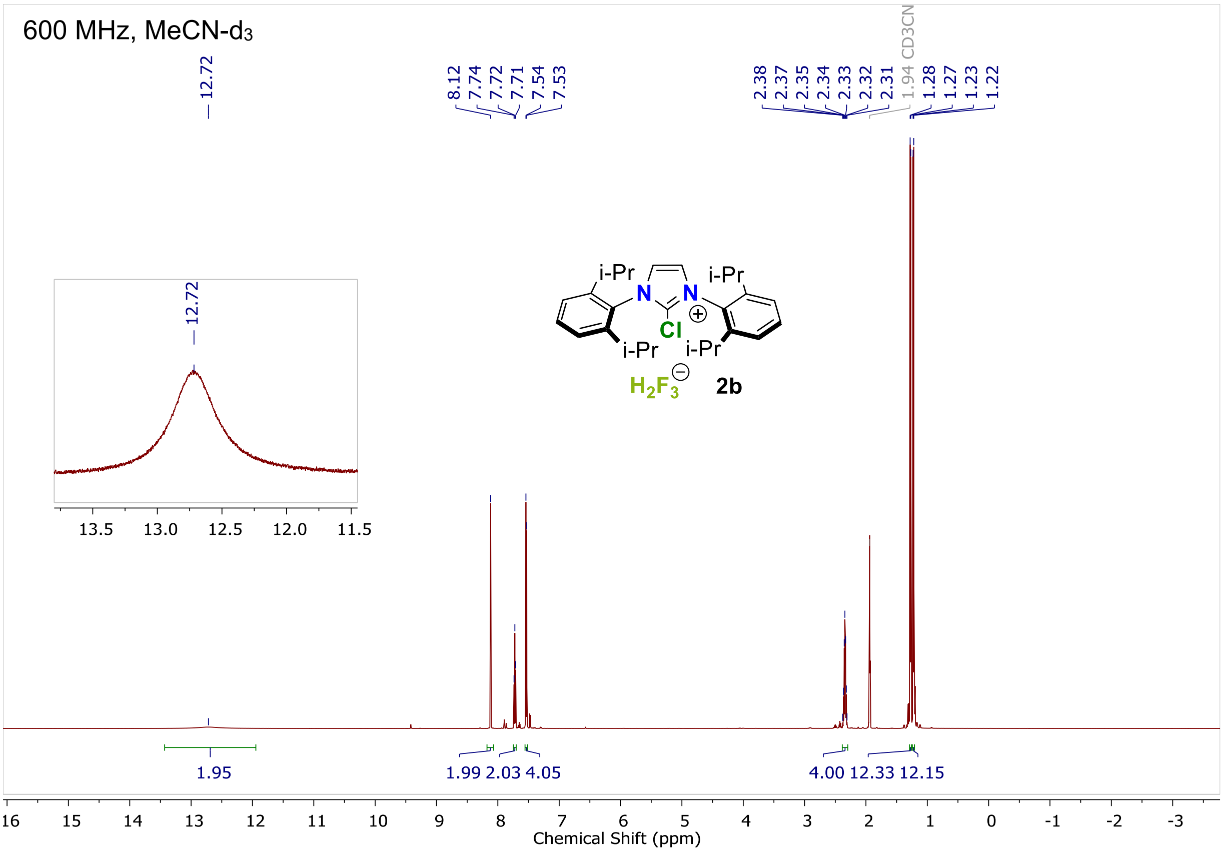

^{13}C NMR of 1,3-Bis(2,6-diisopropylphenyl)-2-chloroimidazolium dihydrogen trifluoride (2b)

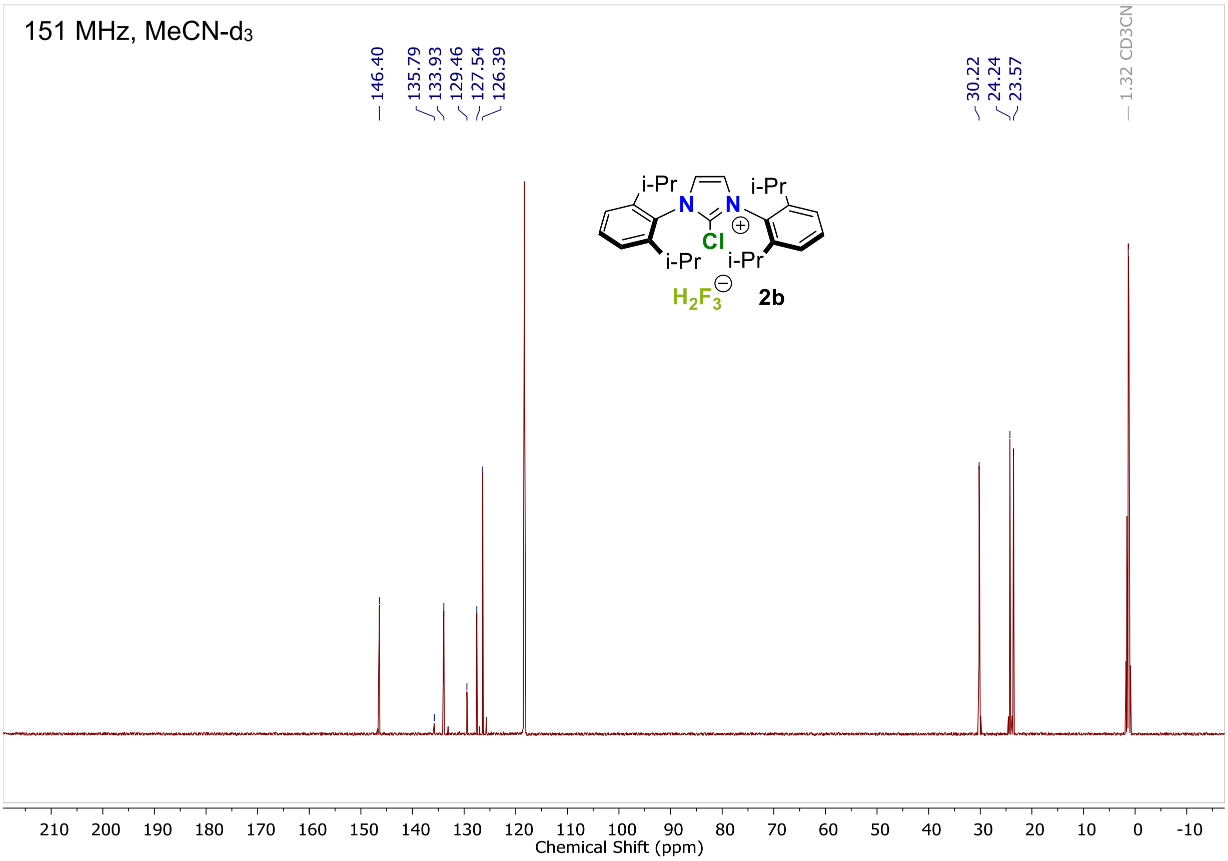

^{19}F NMR of 1,3-Bis(2,6-diisopropylphenyl)-2-chloroimidazolium dihydrogen trifluoride (2b)

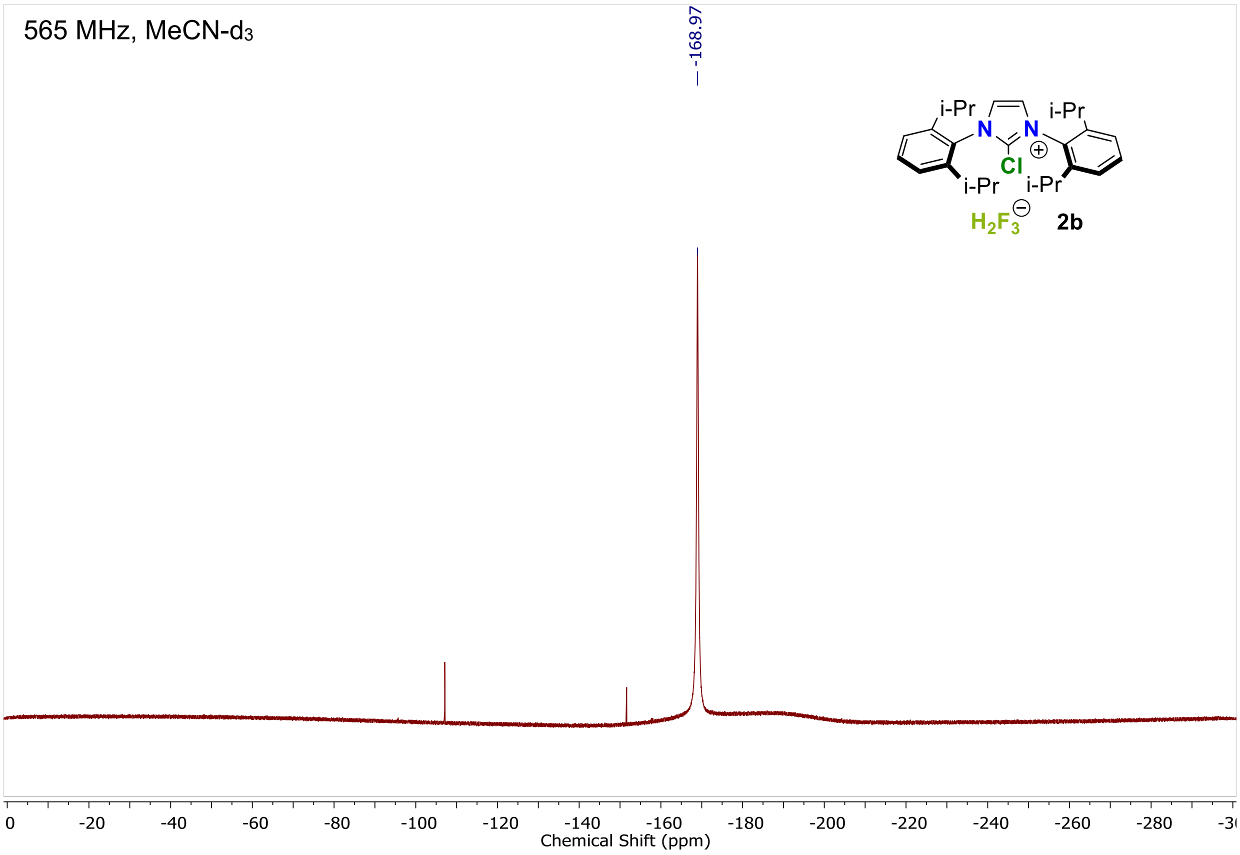

^{1}H NMR of 1,3-Bis(2,6-diisopropylphenyl)-2-chloroimidazolium chloride (2c)

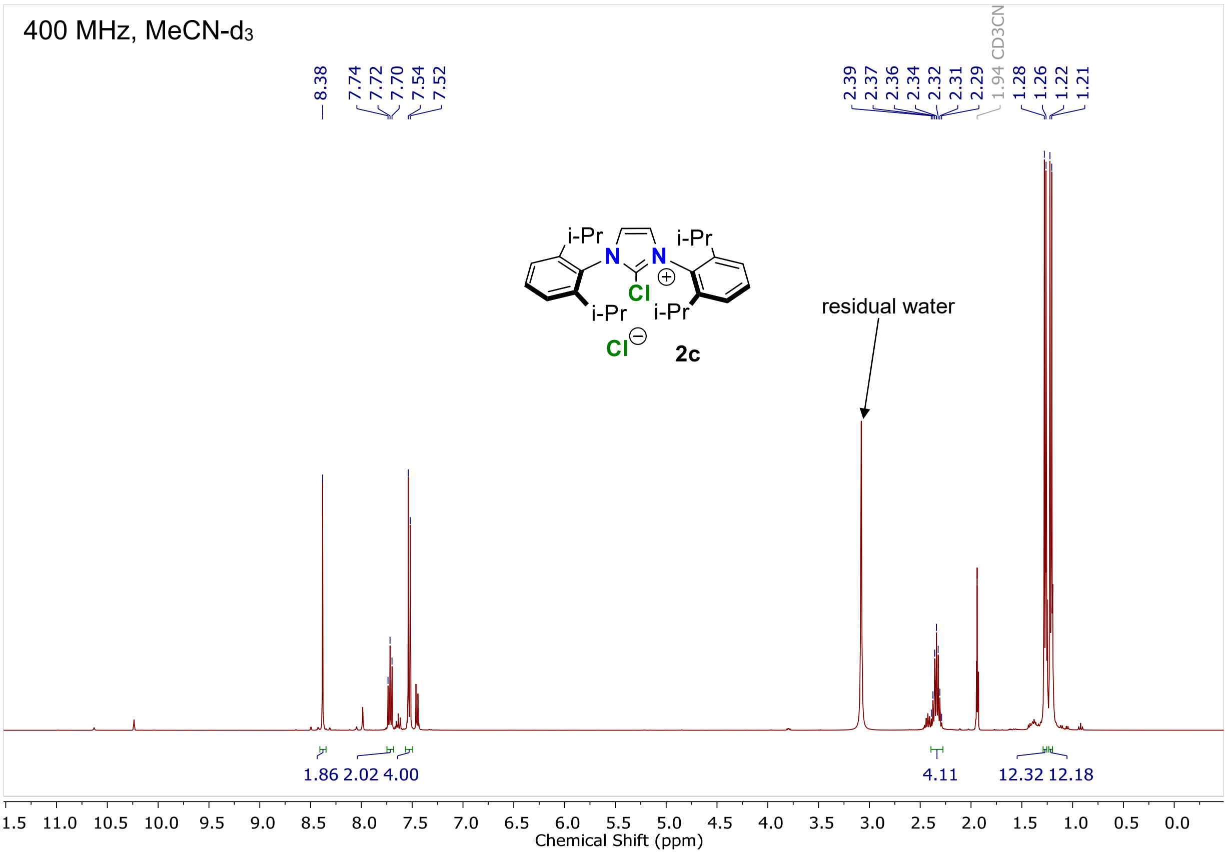

^{13}C NMR of 1,3-Bis(2,6-diisopropylphenyl)-2-chloroimidazolium chloride (2c)

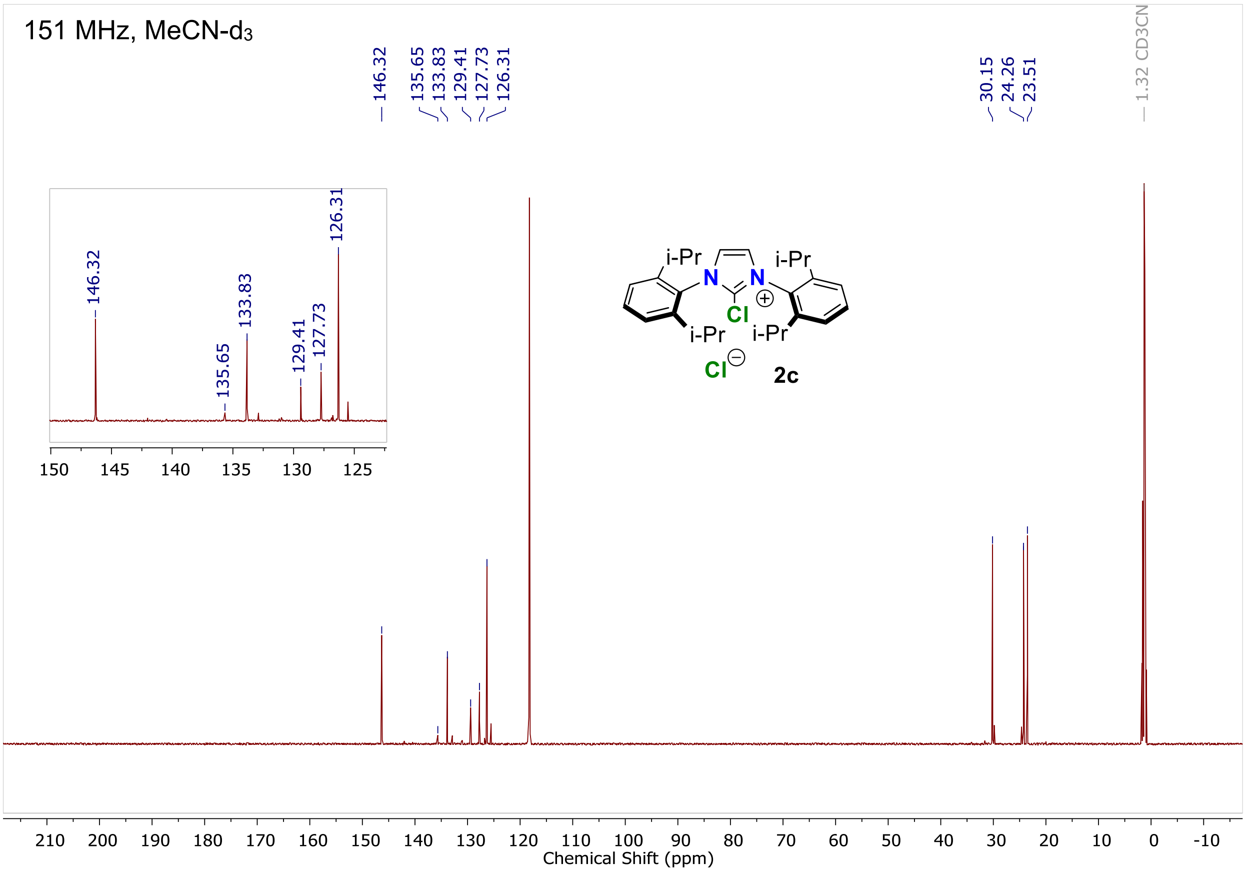

^{1}H NMR of 1,3-Bis(2,6-diisopropylphenyl)-2-imidazolone (3)

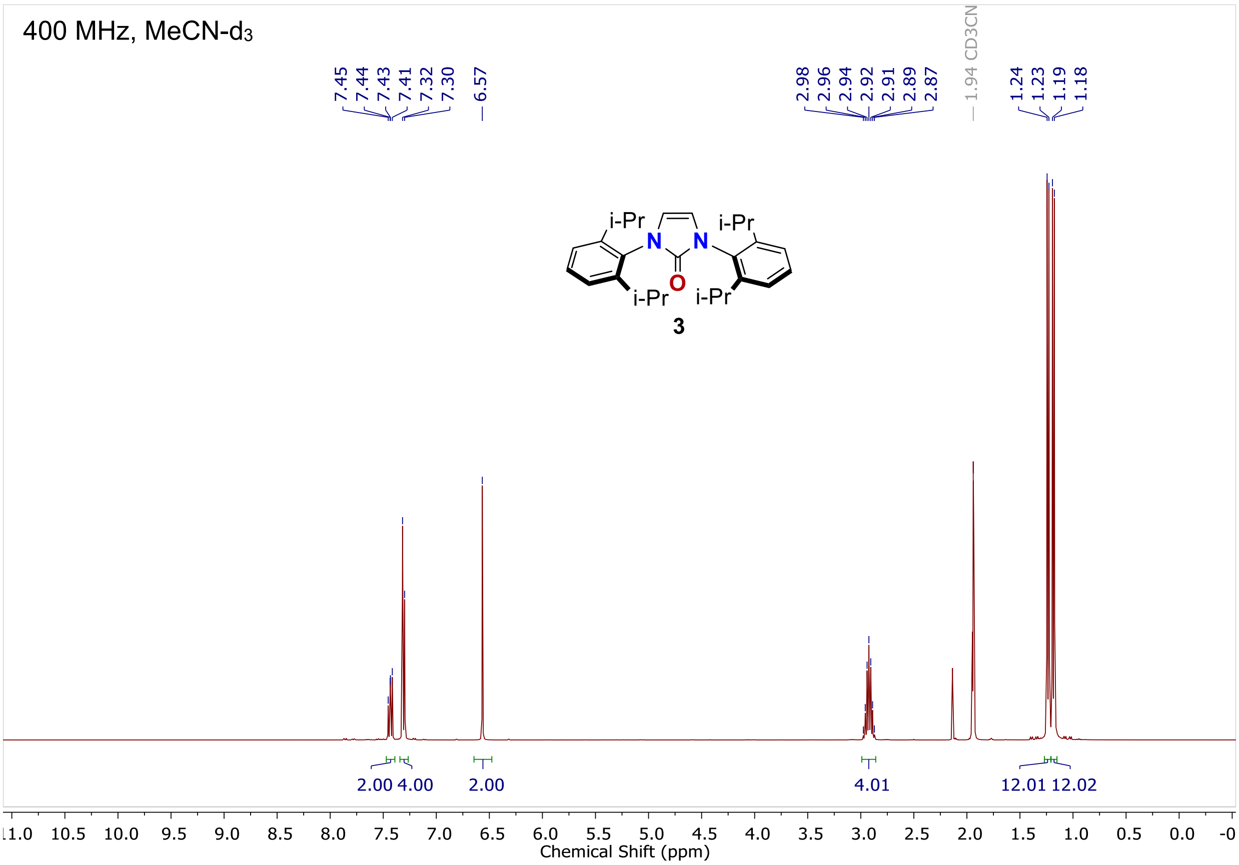

^{13}C NMR of 1,3-Bis(2,6-diisopropylphenyl)-2-imidazolone (3)

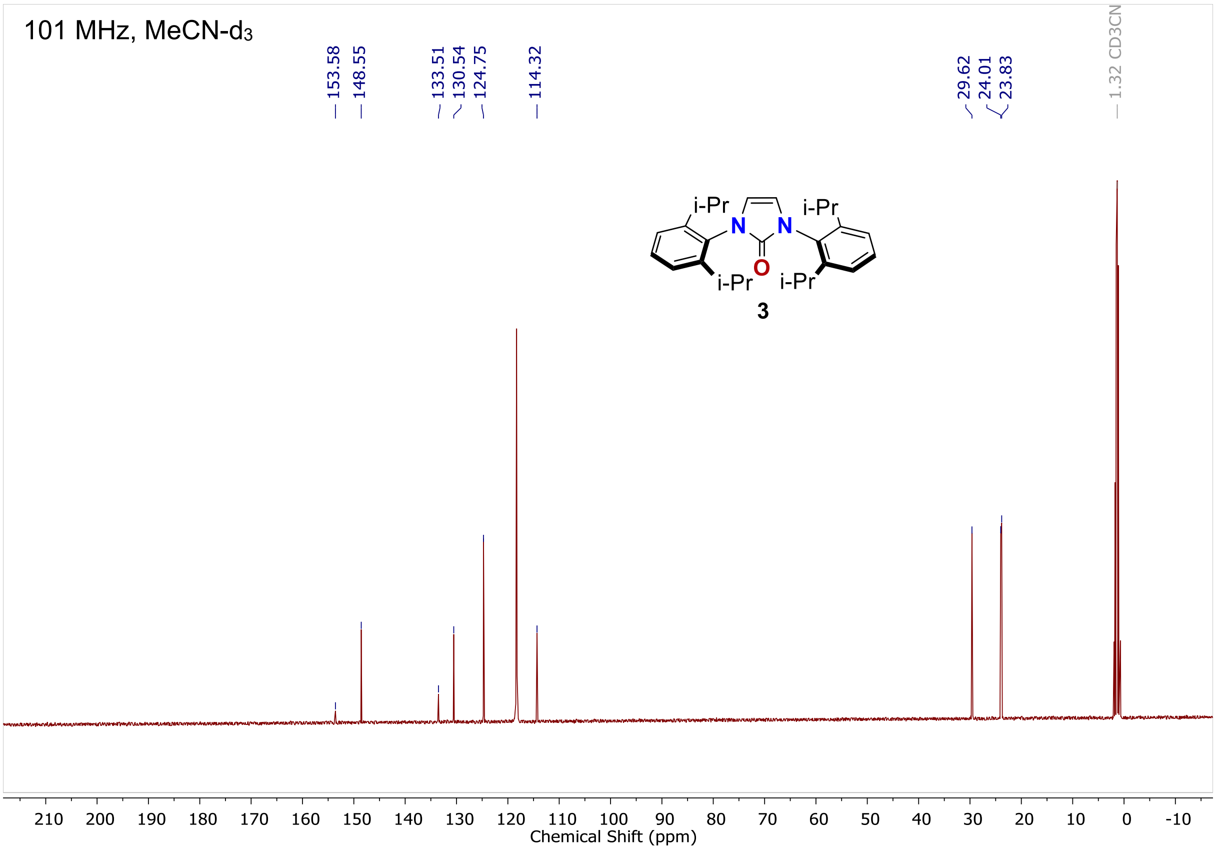

^{1}H NMR of Menthyl 6-hydroxynicotinate (4s)

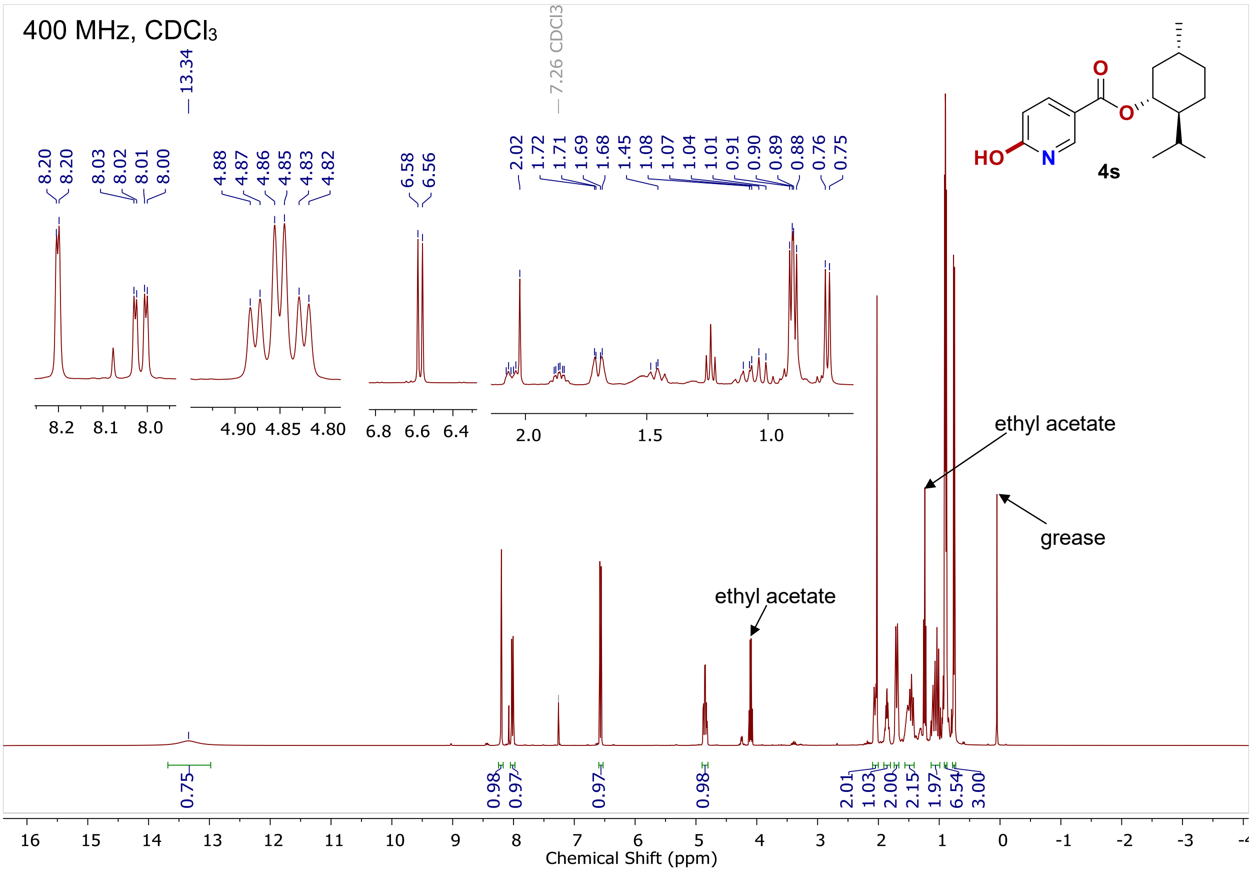

^{13}C NMR of Menthyl 6-hydroxynicotinate (4s)

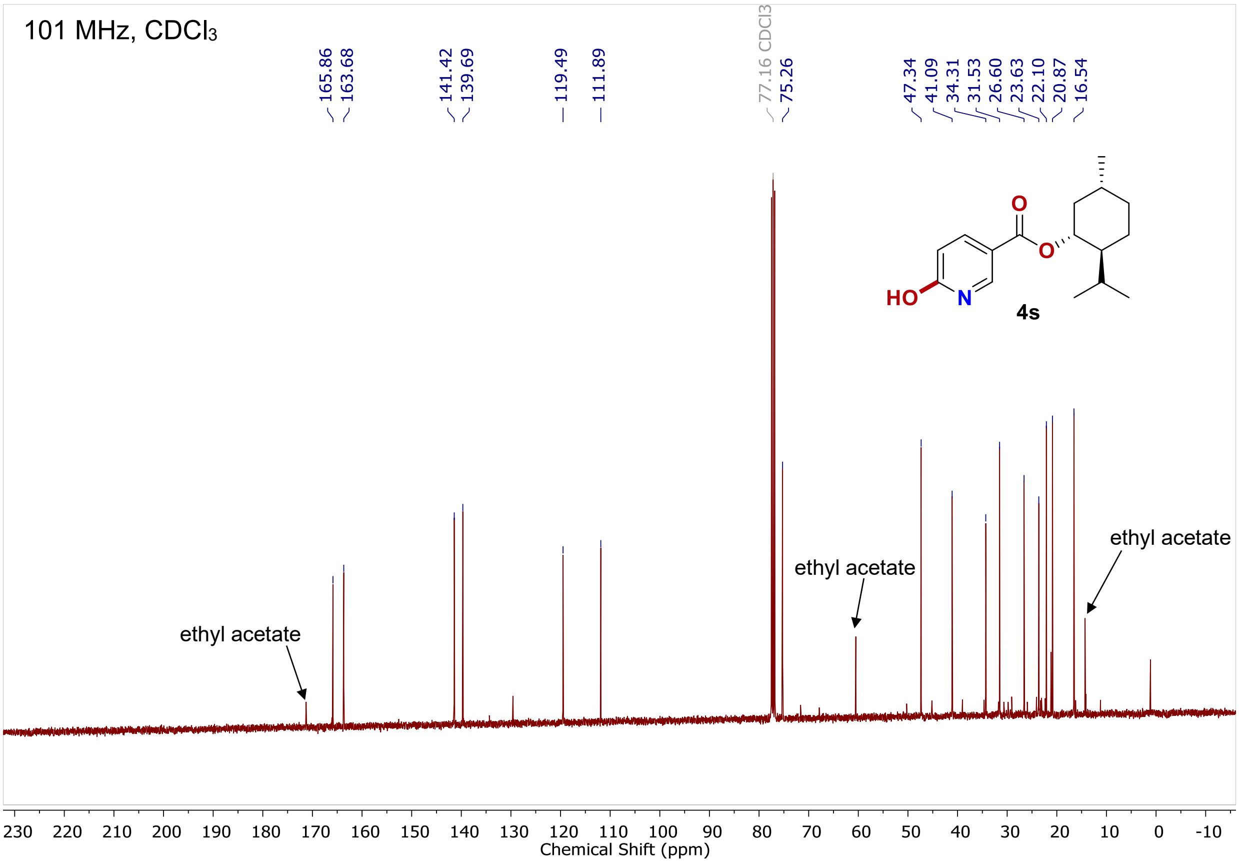

^{1}H NMR of 4-Fluorobenzophenone (5a)

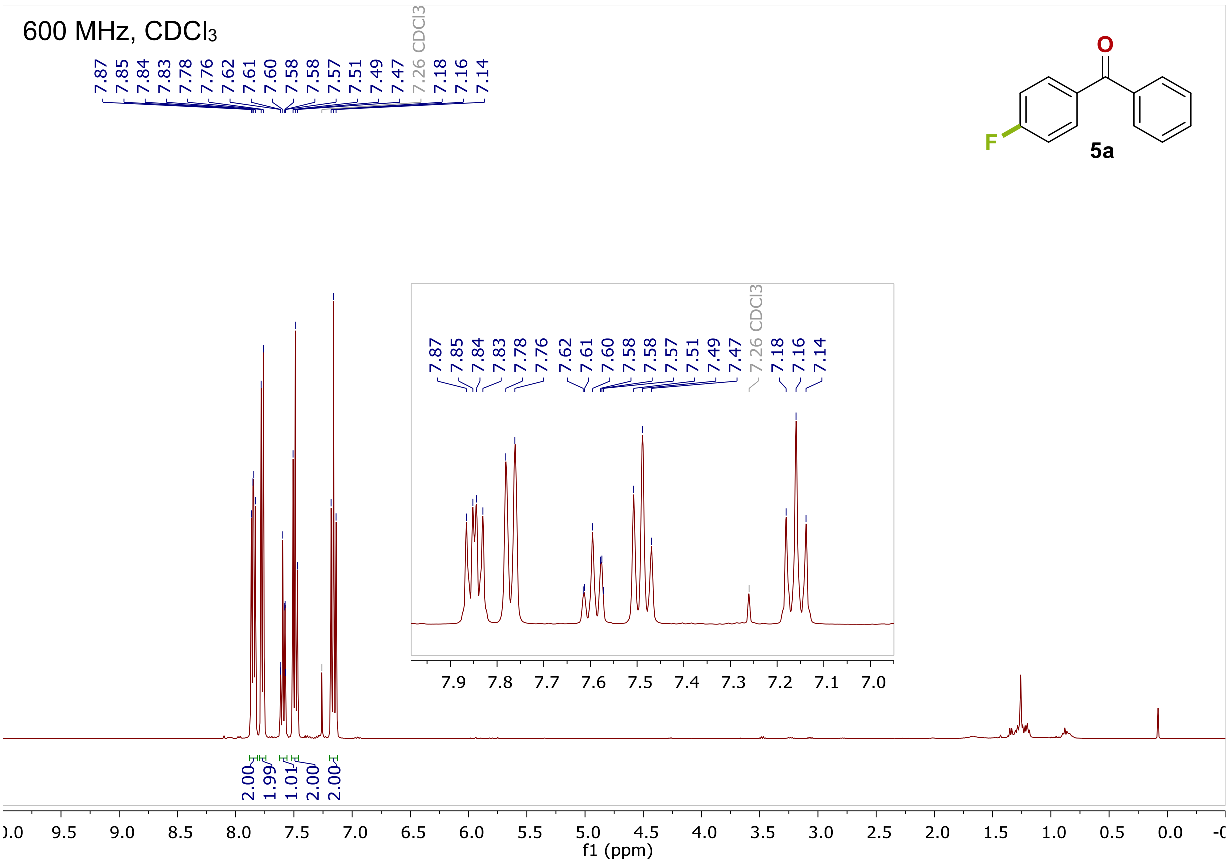

^{19}F NMR of 4-Fluorobenzophenone (5a)

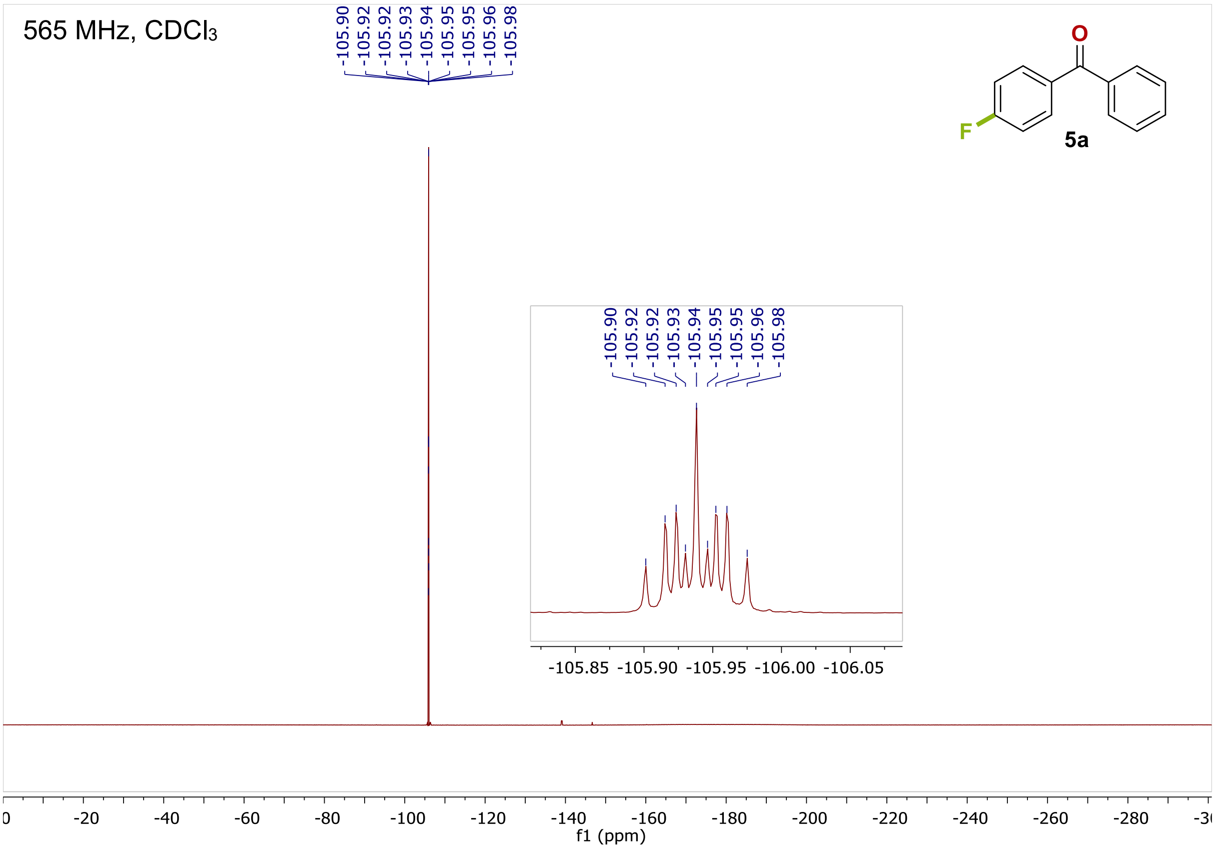

^{1}H NMR of 4-Fluoroacetophenone (5b)

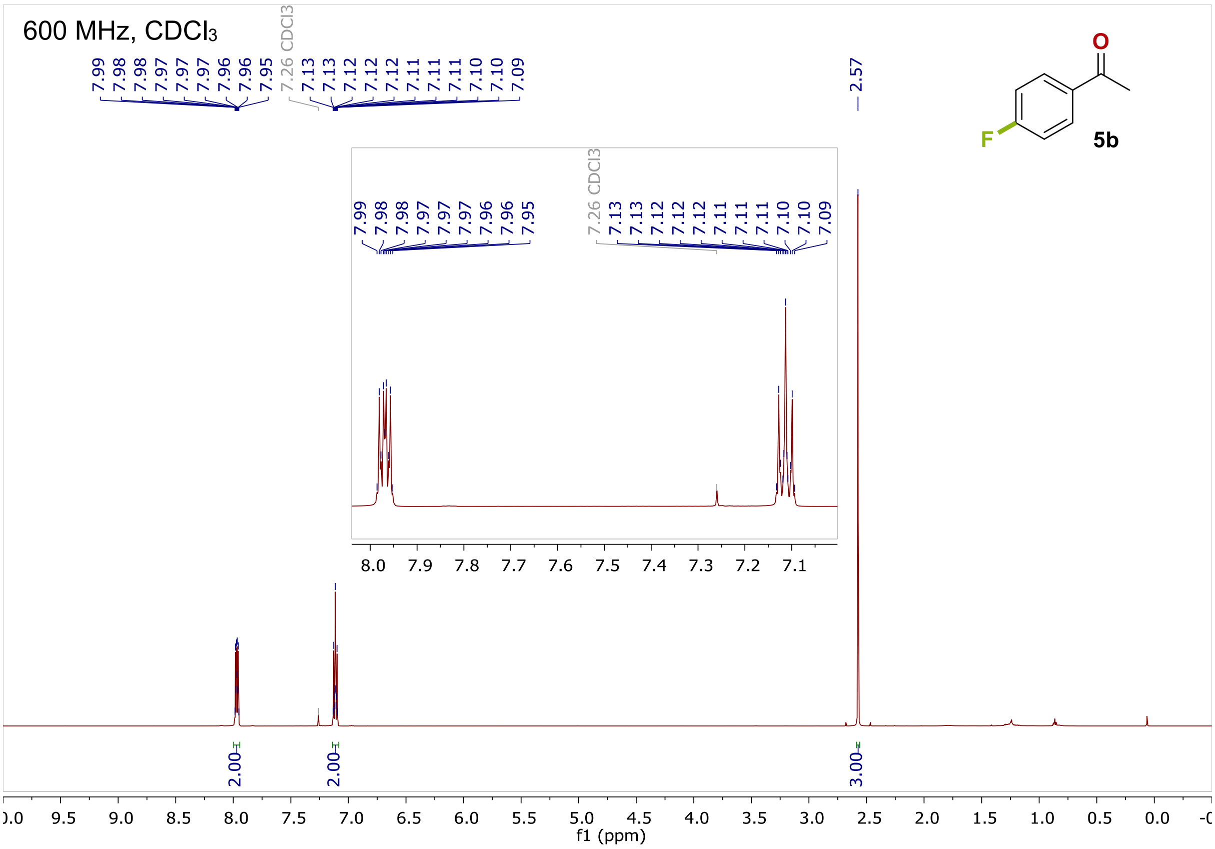

^{13}C NMR of 4-Fluoroacetophenone (5b)

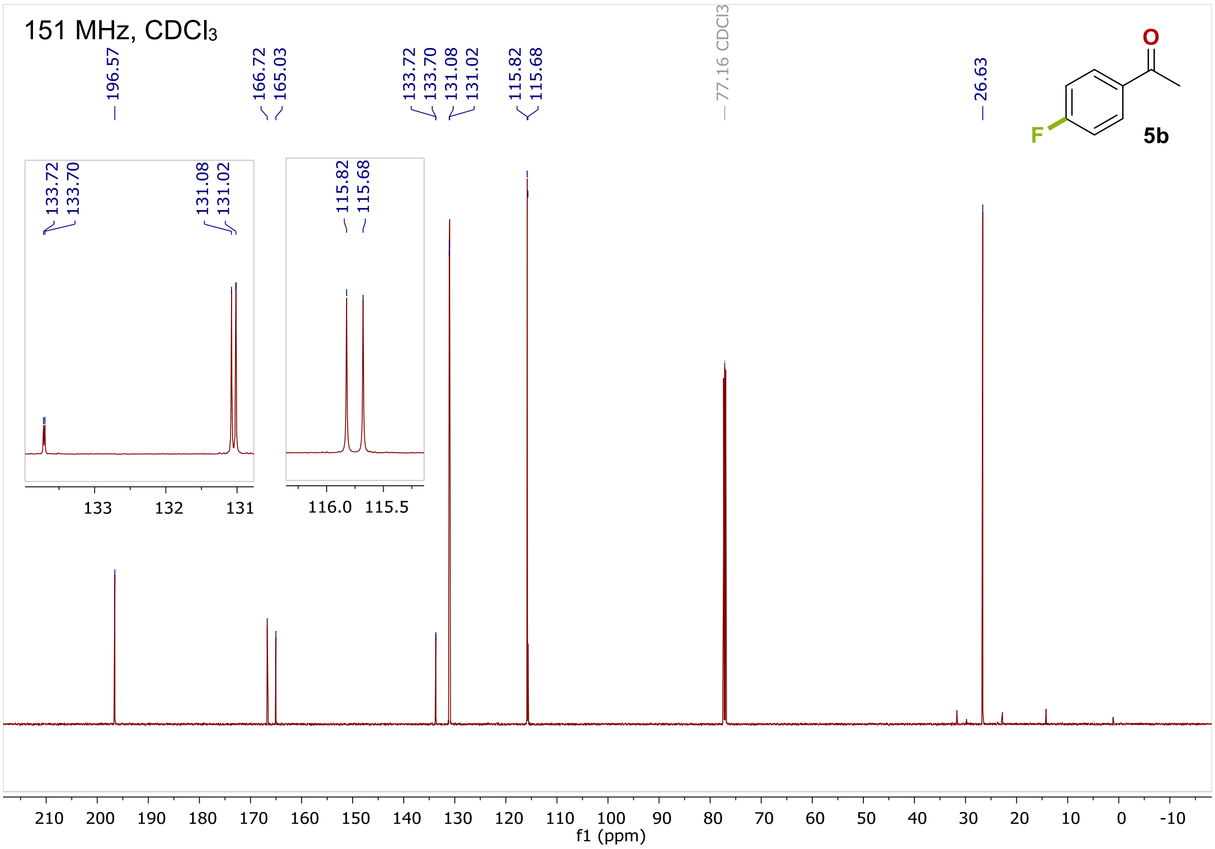

^{19}F NMR of 4-Fluoroacetophenone (5b)

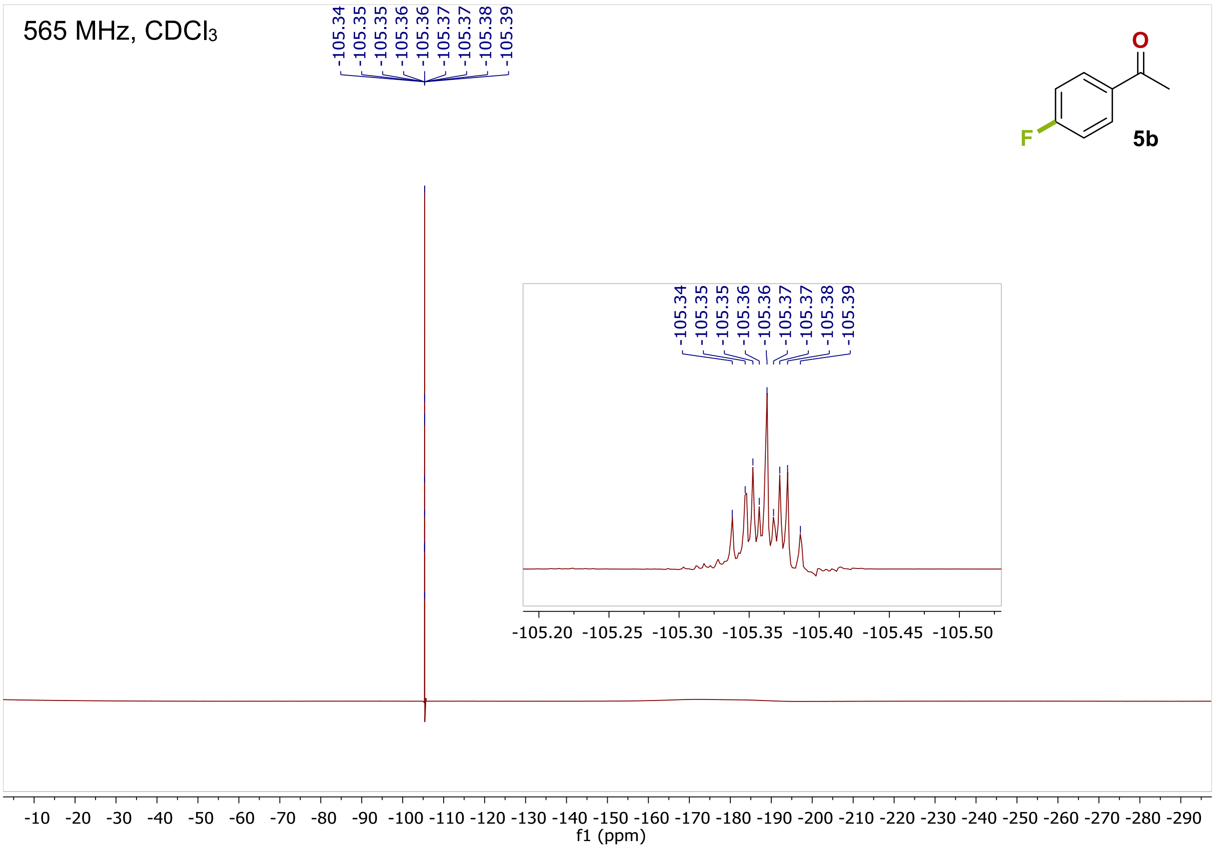

^{1}H NMR of 4-Fluoronitrobenzene (5c)

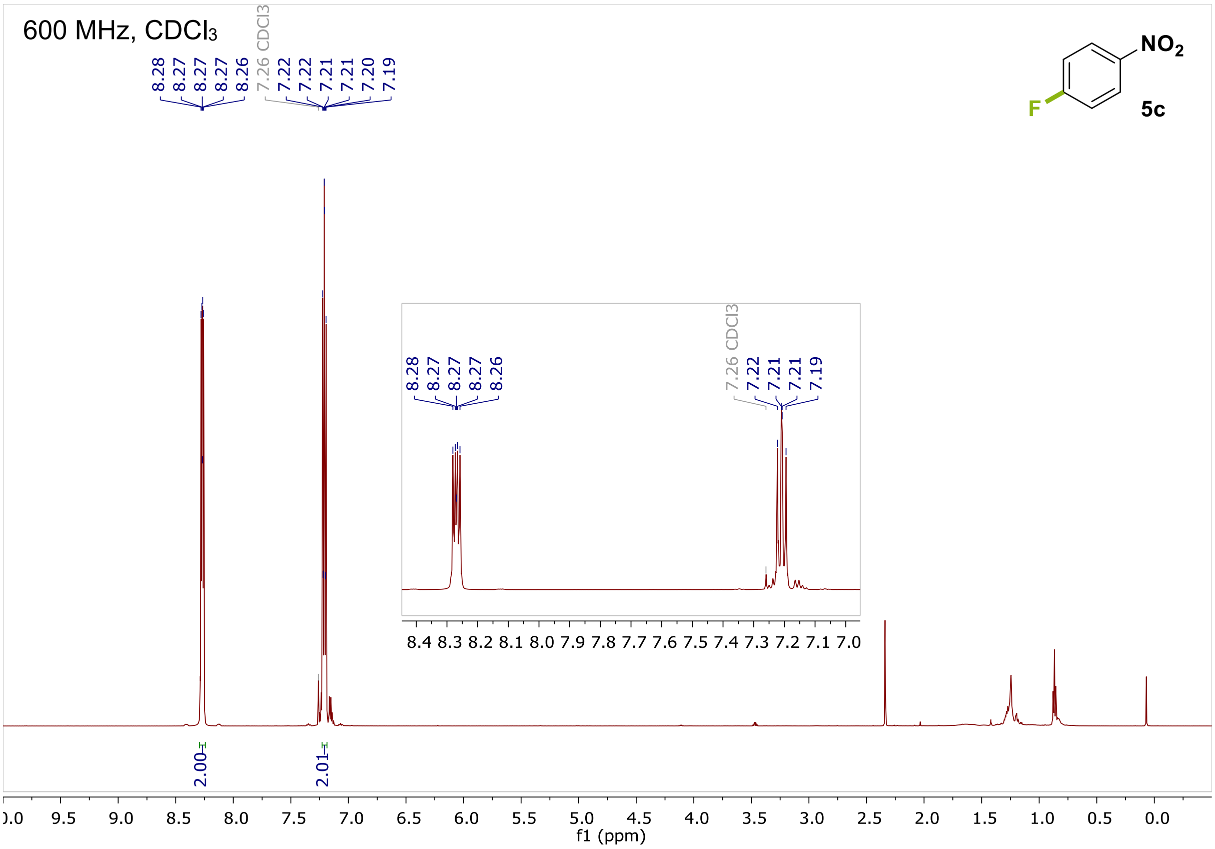

^{13}C NMR of 4-Fluoronitrobenzene (5c)

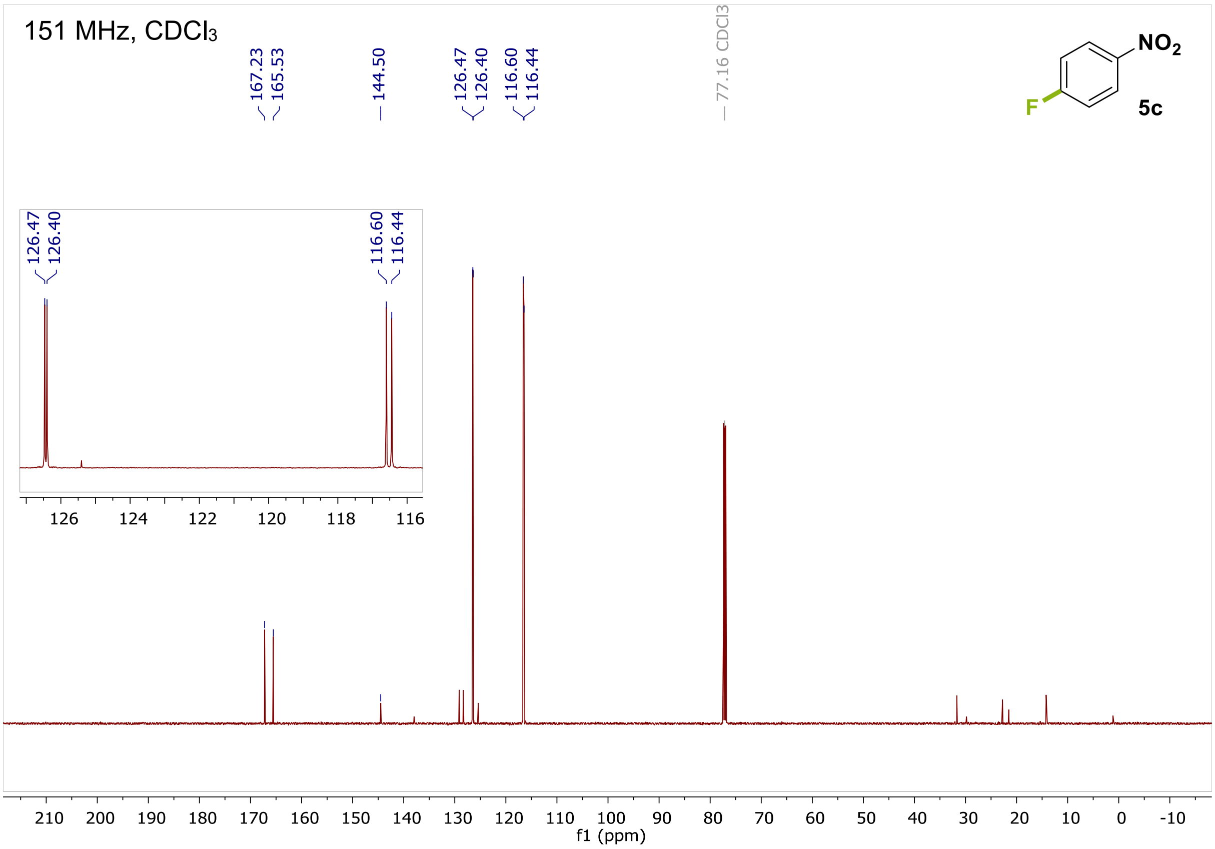

^{19}F NMR of 4-Fluoronitrobenzene (5c)

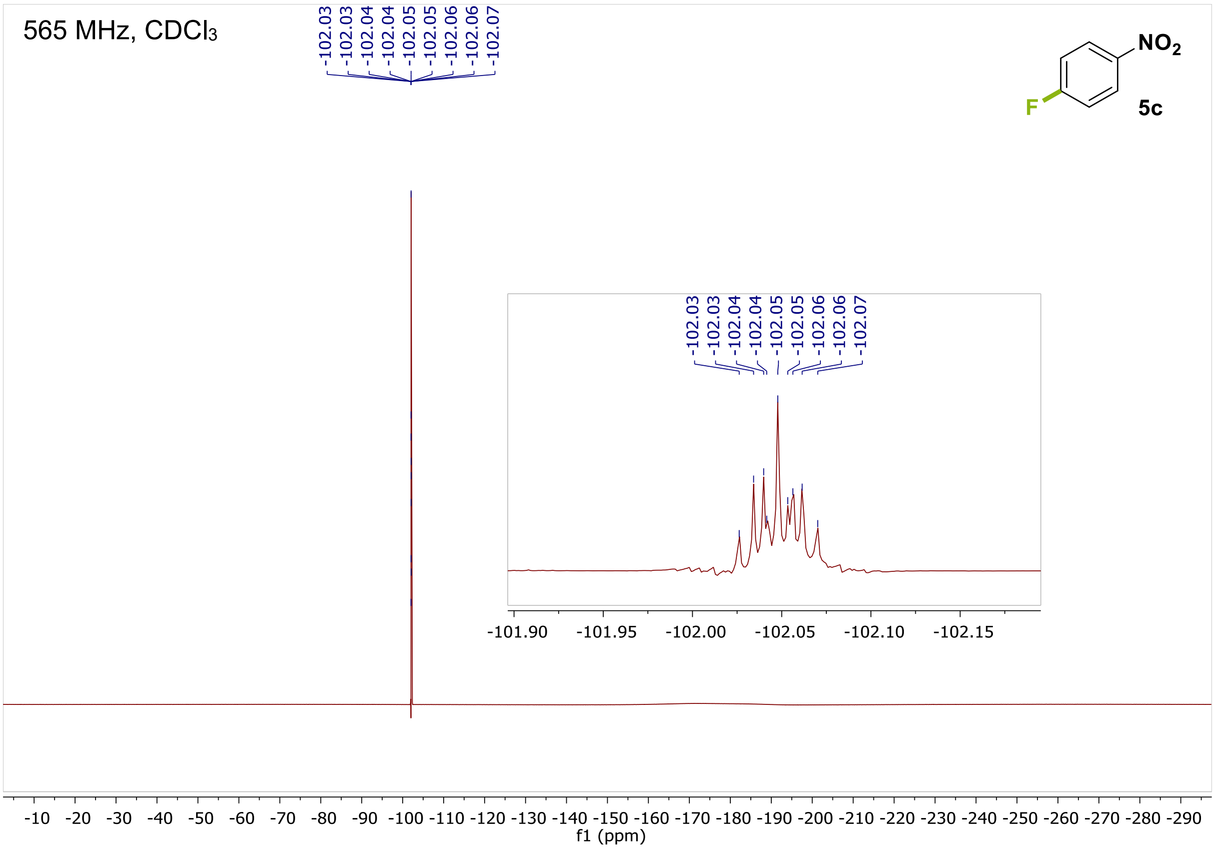

^{1}H NMR of 4-Fluorobenzaldehyde (5e)

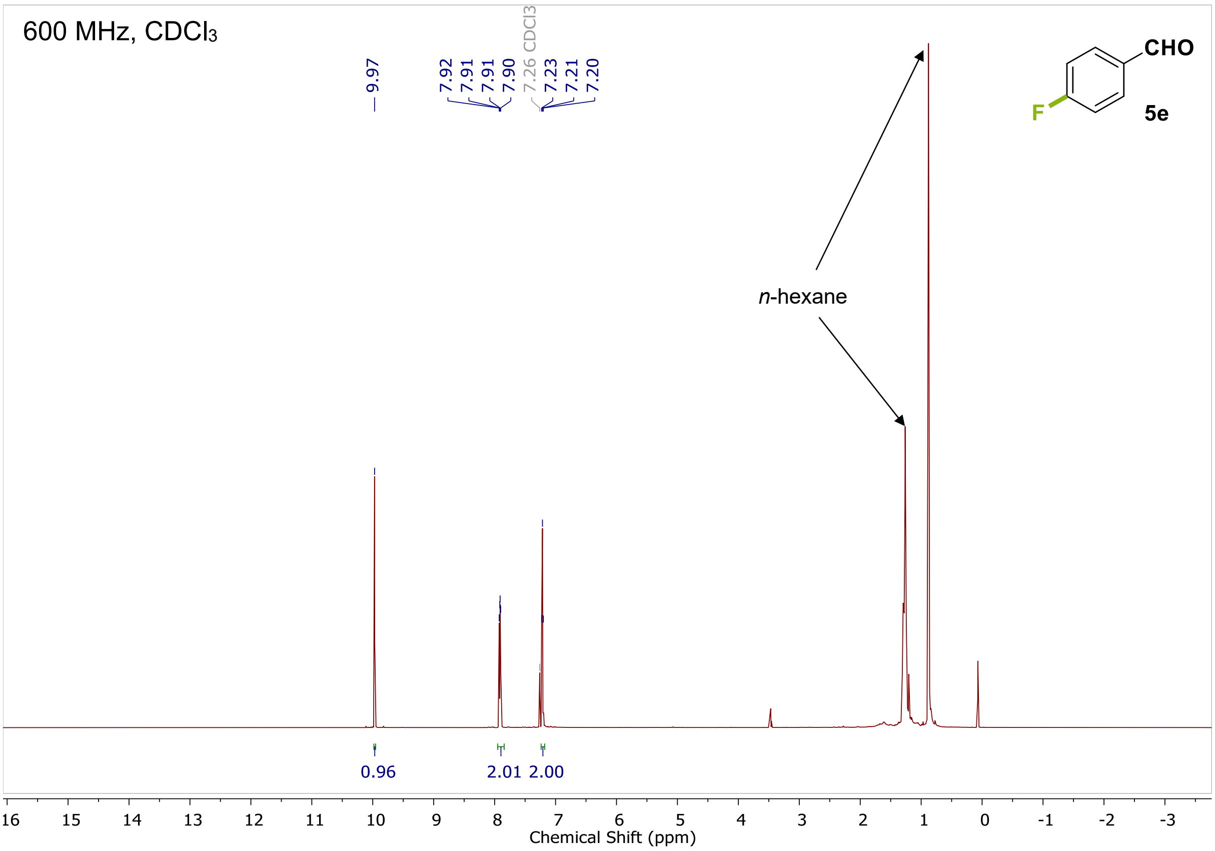

^{13}C NMR of 4-Fluorobenzaldehyde (5e)

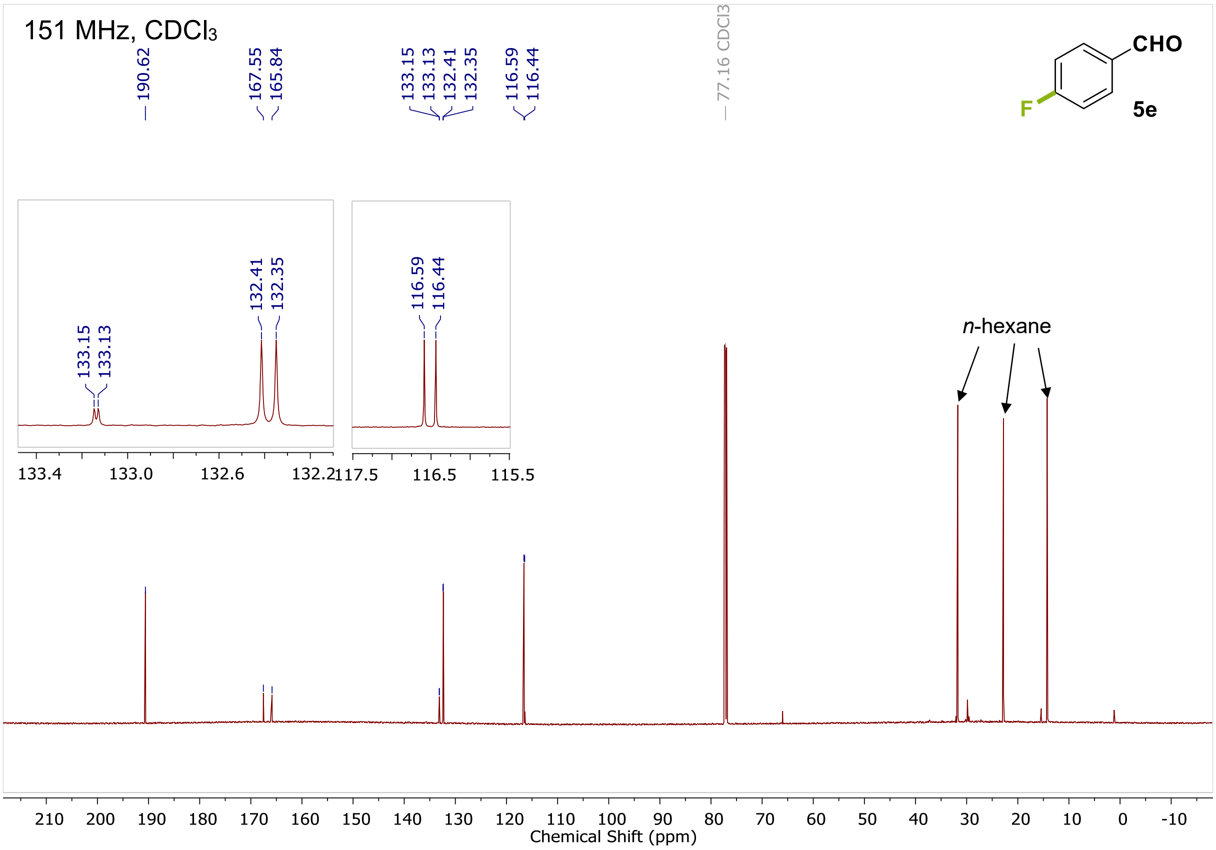

^{1}H NMR of Ethyl 4-fluorobenzoate (5f)

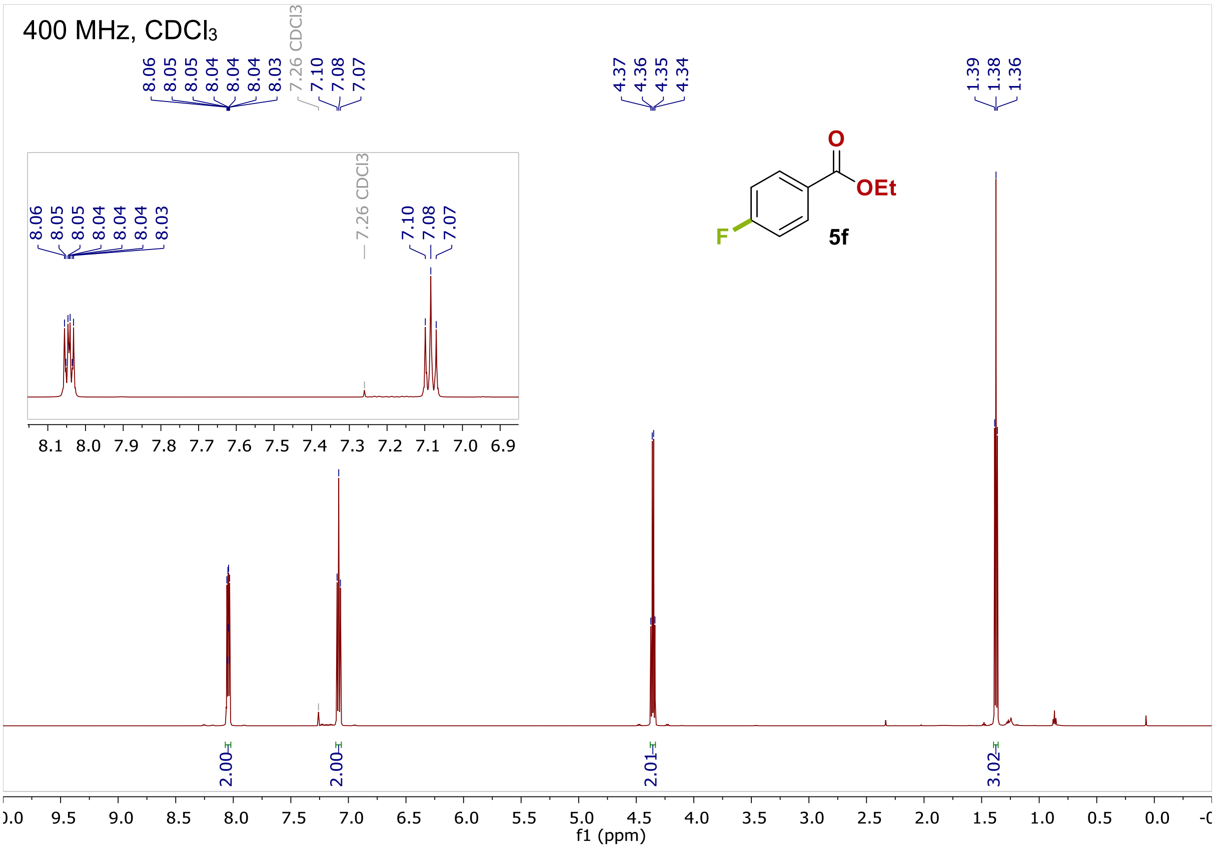

^{13}C NMR Spectra of Ethyl 4-fluorobenzoate (5f)

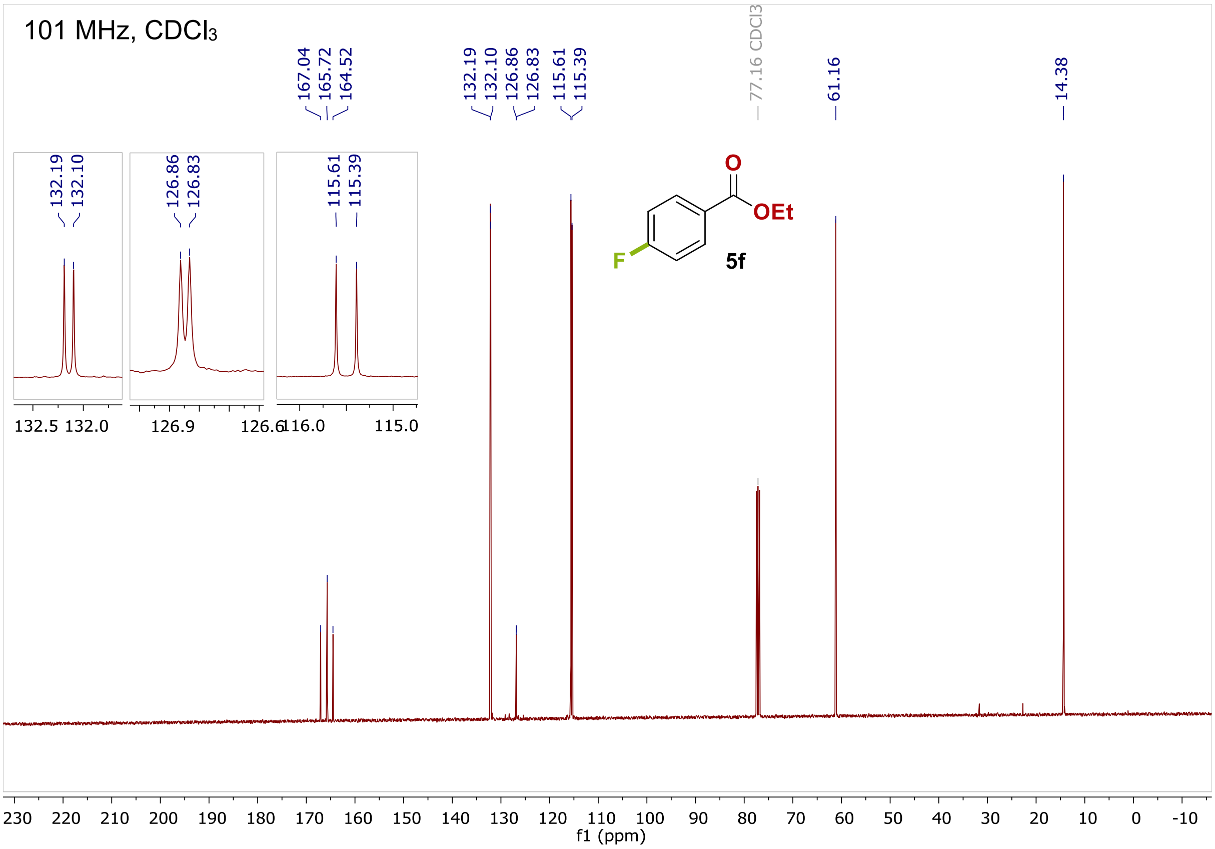

^{19}F NMR Spectra of Ethyl 4-fluorobenzoate (5f)

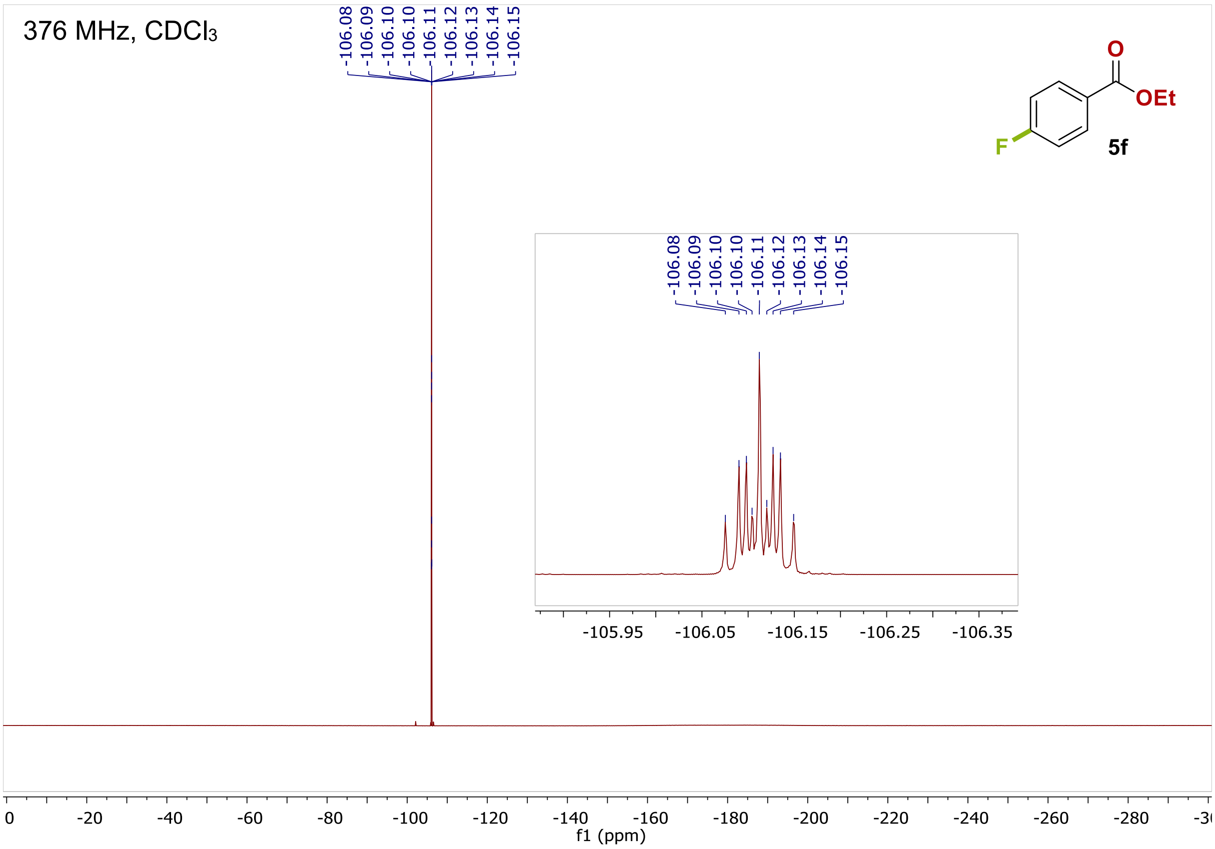

^{1}H NMR of Ethyl 4-fluorocinnamate (5g)

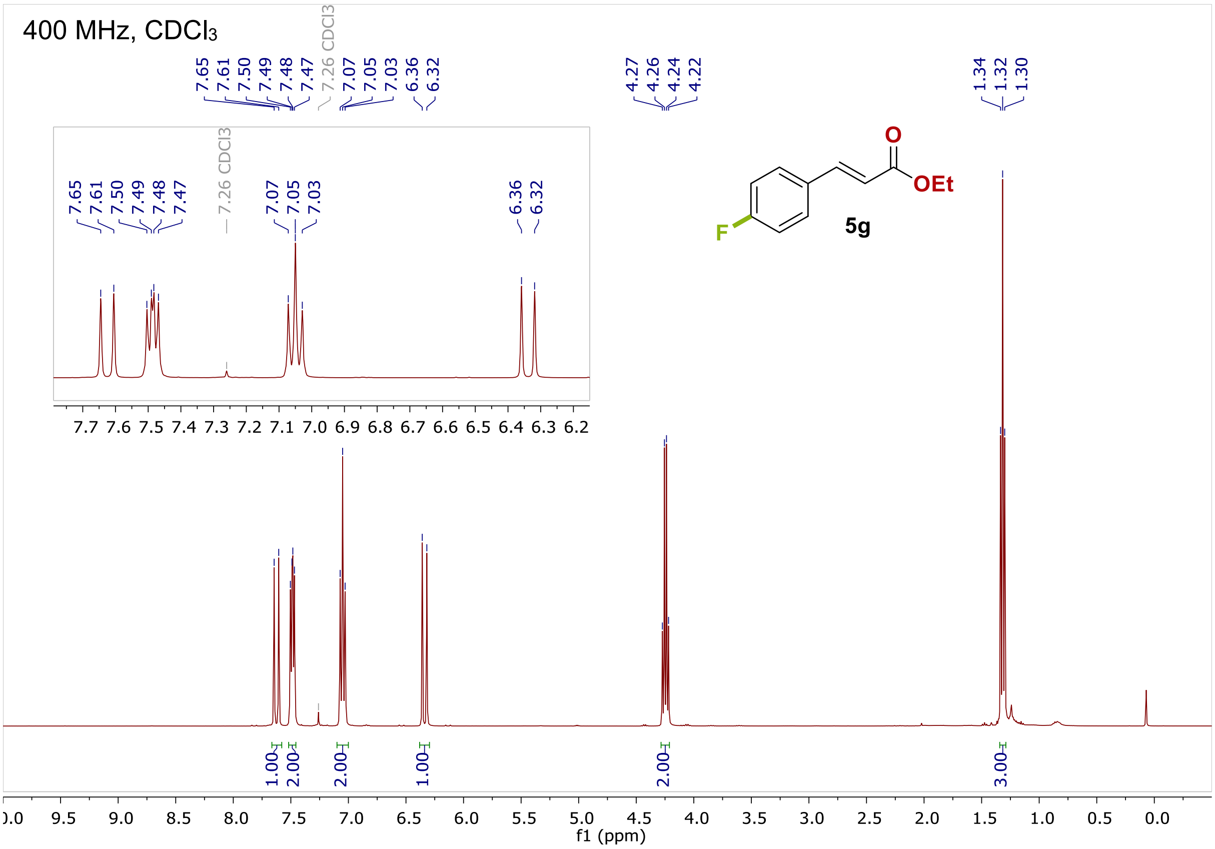

^{13}C NMR of Ethyl 4-fluorocinnamate (5g)

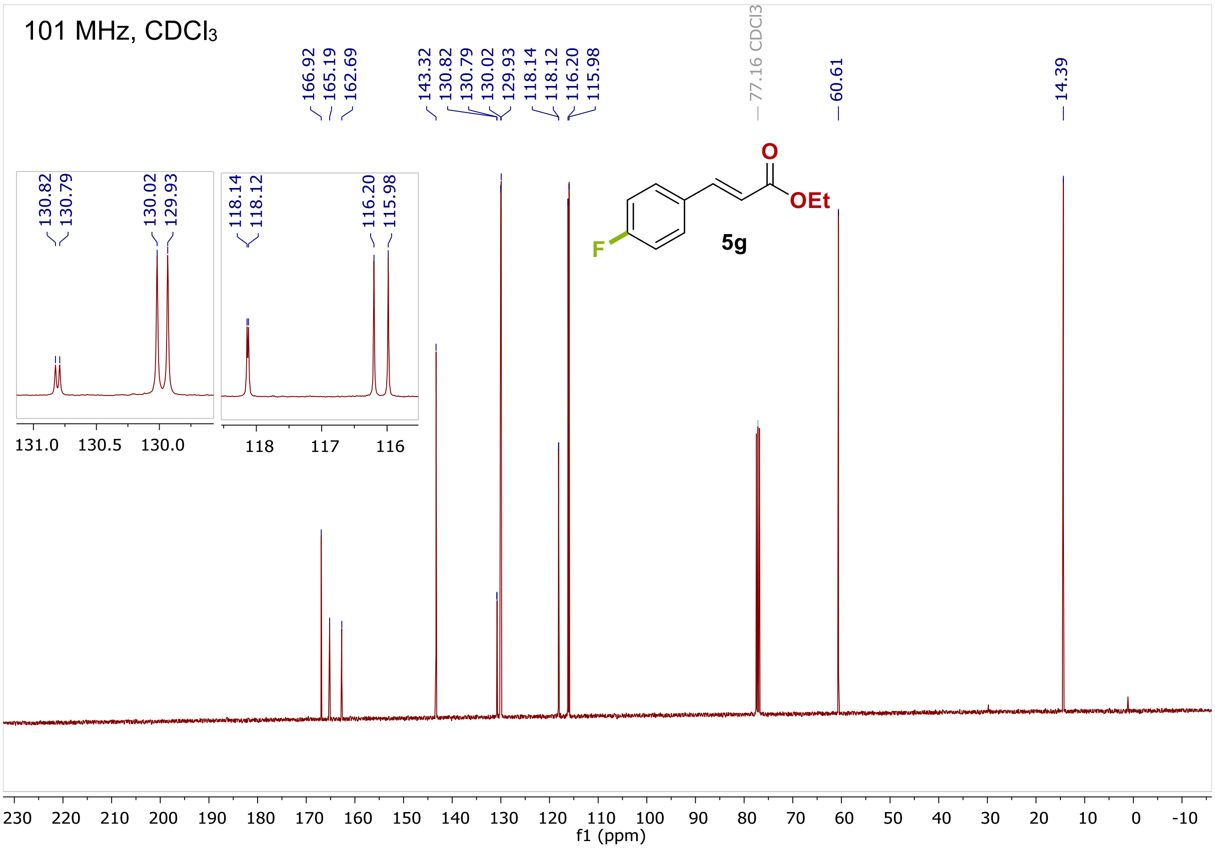

^{19}F NMR of Ethyl 4-fluorocinnamate (5g)

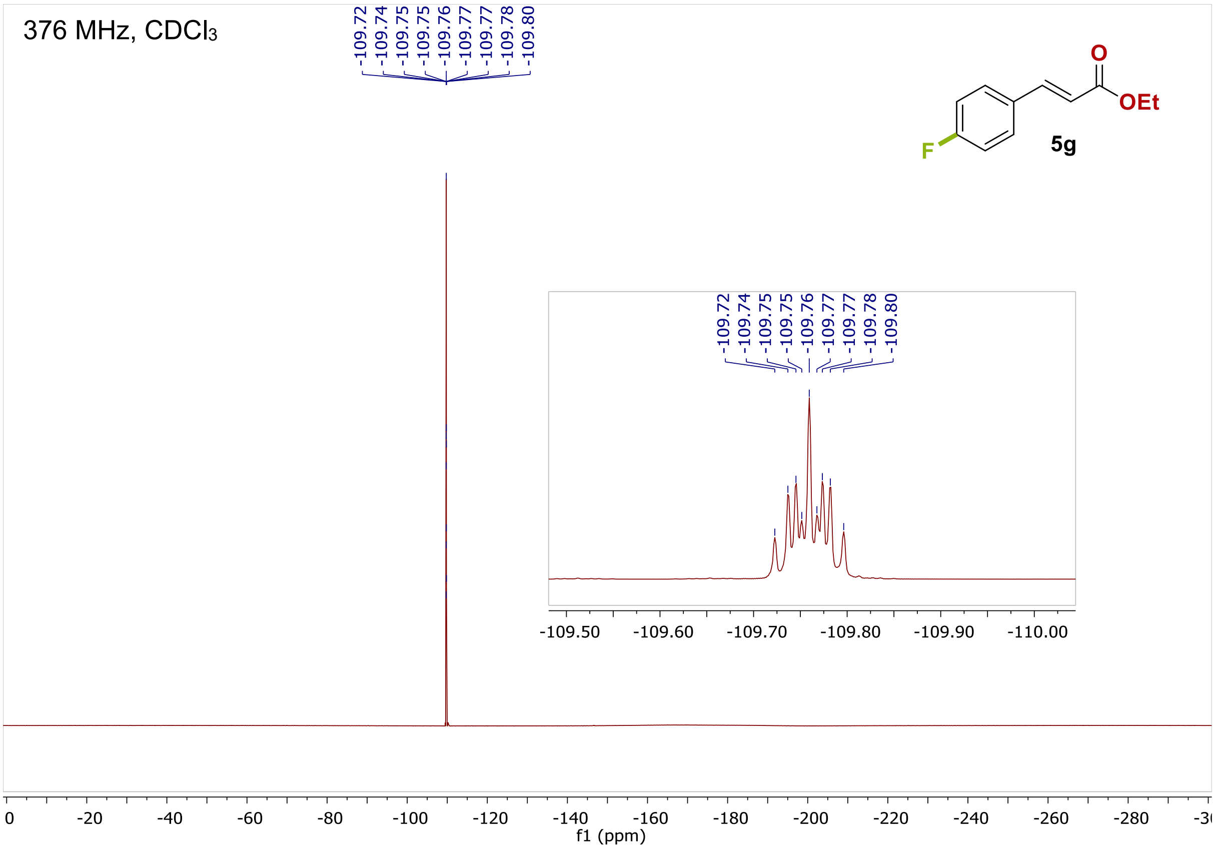

^{1}H NMR of 4-Fluorochalcone (5h)

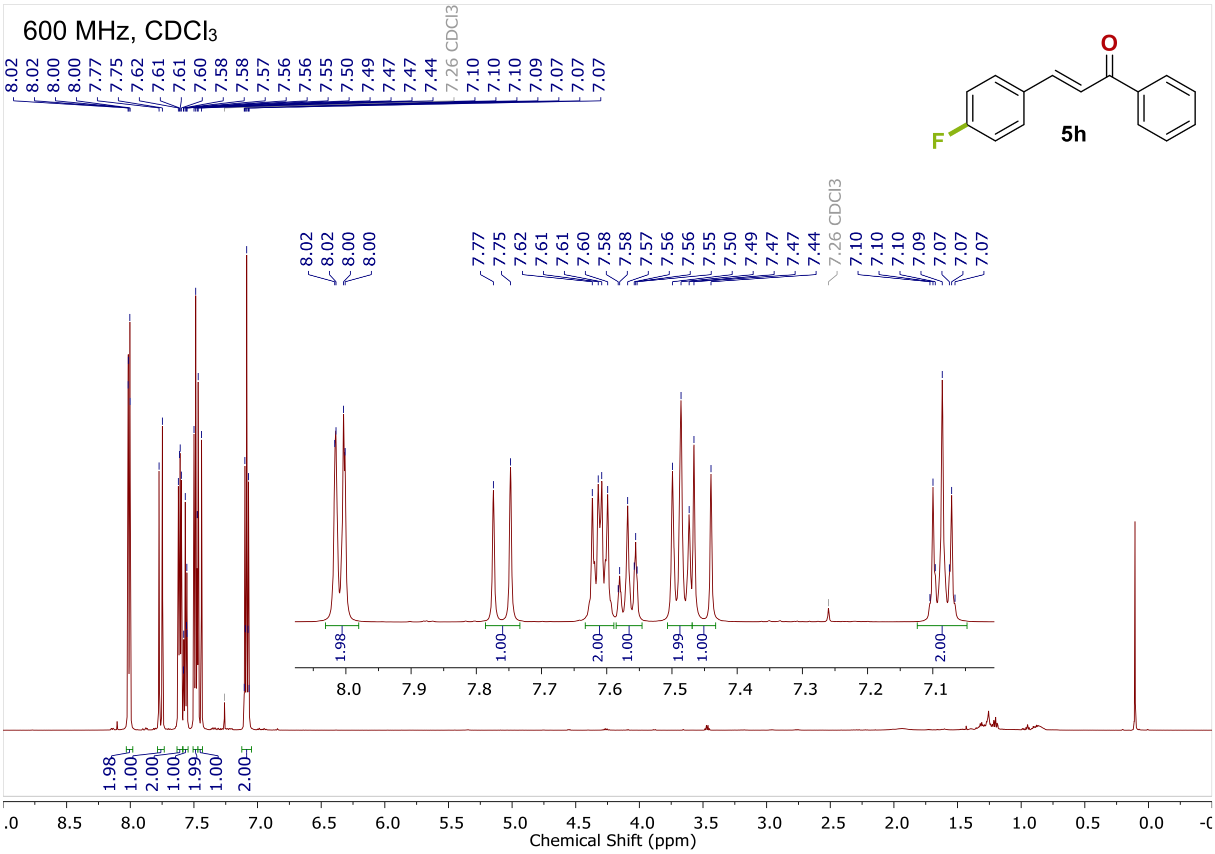

^{13}C NMR of 4-Fluorochalcone (5h)

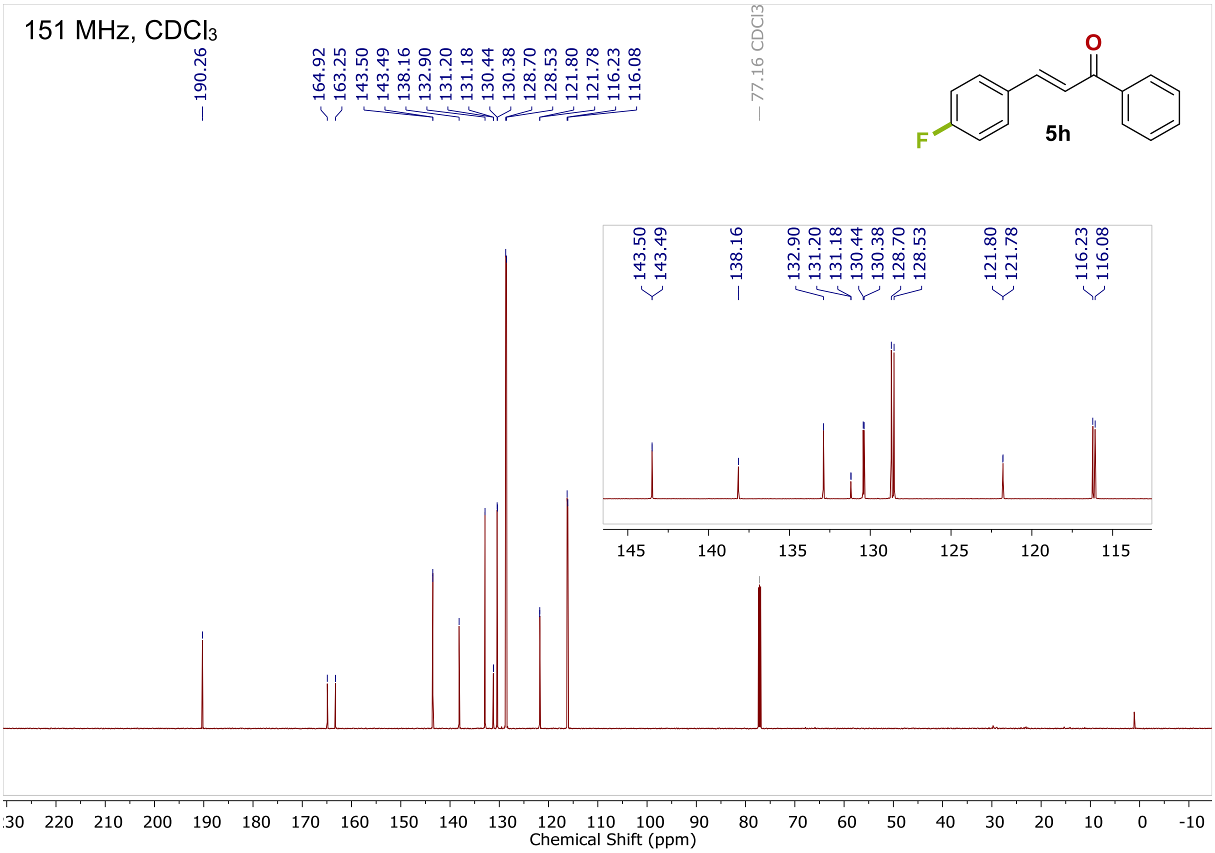

^{19}F NMR of 4-Fluorochalcone (5h)

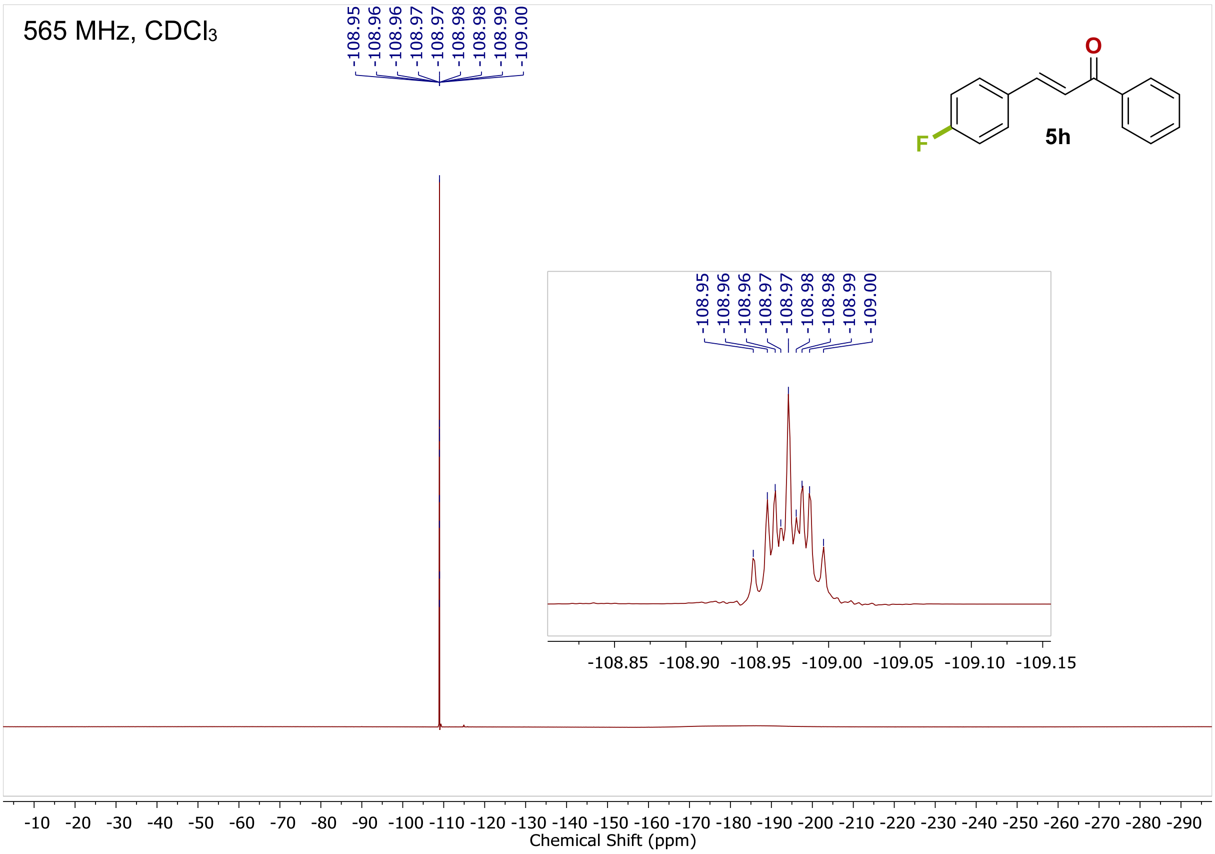

^{1}H NMR of 4'-Fluorochalcone (5i)

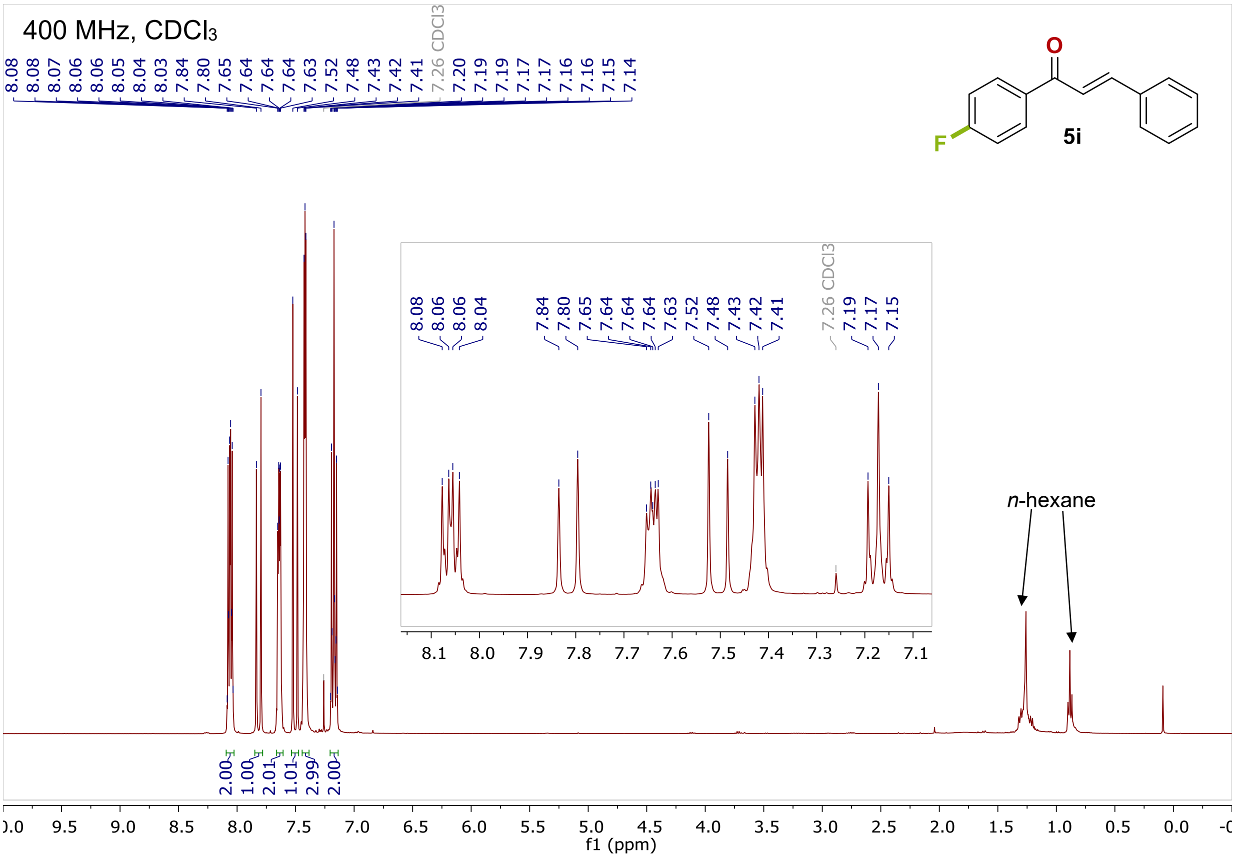

^{13}C NMR of 4'-Fluorochalcone (5i)

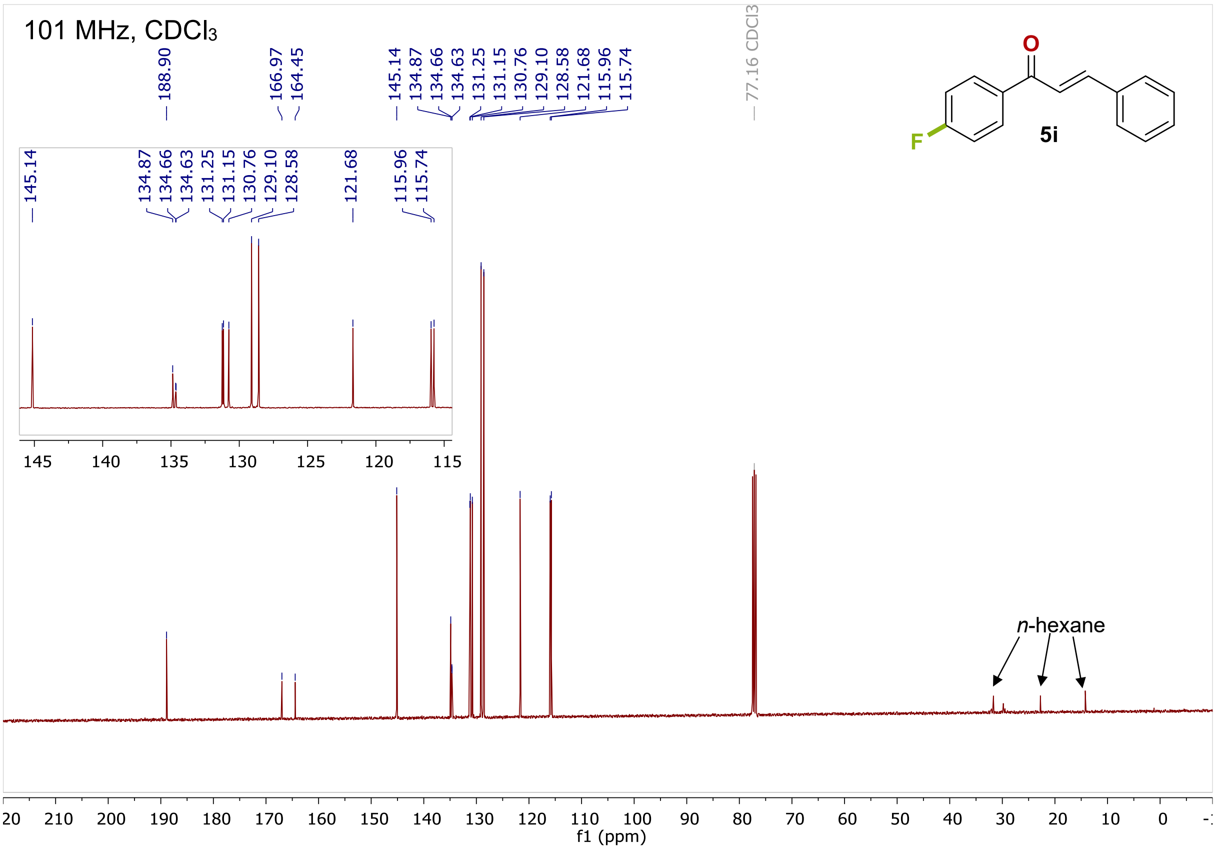

^{19}F NMR of 4'-Fluorochalcone (5i)

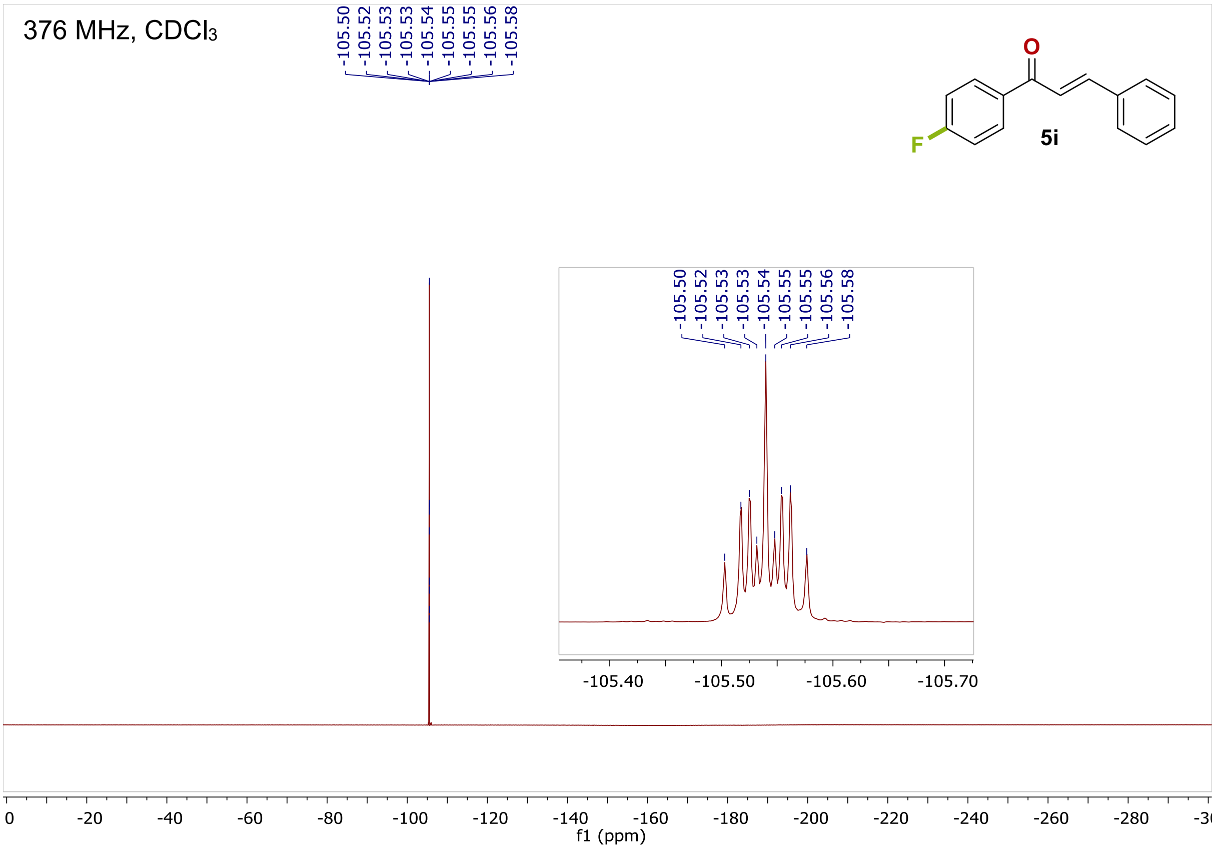

^{1}H NMR of 4-Fluoro-1-chloro-2-nitrobenzene (5k)

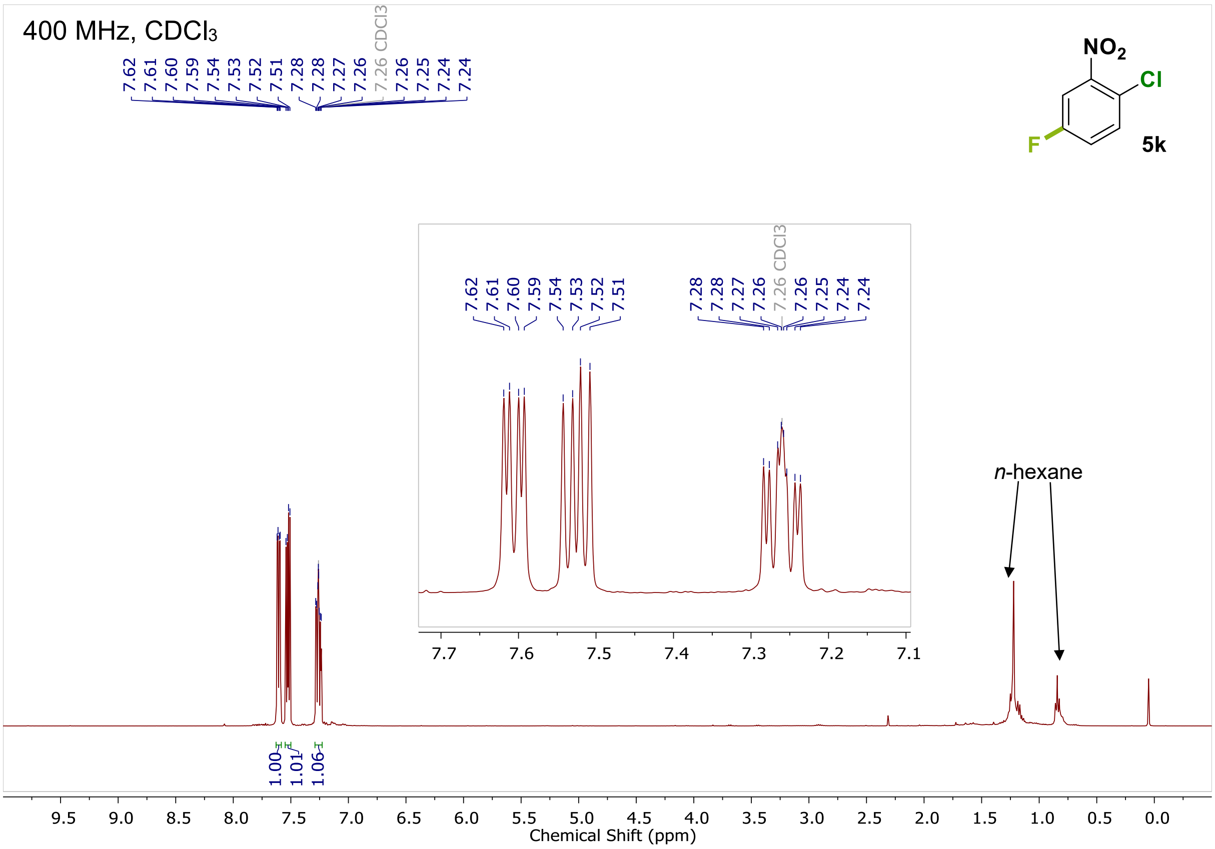

^{13}C NMR of 4-Fluoro-1-chloro-2-nitrobenzene (5k)

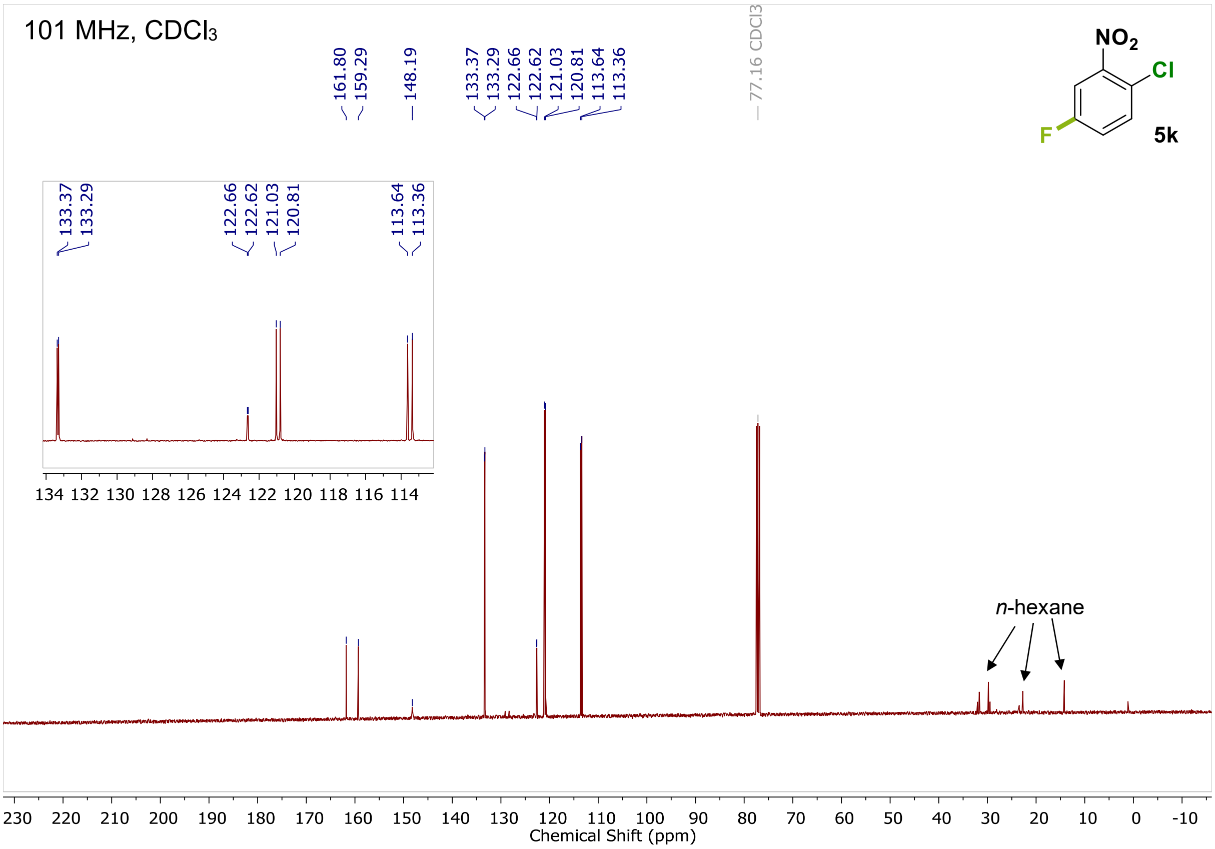

^{19}F NMR of 4-Fluoro-1-chloro-2-nitrobenzene (5k)

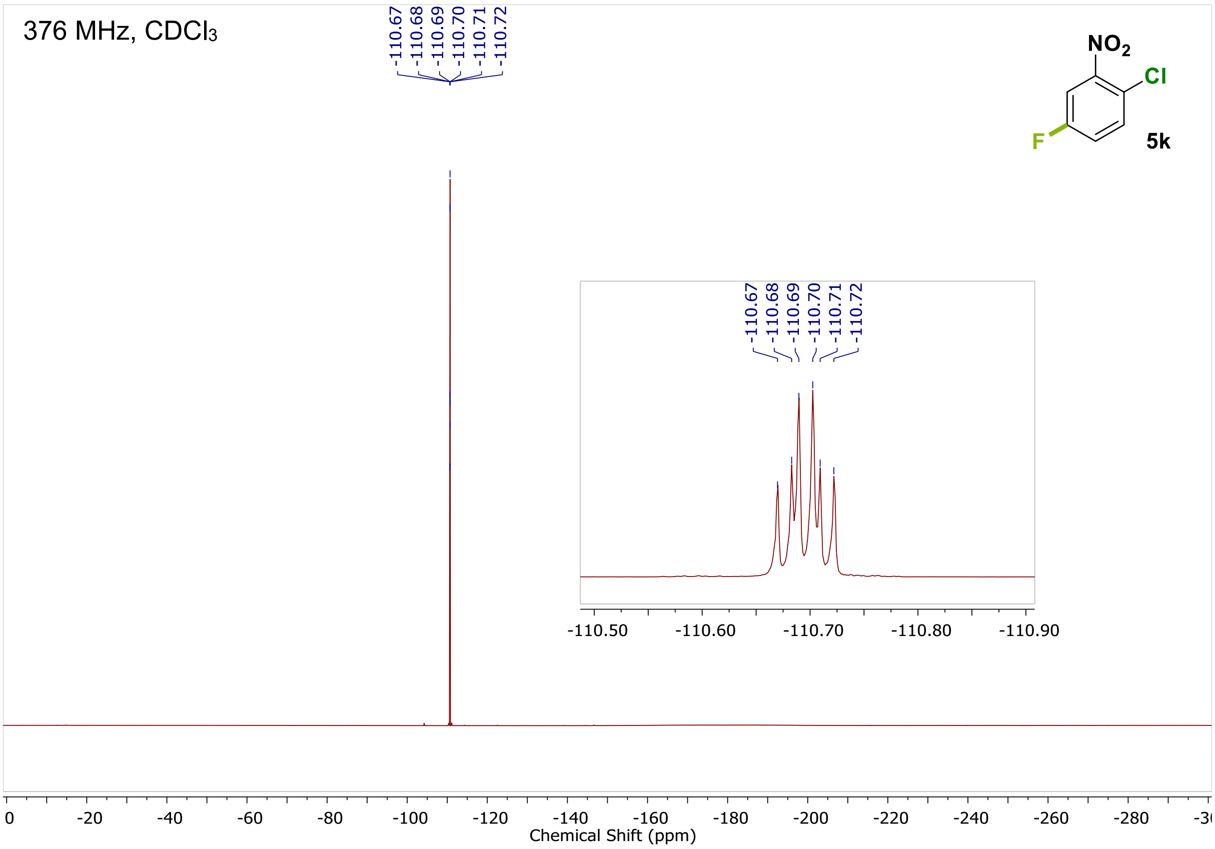

^{1}H NMR of 7-Fluorocoumarin (5m)

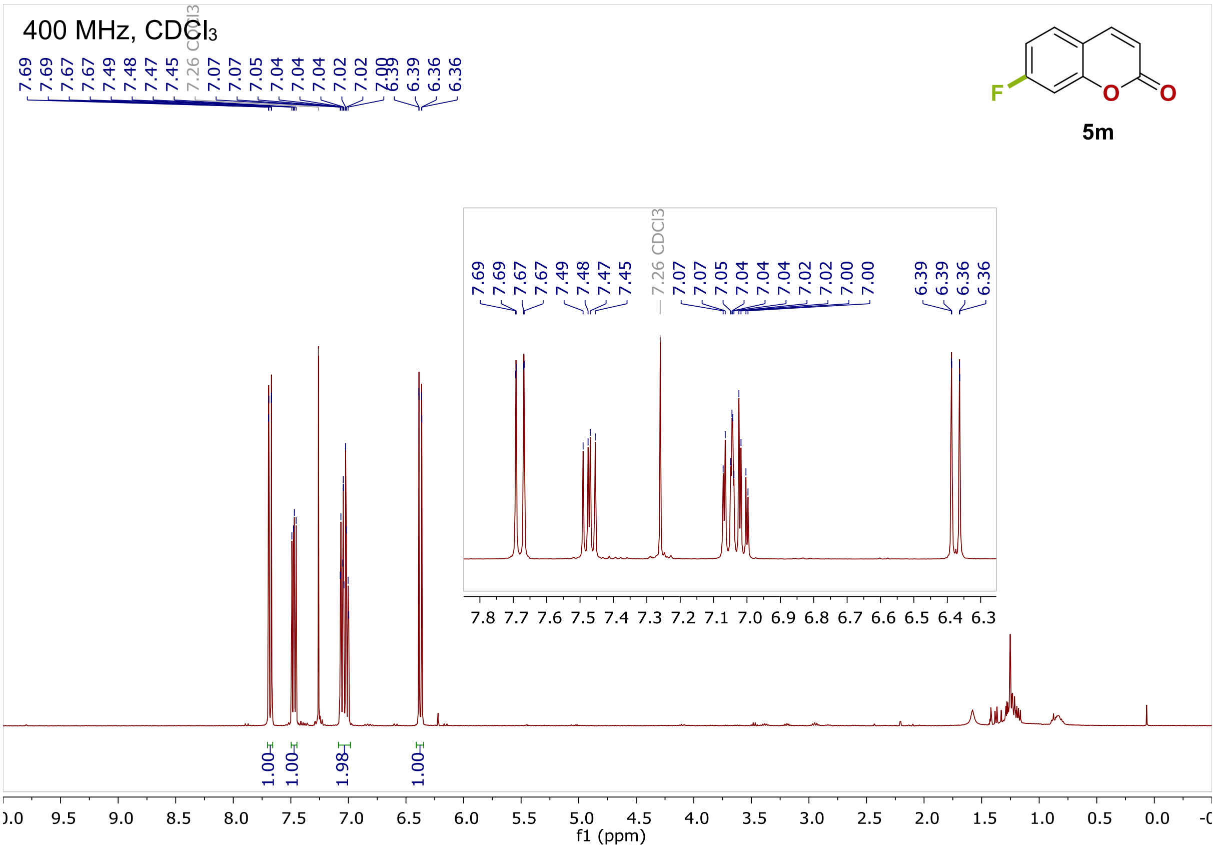

^{19}F NMR of 7-Fluorocoumarin (5m)

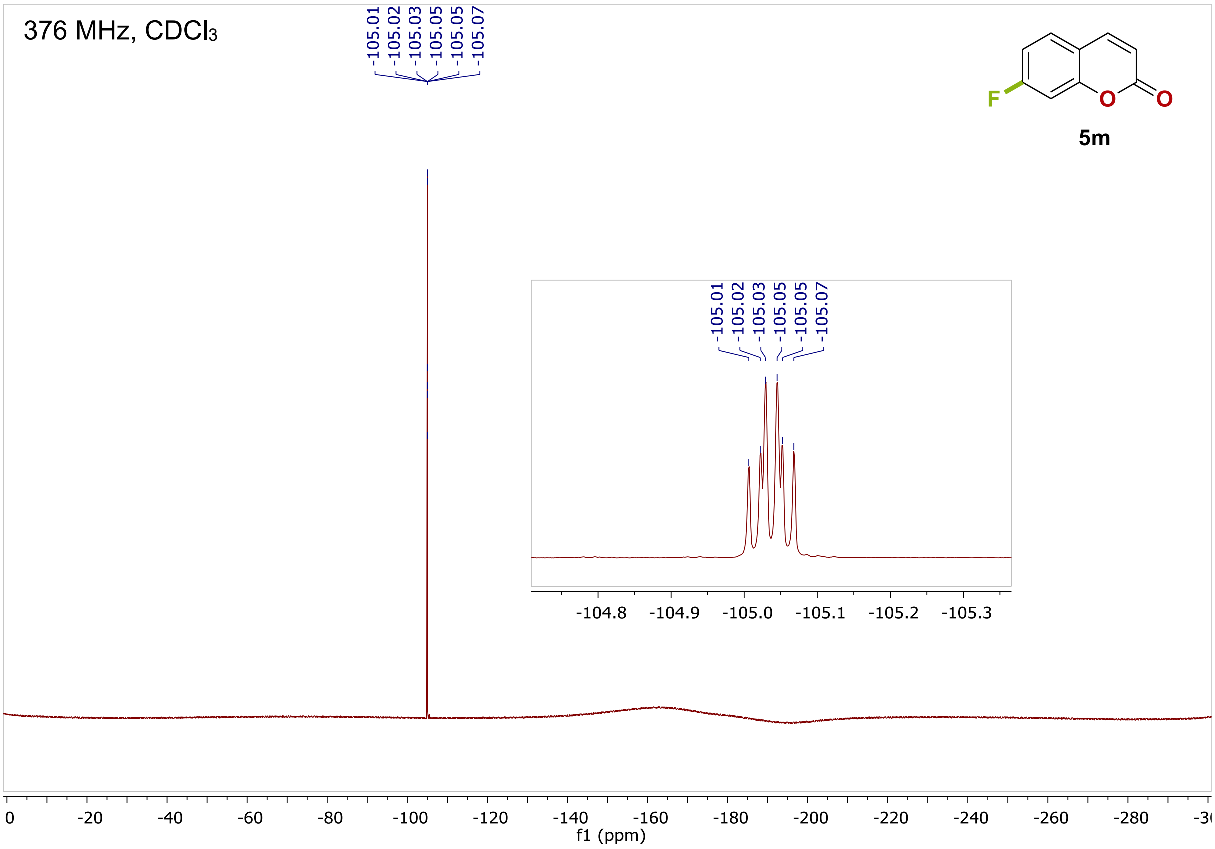

^{1}H NMR of 8-Fluoro-5-nitroquinoline (5n)

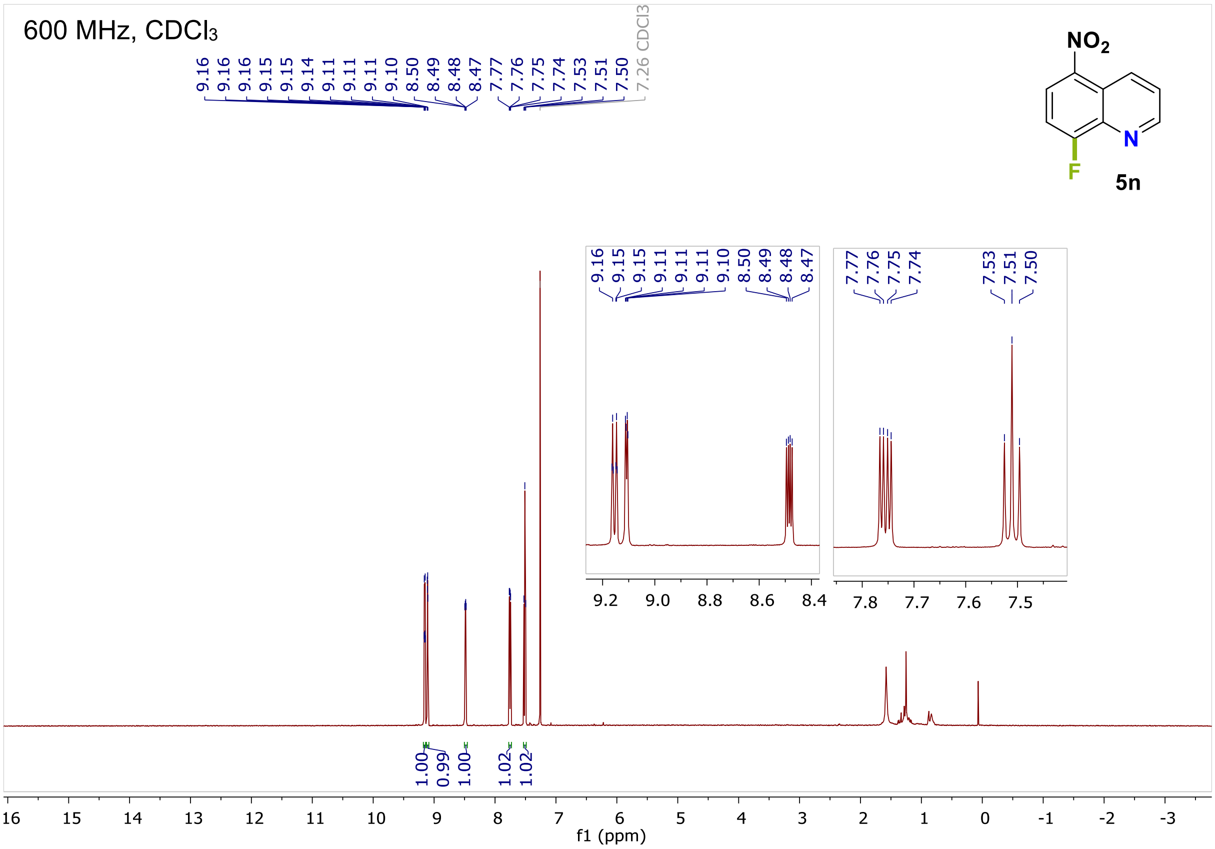

^{13}C NMR of 8-Fluoro-5-nitroquinoline (5n)

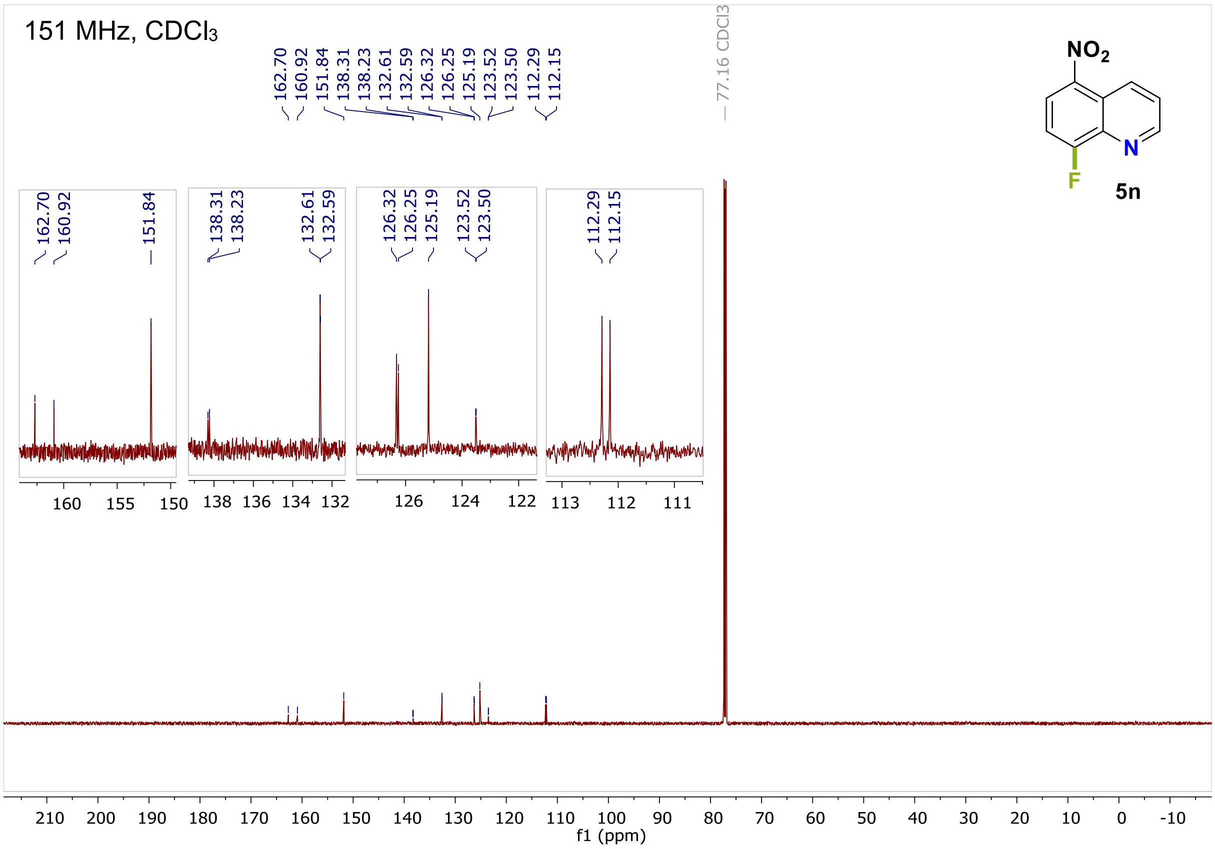

^{19}F NMR of 8-Fluoro-5-nitroquinoline (5n)

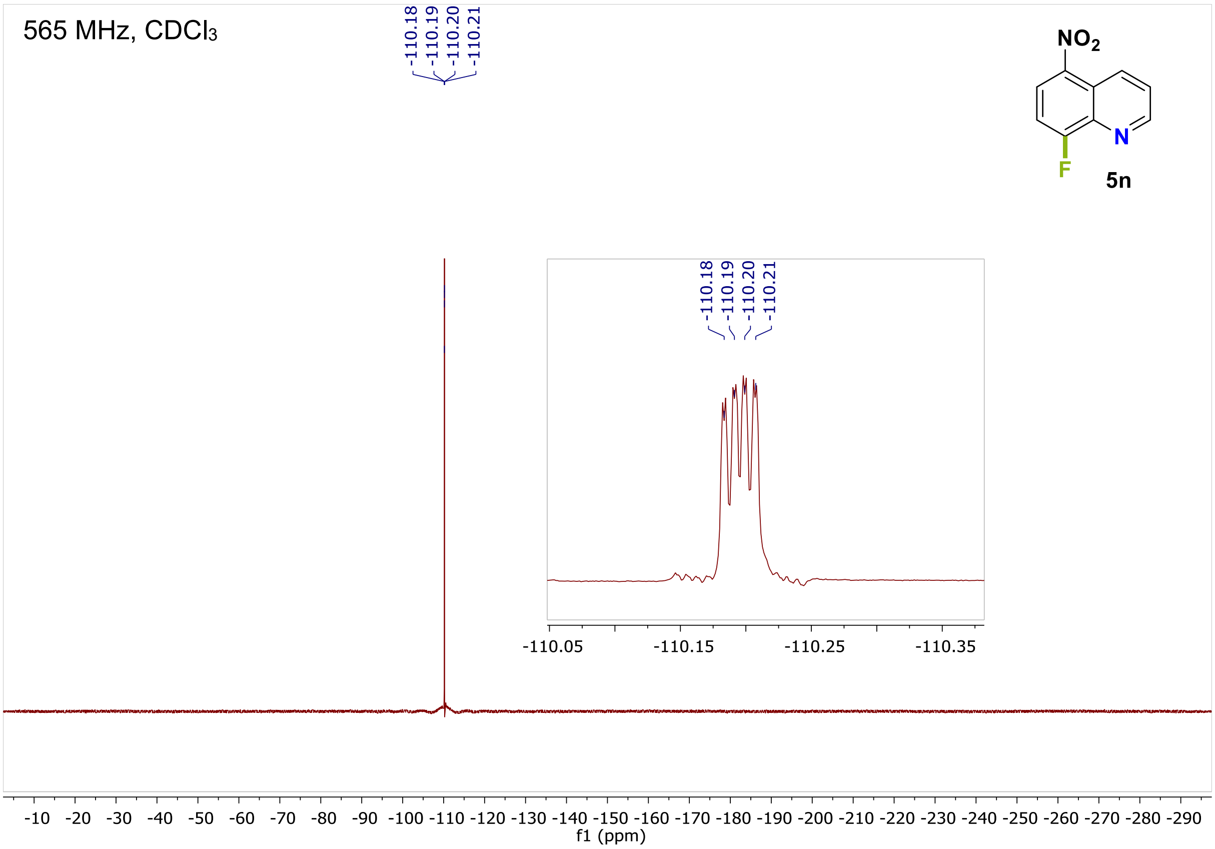

^{1}H NMR of 4-Fluoroazobenzene (5o)

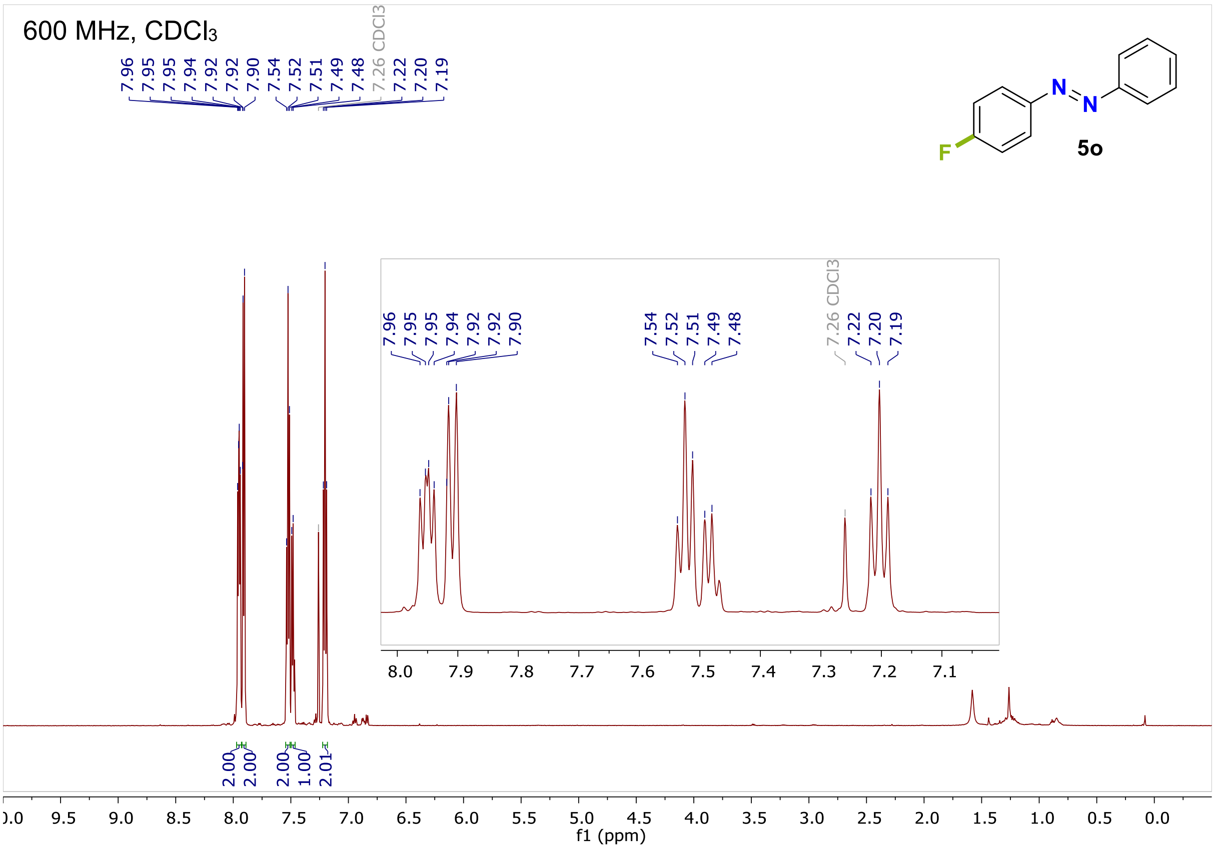

^{19}F NMR of 4-Fluoroazobenzene (5o)

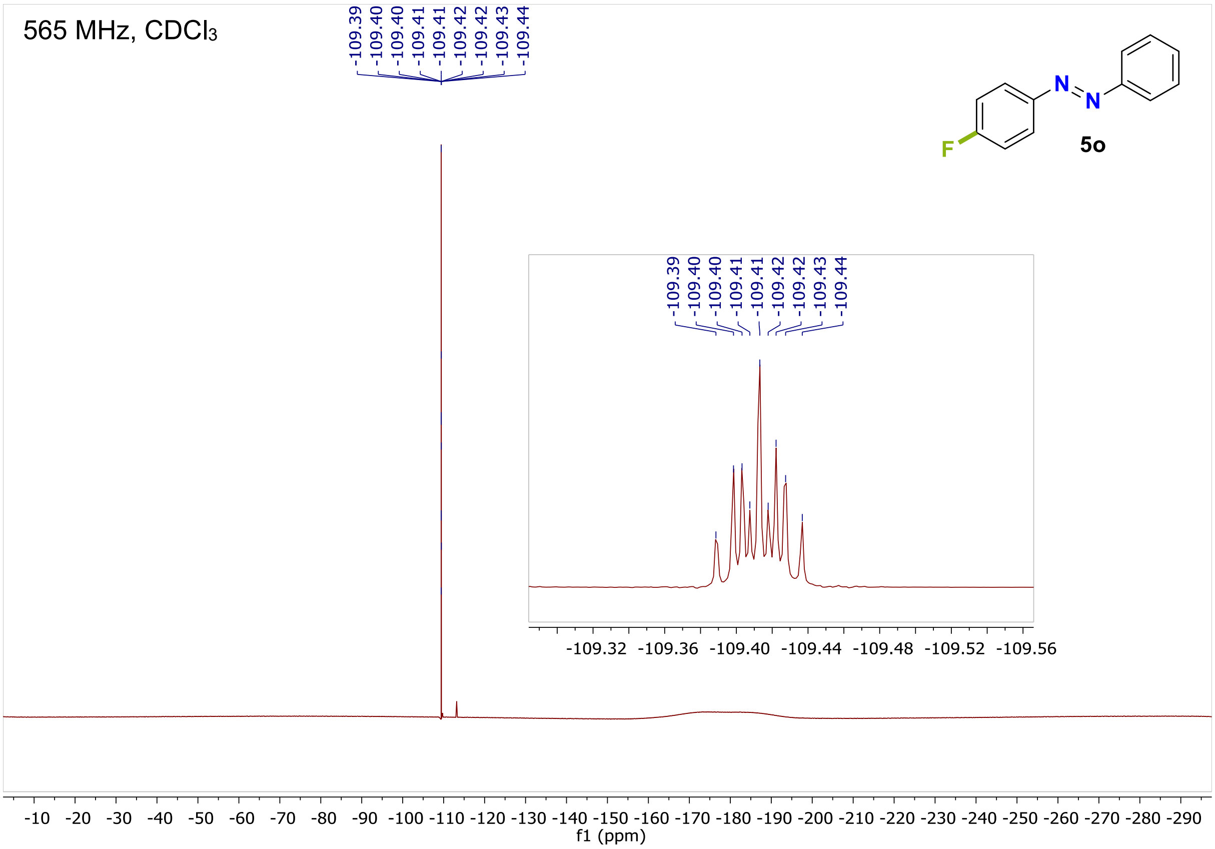

^{1}H NMR of 4-Fluoro-3-methoxybenzaldehyde (5p)

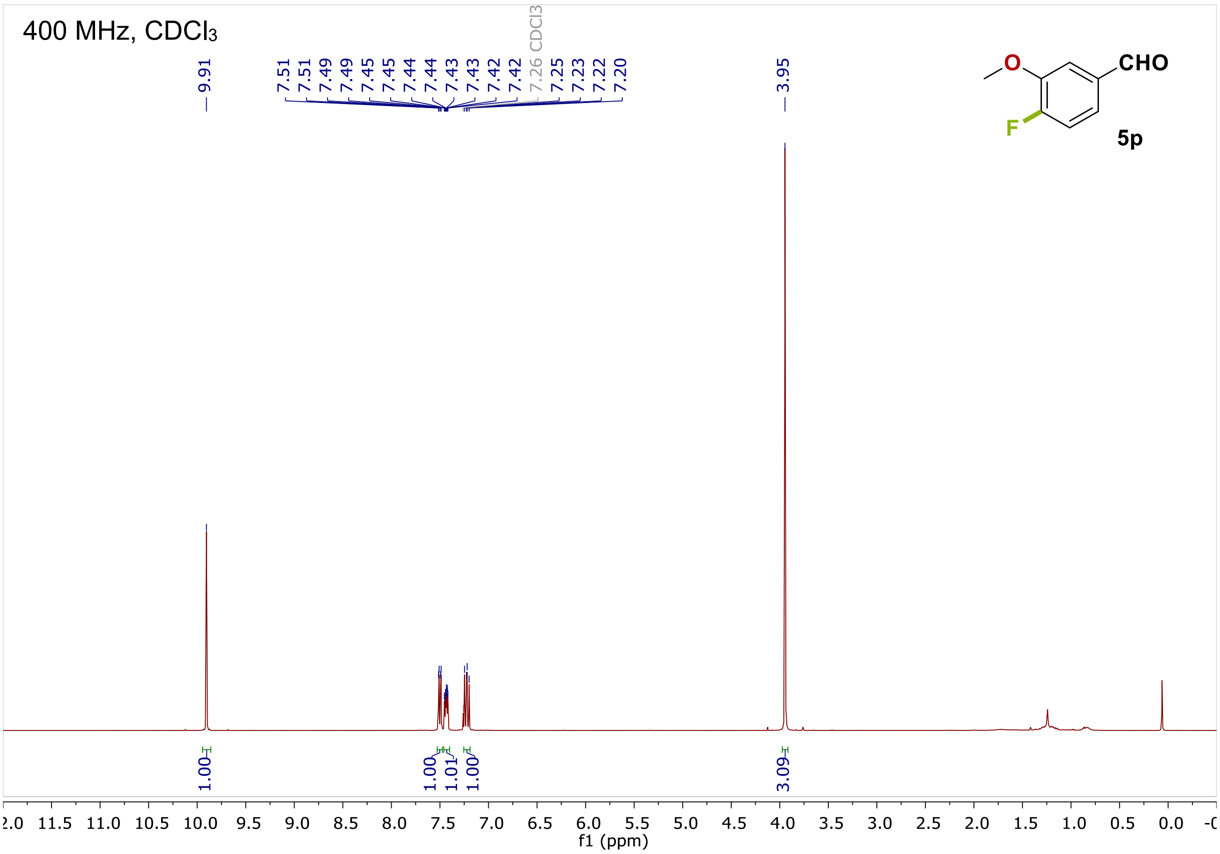

^{13}C NMR of 4-Fluoro-3-methoxybenzaldehyde (5p)

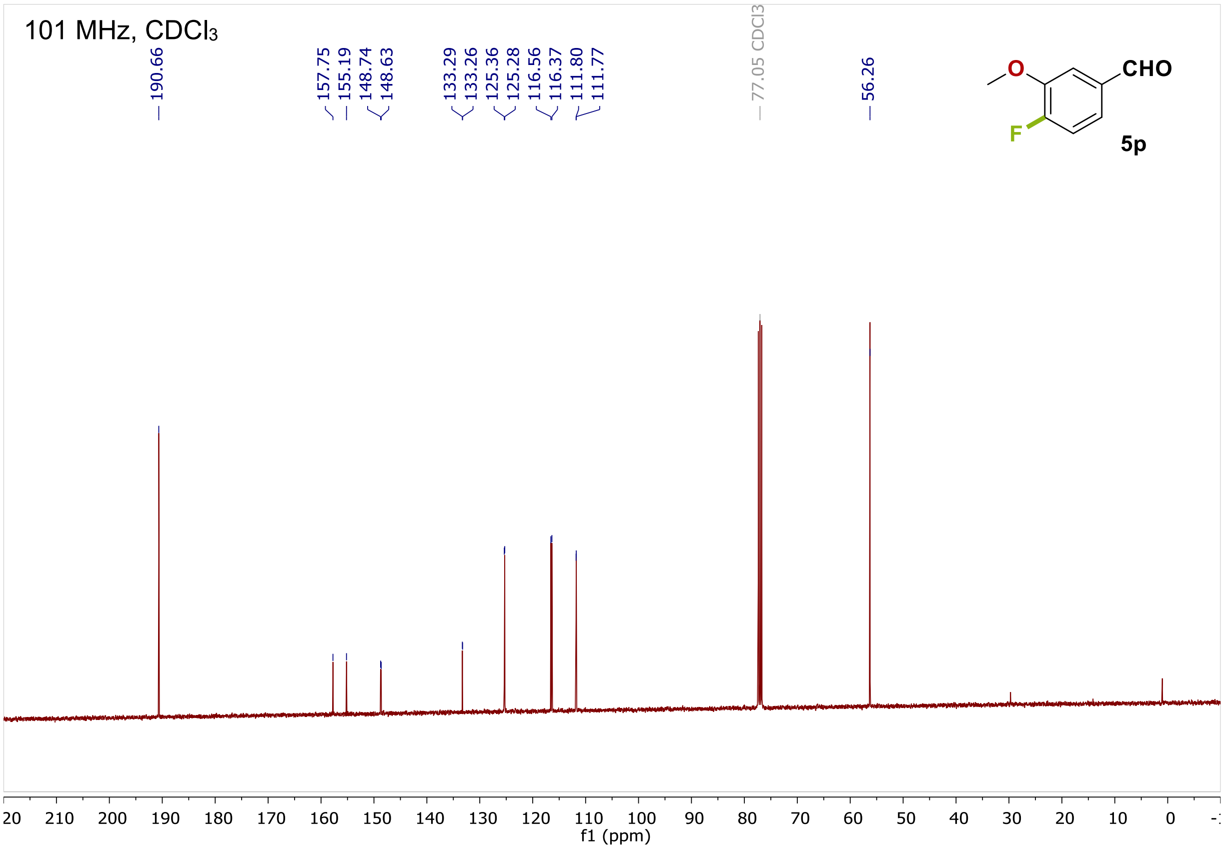

^{19}F NMR of 4-Fluoro-3-methoxybenzaldehyde (5p)

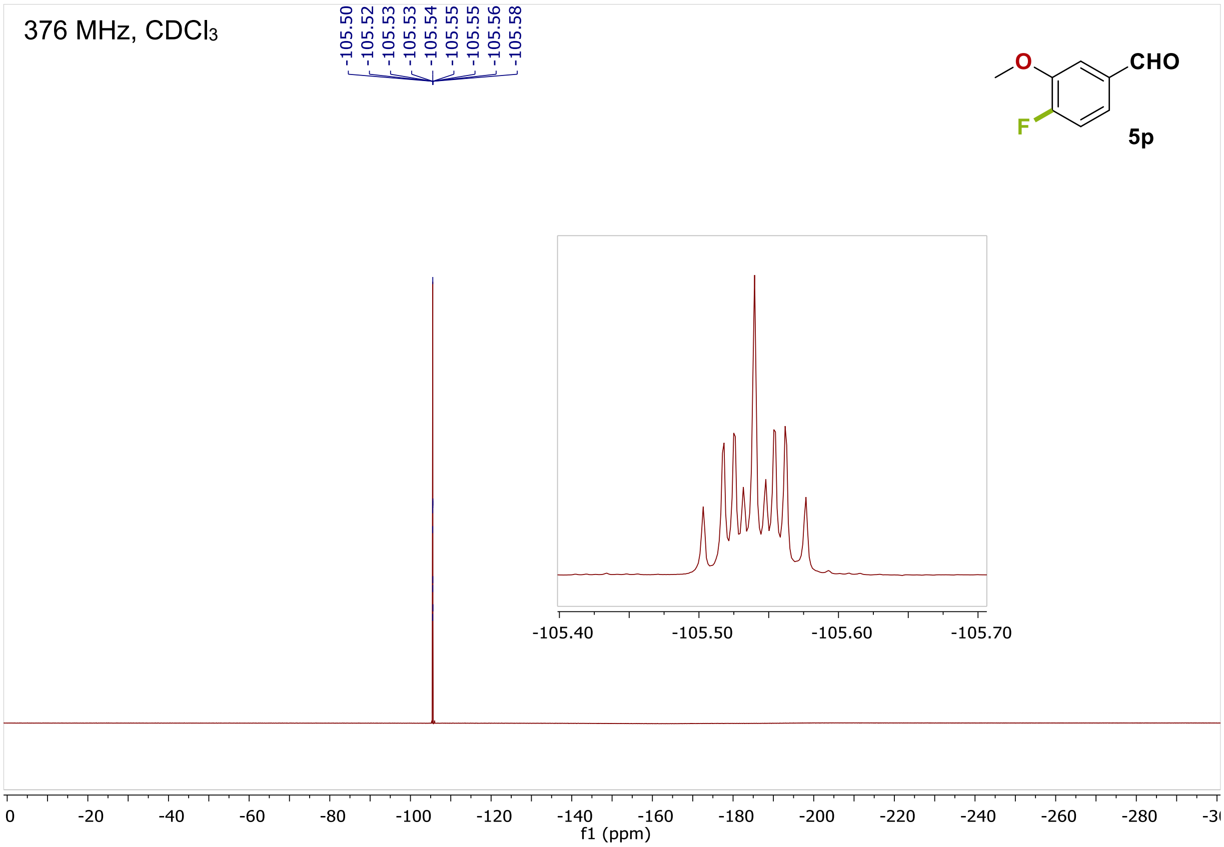

^{1}H NMR of 4,4'-Difluorobenzophenone (5q)

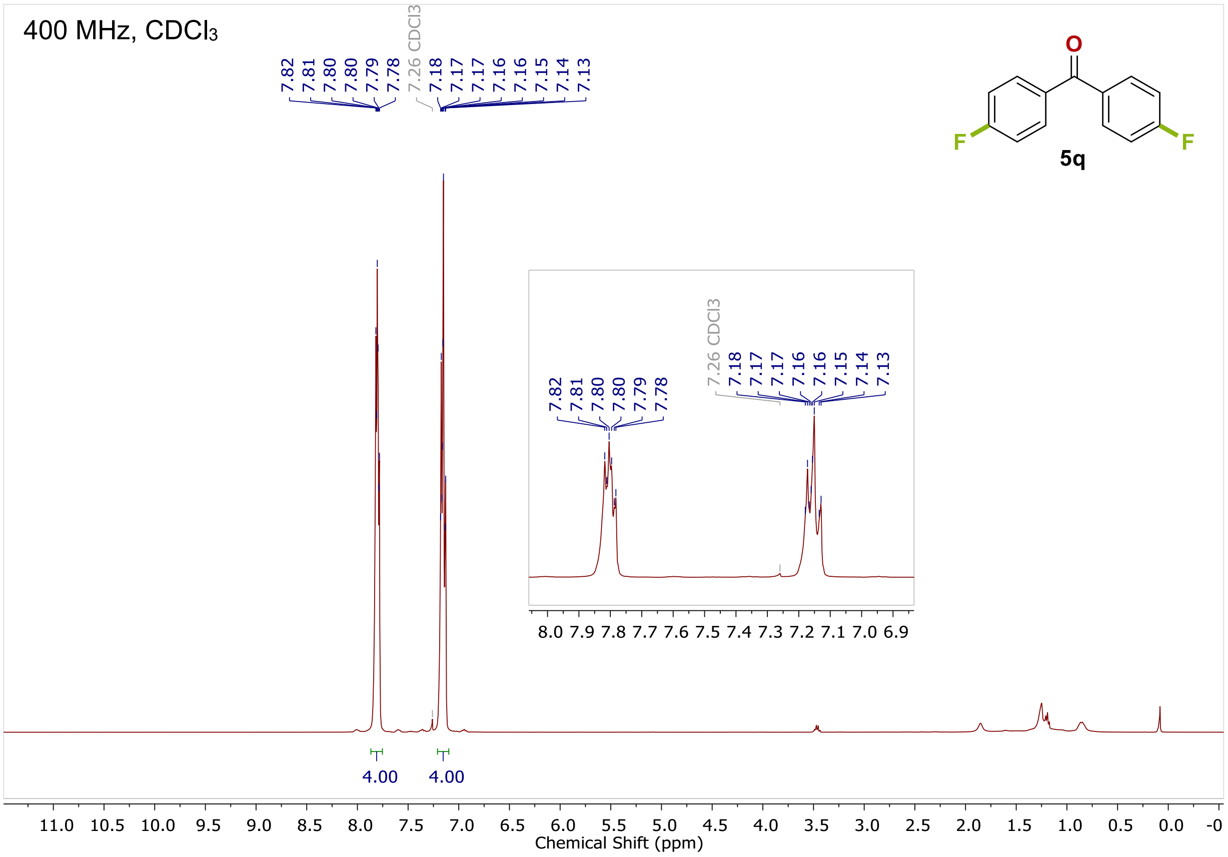

^{13}C NMR of 4,4'-Difluorobenzophenone (5q)

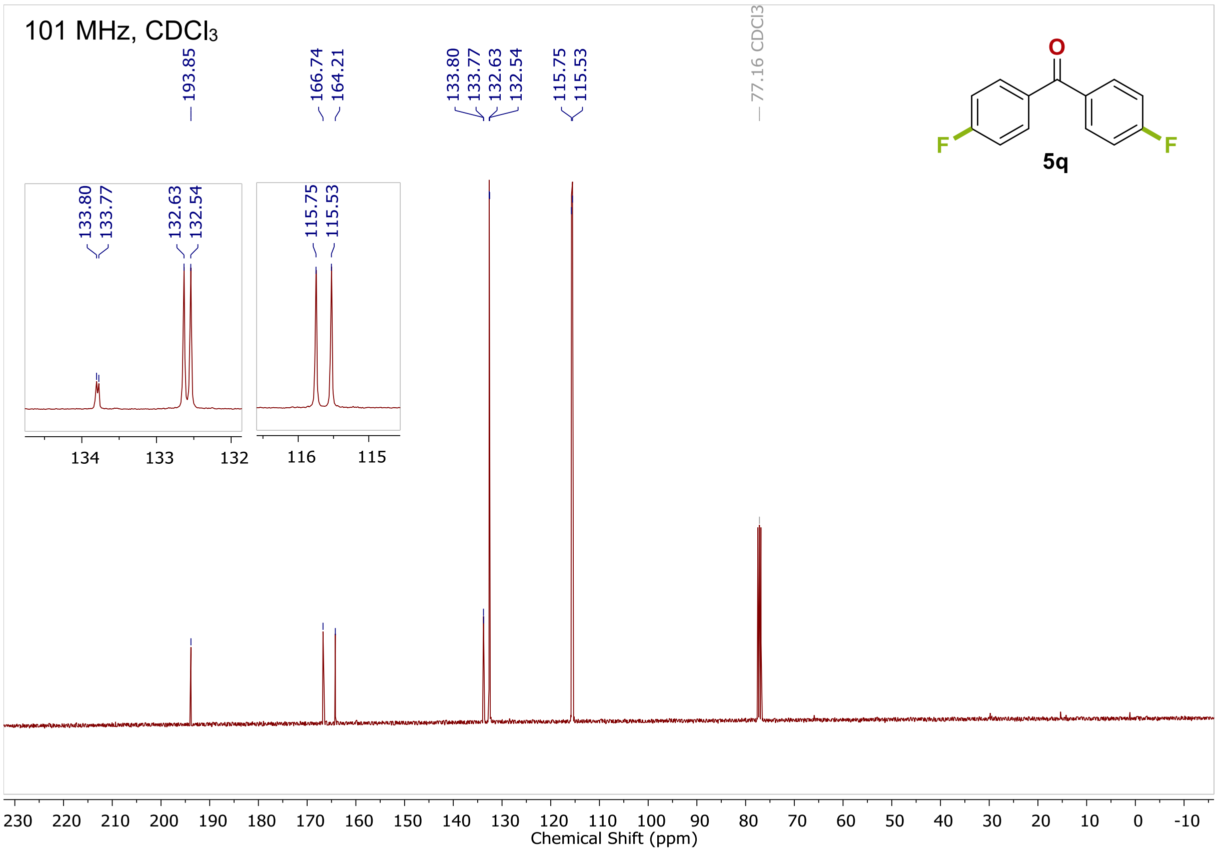

¹⁹F NMR of 4,4'-Difluorobenzophenone (5q)

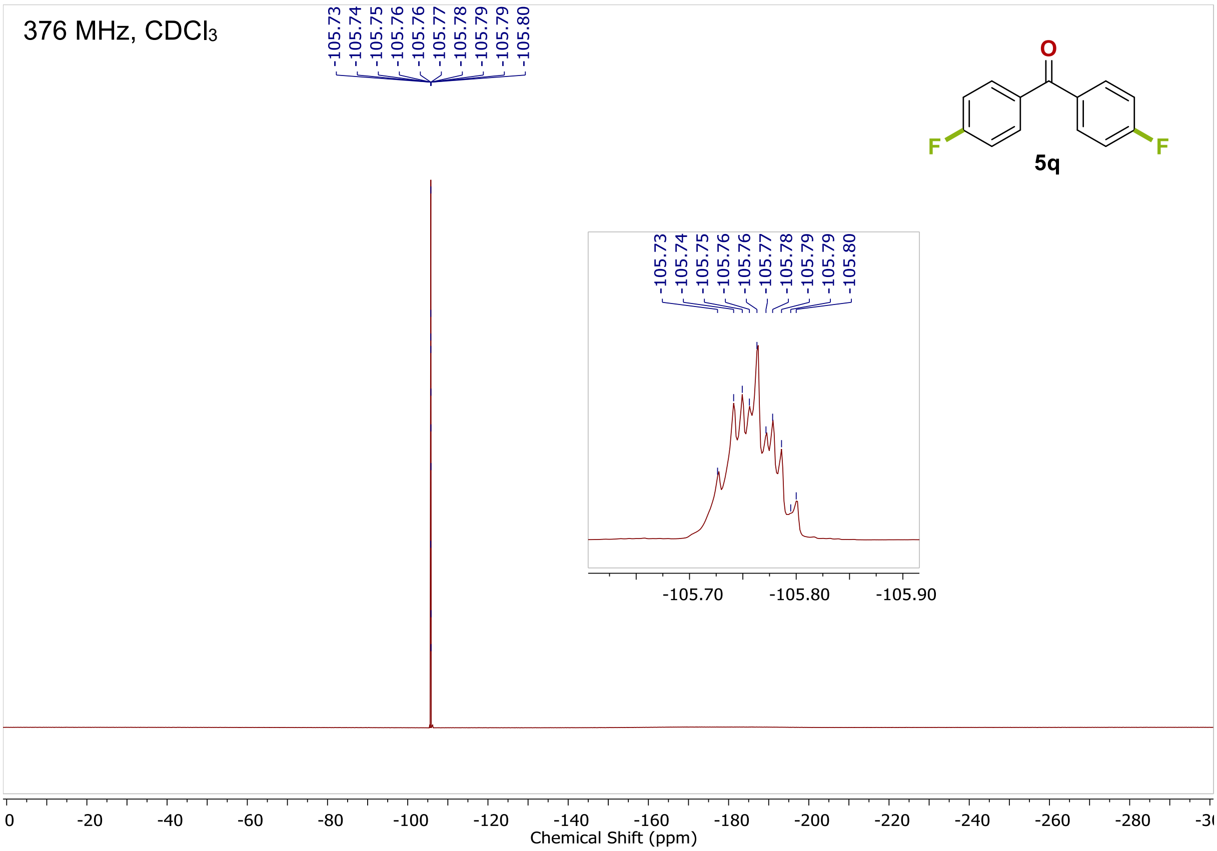

¹H NMR of Methyl 5-fluoronicotinate (5r)

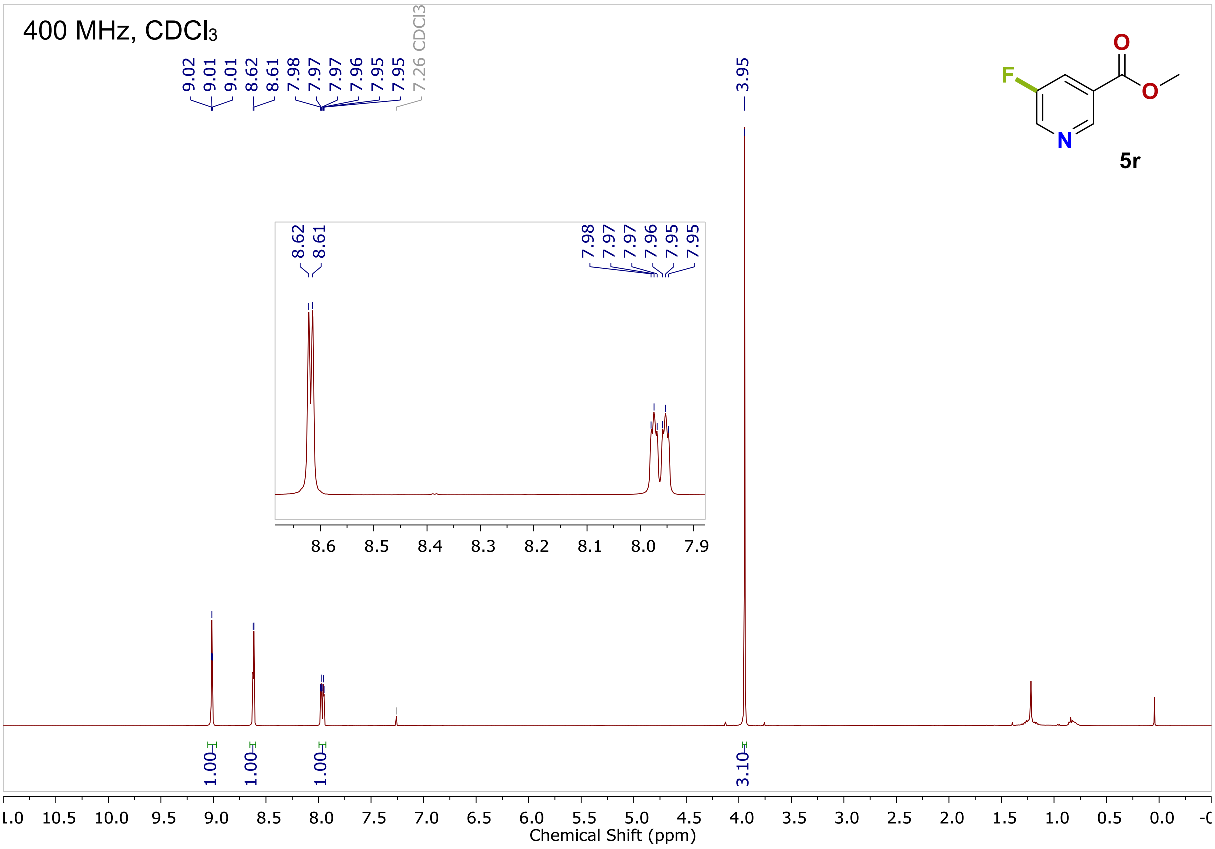

^{13}C NMR of Methyl 5-fluoronicotinate (5r)

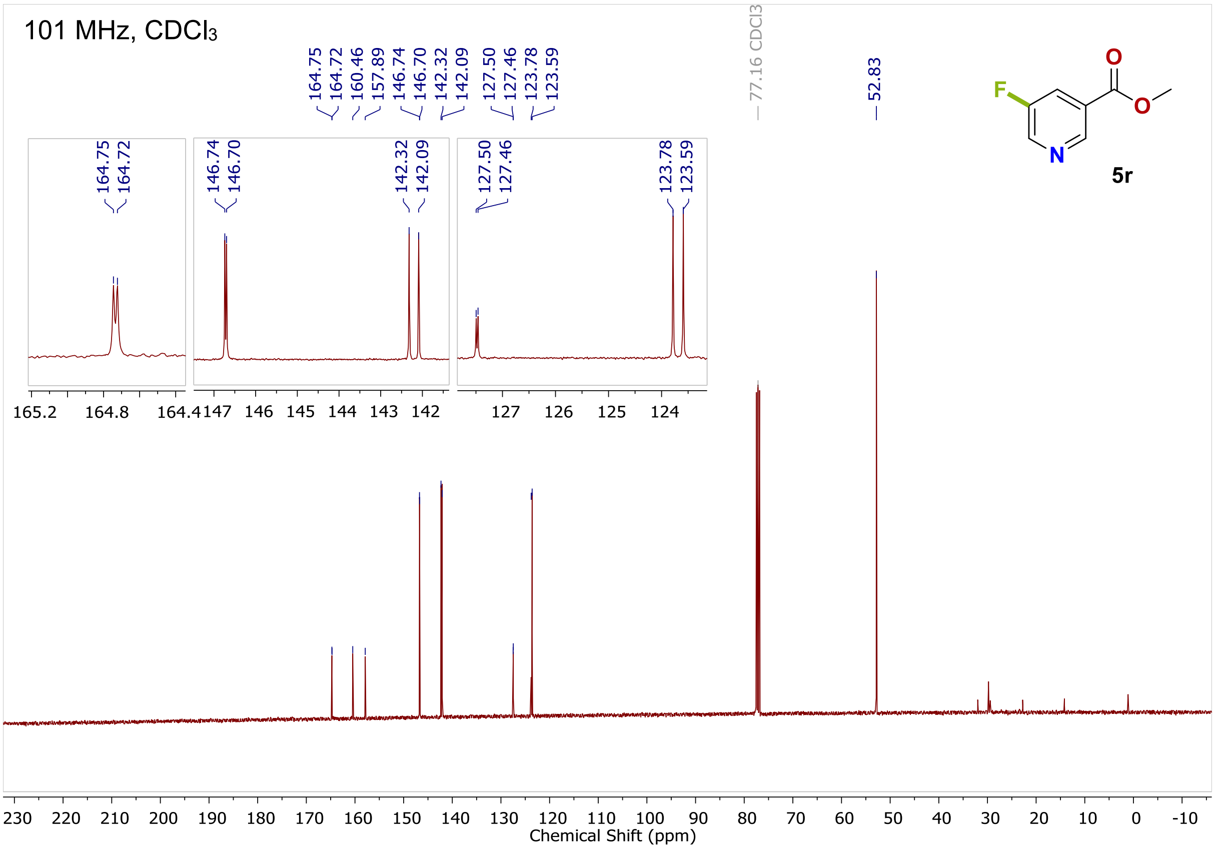

^{19}F NMR of Methyl 5-fluoronicotinate (5r)

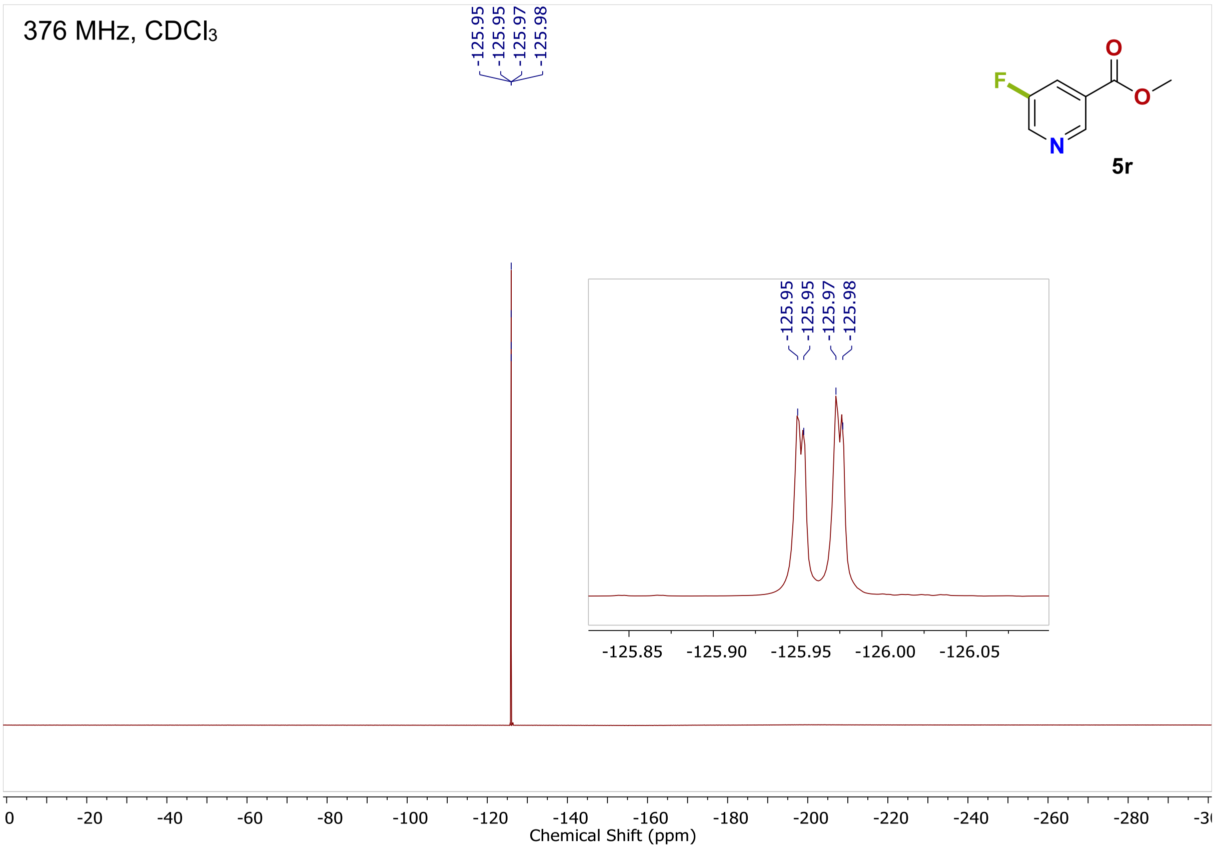

^{1}H NMR of Menthyl 6-fluoronicotinate (5s)

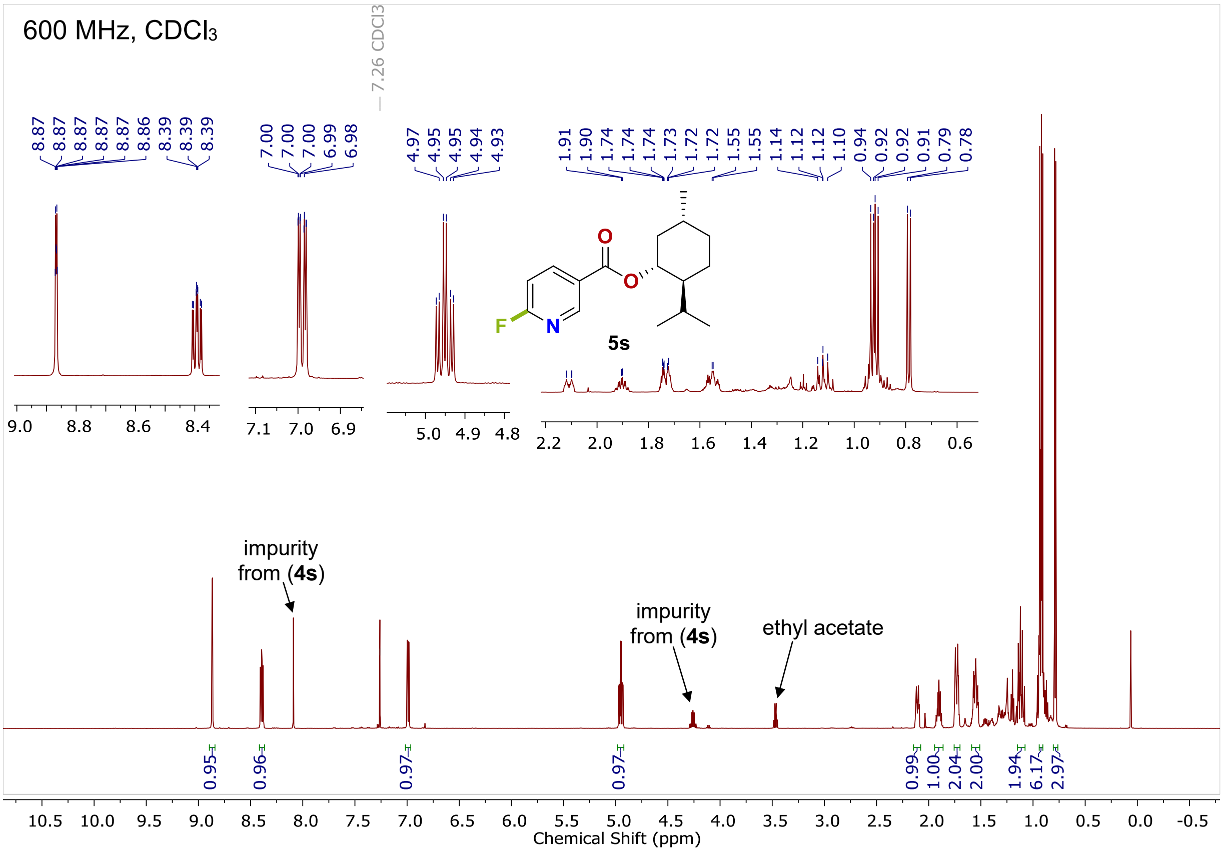

^{13}C NMR of Menthyl 6-fluoronicotinate (5s)

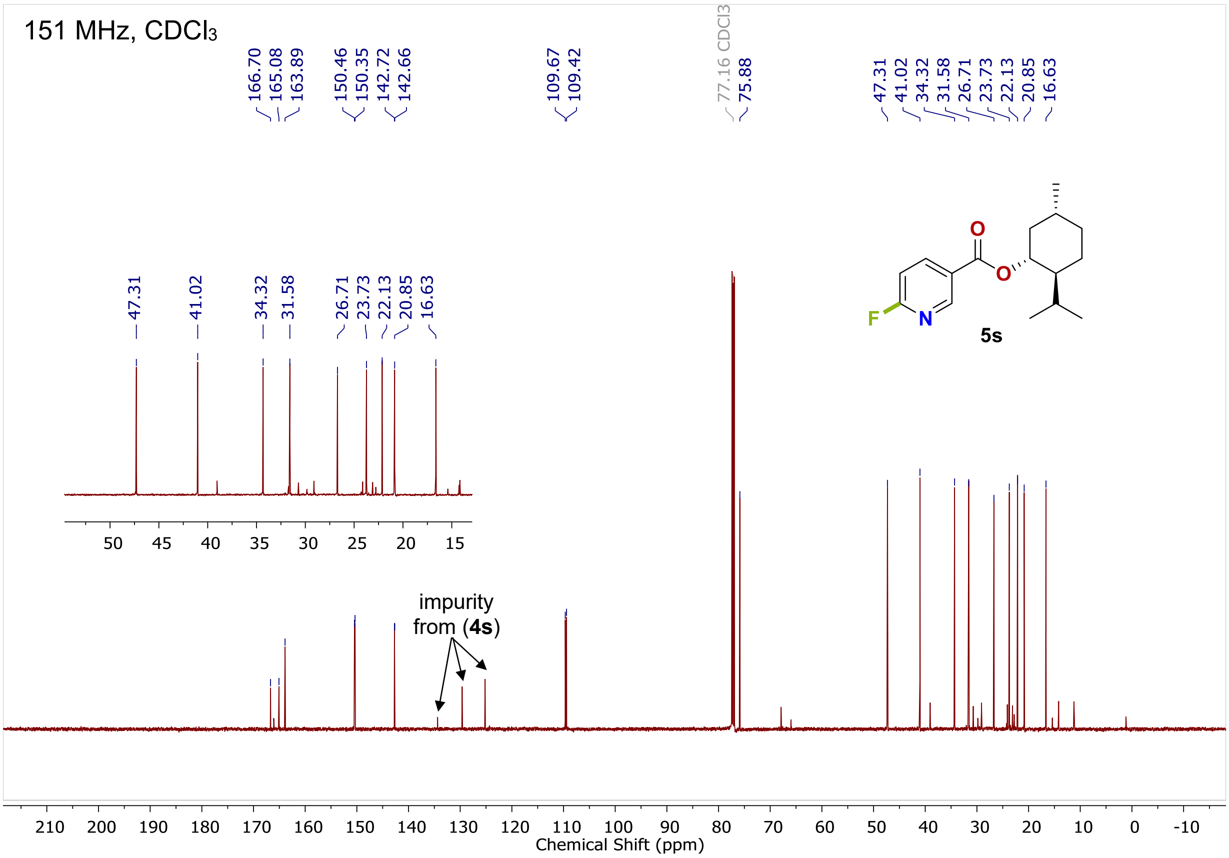

^{19}F NMR of Menthyl 6-fluoronicotinate (5s)

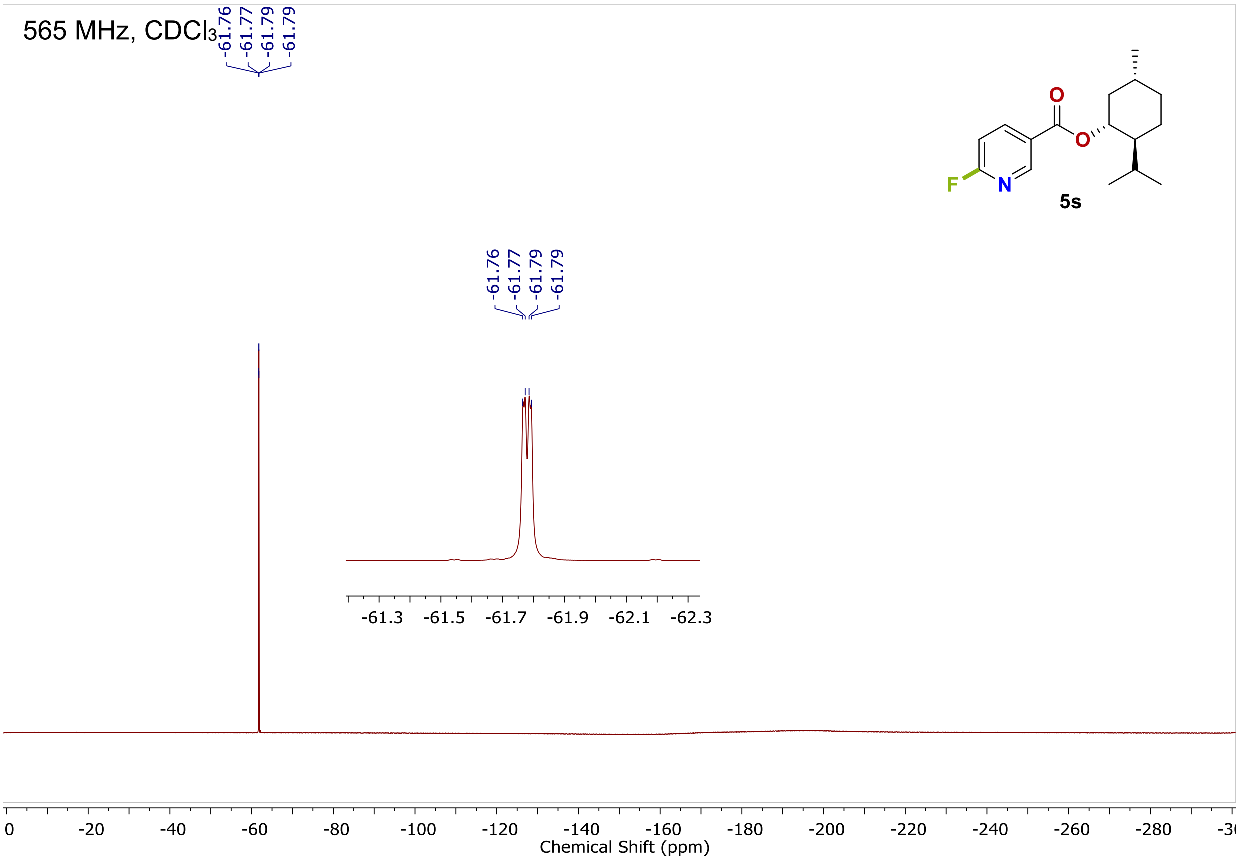

^{1}H NMR of 2-Butyl-3-(4-fluorobenzoyl)benzofuran (5t)

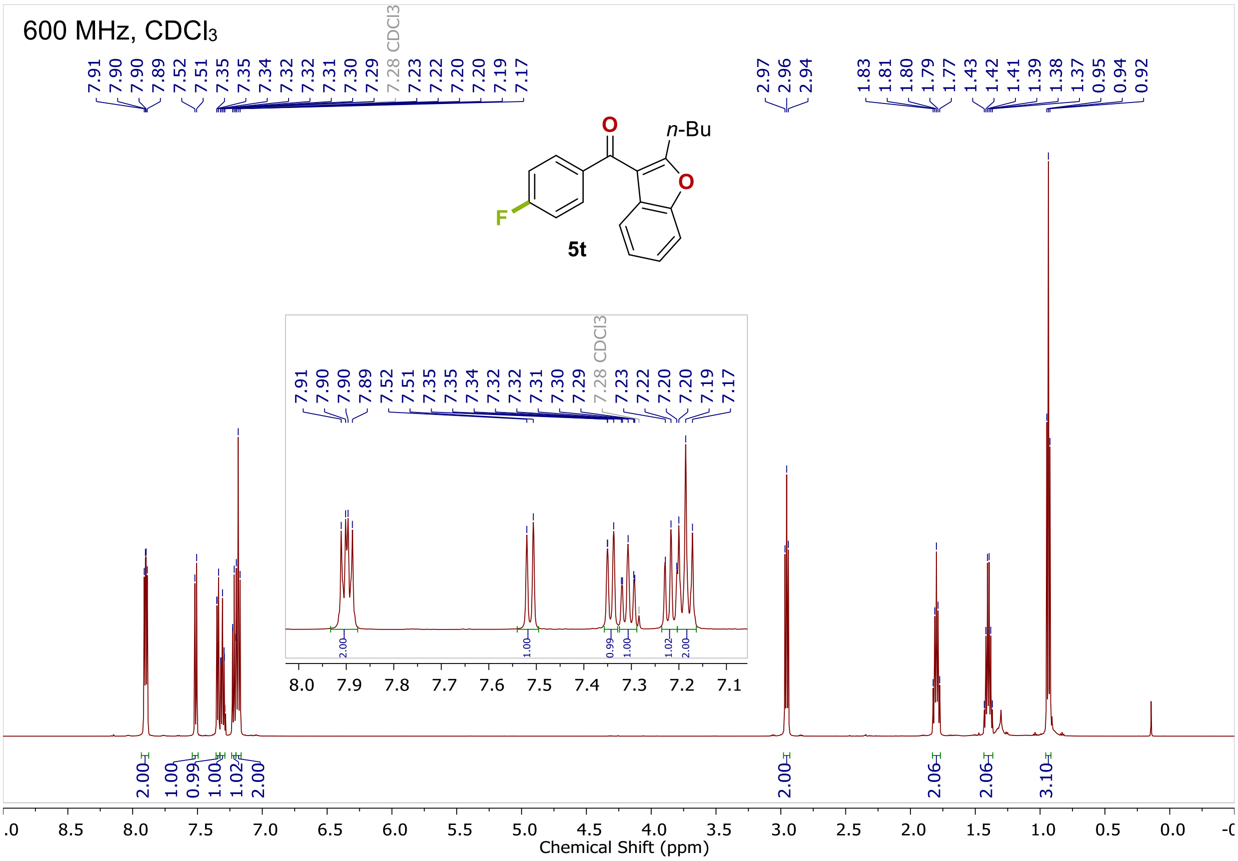

^{13}C NMR of 2-Butyl-3-(4-fluorobenzoyl)benzofuran (5t)

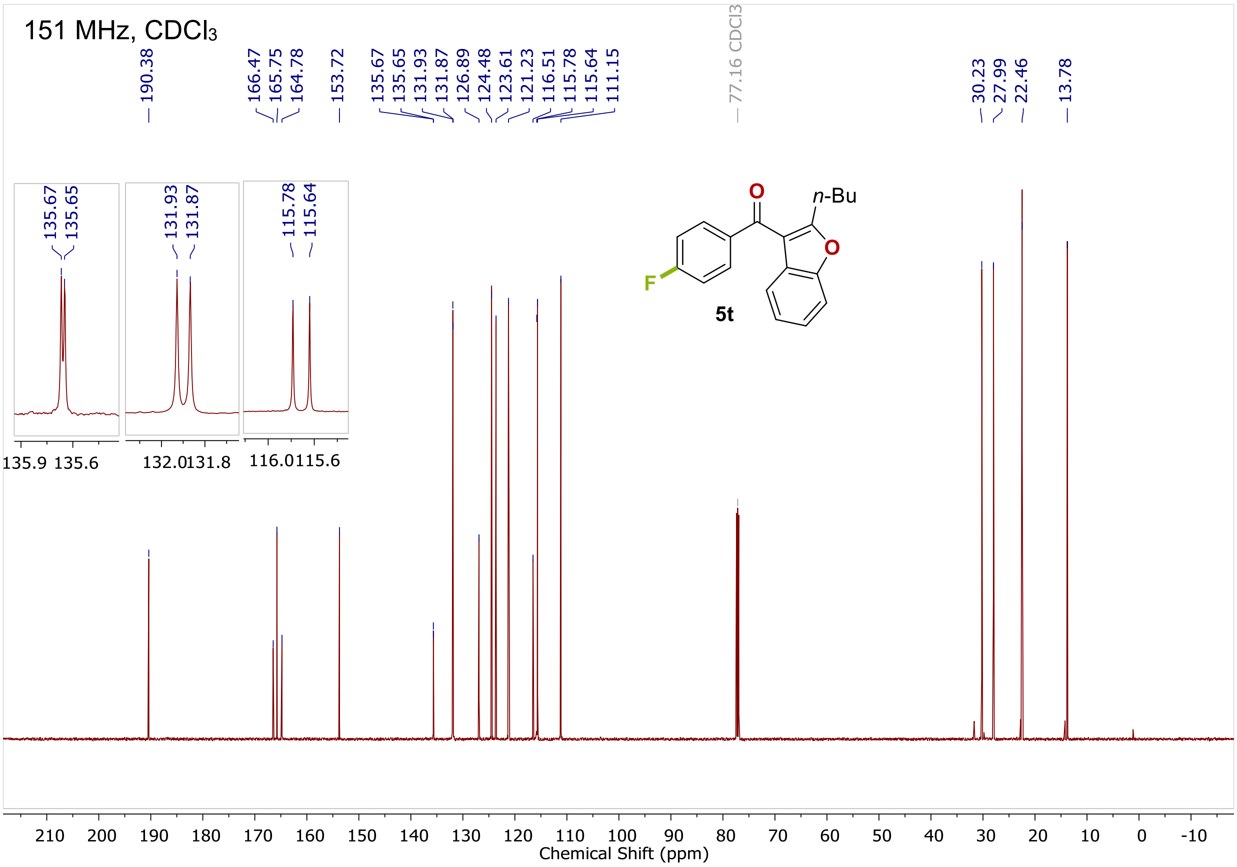

^{19}F NMR of 2-Butyl-3-(4-fluorobenzoyl)benzofuran (5t)

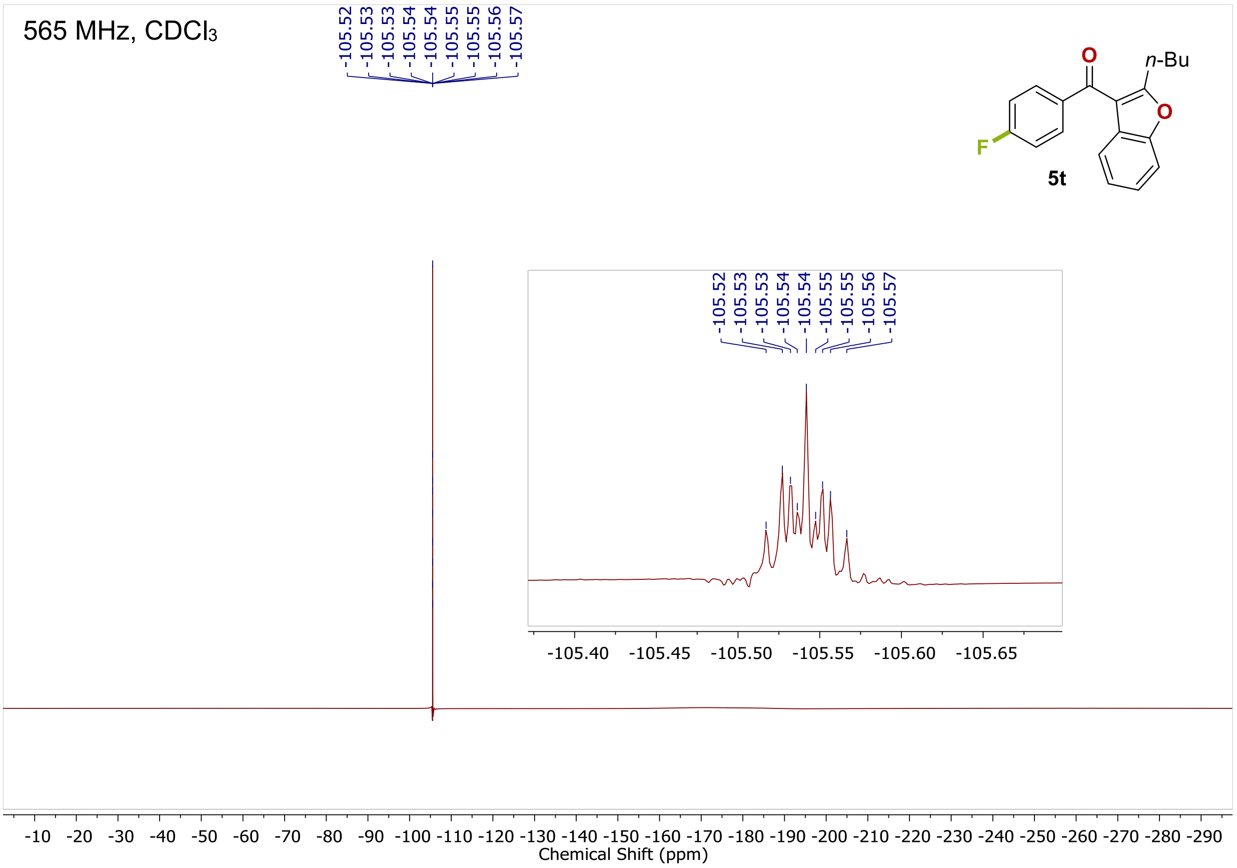

^{1}H NMR of 4-Fluoro(methylsulfonyl)benzene (5u)

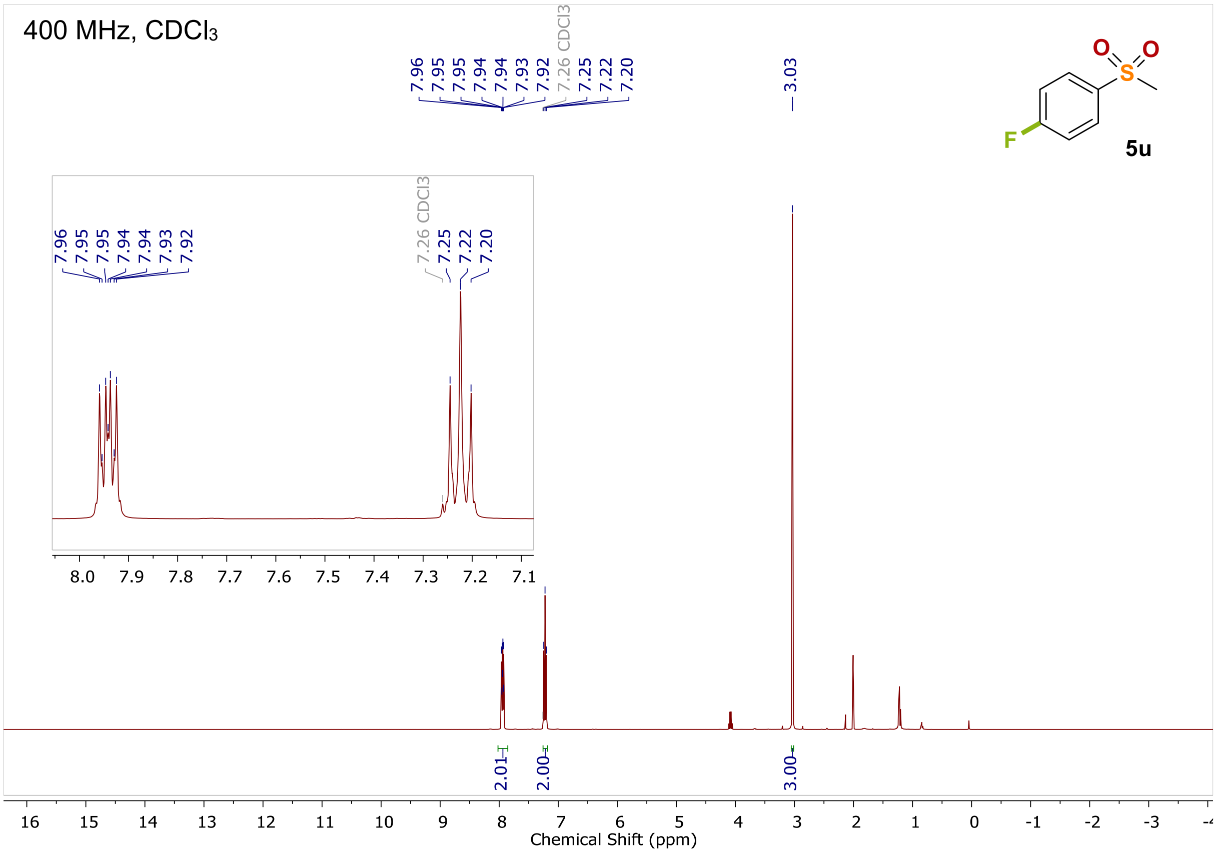

^{13}C NMR of 4-Fluoro(methylsulfonyl)benzene (5u)

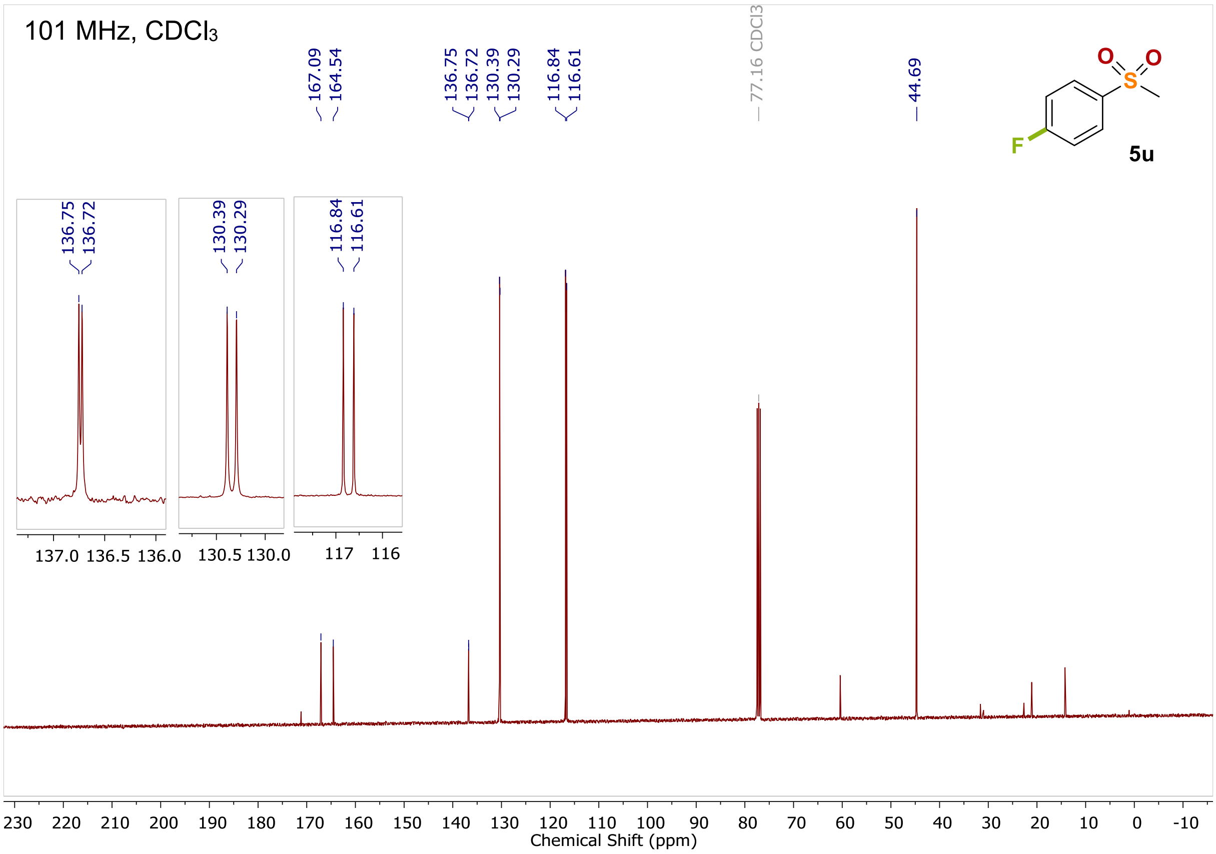

^{19}F NMR Spectra of 4-Fluoro(methylsulfonyl)benzene (5u)

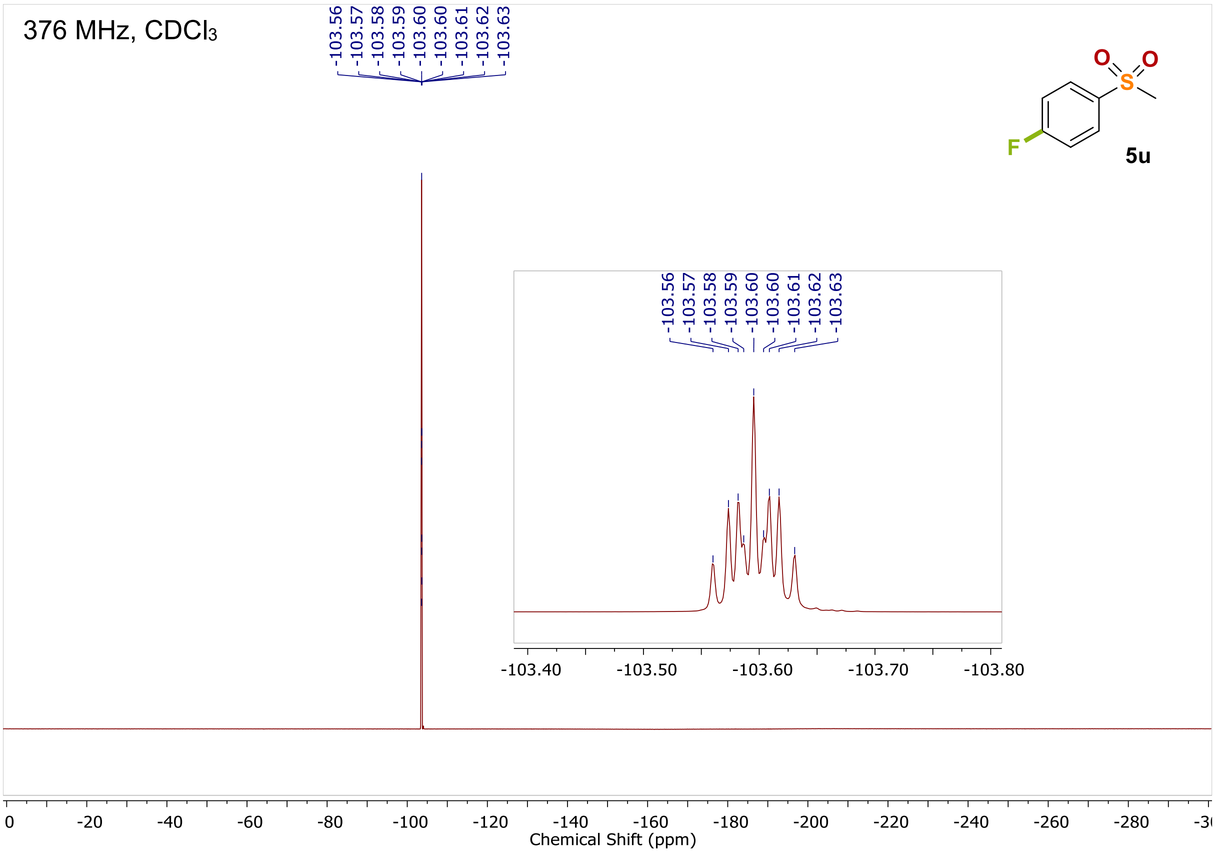

^{1}H NMR of 7-Fluoroflavone (5v)

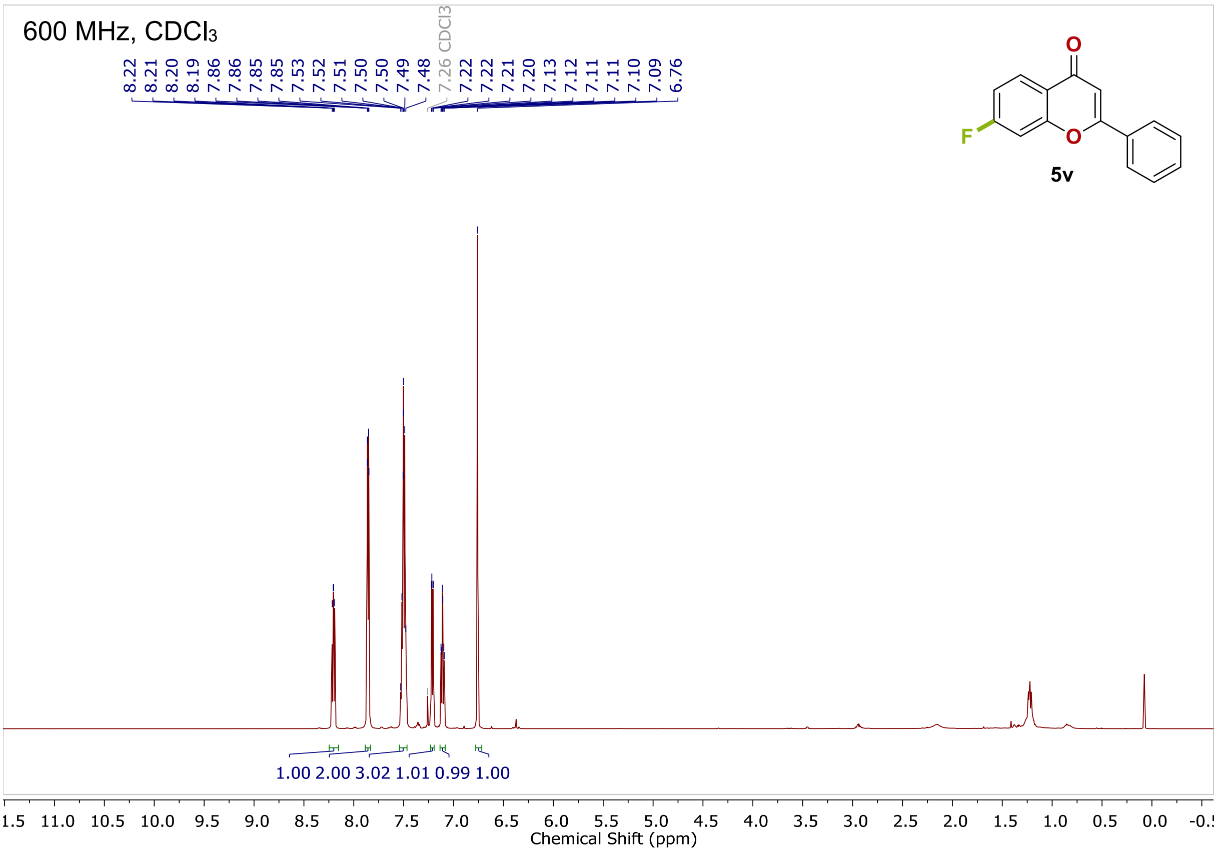

^{13}C NMR of 7-Fluoroflavone (5v)

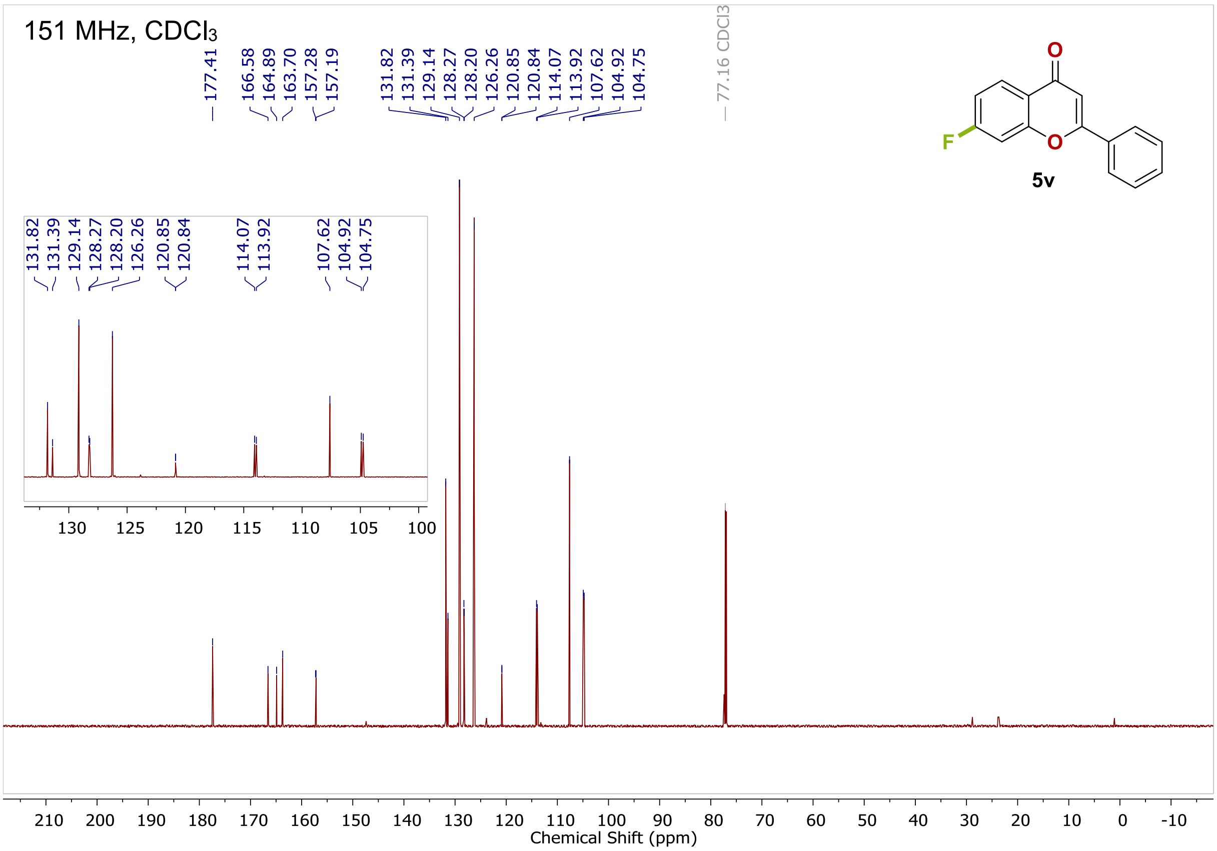

^{19}F NMR of 7-Fluoroflavone (5v)

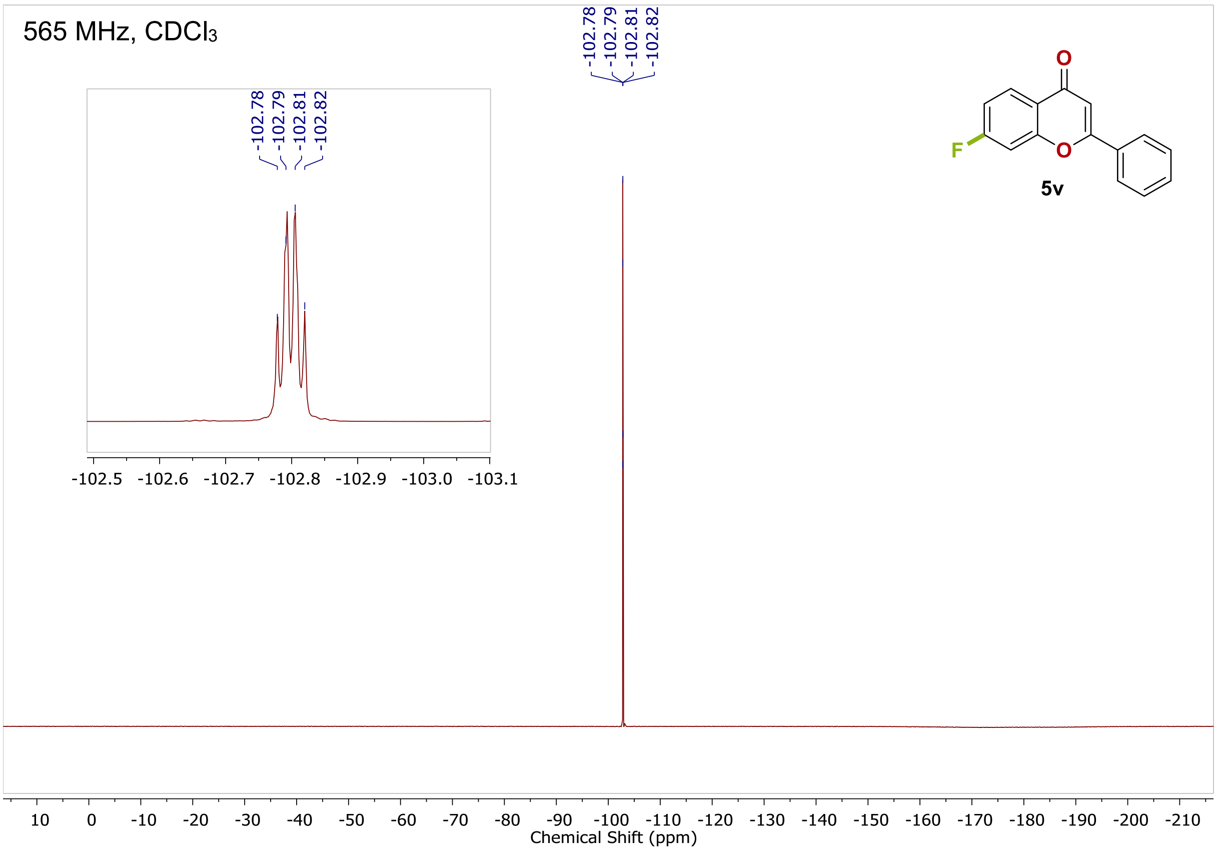

^{1}H NMR of Methyl 2-(6-fluoro-3-oxoxanthen-9-yl)benzoate (5w)

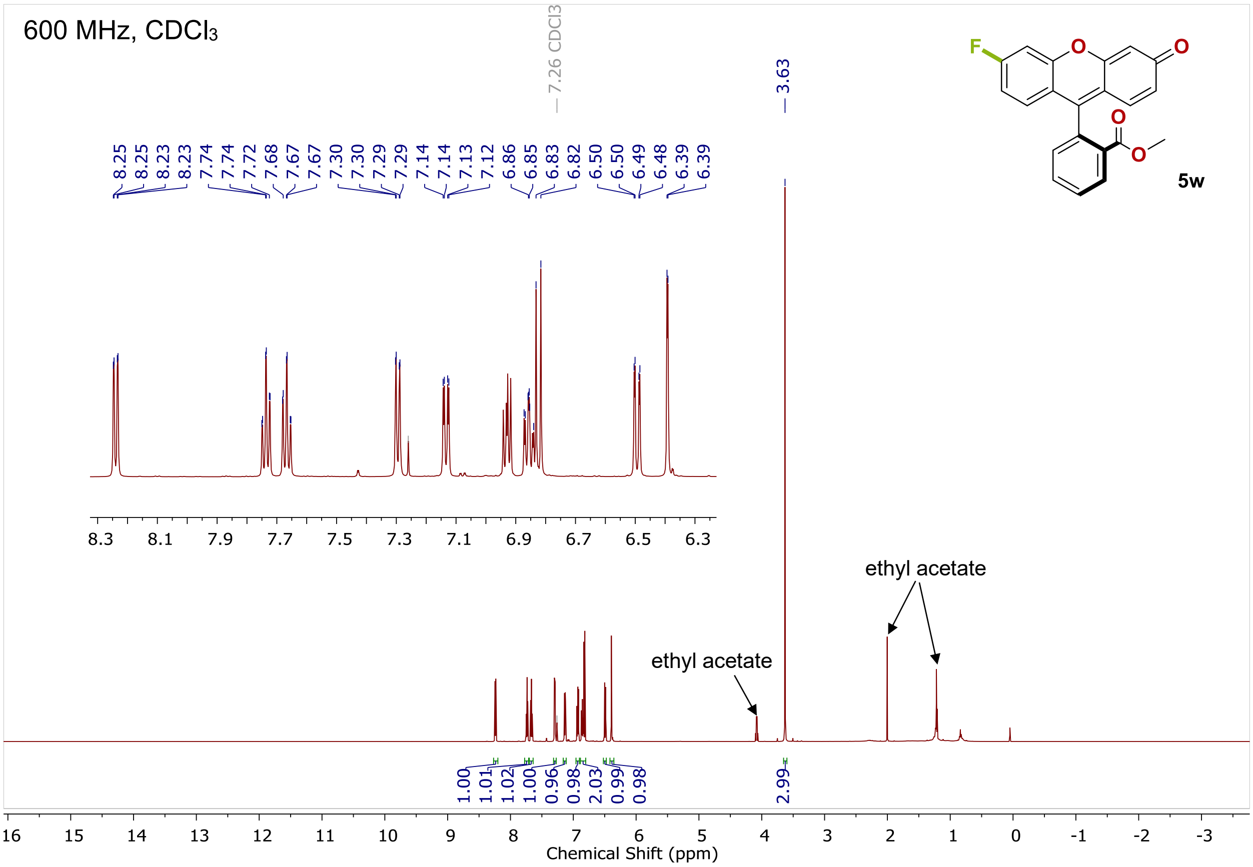

^{13}C NMR of Methyl 2-(6-fluoro-3-oxoxanthen-9-yl)benzoate (5w)

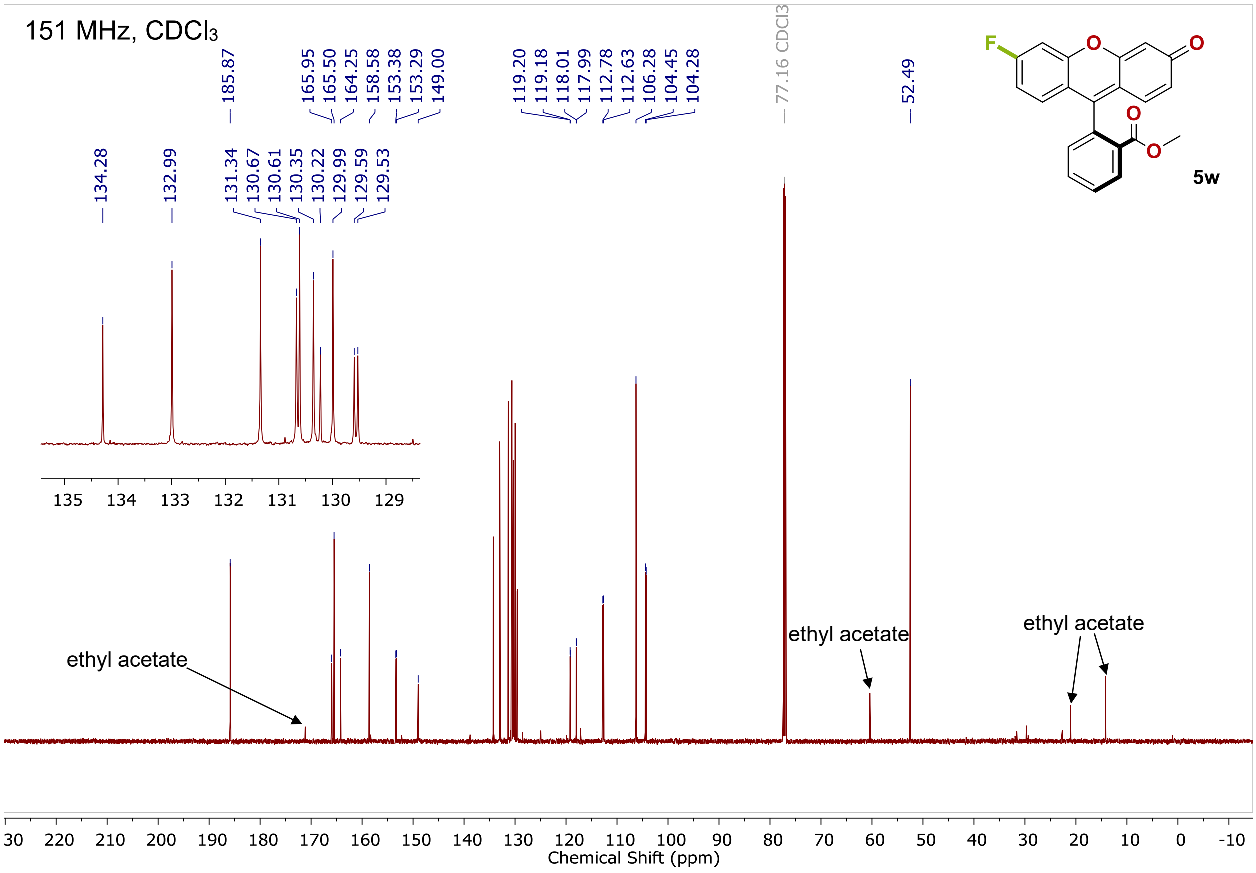

^{19}F NMR of Methyl 2-(6-fluoro-3-oxoxanthen-9-yl)benzoate (5w)

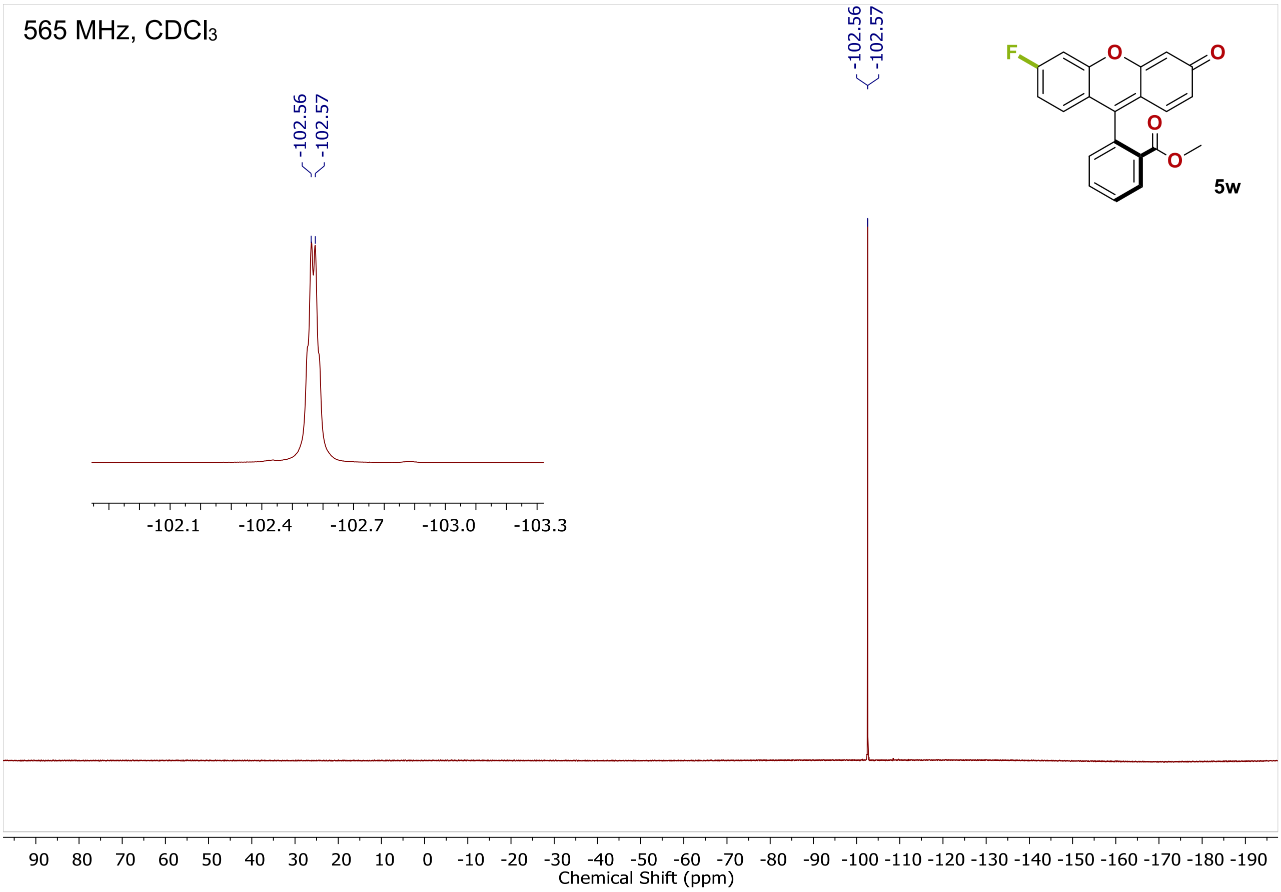

S17 References

(1) Fujimoto, T.; Ritter, T. PhenoFluorMix: Practical Chemoselective Deoxyfluorination of Phenols. *Org. Lett.* **2015**, *17* (3), 544–547.

(2) Magano, J.; Chen, M. H.; Clark, J. D.; Nussbaumer, T. 2-(Diethylamino)Ethanethiol, a New Reagent for the Odorless Deprotection of Aromatic Methyl Ethers. *J. Org. Chem.* **2006**, *71* (18), 7103–7105.

(3) Park, J. K.; Lackey, H. H.; Ondrusek, B. A.; McQuade, D. T. Stereoconvergent Synthesis of Chiral Allylboronates from an *E* / *Z* Mixture of Allylic Aryl Ethers Using a 6-NHC−Cu(I) Catalyst. *J. Am. Chem. Soc.* **2011**, *133* (8), 2410–2413.

(4) Spink, E.; Ding, D.; Peng, Z.; Boudreau, M. A.; Leemans, E.; Lastochkin, E.; Song, W.; Lichtenwalter, K.; O'Daniel, P. I.; Testero, S. A.; Pi, H.; Schroeder, V. A.; Wolter, W. R.; Antunes, N. T.; Suckow, M. A.; Vakulenko, S.; Chang, M.; Mobashery, S. Structure–Activity Relationship for the Oxadiazole Class of Antibiotics. *J. Med. Chem.* **2015**, *58* (3), 1380–1389.

(5) Jereb, M. Highly Atom Economical Uncatalysed and I2-Catalysed Silylation of Phenols, Alcohols and Carbohydrates, Using HMDS under Solvent-Free Reaction Conditions (SFRC). *Tetrahedron* **2012**, *68* (20), 3861–3867.

(6) Curini, M.; Epifano, F.; Marcotullio, M. C.; Rosati, O.; Costantino, U. Heterogeneous Catalysis in Trimethylsilylation of Alcohols and Phenols by Zirconium Sulfophenyl Phosphonate. *Synth. Commun.* **1999**, *29* (3), 541–546.

(7) Tang, P.; Wang, W.; Ritter, T. Deoxyfluorination of Phenols. *J. Am. Chem. Soc.* **2011**, *133* (30), 11482–11484.

(8) Wise, D. E.; Gogarnoiu, E. S.; Duke, A. D.; Paolillo, J. M.; Vacala, T. L.; Hussain, W. A.; Parasram, M. Photoinduced Oxygen Transfer Using Nitroarenes for the Anaerobic Cleavage of Alkenes. *J. Am. Chem. Soc.* **2022**, *144* (34), 15437–15442.

(9) Lacour, M.-A.; Zablocka, M.; Duhayon, C.; Majoral, J.-P.; Taillefer, M. Efficient Phosphorus Catalysts for the Halogen-Exchange (Halex) Reaction. *Adv. Synth. Catal.* **2008**, *350* (17), 2677–2682.

(10) Anbarasan, P.; Neumann, H.; Beller, M. A Novel and Convenient Synthesis of Benzonitriles: Electrophilic Cyanation of Aryl and Heteroaryl Bromides. *Chem. Eur. J.* **2011**, *17* (15), 4217–4222.

(11) Moorthy, J. N.; Singhal, N.; Senapati, K. Oxidative Cleavage of Vicinal Diols: IBX Can Do What Dess–Martin Periodinane (DMP) Can. *Org. Biomol. Chem.* **2007**, *5* (5), 767–771.

(12) Nestl, B. M.; Glueck, S. M.; Hall, M.; Kroutil, W.; Stuermer, R.; Hauer, B.; Faber, K. Biocatalytic Racemization of (Hetero)Aryl-Aliphatic α-Hydroxycarboxylic Acids ByLactobacillus Spp. Proceeds via an Oxidation–Reduction Sequence. *European J. Org. Chem.* **2006**, *2006* (20), 4573–4577.

(13) Fang, F.; Li, Y.; Tian, S.-K. Stereoselective Olefination of N-Sulfonyl Imines with Stabilized Phosphonium Ylides for the Synthesis of Electron-Deficient Alkenes. *European J. Org. Chem.* **2011**, *2011* (6), 1084–1091.

(14) Schmink, J. R.; Holcomb, J. L.; Leadbeater, N. E. Testing the Validity of Microwave-Interfaced, in Situ Raman Spectroscopy as a Tool for Kinetic Studies. *Org. Lett.* **2009**, *11* (2), 365–368.

(15) Zhang, K.; Budinská, A.; Passera, A.; Katayev, D. *N* -Nitroheterocycles: Bench-Stable Organic Reagents for Catalytic *Ipso* -Nitration of Aryl- and Heteroarylboronic Acids. *Org. Lett.* **2020**, *22* (7), 2714–2719.

(16) Fu, Z.; Jiang, L.; Zuo, Q.; Li, Z.; Liu, Y.; Wei, Z.; Cai, H. Inexpensive NaX (X = I, Br, Cl) as a Halogen Donor in the Practical Ag/Cu-Mediated Decarboxylative Halogenation of Aryl Carboxylic Acids under Aerobic Conditions. *Org. Biomol. Chem.* **2018**, *16* (30), 5416–5421.

(17) Holling, D.; Sandford, G.; Batsanov, A. S.; Yufit, D. S.; Howard, J. A. K. Direct Fluorination of Coumarin, 6-Methyl-Coumarin and 7-Alkoxy-Coumarins. *J. Fluor. Chem.* **2005**, *126* (9–10), 1377–1383.

(18) Xiaohu Zhang. Heteroaryl compounds as cxcr4 inhibitors, composition and method using the same, **2019**, *patent*: WO2019060860A1.

(19) Griwatz, J. H.; Kunz, A.; Wegner, H. A. Continuous Flow Synthesis of Azobenzenes via Baeyer–Mills Reaction. *Beilstein J. Org. Chem.* **2022**, *18*, 781–787.

(20) Tay, N. E. S.; Chen, W.; Levens, A.; Pistritto, V. A.; Huang, Z.; Wu, Z.; Li, Z.; Nicewicz, D. A. 19F- and 18F-Arene Deoxyfluorination via Organic Photoredox-Catalysed Polarity-Reversed Nucleophilic Aromatic Substitution. *Nat. Catal.* **2020**, *3* (9), 734–742.

(21) Rao, M. L. N.; Venkatesh, V.; Dasgupta, P. Oxalyl Chloride as Carbonyl Synthon in Pd-Catalyzed Carbonylations of Triarylbismuth and Triarylindium Organometallic Nucleophiles. *Tetrahedron. Lett.* **2010**, *51* (38), 4975–4980.

(22) Kyba, E. P.; Liu, S. T.; Chockalingam, K.; Reddy, B. R. A General Synthesis of Substituted Fluorenones and Azafluorenones. *J. Org. Chem.* **1988**, *53* (15), 3513–3521.

(23) Chimenti, F.; Fioravanti, R.; Bolasco, A.; Chimenti, P.; Secci, D.; Rossi, F.; Yáñez, M.; Orallo, F.; Ortuso, F.; Alcaro, S.; Cirilli, R.; Ferretti, R.; Sanna, M. L. A New Series of Flavones, Thioflavones, and Flavanones as Selective Monoamine Oxidase-B Inhibitors. *Bioorg. Med. Chem.* **2010**, *18* (3), 1273–1279.
